# From the RNA-Peptide World: Prebiotic Reaction Conditions Compatible with Lipid Membranes for the Formation of Lipophilic Random Peptides in the Presence of Short Oligonucleotides, and More

**DOI:** 10.3390/life14010108

**Published:** 2024-01-09

**Authors:** Augustin Lopez, Antoine Vauchez, Ghinwa Ajram, Anastasiia Shvetsova, Gabrielle Leveau, Michele Fiore, Peter Strazewski

**Affiliations:** 1 Laboratoire de Chimie Organique 2 (LCO2), Institut de Chimie et Biochimie Moléculaires et Supramoléculaires (ICBMS, UMR CNRS 5246), Bâtiment Edgar Lederer, Université Claude Bernard Lyon 1, Université de Lyon, 1 rue Victor Grignard, 69100 Villeurbanne, Francemichele.fiore@univ-lyon1.fr (M.F.); 2 Centre Commun de la Spectrométrie de Masse (CCSM), ICBMS, Bâtiment Edgar Lederer, 1 rue Victor Grignard, 69100 Villeurbanne, France; antoine.vauchez@univ-lyon1.fr

**Keywords:** chimeras, DNA, hot springs, ligation, lipids, oligonucleotides, peptides, RNA, systems chemistry, translation, vesicles

## Abstract

Deciphering the origins of life on a molecular level includes unravelling the numerous interactions that could occur between the most important biomolecules being the lipids, peptides and nucleotides. They were likely all present on the early Earth and all necessary for the emergence of cellular life. In this study, we intended to explore conditions that were at the same time conducive to chemical reactions critical for the origins of life (peptide–oligonucleotide couplings and templated ligation of oligonucleotides) and compatible with the presence of prebiotic lipid vesicles. For that, random peptides were generated from activated amino acids and analysed using NMR and MS, whereas short oligonucleotides were produced through solid-support synthesis, manually deprotected and purified using HPLC. After chemical activation in prebiotic conditions, the resulting mixtures were analysed using LC-MS. Vesicles could be produced through gentle hydration in similar conditions and observed using epifluorescence microscopy. Despite the absence of coupling or ligation, our results help to pave the way for future investigations on the origins of life that may gather all three types of biomolecules rather than studying them separately, as it is still too often the case.

## 1. Introduction

The presence of three types of biomolecules (peptides, oligonucleotides and lipids) and their cooperation were probably necessary to explain the transition between non-living and animated matter [1,2,3]. Several interactions between the partners can be mentioned and might be involved in the origins of life. One of them is the peptide–oligonucleotide covalent coupling that would explain the appearance of an early translation, among other possible early functions for such chimeras [4]. One of these alternatives is the formation of amphiphilic peptide–oligonucleotide conjugates, viz., chimeras composed of a lipophilic peptide and a nucleic acid, that could allow for the invasion of oligonucleotides inside vesicles [5,6]. On the one hand, as chimeras, the hydrophilic oligonucleotides could maintain hydrophobic peptides in a solution that were likely formed from amino acids thought to have been abundant on the early Earth (glycine, alanine, valine, leucine) [7,8]. Such peptides would otherwise precipitate, be unable to grow further in size or exert hardly any catalytic activity on their surroundings. On the other hand, these lipophilic peptides could anchor to lipid membranes, thereby maintaining a high local concentration of covalently bound oligonucleotides in their vicinity [9]. Once present at or in the membranes, some of the chimeras could cross them and get inside the vesicles. Membrane defects and permeability variations are provoked by pH, osmotic pressure or temperature changes that might occur in prebiotic environments such as (not submarine) hot springs [10]. When inside their host vesicles, the oligonucleotides could grow in size, for instance through slow ligation, which would make them less prone to leak out. Their presence and that of lipophilic peptides could contribute to the persistence of the vesicles over time. For example, lipophilic peptides could rigidify labile vesicle membranes and refine, through pore formation, the selectivity of their permeability for certain molecules against others and provoke the separation of smaller vesicles through budding [11,12]. In this way, encapsulated genetic information could be carried over to daughter vesicles, which is very likely essential for the emergence of a biological system from a chemical system [13]. The location and stage of chemical evolution that would generate and host such starting molecules, also providing the necessary reaction conditions, could thus be hot springs or analogous evaporative surface environments that provided rivulets and lakes of aqueous solutions of amino acids, monomeric and short (oligomeric) nucleotides pooled on periodically dry surfaces, some hosting reactive small molecules that would chemically activate the amino acids and oligonucleotides (prebiotic dehydrating ‘coupling’ agents) and some periodically covered with a mixture of spontaneously formed lipids able to swell upon rehydration to liberate lipid vesicles and make them grow in size and numbers. Other similar scenarios that would not critically depend on autocatalytically replicating ribozymes are also thinkable [14].

Most of the published work on plausibly prebiotic peptide–oligonucleotide coupling studied the formation of amino acid–(oligo)nucleotides that would yet have to co-polymerise in order to yield peptide–oligonucleotides [15,16,17,18,19]. However, short oligonucleotides and short peptides were probably available on the early Earth [1,2,20,21]. They might have coupled upon activation with prebiotic condensing agents to generate chimeric conjugates. Here, we investigated the formation of peptide–oligo(deoxy)ribonucleotides, viz., amphiphilic peptide-RNA and peptide-DNA conjugates that would be able in part to invade lipidic vesicles, with a particular focus on peptido-RNA bearing a phosphoramidate linkage between a lipophilic peptide and RNA. Peptido-RNAs are particularly interesting in that regard, because they possess a P-N bond that is quite long-lasting in pH-neutral conditions or slightly alkaline but labile in acidic conditions. Vesicles formed under more acidic conditions, for example, containing citric acid in their lumen (inside volume), could be brought into contact with peptido-RNA at a neutral or slightly basic pH. This would prompt the disconnection and release of both parts after a potential invasion [22]. At high peptide concentrations, such a chimera formation, invasion and release mechanism would be catalysed, in the sense of turned over, by the more robust lipophilic peptides.

**Figure 1:**
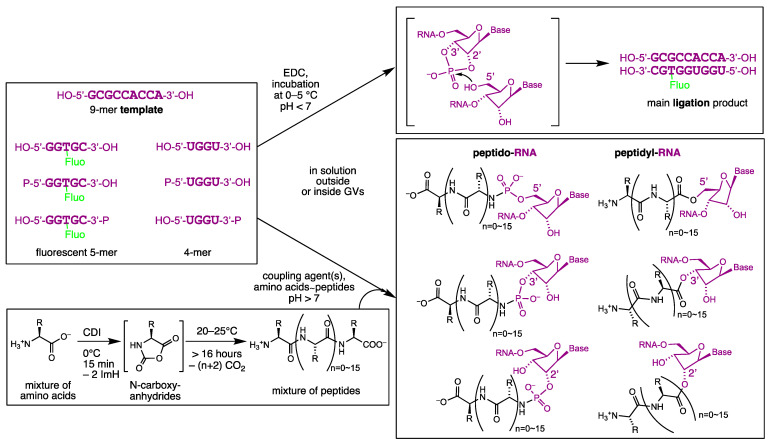
Work plan: RNA oligomers (**upper left box**, also DNA and DNA-RNA hybrid constructs) for their ligation (**upper right box**, also untemplated elongation) and their peptidylation with relatively lipophilic peptide mixtures (**lower left box**) to give amphiphilic peptide–oligonucleotide chimeras (**lower right box**) outside or inside the lumen of mixed-lipid giant vesicles (GVs). A = adenylate (or 2′-deoxyadenylate), G = guanylate (or 2′-deoxyguanylate), C = uridylate, T = thymidylate, Fluo = fluorophore, P = terminal phosphate, OH = terminal hydroxy group, EDC = 1-ethyl-3-(3-dimethylaminopropyl)carbodiimide, CDI = *N,N’*-carbonyldiimidazole, ImH = imidazole, R = H (Gly), CH_3_ (Ala), isopropyl (Val), isobutyl (Leu), CH_2_OPO_3_H_2_ (phosphoserine), CH_2_N_3_ (β-azido-alanine).

Our initial work plan is sketched out in Figure 1, according to which different oligonucleotides of a defined structure were to be ligated to furnish longer oligo(deoxy)ribonucleotides when hybridised (or not) to a template strand. In addition, the same oligonucleotides would be peptidylated instead of ligated; hence, chimeric peptide-RNA or peptide-DNA conjugates were expected to be observed at least transiently. These chimeras could be present in different isomeric forms, for instance, as hydrolytically more stable peptido-RNA (peptido-DNA), in which the peptides were connected through their N-terminus to the oligonucleotide’s terminal phosphate group either at their 5′- or 3′/2′-terminus depending on what kind of oligonucleotide would be furnished. Alternatively, isomeric peptidyl-RNA (peptidyl-DNA) chimeras could also be created where the peptide’s C-terminus would serve as a hydrolytically more labile carboxy ester linkage connected with either the 5′- or 3′/2′-hydroxyl terminus of the corresponding oligonucleotide. The 2′/3′-terminal linkage in peptidyl-RNA would model those present in natural peptidyl transfer RNA that appear transiently bound to the P site of ribosomes and are the growing point of every nascent peptide to become a protein outside, after its passage through the ribosome’s nascent peptide tunnel. In our experiments, the peptides to be linked to one generic oligonucleotide constitute a mixture of peptide lengths and amino acid sequences that then, in the form of chimeras, serve as a pool to be selected by giant vesicles (GVs) composed of a mixture of lipids. In prior preliminary experiments, we could hope for peptide lengths of up to 16-mers.

Hence, the prebiotic conditions tested here would have to be compatible with the presence of lipidic vesicles that might be invaded by such chimeric species. Indeed, some of the conditions previously reported would not be relevant in our scenario, since high concentrations of salts and divalent cations proved to be irreconcilable with the presence of most of the prebiotic lipidic vesicles [23,24]. First, random mixtures of (mainly) lipophilic peptides were generated from selected pools of prebiotic amino acids through activation with *N,N’*-carbonyldiimidazole (CDI) [25]. After characterization using mass spectrometry (MS), several attempts of coupling between these random peptides and synthesised RNA were performed in prebiotic conditions compatible with the presence of lipidic vesicles, as assessed using epifluorescence microscopy. The results of these couplings were analysed using liquid chromatography-MS (LC-MS). In addition, similar reaction conditions were tested in the absence of amino acids or peptides for the chemical ligation of almost completely complementary oligonucleotides (eight Watson–Crick pairs and one wobble mismatch).

## 2. Materials and Methods

*Production of random peptides from amino acid mixtures*: 200 mm l-amino acids in 1 mL of H_2_O/D_2_O (9:1 *v*/*v*) were analysed using NMR spectroscopy, then cooled down in an ice/water bath. Afterwards, the cold solution was added to dry, cold and solid CDI (500 mm final concentration). After 15 min of the reaction under agitation in an ice/water bath, the sample was analysed using NMR. Finally, the reaction was left for 64 h at room temperature under agitation before a last analysis using NMR was performed.

*Peptide–oligonucleotide coupling attempts*: Depending on the experiment, the conditions and the concentrations marginally varied. Essentially, the oligonucleotide of interest (pUGGU alias p4R, cf. Table 1) was activated for several hours in the presence of the condensing agent 1-ethyl-3-(3-dimethylaminopropyl)carbodiimide (EDC) and nucleophilic catalysts 1-ethylimidazole (EtIm) or 4-(dimethylamino)pyridine (DMAP). Then, random peptides were added and the reaction was left for several hours prior to being analysed using LC-MS. The reactions were performed at pH 7.5 or 8.0 without a buffer or agitation, in order to be as prebiotic as possible.

*Oligonucleotide templated ligation attempts*: Depending on the experiment, the conditions and the concentrations varied. Essentially, the oligonucleotides of interest (one 4-mer, one fluorescent 5-mer and one 9-mer) were activated in the presence of condensing agent EDC, and sometimes in the presence of the catalyst *N*-hydroxysulfosuccinimide (sulfo-NHS) and/or NaCl. After several hours of the reaction at ambient temperature, for three samples also after 10 months at 4–6 °C, the mixtures were analysed using high-performance liquid chromatography (HPLC) coupled with ultraviolet (UV) absorption and fluorescence emission detection and using LC-MS. The reactions were performed at pH 5.5 or 6.0 without a buffer or agitation, in order to be as prebiotic as possible.

*Vesicle production and microscopic analysis*: An equimolar ternary lipid mixture of lauric acid (LA), 1-monolauroyl glycerol (MLG) and 1,2-dilauroyl-*sn*-glycerol-3-phosphate (DLPA) was dissolved in 1 mL of MeOH (5 mm total lipid concentration) and evaporated under agitation to form a thin lipid film. After drying for several hours, the film was gently rehydrated with 1 mL of a buffer of interest for several hours. The vesicles were observed using epifluorescence microscopy thanks to a fluorescent lipid previously incorporated in minute amounts, 1,2-dioleoyl-*sn*-glycero-3-phosphoethanolamine-*N*-(lissamine rhodamine B sulfonyl (DOPE-RhB), or by adding low amounts of an ethanolic solution of Nile Red™ dye. See Supplementary Materials for additional information and details.

## 3. Results

### 3.1. Production and Characterisation of Mixtures of Random Peptides

**Figure 2:**
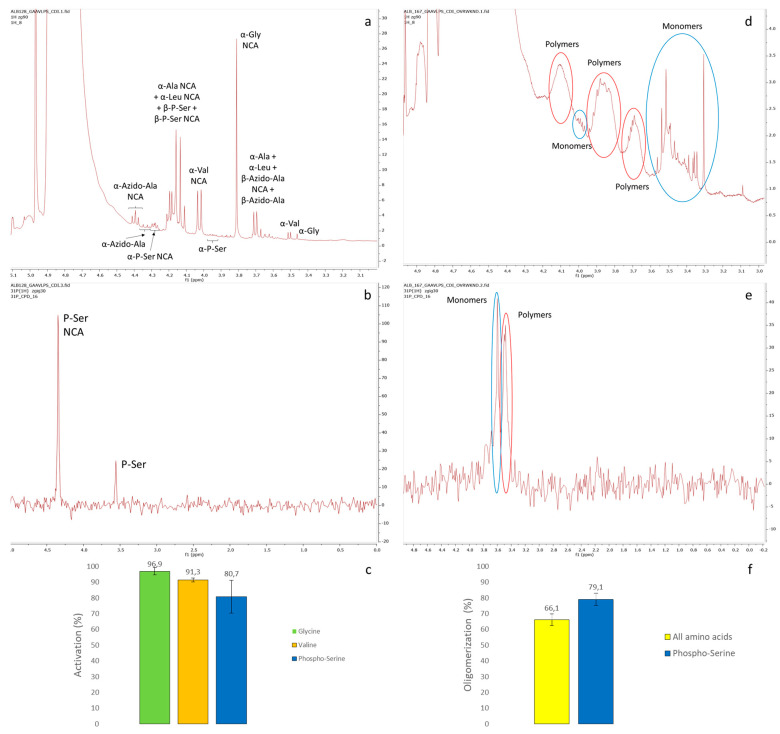
Activation and oligomerisation extents determined using NMR for different amino acids. (**a**) Exemplary ^1^H NMR spectrum (300 MHz) obtained from the GAVLPSAA mixture after 15 min of activation with CDI used to determine the activation extent of glycine and valine. (**b**) Exemplary proton-decoupled ^31^P{^1^H} NMR spectrum (120 MHz) obtained from the GAVLPSAA mixture after 15 min of activation with CDI used to determine the activation extent of phosphoserine. (**c**) Activation extents (%) of glycine, valine and phosphoserine determined using NMR from 3 independent experiments. (**d**) Exemplary ^1^H NMR spectrum (300 MHz) obtained from the GAVLPSAA mixture after 15 min of activation with CDI and 64 h of polymerisation used to determine the oligomerisation extent of all amino acids. (**e**) Exemplary ^31^P{^1^H} NMR spectrum (120 MHz) obtained from the GAVLPSAA mixture after 15 min of activation with CDI and 64 h of polymerisation used to determine the oligomerisation extent of phosphoserine. (**f**) Oligomerization extents (%) of all amino acids and phosphoserine determined using NMR from 3 independent experiments.

A common method to produce peptides from amino acids is the activation into N-carboxyanhydrides (NCAs) using CDI. This activating compound could stem from the reaction of imidazole with urea in an evaporative milieu and would provide one of several possible pathways for how short peptides could have emerged on the primitive Earth. NCAs could then polymerise amino acids with each other in slightly basic conditions (due to released imidazole) to yield quite long peptides (due to the transiently still N-carboxylated main chains) [26,27]. In order to produce prebiotic and lipophilic peptides, a specific composition of amino acids was chosen. Among the amino acids that were probably available on the early Earth [7,8], a maximally large proportion of valine (V) and leucine (L) was selected in order to obtain lipophilic peptides [28]. Lower amounts of glycine (G) and alanine (A) were added to avoid immediate precipitation of the peptides produced. The amino acids were activated with 2.5 equivalents of CDI at 0 °C, yielding activated NCAs after a few minutes, as assessed using ^1^H NMR spectroscopy. These amino acids were tested independently as NMR standards (Figures S1–S4). The activated amino acids could then slowly polymerise to yield peptides (Scheme S1). Several amino acid proportions were tested, in order to lower the proportion of precipitated peptides. For that, the weights of the finally formed soluble and precipitated peptides were compared. Since the extent of precipitation was around 19 weight% (w%) with a composition of 200 mm G:A:V:L 10:40:20:30 (mol ratios = mol/mol/mol/mol) (Figure S5), we decided to replace some of the alanine with a polar and charged prebiotic amino acid: *O*-phosphoserine (PS) [29]. In addition, this amino acid could chemically simulate the presence of charged (oligo)nucleotides that might couple with the peptides and maintain them in a solution. Moreover, contrarily to cationic amino acids containing a side-chain-terminal amine (such as Lys or Arg), only main-chain elongation and no side-chain elongation was expected to occur in the case of PS-containing peptides. Its addition in our combination of amino acids proved to yield more soluble peptides: around 4 w% of precipitation from 10 mol% PS and around 6 w% from 5 mol% PS (Figure S5). The activation of PS with CDI, in similar conditions as previously, was also tested independently as an NMR standard using ^1^H NMR (Figure S6). Later, some of the alanine was replaced by a non-prebiotic amino acid, β-azidoalanine (AA), producing peptides able to click with ring-strained cycloalkyne-functionalised molecules [30]. The activation of β-azidoalanine with CDI was independently tested as well, again in similar conditions as previously, as an NMR standard using ^1^H NMR spectroscopy (Figure S7). In the end, we selected to activate and polymerise a mixture of six 200 mm amino acids composed of glycine, alanine, valine, leucine, phosphoserine and β-azidoalanine, called GAVLPSAA, in the mol proportions 10:30:20:30:5:5 that should have a precipitation extent between 4 and 6 w%, as β-azidoalanine is more polar than alanine (see Figure S8 for the structure of the amino acids chosen). Using NMR, thanks to our standards (Figure 2, Figure S9 and S10), we could estimate the extent of amino acids that were effectively converted to NCAs and then oligomerised to peptides. For that, NMR spectra were taken before activation, after 15 min of activation with CDI and after 64 h of polymerisation (Figure 1 and Scheme S1). After 15 min, the formation of the NCA could be detected due to the chemical shifts of the protons of α-carbons (of all amino acids) or of the phosphorus nuclei (phosphoserine only). Thus, integrating the areas of both peaks (NCA or amino acid) for the amino acids obtained using ^1^H NMR (glycine and valine) or ^31^P NMR (phosphoserine) allowed us to determine their activation extents (Figure 2a–c). However, due to overlapping between the peaks, no activation extent could be determined for the other amino acids (alanine, leucine and β-azidoalanine). The activation extents obtained were around 97% for glycine, 91% for valine and 81% for phosphoserine. Such amounts showed that the activation into NCA was efficient and should enable a good polymerisation. Similarly, the proportion of amino acids that were effectively converted to peptides could be estimated in the same NMR window due to the molecular tumbling effect. The broad peaks corresponded to peptides and the sharp peaks to hydrolysed or not activated amino acids. Both types of peaks were integrated and allowed us to estimate the oligomerisation extent of all amino acids (using ^1^H NMR) and of phosphoserine only (using ^31^P NMR) (Figure 2d–f). Around 66% of the amino acids were polymerised into peptides and this extent depended on the type of amino acids, since about 79% of the phosphoserine oligomerised.

**Figure 3:**
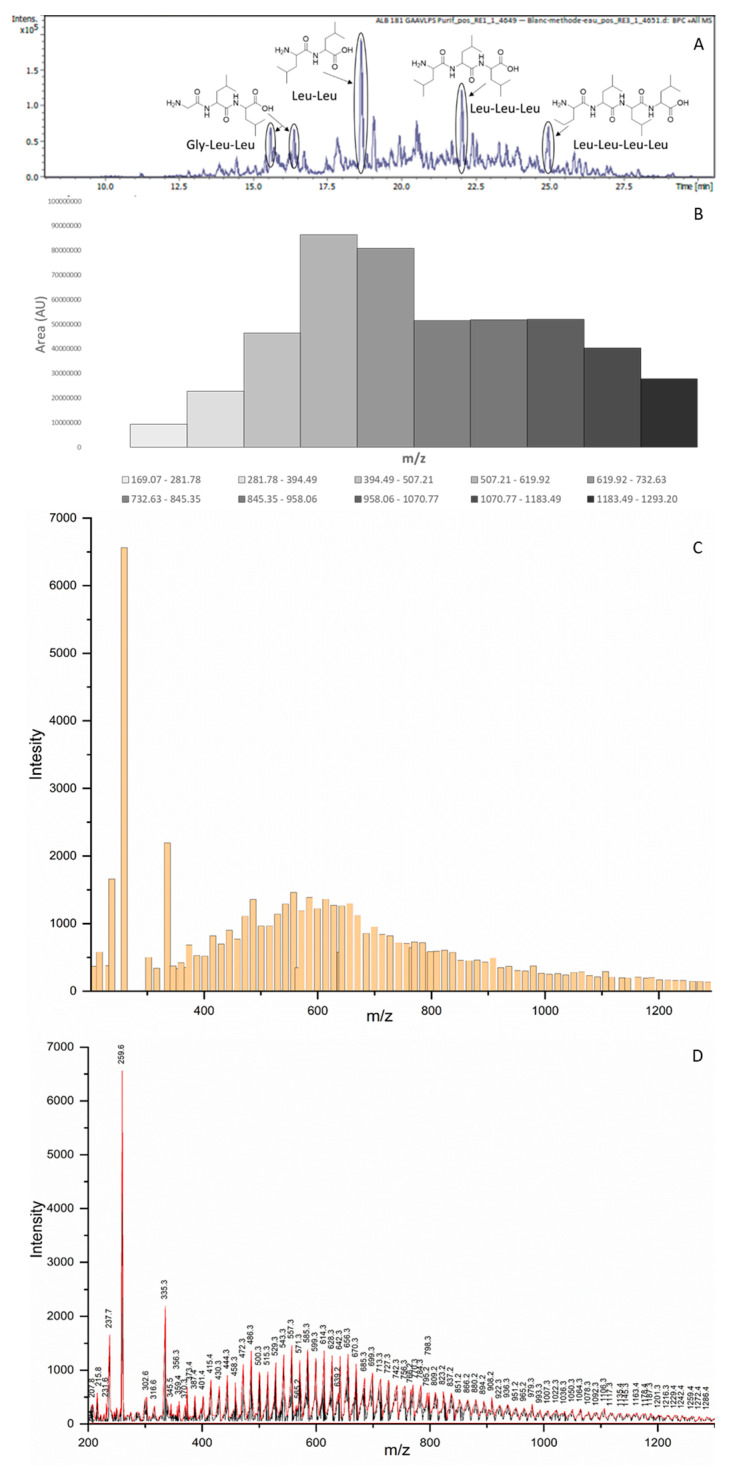
Mass spectrometry (MS) analysis and results of the soluble peptides obtained from CDI-activated GAVLPSAA mixtures in our conditions (Scheme S1). (**A**) LC-ESI-MS positive-ion chromatogram (RP-HPLC binary gradient elution with a = 5 mm NH_4_OAc (pH 8.0) and b = CH_3_CN/a 95:5) of the GAVLPSAA mixture after polymerisation mediated by CDI. The peptides present in the main (mono-charged) peaks are depicted (Gly-Leu-Leu could be also Leu-Gly-Leu and/or Leu-Leu-Gly). (**B**) LC-ESI-MS results shown as a histogram of the intensity taken from the area for the *m*/*z* signals detected in positive-ion mode of the extracted-ion chromatograms (EICs) divided into ten mass ranges (cf. legend of histogram and EICs in Figure S12) representing the estimated average masses of peptides generated from GAVLPSAA mixtures (2-mers to 11-mers). (**C**,**D**) MALDI-ToF-MS analysis on the same GAVLPSAA mixture, after polymerisation mediated by CDI and purification through a Sephadex G-10 column, on the supernatant isolated through centrifugation. The raw data (black line in (**D**)) were convoluted to depict groups of single peaks each within 5 Da (red line in (**D**)) and a histogram gathering populations of several peaks in the same *m*/*z* range (**C**), to be compared with (**B**).

In order to assess that the produced peptides were long enough to be lipophilic, we analysed them using MS. The most intense peaks detected using LC-MS corresponded to short peptides (2–4-mers) (Figure 3A and Figure S11). Intriguingly, these peaks corresponded to Leu-rich peptides. We assumed that it was due to an enhanced signal that this amino acid produced in mass spectra. Interestingly, it also meant that at least some of the peptides were also lipophilic and might serve as anchors on lipidic membranes, after coupling them with oligonucleotides. Nevertheless, these peptides were probably too small in that regard. We decided to determine the distribution of the produced peptides after their separation using liquid chromatography coupled to electrospray-ionisation mass spectrometry (LC-ESI-MS or simply LC-MS, Figure 3B) but also as a bulk mixture using matrix-assisted laser desorption (coupled to time-of-flight detection) mass spectrometry (MALDI-Tof-MS or simply MALDI-MS, Figure 3C,D), in order to more sensitively detect longer peptides [25]. The LC-MS was performed under ionisation conditions that strongly favoured single-positive-charge molecular ions. The total-ion chromatograms were used to create extracted-ion chromatograms (EICs) of the estimated average mass (size) ranges of short peptides produced from GAVLPSAA (from 2-mers to 11-mers). The result looked like a close to normal distribution with a majority of 5-mers of around 600 Da, but longer and more interesting peptides (up to 11-mers) were also detected (Figure 3B and Figure S12). In order to confirm this distribution, similar analyses were performed using MALDI-MS. For that, the samples were pre-purified using gel filtration in order to remove most of the imidazole that may bother the MALDI analysis and then separated into two portions, a supernatant and a precipitate, through centrifugation. The results obtained with the supernatant, thus, the peptides that might actually couple with oligonucleotides, were simplified in order to reduce the huge number of MALDI peaks detected. They were comparable with the peptides measured using LC-MS with a normal-like distribution showing a majority of peptides around 600 Da (≈5-mers) and longer peptides in smaller amounts (up to 1200 Da ≈ 11-mers) (Figure 3C,D). Thus, the supernatants were representative of the whole samples, which seemed logical as they represented around 95 w%. These features should be the ones to take into account for further couplings. The pellets of the precipitate were also analysed using MALDI-MS and, likewise, they showed a distribution similar to the one expected from a normal law. As expected, the peptides found were heavier, explaining their precipitation, with the most abundant peptides of about 800 Da (≈7-mers) and the longest of around 1600 Da (≈14-mers) (Figure S13). Interestingly, long and lipophilic peptides were indeed produced through polymerisation mediated by CDI but these precipitated peptides represented a kind of waste as they could probably no longer couple with the oligonucleotides.

**Figure 4:**
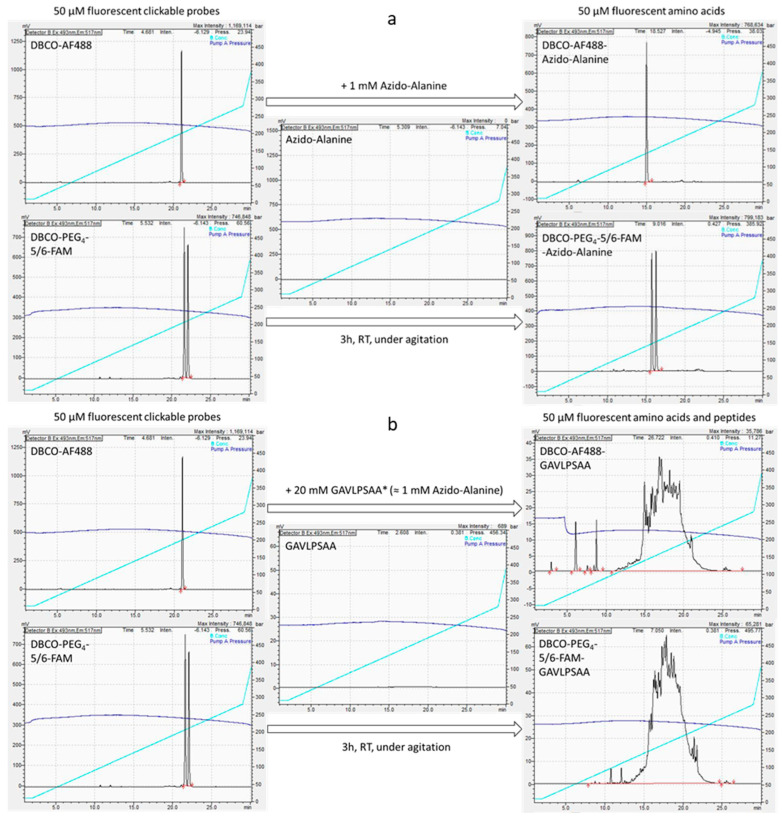
HPLC chromatograms with fluorescence detection at 493/517 nm excitation/emitted wavelength (black: emitted) of click reactions between fluorescent probes and (**a**) β-azidoalanine (as a control) and (**b**) GAVLPSAA peptides. RP-HPLC binary gradient elution with A = 5 mm NH_4_OAc (pH 8.0) and B = CH_3_CN/A 95:5 (light blue line: B concentration, dark blue: pump A pressure). (**a**) The sample was injected before and after the click reaction of β-azidoalanine (1 mm, 3 h, rt, slow agitation at 300 rpm) with 50 µm fluorescent clickable probes (DBCO-AF488 or regio-isomeric DBCO-PEG_4_-5/6-FAM). The peaks shifted to shorter retention times, thus showing that the click reaction occurred with an almost 100% yield and produced clicked products more polar than the unclicked probes. (**b**) The sample was injected before and after the click reaction of GAVLPSAA peptides (20 mm ≈ 1 mm β-azidoalaninyl residues, 3 h, rt, slow agitation at 300 rpm) with 50 µm fluorescent clickable probes (DBCO-AF488 or DBCO-PEG_4_-5/6-FAM). “Forest” of peaks were obtained, showing all the GAVLPSAA peptides containing β-azidoalanine residues, which clicked with the fluorescence probes, yielding numerous fluorescent peptides. * The concentration of GAVLPSAA peptides could only be estimated, since the mixtures contained random peptides, and amino acids and imidazole were also added with the peptides.

Another interesting feature of these random peptides was to make sure that some of them indeed contained β-azidoalanine and could click to a cycloalkyne-functionalised fluorophore, such as the more hydrophilic (di-sulfonyl) DBCO-AF488 and the more lipophilic DBCO-PEG_4_-5/6-FAM. First of all, a control reaction was carried out with the pure amino acid β-azidoalanine (Scheme S2). For that, a 50 µm solution of each fluorophore probe was independently incubated with 1 mm β-azidoalanine for 3 h at room temperature under slow agitation (300 rpm). The result of the reaction was analysed using reversed-phase HPLC (RP-HPLC) coupled with fluorescence detection. It appeared that the retention time of the fluorescent compounds was effectively reduced after their reaction with β-azidoalanine, which showed that the click reaction yielded the clicked fluorescent amino acids that were more polar than the unclicked probes (Figure 4a). Given this, click reactions were performed with GAVLPSAA mixtures using similar concentrations (20 mm GAVLPSAA peptides ≈ 1 mm β-azidoalanine and 50 µm fluorescent probe). The concentration of GAVLPSAA peptides was only an estimation as the mixture contained random peptides but also salts, imidazole and amino acids; see Supplementary Materials for further details about the calculation of peptide concentrations. After the reaction in similar conditions, we could observe using RP-HPLC that a multitude of fluorescent compounds were produced in a “forest” of peaks, highlighting the diversity of all the peptides containing at least one β-azidoalanine initially present and now clicked to the fluorescent probes (Figure 4b). Spiking injections were performed and showed that clicked (monomeric) β-azidoalanine or not-clicked probes co-eluted in these forests, but the majority of the fluorescent compounds corresponded to other compounds, to clicked-GAVLPSAA peptides (Figure S14). Furthermore, we investigated the possibility of generating even more clicked peptides, as most of the β-azidoalanine (around 950 µm) should still be available after this first reaction. Thus, another reaction was performed, adding more of the fluorescent probe (50 µm) to these clicked peptides. After incubation overnight in similar conditions, the chromatograms clearly showed that most of the subsequently added probe was once again converted to fluorescent peptides through the click reaction (Figure S15). Given this, we knew for certain that some of the peptides present in the GAVLPSAA mixture contained one or several β-azidoalanine residues that could click to fluorescent probes. After these extensive characterisations, we could use these peptides for coupling experiments with oligonucleotides. The GAVLPSAA mixtures contained peptides but also amino acids and imidazole. They were used as such for coupling experiments, in order to be as representative as possible of a prebiotic soup.

### 3.2. Coupling Attempts of Peptides with Oligonucleotides in Prebiotic Conditions Compatible with the Presence of Lipidic Giant Vesicles

#### 3.2.1. Conditions Compatible with the Presence of Lipidic Giant Vesicles

As previously reported in the literature, the coupling between peptides/amino acids and (oligo)nucleotides might occur upon the activation of the terminal phosphate with a condensing agent such as EDC widely used in prebiotic chemistry experimentation. Such activated terminal phosphates could be targeted by the amino group of peptides/amino acids, leading to the release of hydrolysed EDC, being the corresponding urea EDU, and yielding peptido-oligonucleotides (Figure 1 and Scheme S3). EDU is cleaved from O-isoureyl phosphate esters (the EDC-activated oligonucleotides) but these are known to slowly isomerise to the much less reactive N-ureyl phosphoramidates. To suppress this, a nucleophilic catalyst was added, such as EtIm or DMAP, in order to form another activated intermediate from the initially activated EDC-oligonucleotide, offering an even better leaving group than EDU. The above tertiary amines were expected to transiently generate EtIm-oligonucleotides or DMAP-oligonucleotides bearing preferentially 5′-terminal zwitterionic quaternary ammonium phosphate groups (EtIm⊕–PO_2_⊖–O-C5′ or DMAP⊕–PO_2_⊖–O-C5′) that would yield peptido-oligonucleotides upon their aminolysis by the peptides. In order to investigate this reaction, it was first of all necessary to produce an oligonucleotide that would be used for all couplings experiments. We decided to synthesise the short 5′-phosphorylated RNA pUGGU as it might be small enough to cross lipid membranes after coupling with a lipophilic peptide. This nucleic acid was produced using solid-supported synthesis, deprotected manually [31] and purified using semi-preparative RP-HPLC. The yield was calculated with OligoCalc™ from the absorbance measured ultraviolet (UV)-wise at 260 nm (see Figure S16 for additional details in the Supplementary Materials). Once this oligonucleotide was available in sufficient amounts, we could investigate its coupling with our random-sequence peptide mixtures. One requirement for the successful coupling of peptide–oligonucleotide chimeras in our prebiotic scenario would be the compatibility of this reaction with the presence of lipidic giant vesicles that might be invaded by these amphiphilic compounds. Some conditions were previously reported and we decided to adapt those by using low concentrations and removing any element that might be deleterious for lipidic vesicles. This is why all coupling experiments were performed with 50 µm pUGGU (≝1 eq.). In addition, all our experiments were performed without a buffer or mechanical agitation, in order to be as prebiotic as possible.

**Figure 5:**
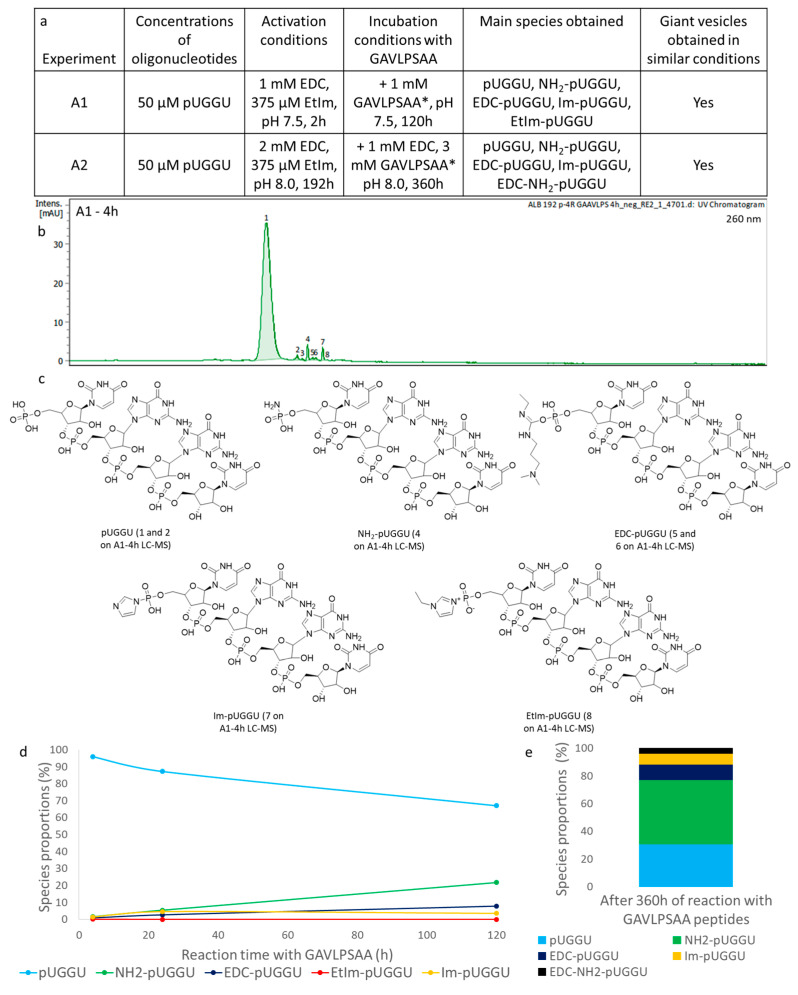
Coupling attempts between pUGGU and GAVLPSAA peptides, to form peptido-oligonucleotides, in the presence of EDC and EtIm in 2 experiments, A1 and A2. (**a**) Presentation of the experiments A1 and A2. For each experiment, the activation and the incubation conditions are reported with the main species obtained and detected using LC-MS (see Figure 5c for the structures of the most represented compounds and Figures S17–S20 for the others). Im stands for imidazole whereas EtIm stands for 1-ethylimidazole. The formation of giant vesicles (GVs) was investigated in conditions similar to both experiments and GVs were indeed observed (see Figure 5 for further details and GV pictures). * The concentration of GAVLPSAA peptides could only be estimated since the mixtures contained random peptides, amino acids and imidazole. (**b**) Exemplary UV chromatogram at 260 nm (RP-HPLC binary gradient elution with A = 5 mm NH_4_OAc (pH 8.0) and CH_3_CN/A 95:5) of the A1 experiment after 4 h of reaction. LC-MS (negative ion mode) and UV chromatograms (260 nm) of the other coupling experiments are displayed in the Supporting Information (Figures S17–S20). (**c**) The structures of compounds associated with the UV peaks (annotated in (**b**)). In EDC adducts, the linkage to the P-atom can also be through one of the urea N-atoms (N-ureyl phosphoramidate structure not shown here, see Figures S17 and S20). (**d**) Decay, growth and final composition of the species obtained from the coupling between pUGGU and GAVLPSAA peptides for A1 and (**e**) final composition of the A2 experiments. See Figure 5c for the structures of the main species. These ratios were calculated using the UV (260 nm) absorbance of the corresponding compound peaks detected using LC-MS analysis (Figures S17–S19 for A1 and Figure S20 for A2 experiments).

The first coupling experiments were carried out in conditions that we adapted from Clemens Richert and co-workers using EDC, a proxy for carbodiimides derived from the tautomerisation of cyanamide in prebiotic conditions [32], and 1-ethylimidazole (EtIm), as imidazole derivatives were commonly considered as being prebiotically available [33]. Thus, for 50 µm RNA, 1 mm EDC and 375 µm EtIm were used for the activation of the 5′-phosphate of pUGGU at pH 7.5 [34]. After 2 h in these conditions, around 1 mm soluble peptides, obtained from the GAVLPSAA polymerisation, were added. The reaction was then left for 120 h and was periodically analysed after 4, 24 and 120 h using LC-MS (experiment A1, Figure 5a and Figure S17–S19). In addition to unreacted RNA, most of the species corresponded to activated compounds (such as EDC-pUGGU or EtIm-pUGGU). Of note, the imidazole (Im) present in the GAVLPSAA mixture could also react to generate activated Im-pUGGU. These activated compounds may also couple with peptides despite their lower hydrolytic stability when compared to EtIm-pUGGU. The primary phosphoramidate H_2_N-pUGGU was detected using LC-MS and its amount seemed to increase over the reaction time, while the concentrations of the other activated compounds remained the same (Figure 5b,c). We suspected that this compound, having the molecular mass of H_2_N-pUGGU, originated from the reaction between ammonia, from ammonium acetate present in the LC-MS analysis buffer, and activated RNA or even peptido-RNA. In order to confirm this hypothesis, we tried to perform the RP-HPLC analysis without an ammonium buffer but the separation was not good enough to allow for the detection of any oligonucleotide. With the aim of kinetically favouring the aminolysis by peptides (instead of ammonia), the reaction conditions were optimised in another reaction (experiment A2). For that, the RNA was incubated with twice as much EDC, with respect to the A1 experiment, during a longer period of activation in the presence of EtIm and at a higher pH of 8.0 that should increase the aminolysis (peptidolysis) efficiency. After this enhanced RNA activation over 192 h, the amount of added peptides was tripled with respect to the previous A1 experiment. In addition, additional EDC was added together with the peptides, in order to let it react with remaining non-activated oligonucleotides, since EDC was expected to hydrolyse after a few days in the solution [35] (experiment A2, Figure 5a). Even though these improvements were carried out to favour the coupling reaction, no peptido-RNA or peptidyl-RNA was detected using LC-MS after 360 h of reaction time and despite the presence of peptides detected using LC-MS (for instance, β-azidoalanine-valine and β-azidoalanine-leucine dipeptides were observed using LC-MS, Figure S20). The compounds found were similar to the ones previously observed in the A1 experiment and in comparable amounts (Figure 5a,d). Interestingly, more H_2_N-pUGGU was detected after a longer reaction period of time: around 46% after 360 h in the A2 experiment (Figure 5d) compared to 21% after 120 h in the A1 experiment (Figure 5c). We assumed that ammonia from the HPLC buffer could replace progressively more activated or peptide-coupled oligonucleotides.

**Figure 6:**
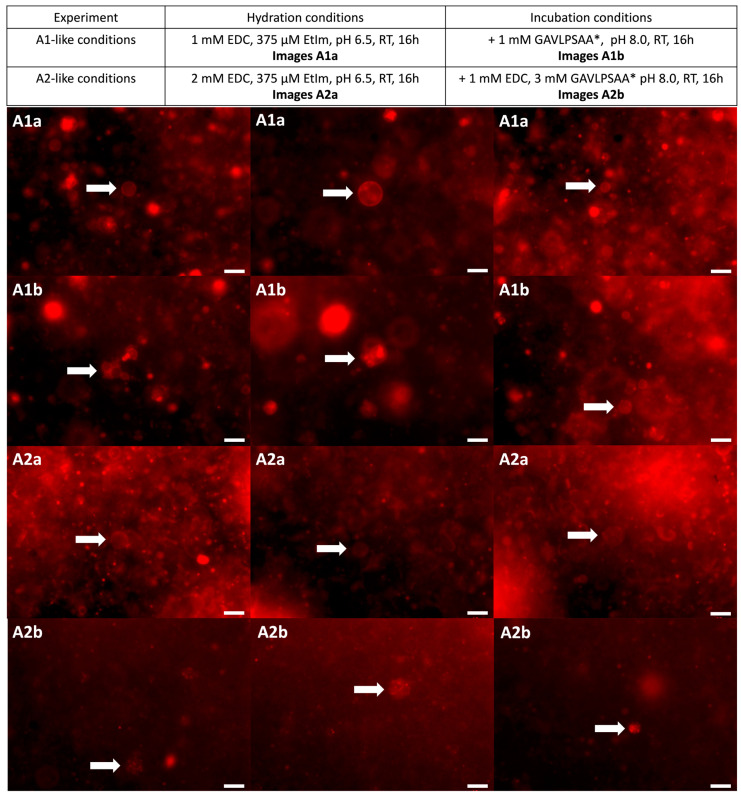
The formation of giant vesicles (GVs) was assessed in conditions similar to **A1** and **A2** experiments (measured osmolality up to 0.3 Osm/kg). Giant vesicles were observed using epifluorescence microscopy in all the cases (white arrows mark representative, in part multivesicular GVs). * The concentrations of the GAVLPSAA peptides added could only be estimated. GVs were observed in A2-like conditions after activation and incubation even though they were mainly giant vesicles encapsulating other giant vesicles in the second case. These observations were carried out with a red channel (543 nm) on a Carl Zeiss inverted microscope, Observer Z1, equipped with 20×, 50× and 100× oil immersion objectives and AxioCam recording. The scale bars represent 20 µm on all the images.

Despite the absence of coupled peptide–oligonucleotides, we wanted to make sure that our conditions were effectively compatible with the presence of lipidic giant vesicles that could be later invaded by these compounds. For that, we selected a composition of short-chain lipids by blending phosphorylated and non-phosphorylated compounds, i.e., an equimolar ternary mixture containing lauric acid (LA), 1-monolauroyl glycerol (MLG) and 1,2-dilauroyl-*sn-*glycerol-3-phosphate (DLPA), thus, LA:MLG:DLPA (1:1:1 mol/mol/mol/mol). Such composition would be at the same time relevant in a prebiotic context and able to form vesicles, gathering fluidity and robustness [36,37]. The formation of vesicles for this composition was tested through gentle hydration, applying the “natural swelling” method [38] that might simulate the formation of vesicles in hot springs [10]. In order to evaluate the formation of vesicles in our conditions, lipidic films were rehydrated with similar buffers as the ones used for the activation steps of oligonucleotide–random-peptide mixtures. The only difference was a slightly lower pH of 6.5, so that the lumen of the formed vesicles would be slightly more acidic, leading to the accelerated hydrolysis of the phosphoramidate groups of those chimeras that might invade these vesicles. After hydration for 16 h at room temperature, the formed vesicles were observed using epifluorescence microscopy thanks to minute quantities of fluorescent DOPE-RhB phospholipids initially added during the hydration step. Both conditions (A1 and A2 experiments) were conducive with the formation of vesicles (Figure 6). Simulating the second step with the addition of peptides, GAVLPSAA mixtures were also added to these vesicles together with EDC (for A2-like conditions only) in similar concentrations as the ones used for the incubations in A1 and A2 experiments. The pH was also adjusted to 8.0 to simulate the more basic environment present outside the vesicles where the RNA-peptide couplings could occur. After incubation for 16 h at room temperature, vesicles could still be observed in both cases (Figure 6). Interestingly, it seemed that the conditions with higher concentrations (A2 experiment) preferentially led to the formation of multivesicular objects. Based on these observations, and previous ones, we assumed that the high concentration of EDC was responsible for this phenomenon (Figure 6).

#### 3.2.2. Forced Coupling Conditions

**Figure 7:**
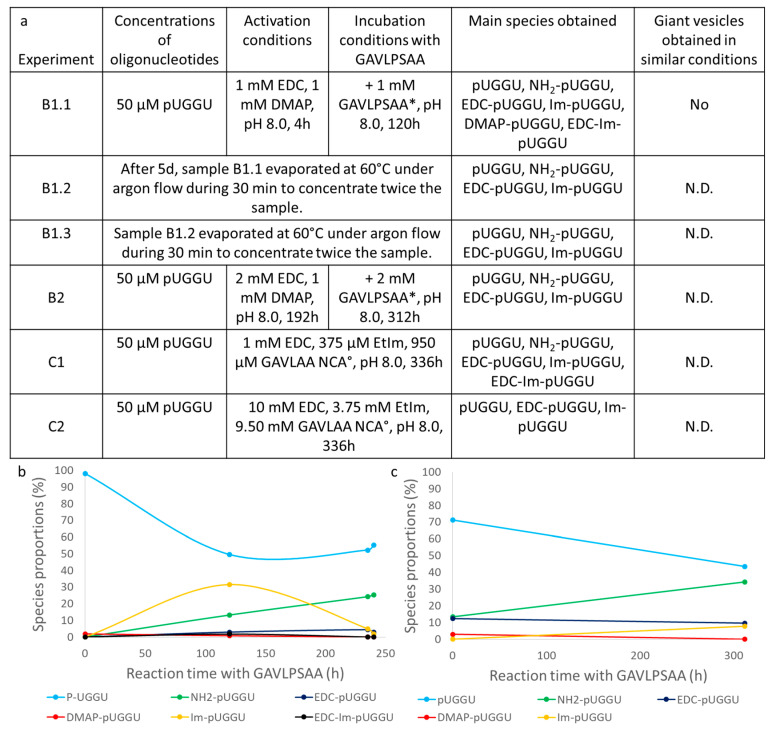
Coupling attempts between pUGGU and GAVLPSAA peptides/NCA, to form peptido-oligonucleotides, in the presence of EDC and DMAP/EtIm in 4 experiments, B1, B2, C1 and C2. (**a**) Presentation of the experiments B1, B2, C1 and C2. For each experiment, the activation and the incubation conditions are reported with the main species obtained detected using LC-ESI-MS (see Figure 5c for the structures of the main compounds and Figures S21–S28 for the others). Im stands for imidazole, DMAP stands for 4-(dimethylamino)pyridine and EtIm stands for 1-ethylimidazole. The formation of giant vesicles (GVs) was investigated in conditions similar to experiment B1 and was not fruitful. The formation of GVs was not determined in the other cases (N.D.). * The concentration of GAVLPSAA peptides could only be estimated since the mixtures contained random peptides, amino acids and imidazole. ° GAVLAA NCA were prepared as described previously (see also Scheme S1) and directly placed in the presence of oligonucleotides, EDC and EtIm. Growth and decay of the species obtained from the coupling between pUGGU and GAVLPSAA peptides for (**b**) B1 and (**c**) B2 experiments. These ratios were calculated using the UV (260 nm) absorbance of the corresponding peaks detected using LC-MS analysis (see Figures S21–S24 for experiment A1 and Figures S25 and S26 for experiment A2). Of note, the amounts of EDC-pUGGU appear slightly overestimated for experiments B1 and B2 since the EDC-pUGGU peaks co-eluted with other minor compounds.

With the aim of forcing the formation of peptide–oligonucleotide chimeras, several other approaches were attempted. One possibility was to use another nucleophilic catalyst, as EtIm was a prebiotic catalyst but perhaps not efficient enough. This is why a proxy was used with DMAP replacing EtIm. This new catalyst was placed in higher concentrations: 1 mm DMAP (compared to 375 µm EtIm), meaning as much as the condensing agent EDC [39]. For the rest, the conditions used for this experiment (B1.1, Figure 7a) were similar to the first experiment (A1) except for a longer period of RNA activation (4 h) and pH 8.0, which should both favour the coupling reaction. In that case, the compounds found after activation or after 120 h of incubation were still only activated oligonucleotides or the phosphoramidate generated from the reaction with ammonia during the RP-HPLC analysis (Figures S21 and S22). Trying to enhance the reaction, the same sample was concentrated to reach half of its initial volume through evaporation under argon flow at 60 °C, simulating a terrestrial hydrothermal environment [40]. This was performed twice (experiments B1.2 and B1.3) but no new compounds or conjugates were detected using LC-MS (Figures S23 and S24). Interestingly, it seemed that, after a maximum, the amount of the activated Im-pUGGU decreased whereas, as previously observed with EtIm, the concentration of H_2_N-pUGGU gradually increased to reach a maximum level of about 25% of the oligonucleotide derivatives (Figure 7b). As performed earlier, more EDC was added from the start (2 mm) with a longer period of time for the activation (192 h) in order to enhance the coupling reaction. These changes seemed to bear fruit as more activated compounds (EDC-pUGGU, Im-pUGGU, DMAP-pUGGU and H_2_N-pUGGU originating from activated oligonucleotides) were observed using LC-MS after this activation (around 29%, experiment B2, Figure 7c) when compared to the previous experiment (around 2%, experiment B1.1, Figure 7b). In addition, as conducted before in the experiments performed with EtIm, more GAVLPSAA peptides were added (around 2 mm) and the incubation was left for a longer period of time (312 h) (Figure 7a). Nevertheless, even after this extended incubation, the compounds detected using LC-MS were similar to the ones previously observed and in comparable amounts except for H_2_N-pUGGU (Figure 7c, Figure S25 and S26). Once again, it seemed that the longer the reaction, the higher the concentration of these ammonia adducts. No peptide-RNA conjugates were observed even though some short peptides could be detected using LC-MS (Figure S26).

Despite the absence of chimeric peptide–oligonucleotides, we wanted to see if these conditions tested in the B1 experiment were still compatible with the presence of prebiotic lipidic vesicles. They were prepared similarly as before, in the case of EtIm experiments, but no GVs could be observed using epifluorescence microscopy. Thus, it seemed that DMAP was conflicting with the presence of prebiotic lipidic vesicles (see Supplementary Materials for further details) and was no longer used for coupling experiments. At that point, we assumed that one reason why no coupling occurred was because the activation of the peptides was not good enough and that activated amino acids (NCA)/peptides should be directly placed with activated oligonucleotides. Willing to confirm this hypothesis, NCAs were produced in the presence of the same oligonucleotide pUGGU in similar conditions to the C1 experiment (C3, Figure 7a) or in higher concentrations when compared to the C2 experiment (C4, Figure 7a). Higher concentrations were also used, in order to increase the coupling rate. Since the amino acids were already activated, no further activation period was used in both cases. As a subtility, in contrast to the previous coupling attempts, the equivalent of phosphoserine (50 µm) was replaced with the oligonucleotide that could directly be attached to the NCAs generated from the overall 950 µm GAVLAA mixture (10:30:20:30:5, no PS). However, even in this case, no conjugate was detected using LC-MS. The species found were the same as previously. Thus, it seemed that the activation of the amino acids/peptides could not solve this coupling issue. The GAVLPSAA peptides might be too diluted even though some peptides were effectively detected in the solution (for instance, β-azidoalanine-valine and β-azidoalanine-leucine dipeptides were both observed using LC-MS, Figure S20). Another possibility, as already mentioned, would be that the primary phosphoramidate H_2_N-pUGGU, formed during the RP-HPLC analysis through the reaction of activated oligomers with ammonia from the LC-MS eluant, could replace the peptides that had been previously attached to the 5′-phosphates after all. Finally, it cannot be rigorously excluded that, since random peptides were used, the concentration of each peptide was very low and, even if couplings occurred, the concentration of the produced peptido-RNA was too low to be detected using LC-MS.

### 3.3. Templated Ligation Attempts between Activated Complementary Oligonucleotides in Prebiotic Conditions Compatible with the Presence of Lipidic Giant Vesicles

#### 3.3.1. Design and Synthesis of the Oligonucleotides Used

Irrespective of the presence of lipophilic peptides, we asked ourselves whether it would be possible to elongate oligonucleotides through templated ligation [41,42,43,44,45] under conditions that would be compatible with lipid membranes. Indeed, numerous achievements were carried out on this topic during the past decades but few of them can be considered as prebiotic and/or compatible with the presence of lipidic vesicles despite their importance for the origins of life [46]. High concentrations of salts and divalent cations were bread-and-butter conditions for any condensation reaction involving oligonucleotides, since they favoured their hybridisation to complementary double strands needed for templated and primed strand elongation or ligation. However, these conditions would probably be fatal to most prebiotic lipid vesicles [23,24]. It seemed natural to presume that conditions similar to the ones used for the coupling attempts between peptides and oligonucleotides would be compatible with the presence of lipidic vesicles. We noticed that 5′-phosphorylated RNA was commonly used and reported in the literature, whereas RNA-2′,3′-cyclic phosphates were most likely produced in prebiotic conditions, from direct synthesis [47] or nucleoside phosphorylation [48,49]. Furthermore, artificial (unnatural) 5′-amino-5′-deoxy-, 2′-amino-2′-deoxy- and 3′-amino-3′-deoxyribonucleotides were commonly used to favour the ligation or elongation of sometimes encapsulated oligonucleotides [43,44,45,47,48] even though neither the formation of these much more nucleophilic (5′-, 2′- or 3′-) “aminodeoxyribonucleotides” from simpler precursors nor their transformation to the regular ribosyl ones has been achieved under plausibly prebiotic conditions, nor is this issue sufficiently discussed in the literature.

In order to remain as prebiotic as possible, we decided to investigate the nonenzymatic templated ligation of oligonucleotides involving 2′,3′-cyclic phosphates. Such short oligonucleotides could form from the monomers on the early Earth [20,50,51,52,53,54] and be available for ligation reactions. To study the ligation of a 2′,3′-cyclic phosphate oligonucleotide with a 5′-OH oligonucleotide (Figure 1 and Scheme S4), we designed and synthesised in addition to the previously used pUGGU (alias p4R) the following oligonucleotides: both 2′-deoxyribo- (D) and ribo-congeners (R), as well as hybrid (H) DNA-RNA nona-, tetra- and fluorescent pentanucleotides (5′-D/R/H-3′)—9D, 9R, 4Dp, p4D, 4Hp, 4Rp, p4R, 5Dp, p5D, 5Hp, 5Rp and p5R (see Table 1). The 3′-phosphorylated 4-mers and 5-mers could ligate, ligating best when hybridised to terminally not-phosphorylated 9-mers, to yield the almost fully complementary fluorescent phosphorylated 9-mers or, less likely, longer and/or untemplated ligation products. These tetra- and pentanucleotides could be short enough to be able to cross lipidic bilayers and long enough to permit the formation of a strong enough duplex to enhance their ligation through the template effect of the hybridised 9-mer. The most important ligation product most likely to form in prebiotic conditions should be 9Rf>p (Table 1) because RNA oligomers, when activated, generate terminal 2′,3′-cyclic phosphates and RNA-RNA duplexes are somewhat more stable than DNA-DNA duplexes. Comparisons were planned to detect 9Hfp, a perhaps less likely ligation product that would form from 2′,3′-cyclic phosphates as well but duplexed less regularly in terms of conformation of the double helix; then, 9Dfp, perhaps p9Rf and p9Df, all resulting from chemically activated terminal, acyclic phosphate monoesters, would be used not only to furnish kinetic data and confirm already known chemical mechanisms, but mainly to provide deeper insight into realistic competitive scenarios in modelled prebiotic environments.

**Table 1:** Twelve oligonucleotides synthesised to study the templated ligation (Figure 1 and Scheme S4) with their associated nomenclatures. Last row: complementary fluorescent 9-mers expected from the templated ligation of a 4-mer and 5-mer duplexed with a 9-mer template through eight Watson–Crick pairs and one GT wobble mismatch. P (alias p) = terminal phosphate, cP (alias >p) = 2′,3′-terminal cyclic phosphate, d (alias D) = 2′-deoxy, OH = terminal hydroxy group, f = fluorescent thymidylate (5/6-FAM bound through C5 of T, from the commercial phosphoramidite monomer). The fact that the fluorescent thymidylate (_f_T) bears a 2′-deoxyribose is not shown in the right column.

5′-D/H/R-3′	DNA	Hybrid	RNA
Template 9-mers	HO-5′-d(**GCGCCACCA**)-3′-OH 9D		HO-5′-**GCGCCACCA**-2′,3′-(OH)_2_ 9R
Ligation partner: 4-mers	HO-5′-d(**TGGT**)-3′-P 4Dp	HO-5′-d(**TGG**)**U**-3′-P 4Hp	HO-5′-**UGGU**-3′-P 4Rp
	P-5′-d(**TGGT**)-3′-OH p4D		P-5′-**UGGU**-2′,3′-(OH)_2_ p4R
Ligation partner: Fluorescent 5-mers	HO-5′-d(**GG_f_TGC**)-3′-P 5Dp	HO-5′-d(**GG_f_TG**)**C**-3′-P 5Hp	HO-5′-**GG_f_TGC**-3′-P 5Rp
	P-5′-d(**GG_f_TGC**)-3′-OH p5D		P-5′-**GG_f_TGC**-2′,3′-(OH)_2_ p5R
Main ligation products: Fluorescent 9-mers	HO-5′-d(**TGGTGG_f_TGC**)-3′-P 9Dfp (from 4Dp + 5Dp + 9D) P-5′-d(**TGGTGG_f_TGC**)-3′-OH p9Df (from p4D + p5D + 9D)	HO-5′-d(**TGG**)**U**d(**GG_f_TGC**)-3′-P 9Hfp (from 4Hp + 5Dp + 9D)	HO-5′-**UGGUGG_f_TGC**-2′,3′-cP 9Rf>p (from 4Rp + 5Rp + 9R) P-5′-**UGGUGG_f_TGC**-2′,3′-(OH)_2_ p9Rf (from p4R + p5R + 9R)

These primary sequences were precisely designed. First of all, the 3′-terminal sequence of the template 9-mer contained the conserved 3′-end of all elongator transfer RNAs (ACCA) that is single-stranded in the natural context and enzymatically linked to (‘charged with’) one of the proteinogenic amino acids to furnish a ‘cognate’ aminoacyl-tRNA. We recognised like others that this tRNA sequence might have an important bearing in the prebiotic world, for both the formation of peptide–oligonucleotide chimeras and, consequently, as a likely molecular starting point for the self-evolution of translation [55]. Second, the 5′-terminal sequence of the 9-mer was designed in order to maximise the stability of pairing of the two partner strands with a high number of nucleotides (GCGCC) that would each establish three hydrogen bonds with their non-palindromic (not self-complementary) complement. Third, the size of the 9-mer corresponded to the size of three triplet codons. Fourth, a modified (commercial) fluorescent nucleotide was used as a tracker to detect even low amounts of the complementary 9-mer produced through ligation. This deoxynucleotide (the fluorescent thymidine derivative _f_T) was placed in the middle of the 5-mers in order to reduce as little as possible the stability of the oligonucleotides on their template when compared to the fully complementary 5-mer GGCGC. Finally, the sequences would not allow self-hybridisation or the formation of a stable hairpin loop that could prevent intermolecular reactions of interest.

In order to perform the ligation passing through 2′,3′-cyclic phosphates, 3′-phosphorylated 4-mers and 5-mers but terminally not-phosphorylated 9-mers were synthesised. In addition, to highlight the contribution of the cyclic phosphate and to test the difference between DNA and RNA (B and A forms of the corresponding double strands), hybrid nucleic acids were synthesised for all three ligation partners. DNA would not be able to yield 2′,3′-cyclic phosphates as the 2′-terminal OH is missing, but could be ligated through the chemical activation of their 3′-terminal phosphates. Hybrid 4-mers and 5-mers, which were mostly DNA but terminating with a 3′-ribonucleotide, thus providing a 2′-terminal OH able to yield 2′,3′-cyclic phosphates, were also produced. As additional controls, 5′-phosphorylated DNA and RNA 4-mers and 5-mers were synthesised as well. They could have been used to see whether a ligation occurred without forming 2′,3′-cyclic phosphates, but through the chemical activation of their 5′-terminal phosphates. All these oligonucleotides (Table 1) were synthesised on a 6 µmol scale, deprotected and purified similarly to pUGGU (see Figure S29 in the Supplementary Materials for additional details).

#### 3.3.2. Templated Ligation Attempts Performed in Two Steps

**Figure 8:**
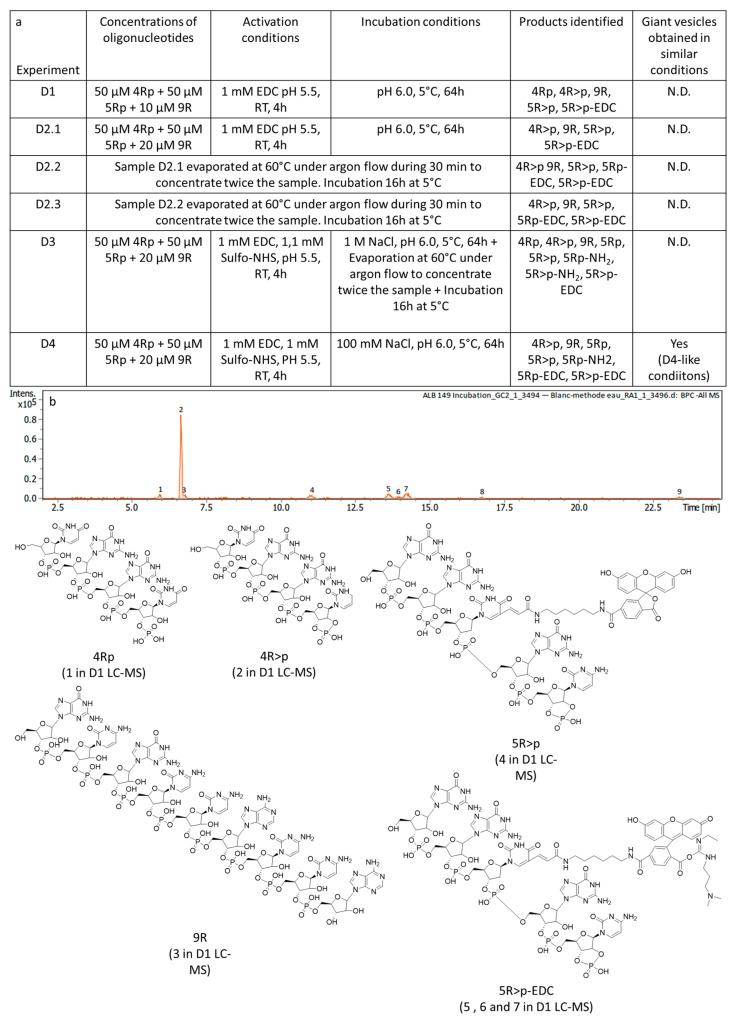
(**a**) Summary of the experiments performed in two steps in order to investigate the templated ligation of oligonucleotides. For each experiment, the concentration of the oligonucleotides, the activation and the incubation conditions are reported. The species obtained for each experiment and detected using LC-MS are given; see Figure 8b for the structures of the main compounds and Figures S31–S37 for the others. Of note, in EDC adducts (like 5Rp-EDC), the linkage can also be made through one of the urea N-atoms (isomeric N-ureyl imide structure not shown; see Figure S31). The formation of giant vesicles (GVs) was only investigated in D4-like conditions (N.D. stands for not determined in the other cases) and GVs were effectively obtained (see Figure 9 for images). (**b**) LC-MS chromatogram (RP-HPLC elution with 5 mm NH_4_OAc pH 8.0 and CH_3_CN/A 95:5) in the negative ion mode of the D1 experiment as an example of the chromatograms obtained for the experiments on the templated ligation of oligonucleotides. The main compounds associated with the LC-MS peaks are shown annotated and reappear below the structures and in Figures S31–S37.

To test the templated ligation of oligonucleotides passing through 2′,3′-cyclic phosphates, we first sought efficient prebiotic conditions to produce these cyclic phosphate–oligonucleotides. Naturally, we tested conditions that were very similar to the ones tested for the coupling attempts to create chimeras, as they were considered prebiotic, compatible with the presence of lipidic giant vesicles and proved to be able to activate terminal phosphate groups. This is why we tried first the cyclisation of 4Rp (100 µm) to make 4R>p in the presence of EDC (1 mm), following a pioneering work by Naylor and Gilham dedicated to studying the reactivities of oligonucleotides in a solution (4 h at pH 5.5 and room temperature) [41]. The more acidic pH would favour the cyclisation of the terminal phosphate. Interestingly, this study did not mention any buffer, which was also in our interest. The resulting solution was analysed using LC-MS and the same HPLC method was used for all further experiments (4)2) in the Supplementary Materials). After 4 h, the reaction was almost over, since almost all of the starting compound 4Rp was consumed (less than 5% left) and reacted to yield a majority of the cyclic phosphate–oligonucleotide of interest 4R>p (around 74%) (Figure S30). Even though an unexpectedly large proportion of the 4-mer was dephosphorylated during this experiment (around 21% 4R), we pondered that this reaction occurred due to a lack of precautions. Considering that the desired 2′,3′-cyclic phosphate was produced in a high amount in these conditions, we decided to keep these parameters to try the templated ligation with 4Rp, 5Rp and 9R in the D1 experiment. Similar conditions were used for the activation with 100 µm of 3′-phosphorylated oligonucleotides, 4Rp and 5Rp (50 µm each), in order to respect the 1/10 ratio with EDC, and with 10 µm of the template 9R. After the activation with EDC, the pH was set to 6.0 by adding 100 mm NaOH and the mixture was incubated for 64 h at 5 °C (Figure 8a). The low temperature would favour the hybridisation of the oligonucleotides with their template, thus, the templated versus the untemplated ligation reaction. After that, the solution was analysed using LC-MS (Figure 8b and Figure S31). However, no fluorescent ligation product, 9Rf>p (or 2′,3′-ring-opened 9Rfp), could be detected, neither using fluorescence-detected HPLC nor by analysing LC-MS chromatograms peak by peak. All new species observed were activated 3′-phosphorylated oligomers that became 2′,3′-cyclic and some of those chemically bonded with EDC. Unfortunately, it seemed that EDC could also react with the fluorescein part of the 5-mers (Figure 8b), resulting in a decreased fluorescence and the consumption of the condensing agent.

To detect a product of ligation despite this first failure, other experiments were performed with higher concentrations of the template and other conditions were explored (experiment D2.1, Figure 8a). Given the absence of ligation detected using LC-MS with this doubled concentration for the template (Figure 8a and Figure S32), we concentrated the sample in order to favour the ligation reaction (simulating evaporative conditions that could take place in hot springs) before additional incubations at 5 °C (experiments D2.2 and D2.3, Figure 8a). However, increasing the concentrations did not yield any ligation product that could be detected using LC-MS (Figure 8a, Figures S33 and S34). The proportions of the activated species obtained from 3′-phosphorylated 4-mers and 5-mers could be followed over these concentrations (Figure S35). No change was observed for 4-mers that were only terminally cyclised as 4R>p before any concentration. For the 5-mers, the concentration only had a small impact as well and three types of activated species coexisted: 5R>p, 5R>p-EDC and 5Rp-EDC.

In the following experiments, sodium chloride was added, since high salt concentrations would enhance the hybridisation between complementary oligonucleotides. In addition, N-hydroxysulfosuccinimide (sulfo-NHS) that could act as a better leaving group after the action of EDC, was also integrated in the activation conditions to catalyse the reaction [4]. Once again, the sample was concentrated in order to increase the concentrations and the result was analysed using LC-MS after incubation at 5 °C (experiment D3, Figure 8a). Nevertheless, these additional parameters did not trigger any templated ligation (Figure S36). Of note, for some compounds, an amino group replaced an OH group in the activated oligonucleotides, furnishing primary phosphoramidates from (probably terminal) phosphate esters. Again, this was due to ammonia generated in the pH-buffered HPLC eluant (NH_4_OAc). As mentioned previously, an attempt to run LC-MS without a buffer was tested to avoid this reaction but nothing could be detected. It seemed that the buffer was necessary for any separation using RP-HPLC and such derivatives were unavoidable. Finally, one last experiment performed in two steps was undertaken (experiment D4, Figure 8a). Indeed, we assumed that the previous conditions were probably too rough for the formation of prebiotic vesicles, so we wanted to try similar parameters but diminishing the concentration of sodium chloride. Unfortunately, these conditions were not suitable for the ligation reaction even though the activated compound 4R>p was present in high amounts, as was the case previously (Figure S37).

**Figure 9:**
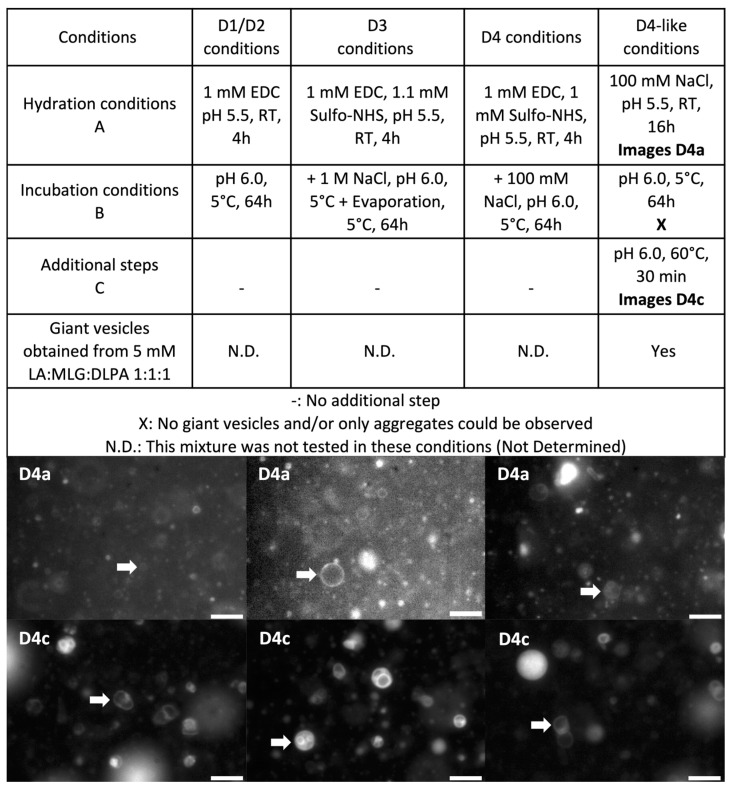
The formation of giant vesicles (GVs) was assessed in conditions similar to the D4 experiment. Among the previous experiments, only the D4-like conditions were investigated for the formation of GVs as they included high concentrations of NaCl considered to be the most challenging parameter (measured osmolality up to 0.9 Osm/kg). GVs were effectively produced in these conditions; see micrograph images of the vesicles. GVs could always be observed except after an incubation for 64 h at 5 °C. However, they could be restored after 30 min at 6 °C in that case. These observations were carried out with a red channel (543 nm) on a Carl Zeiss inverted microscope, Observer Z1, equipped with 20×, 50× and 100× oil immersion objectives and AxioCam recording. The scale bar represents 20 µm on all the images.

As before, we were curious to see if prebiotic vesicles could be produced in such a saline environment (100 mm NaCl). This is why the formation of GVs from a mixture of lipids considered to be prebiotic (1:1:1 LA:MLG:DLPA; see 3)2)1) in the Supplementary Materials) was investigated in similar conditions and giant vesicles were indeed observed using epifluorescence microscopy (Figure 9). This information was very interesting given that high concentrations of salts were probably necessary for the more efficient hybridisation of short nucleic acids but are often regarded as one of the parameters limiting the formation of prebiotic vesicles. The concentrations of mono-valent ions were much higher or similar in D4-like conditions when compared to the D1/D2 experiments or the D4 experiment conditions, respectively. This is why it was assumed that GVs could probably also be produced in these conditions. Even though giant vesicles could not be observed after incubation at 5 °C, a brief heating at 60 °C for 30 min allowed us to restore them. It is very likely that cooling the vesicles led to the formation of aggregates and that heat reconverted them to GVs. Actually, such cycles, that might be present in hot springs, could be very interesting for prebiotic compartments, since the compartments could exchange some material at cold temperatures, when aggregates are formed, and be isolated again at high temperatures, allowing a reshuffling of their contents. A similar mechanism was already suggested in the literature (but for fatty acid bilayers and, in that case, it was the hot temperature that would be responsible for the reshuffling) [56].

#### 3.3.3. Templated Ligation Attempts Performed in One Step

**Figure 10:**
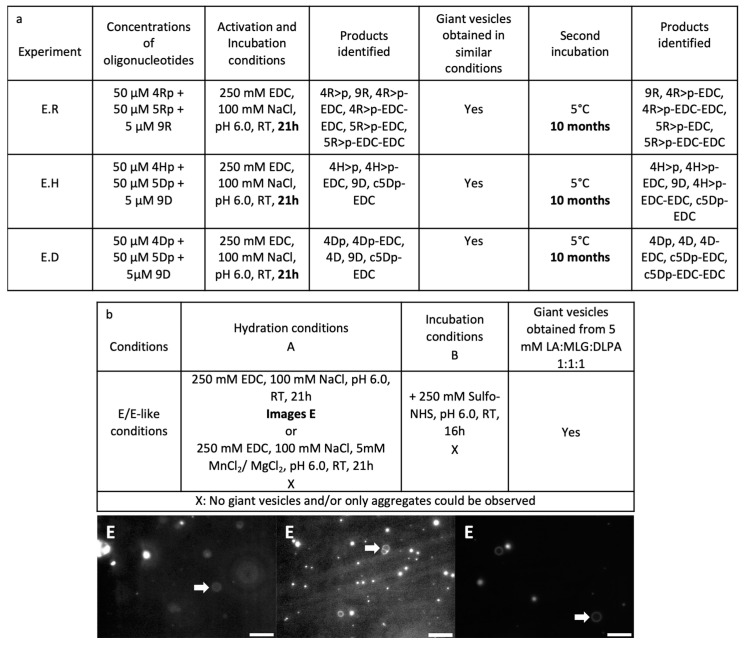
(**a**) Summary of the experiments performed in one step in order to investigate the templated ligation of oligonucleotides. For each experiment, the concentration of the oligonucleotides and the activation/incubation conditions with the species obtained for each experiment are reported. Cyclo-5D-EDC derivatives (c5Dp-EDC) are cyclic 5-mers (cyclopentanucleotides) containing EDC; see Figures S38–S40 for the structures of the most representative compounds. (**b**) The formation of giant vesicles (GVs) was investigated in similar conditions and GVs were effectively observed. These conditions were conducive to the formation of GVs. However, any addition (E-like conditions with MgCl_2_, MnCl_2_ or sulfo-NHS) that might have helped the ligation, or an osmolality above 1 Osm/kg, was fatal for the stability of GVs. These observations were carried out with a red channel (543 nm) on a Carl Zeiss inverted microscope, Observer Z1, equipped with 20×, 50× and 100× oil immersion objectives and AxioCam recording. The scale bar represents 20 µm for all the images.

Given the absence of any ligation product even with a high concentration of salts, or at higher concentrations reached through evaporation, we decided to take a new approach in order to provoke a templated ligation with our oligonucleotides. For that, we tried a reaction performed in one step with high concentrations of condensing agents and salts coupled with lower concentrations of the template [57]. In addition, we expected a difference of stability between double-stranded DNA and RNA and this is why the ligation conditions for two RNA-templated oligoribonucleotides (R) were also tested on DNA-templated hybrid oligo(deoxyribo-ribo)nucleotides (H) able to be transformed to a 2′,3′-cyclic phosphate (experiments E.R and E.H, respectively, Figure 10a). Pure DNA nucleic acids, unable to form 2′,3′-cyclic phosphates but that could be ligated through the chemical activation of their 3′-terminal phosphates, were also tested in these conditions (experiment E.D, Figure 10a). After a first reaction period of 21 h at ambient temperature, the mixtures were analysed using LC-MS, (i) in searching for the most likely formed negative ions (double-, triple- and four-fold-charged) of the most expected ligation products through targeted ion-extraction filtering and (ii) by examining the chromatograms peak by peak (4)3) in the Supplementary Materials). Unfortunately, as was the case previously, no product of ligation could be observed, and that is, 9Rf>p, 9Rfp, 9Hfp or 9Dfp and no EDC derivatives thereof. The obtained compounds were very similar to the ones produced from a two-step ligation procedure. Interestingly, it seemed that the huge amount of the condensing agent used could yield activated compounds with two EDC residues, and some of the DNA 5-mer appeared as cyclopentanucleotide c5Dp (see Figures S38–S40). Hence, despite this higher level of activation, no ligation would occur within the tested incubation period of time. Given these very concentrated conditions, one might doubt the possibility of vesicles co-existing in these conditions. Nevertheless, giant vesicles were effectively produced in these conditions from a prebiotic mixture of lipids (Figure 10b). It seemed that these parameters were still compatible with a templated ligation of oligonucleotides encapsulated in GVs. Any additional compound that might have aided the ligation, such as divalent cations (MgCl_2_ or MnCl_2_) or sulfo-NHS, was shown to be incompatible with the formation or the stability of giant vesicles. Thinking that the reaction of ligation could happen in these conditions but that it might be very slow, we decided to keep the same samples and to prolong the incubation time for 10 months at 4–6 °C. The low temperature was used once again, in order to favour the hybridisation and so the templated ligation. Even after such a long reaction time, no ligation could be detected whatever the type of oligonucleotide. Surprisingly, the activated compounds (cyclic and EDC derivatives) were still present and not hydrolysed, as one might expect. The identified products were similar to the ones produced after 21 h of reaction time. Thus, it seemed that the ligation reaction could not happen in those conditions and that some other players would be necessary.

## 4. Discussion

### 4.1. Summary of the Experiments

This investigation reunited the three molecular partners that were widely acknowledged as being necessary for the origins and self-evolution of cellular life: oligonucleotides, peptides and lipids. In particular, prebiotic chemical conditions that would be at once compatible with the formation of lipidic vesicles and conducive to certain chemical reactions critical for the emergence of life, namely the peptide–oligonucleotide coupling and the templated ligation of oligonucleotides, were sought for. For that, short and random peptides were generated, through activation with CDI, from mainly prebiotic amino acids. Thus, the obtained populations of peptides could be representative of a prebiotic ‘peptide soup’. The analyses of this soup revealed that at least some of the produced peptides could be long enough and perhaps hydrophobic enough (especially those longer than 10-mers) to anchor to lipidic vesicles. The β-azidoalanine-containing peptides could be used as trackers to show that (and which) peptides could effectively get into vesicles where they would react with encapsulated fluorescent clickable probes. Some short oligonucleotides, which could have been present on the early Earth, were produced using solid-support synthesis, manually deprotected and purified using HPLC. One of them (pUGGU alias p4R) was used to test the coupling between oligonucleotides and peptides in the case of 5′-phosphorylated oligonucleotides that should lead to peptido-RNA phosphoramidates. Indeed, this chemical bond is particularly interesting since it would be transient and metastable depending on the pH of the medium and might explain how lipophobic oligonucleotides could get into lipidic vesicles: thanks to a temporary lipophilic peptide chain. Lipophilic peptides in such chimeras are considered particularly useful in a systemic sense because, being produced from the dehydration of amino acids, they are more readily evolvable in length and composition. Fine-tuned properties, through chemical reversibility of the peptide bond formation, in milder prebiotic environments are more rapidly optimised without collateral destruction than long-carbon-chain lipids, as in lipid-RNA chimeras, composed of many sequential very stable C-C bonds that are difficult to form in prebiotic conditions that still tolerate the integrity of nucleic acids [5].

The coupling between pUGGU and the soup of peptides was tested under several conditions that involved the presence of a condensing agent (EDC) and a catalyst that should enhance the reaction. However, only activated oligonucleotides (EDC derivatives mainly) could be detected using LC-MS. We suspect that the EDC adducts that we detected using LC-MS were in fact those more stable isomers that rearranged from O-isoureyl phosphates (depicted in Figure 5c and Figure 9c) to the much less reactive N-ureyl phosphoramidate isomers. Even though the complete absence of coupling in these conditions should not be underestimated, several elements might explain why no chimeras were observed: (i) the concentration of each peptide present in the soup was too low and so was any peptide–oligonucleotide that could be formed and would remain undetectable, and (ii) any chimera could be aminolysed by the ammonia present in the buffer, which would explain why quite high amounts of 5′-terminal primary phosphoramidates (H_2_N-P-5′) were observed. Unfortunately, this buffer was necessary for the LC-MS analysis. Trying these conditions with one pure and prebiotic peptide prior to the analysis using a method that does not involve the presence of ammonia (such as MALDI-MS) would be ideal for future investigations. Obviously, it may be that other players would be necessary to explain how couplings could occur between these partners, such as other condensing agents and/or catalysts. All the synthesised 3′-phosphorylated oligonucleotides were used to probe the templated ligation of oligonucleotides passing through 2′,3′-cyclic phosphates. This intermediate is easily formed in prebiotic conditions and might explain the elongation of oligonucleotides so crucial for the origins of life. A templated ligation reaction was tested in several conditions involving a condensing agent (EDC) and NaCl and/or a catalyst sulfo-NHS that should boost the reaction. Nevertheless, only chemically activated oligonucleotides (cyclic and EDC derivatives mainly) could be detected using LC-MS and even so when the reaction was prolonged at 4–6 °C for an extensive 10-month period of time. Several reasons could explain why these oligonucleotides could not ligate: (i) The oligonucleotides were too small (or not palindromic). The melting temperature for duplex formation was too low for an efficient hybridisation and templated ligation (about 12 °C for the 4-mers, calculated with OligoCalc™). (ii) Other molecular players would be necessary to explain how this ligation could occur in prebiotic conditions. To fathom these, conditions would still have to be compatible with the presence of lipidic vesicles, as it was the case in most of the previous experiments. Indeed, only by finding truly (modelled) prebiotic conditions (without synthetic buffer, agitation or non-realistic catalysts), still allowing for efficient chemical reactions in the presence of lipidic vesicles, all partners (molecular compounds) could be gathered in such a way that their interactions, vital for the outbreak of life, would be revealed.

### 4.2. RNA World versus RNA-Peptide World

We are not the only experimentalists who were thus far futile in efficiently attaching amino acids and peptides to the terminal phosphate or hydroxy group of a natural oligonucleotide in undirected (through chemical manipulation) plausibly prebiotic conditions. We did orient ourselves in following those experimentalists who were successful in attaching amino acids to nucleoside 5′-monophosphates and elongating these in both directions, albeit growing peptides much faster than nucleotides [34]. Decades-long experimentation on the elongation of RNA, or other nucleic acids, in the absence of protein enzymes, including those catalysed by in vitro-selected ribozymes, be it through templated primer elongation or templated ligation of nucleic acids, just confirms the longer the more that this cannot be a chemically feasible way of making these biopolymers longer, in plausibly prebiotic conditions that model such environments, hence making them more complex, transferring more sequence information to be inherited by succeeding generations and evolving more diverse functions that can serve the whole system to prevail, persist and reproduce. A polymeric material composed of only four different monomeric units that should function at the same time as a genetic (inheritable) information storage system as well as providing catalysis, including that of self-replication, is just too much of squaring the circle. Even when ribozyme sequences have been (and will be) found that indeed catalyse their self-replication [58], they are too error-prone and, what is worse, too deleteriously mutable—only few mutations (replacements of a few nucleotides with others, or deletions) bring their catalytic ability to a halt. The hypothesis of an ‘RNA world’ reproducing through the catalytic action of ribozymes [59], not protein enzymes, Dead Parrot (Supplementary Material) [60].

We need to include a primitive metabolic system that ensures an effective and uninterrupted connection between, on the low entropy side, the making and reproduction of valuable molecules and the increasingly efficient transformation of energy and information into useful work (exergy) and, on the high entropy side, the rate of dissipation of thermal energy given portion-wise back to the surroundings, for more and more autonomy of the system to emerge and develop (evolution) [61,62]. For this to happen and endure, protein enzymes seem unavoidable because they are the only catalysts to be modular, efficient and fast enough to prevent any devastating drain into uncontrolled side reactions of protometabolic reaction networks. Protein production can be regulated through their translation from (transcribed) nucleic acids [63,64]. The key for evolving a chemical system to a biological system is and always was the emergence of an operational genetic code [65,66,67]. The takeover of an RNA world by a RNA-protein world, which is equivalent to the late emergence of a translation apparatus in already living and thriving organisms, is impossible without going back to the (evolutionary) field number one [68,69,70]. The most prominent, throughout all known organisms, totally ubiquitous and best-known natural ribozyme is the ribosome, hence, the cellular machine that produces proteins from amino acids. It is huge (read: old), and consumes GTP rather than the usual ATP (guanosine rather than adenosine-5′-triphosphate), in order to be sufficiently autonomous and precise by means of its many associated protein cofactors. The ribosome produces, apart from these ribosomal cofactors, a defined number of small ribosomal proteins essential for its own existence and functioning. The ribosome spends the rest of its lifetime serving the cellular system with the production of all other necessary proteins that are needed to maintain a metabolism and to structure the cell(s). The production of this totally promiscuous protein synthesis machinery, serving a great many sub-purposes, costs the cell less energy and time when the main scaffold is composed of RNA rather than protein. This has been proposed to be the main reason for why the cellular protein factory has—quite exceptionally—evolved to be a ribozyme [71]. But, if a hypothetic RNA world, that is, thriving populations of all-ribozyme cells, existed on the early Earth, maintained their metabolism, produced all essential cell components and replicated their genome with (catalysed by) ribozymes, how come the fraction of non-coding (nc) DNA, a large part of which is transcribed (>90% of *Homo sapiens* ncDNA, for instance) into regulatory and catalytic ncRNA but not translated from genes to proteins, is growing with the complexity of organisms? *Nanoarchaeum equitans* has less than 5% ncDNA, and this proportion grows higher and higher in progressively complex prokaryotes, primitive eukaryotes, fungi, plants, invertebrates, urochordates, vertebrates and peaks at about 98% ncDNA in *Homo sapiens* [72]. From an initial RNA world, one would expect an ncDNA (→ncRNA) peak much earlier on the evolutionary timeline [64].

### 4.3. RNA-Driven Peptide Synthesis, Peptide-Driven RNA Elongations, the Emergence and Evolution of Translation, and of Nucleic Acid Replication

The failed observation of an expected outcome, which was to obtain terminal phosphate-linked (or more labile carboxy ester-linked) peptide-RNA chimeras and templated oligonucleotide ligation products in almost stubbornly prebiotic, chemically activated conditions that were compatible with the maintenance of reasonably stable vesicular lipid membranes, is of course deceiving. What more than to learn from failed experimental results makes us proceed towards success—so, what to do next? We say, carry on with periodically energising chemical systems composed of at least three compound classes, peptides, nucleotides and lipids [73,74], eventually (or straight away) in the presence of potentially useful metabolite precursors, i.e., small metastable organic molecules like pyruvate and glyoxalate [75], with an open mind but wisely, especially concerning the actual initial molecules and the conditions that model the prebiotic environment. As to the initial molecules, pioneering work on RNA-promoted peptide synthesis from amino acids has been carried out in the labs of Thomas Carell and Clemens Richert [4,39]. Given the importance of generating more diverse, mutationally more robust and catalytically more competent biopolymers (enzymes), composed of a high number of different residues (amino acids, up to 20 ‘letter’ digits, vigintits), translated from polymers that are far less performant catalysts (ribozymes and DNAzymes) but much more accurately copied (by templating) because the number of different residues is much lower (up to four different nucleotides, quaternary digits, quits) [76], the approach taken by Carell and co-workers may have an important bearing on the experimental evolution of a translation machinery. Their idea was that, rather than focusing on the CCA 3′-terminus or the 5′-terminus of tRNA as the attachment point where amino acids are targeted and then grow in length (as in modern ribosomal protein synthesis), the nucleobases (not ribose nor phosphate) that are near their anticodon (the other three-dimensional end of tRNAs), or actually part of it, could be targeted for the connection with amino acids that grow peptides in the *gestalt* of peptide-RNA chimeras. Although several alternative tRNA evolutions have been proposed in the literature (reviewed in [77]), both aforementioned options consider that modern tRNAs are double the size of primordial tRNAs [55]. The chemistry that takes advantage of the connection between the exocyclic amino group of a N-heterocyclic base (adenine) and the amino group of an α-amino acid or peptide through an acid-cleavable urea linkage [4] is much more robust than what is expected to become an unsurmountable issue when trying to attach, in the absence of protein enzymes, amino acids or peptides to the 2′,3′- or 5′-terminus of nucleic acids without too much artificial intervention of the experimenter. Carell’s pioneering “anticodon base approach” has a very convincing asset: double-stranded (ds) RNA can grow a peptide that is transiently attached to both RNA strands, forming a chimeric hairpin structure with a peptide loop. One peptide-RNA linkage remains stable, namely, the carboxamide linkage provided by the peptide accepting the RNA strand, and the other is robust but cleavable, viz., the urea linkage of the peptide donating the RNA strand (similar to the boxed structures in Figure 12).

This chemistry may be further developed, but what can be boiled down from former numerable works is that enzyme-free nucleic acids cannot grow efficiently in length owing to the too-weak nucleophilicity of their OH termini, no matter how high of a chemical activation (electrophilicity) of the to-be-linked phosphate monoester has been achieved by adding electron-withdrawing fast prebiotic leaving groups and providing spatial vicinity through base-paired templating. One of few successful spontaneous and efficient templated (natural) nucleic acid elongations carried out in a liquid aqueous solution in the absence of solid supports, minerals or clays was the autocatalytic ligation of 10–12 mm DNA fragments (each chemically blocked on one terminus to suppress untemplated ligation) using 200 mm EDC, which led to sub-exponential (parabolic) DNA-templated ligation kinetics achieved during 4 days in a 10 µL volume at pH 6.15 in the presence of 50 mm MgCl_2_ [42]. Another quite successful enzyme-free but not autocatalytic DNA ligation started from a pre-organised ligation partner forming a hairpin loop structure, which can be regarded as the religation of a nicked DNA hairpin loop, accompanied by deleterious effects concerning the accumulation of dead-end byproducts with time passing [57]. Our work confirms that the electrophilicity of chemically activated terminal phosphate esters is too weak for their ligation with (perhaps not very well) templated 5′-terminal OH groups, and shows in addition that such activated phosphate termini of oligonucleotides probably do not even react with much more nucleophilic NH_2_ groups present in amino acids and peptides. The aforementioned results obtained by the Carell group show that this fundamental chemical problem may be, if not solved then, pushed considerably further down the complexification pathway by using a combination of two enhancing effects. Both the hairpin-looped template effect *and* the presence of a nucleophilic terminal NH_2_ group provided by the amino acid/peptide acceptor RNA strand, while hybridised to the amino acid/peptide donor RNA strand in these chimeras, can apparently overcome the issue that is inherent to nucleic acids alone.

An as-if collateral benefit for such a chimeric system is the growth of a peptide chain being physically connected to dsRNA, which itself can evolve in close proximity a “primary” codon–anticodon pairing (matched versus mismatched) code, while a “secondary” operational nucleic acid–amino acid recognition (selectivity) code is created. This opens the gate to evolve future aminoacyl transfer RNA synthetase (protein) active sites or, alternatively, a nucleic acid polymerase (protein) active site. In the framework of irreversible thermodynamics of a chemical system driven by the export of entropy and ensuing boundary conditions that follow an extended general evolution criterion [78], this unlocks the bifurcation of an evolutionary pathway, one into the evolution of a genetic code through error-poor genome translation into proteins, and the other into the fidele and *periodic* replication of any nucleic acid genome, i.e., oscillating in concert with the doubling of cell boundaries (reproduction) [61,70].

## 5. Conclusions

Our work shows that it is not obvious how to attach amino acids or peptides to the chemically activated terminus of an oligonucleotide under conditions that tolerate lipid vesicles, neither to a terminal phosphate group of an oligonucleotide nor even less so to its terminal hydroxyl groups. In addition, the templated ligation of oligonucleotides in similar conditions is far from evident either. As for the latter, we are convinced that the overall message of all these failed or unsatisfactory chemistries (ours and others), obtained from testing in convincingly prebiotic model conditions natural nucleic acids alone, have shown that the self-evolution of nucleic acids and peptides must have been driven at first by their co-evolution and mutual collaboration right from the start of their existence on the early Earth (the “molecular deal” [79]). This is not only because of the beneficial physico-chemical properties due to their amphiphilicity when joined to chimeras, but also because, while peptides grew in length on nucleic acids or without them (up to a certain length that made them precipitate), nucleic acids could probably only grow properly in length and quantity when catalysed, chaperoned by peptides or proteins that have eventually evolved to become enzymes, such as nucleic acid polymerases and helicases, also aminoacyl transfer RNA synthetases, and so forth. Carell’s system is different from ours because it starts with attaching an amino acid or peptide to a terminal nucleobase of an oligonucleotide, which opens the door for exactly this asset to be evolved, namely, dsRNA produces hairpin loops by letting peptides (not RNA!) grow on them intramolecularly and thanks to the high nucleophilicity of the growing peptide’s N-terminus being much more effective than the OH-terminus of any nucleic acid—this is, in prebiotic conditions that tolerate the presence of lipid vesicles. The vesicles, protocells, are mandatory for selection mechanisms to become operative.

## 6. Perspective

**Figure 11:**
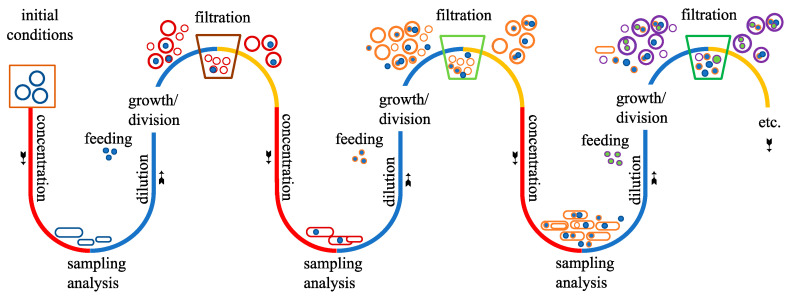
Schematic representation of concentration–dilution (c–d) cycles carried out in a programmable incubator connected with syringe pumps (following the arrows from left to right). Filtration of the diluted vesicle suspension (upper states on the waved line) through a membrane filter selects for the mechanically most fit vesicles being subjected to further rounds of c-d cycles. Vesicles at very high concentration (lower states) appear distorted (flattened) and are prone to fuse and invaginate smaller vesicles. Diluting and feeding large and giant vesicles with amphiphile micelles and small vesicles make them grow in size and divide to grow in numbers. The feeding vesicles could contain peptide and oligonucleotide cargo, also in the form of hydrolytically metastable peptide–oligonucleotide chimeras. Hetero-disperse populations of increasingly mixed membrane compositions (represented as colour changes in vesicle boundaries) become fitter against the filtering when repeatedly filtered, concentrated and rediluted. Colours of the c-d cycle line indicate elevated (red), medium (yellow) and cool (blue) temperatures.

We have proposed to the *Agence Nationale de la Recherche* (ANR) and to the *European Innovation Council* (EIC) research projects called ChemLife and, respectively, SynthCells 2.0 where, among other sub-projects, the periodic addition of chemical activators, amino acids and nucleotides (monomeric and oligomeric) was supposed to impact pre-formed giant mixed-lipid vesicles that would be submitted to temperature cycling, thus, to periodically lower and higher concentrations, also osmotic (salt concentration) and/or pH cycling, and would be intermittently fed with more lipid amphiphiles (micelles or vesicles), in order to let the initial “mother” vesicles grow in size and divide, thus, grow in population size by producing “daughter” vesicles that could evolve to become from generation to generation gradually fitter against periodic shearing stress imposed by repeated gentle (controlled) filtration events (Figure 11).

**Figure 12:**
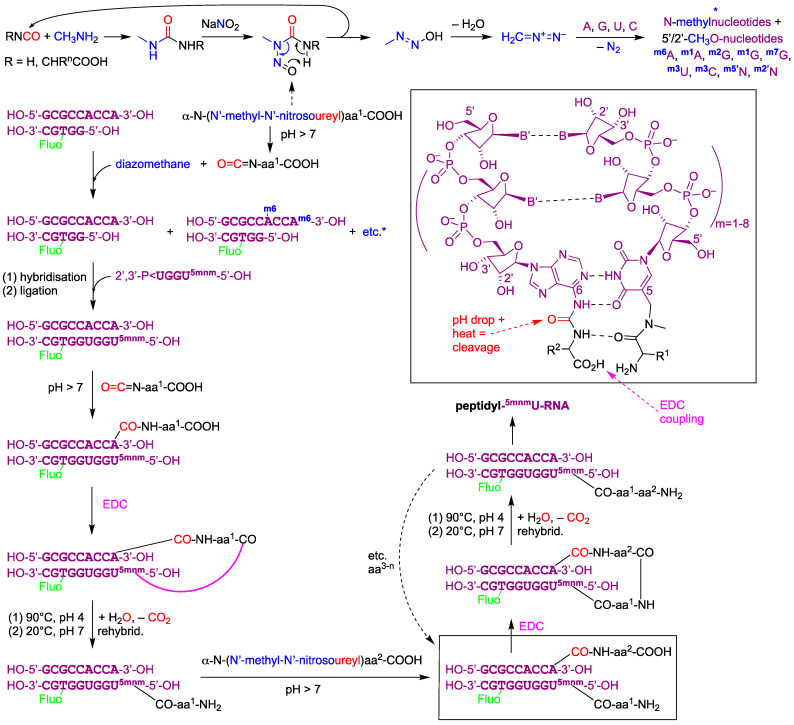
Strongly simplified dsRNA-directed peptide synthesis from RNA and α-*N-*(*N’-*methyl-*N’*-nitrosocarbamoyl)amino acids that (1st row) could be formed from α-*N-*(*N’-*methyl-*N’-*carbamoyl)amino acids (linkage abbreviated with red ‘ureyl’) and nitrite, rapidly degrading at elevated pH values to α-*N-*(amino acid)isocyanates and diazomethane. The final products are 5-(methylamino)methyluridylate-linked peptidyl-RNAs. The intermediate just before peptide bond formation is shown in a square. The asterisks * indicate products from unselective methylation by in situ-generated diazomethane, which might both disturb base pairing (at *N*-methylated sites) or enhance the efficiency of RNA-driven peptide synthesis (2′-*O-*methyl RNA double-strands hybridise more tightly), which gives an even wider playground for selection to impact the c-d cycles (Figure 11).

Both projects have been rejected. We passed the first ANR pre-submission hurdle for ChemLife but did not answer well enough what we would do if we failed (go home?). SynthCells 2.0 would not be sufficiently competitive with other EIC Pathfinder submissions. Figure 12 shows the sequence of sought-after chemical events that is based on Carell’s system [4]. Of note, we inversed with respect to the inventor the direction, i.e., the non-canonical (base-modified) donor and the acceptor termini. That way we would quest—after all—the biological CCA-2′/3′ terminus by testing its amino acid/peptide donor ability in such chimeras. We proposed to include a templated ligation event because we were still convinced at the time that this was feasible, but a similar sequence of events could produce growing peptides on peptide–nucleic acid chimeras without ligation. The whole approach is based on producing mixtures of not exactly predicable composition and letting giant vesicles select the molecules that make them fitter. We expect to evolve through successive vesicle generations peptide sequences that would start catalysing specific RNA aminoacylations, stepwise RNA elongations, RNA-RNA ligations and related functions. We cannot carry out the experiments anymore: “[No] money for dope, [no] money for rope” [80]. Maybe someone else will take this torch.

## Data Availability

All data are included in the article and the Supporting Information file.

## Supplementary Materials

The following supporting information can be downloaded at: https://www.mdpi.com/article/10.3390/life14010108/s1.

## Glossary of Amino Acid Abbreviations

A = l-alanine, AA = β-azido-l-alanine, G = glycine, L = l-leucine, PS = *O-*phospho-l-serine, V = l-valine.
