# Supplementary material for: From the RNA-Peptide World: Prebiotic Reaction Conditions Compatible with Lipid Membranes for the Formation of Lipophilic Random Peptides in the Presence of Short Oligonucleotides, and More
Source: Life (Basel). 2024 Jan 9;14(1):108. doi: 10.3390/life14010108 (PMC10817532; doi:10.3390/life14010108)
Supplement: Supplementary file 1 [file life-14-00108-s001.zip › life-2765134-Supplementary Materials.pdf]

Supporting information — From the RNA-Peptide World: Prebiotic reaction conditions compatible with lipid membranes for the formation of lipophilic random peptides in the presence of short oligonucleotides, and more.

Augustin Lopez, Antoine Vauchez, Ghinwa Ajram, Anastasiia Shvetsova, Gabrielle Leveau, Michele Fiore and Peter Strazewski*

Université de Lyon, Université Claude Bernard Lyon 1, Institut de Chimie et Biochimie Moléculaires et Supramoléculaires (ICBMS), Laboratoire de Chimie Organique 2 (LCO2), Bâtiment Edgar Lederer, 1 rue Victor Grignard, 69100 Villeurbanne, France

* Correspondence: strazewski@univ-lyon1.fr

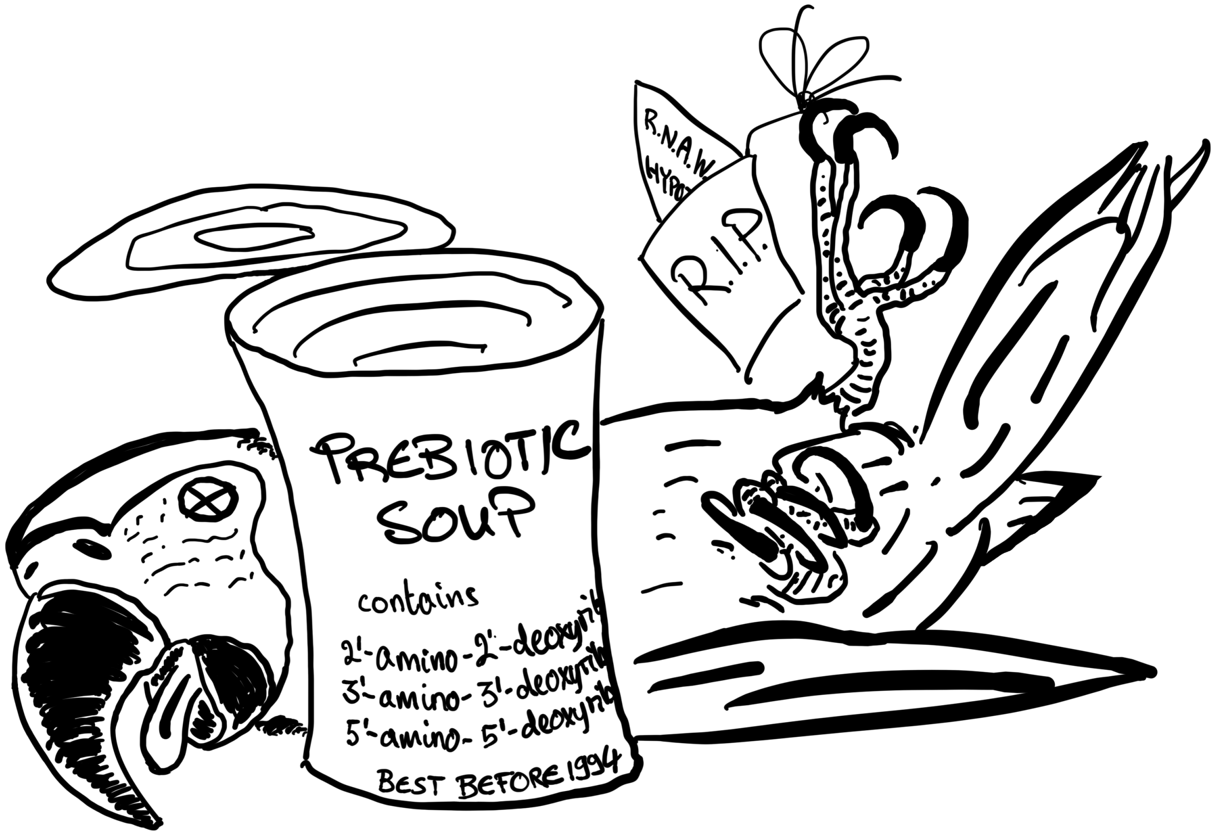

The graphical abstract on the first webpage of the article is an illustration of the current situation in the origins of life sciences where the community is split into those that, like in Monty Python's "Dead Parrot Sketch" [see ref. 60], believe that the RNA world hypothesis is not valid any more (pet buyer: "this parrot is dead, stone-dead, it has deceased, it is no more") and those that still clinger to it (pet seller: "no, no, it's resting, it just sleeps"). Ever since experimenters have shown that, in order to make nucleic acids grow enzyme-free and efficiently in length, they needed to be artificially modified by noticeably more nucleophilic amino groups that replaced the natural hydroxy groups, which was an approach that started 1987, peaked 1994 and went on for one and a half decades. It has thus become more and more evident that natural nucleic acids would just not be able to grow considerably without the help of other molecular players, most likely composed of catalytic peptides, proteins.

Table of contents

1) Chemicals, general instruments and analytical procedures 3

1)1) Chemicals 3

1)2) General instruments 3

1)3) HPLC instruments and materials 3

1)4) Instrument and material for the solid-supported synthesis of oligonucleotides 3

1)5) MS analyses 4

1)6) NMR analyses 4

2) Supplementary information to: Production and characterization of mixtures of random peptides.. 4

2)1) NMR characterizations of the random peptides generated in GAVLPSAA mixtures 4

2)2) MS characterizations of the random peptides generated in GAVLPSAA mixtures 10

2)3) HPLC characterizations of the random peptides generated in GAVLPSAA mixtures (click reactions) 16

3) Supplementary information to: Coupling attempts of peptides with oligonucleotides in prebiotic conditions compatible with the presence of lipidic giant vesicles 18

3)1) pUGGU RNA production and analysis 19

3)2) Coupling attempts and vesicles production in the presence of EDC and EtIm 20

3)3) Coupling attempts and vesicles production in the presence of EDC and DMAP 35

3)4) Coupling attempts with NCA in the presence of EDC and EtIm 54

4) Supplementary information to: Ligation attempts between activated complementary oligonucleotides in prebiotic conditions compatible with the presence of lipidic giant vesicles 59

4)1) Oligonucleotides production and analyses 60

4)2) Templated ligation attempts performed in two steps and vesicle production in the presence of EDC and sulfo-NHS 75

4)3) Templated ligation attempts performed in one step and vesicle production in the presence of EDC and NaCl 100

5) References 127

1) Chemicals, general instruments and analytical procedures

1)1) Chemicals

4-(Dimethylamino)pyridine (DMAP), *Nile Red*™ dye, trimethylamine tris-hydrofluoride (97 %, Et_3N-3HF) were purchased from Acros Organics. 1,2-Dilauroyl-*sn*-glycerol-3-phosphate (DLPA) and 1,2-dioleoyl-*sn*-glycero-3-phosphoethanolamine-N-(lissamine rhodamine B sulfonyl) (DOPE-Rhodamine) were from Avanti Lipids. MeOH, ammonia solution (35 % *v/v*), sodium chloride (NaCl) and methoxytrimethylsilane were from Fisher Scientific. Magnesium chloride, *O*-phospho-L-serine and L-leucine were from Fluka. 3-Azido-L-alanine (β-azidoalanine) and the fluorescent and clickable probes (DBCO-AF488 and DBCO-PEG_4-5/6-FAM) were from Jena Biosciences. Ethanolic methylamine (33 % *v/v*), glycine, L-alanine, L-valine, 1,1'-carbonyl diimidazole (CDI) and lauric acid (LA) were from Sigma-Aldrich. 1-Ethyl-3-(3-dimethylaminopropyl)carbodiimide (EDC), N-hydroxysulfosuccinimide (sulfo-NHS), 1-monolauroylglycerol (MLG) and 1-ethylimidazole (EtIm) were from TCI.

1)2) General instruments

The instruments used were an Eppendorf™ Thermomixer Compact, a Christ Alpha 1-4 LD plus lyophiliser, a Herafreeze™ 586 Basic freezer, a Sigma *Laborzentrifugen* centrifuge from Fisher Scientific, a V-730 UV/visible light spectrophotometer from Jasco, a programmable IPP55plus incubator from Memmert, a SC110A SpeedVac™ concentrator from Savant, an OsmoTouch™ 1 osmometer from Astori, a pH meter and microelectrode from Toledo, a Carl Zeiss inverted microscope Observer Z1 equipped with 20x, 50x and 100x oil immersion objectives and AxioCam recording.

1)3) HPLC instruments and materials

All High-Performance Liquid Chromatography (HPLC) analyses and purifications were performed using reversed-phase elution on a LC20 system from Shimadzu (high-pressure mixing double-pump system; max flow rate 20 mL/min for each pump). The system was equipped with a DGU-20A3R degasser from Shimazu (operative up to 10 mL/min per line), separate analytical and preparative capillary lines, two separate injectors from Rheodyne (100 µL and 2 mL loops). The columns were sometimes placed in a programmable incubator (Memmert) to maintain a thermostatic column temperature. For each analysis 5 µL were commonly injected, purifications could involve up to 1.8 mL per injection, depending on the column, including multiple injections. The eluted compounds were observed using ultraviolet (UV) detection at two wavelengths at a time (SPD-20A from Shimadzu), fluorescence detection (RF-20A XS from Shimadzu) and laser-driven evaporative light scattering detection (ELSD, Sedex 90 LT from Sedere). The three detectors could be used separately, pairwise or all together in the sequential order as mentioned above.

HPLC grade acetonitrile and methanol eluents were from Fisher Scientific. Ultra-pure water was taken from an ACCU20 Ultra-Pure Water System from Fisher Scientific. Glacial acetic acid (HPLC grade) and aqueous ammonia (35 %) were also from Fisher Scientific.

1)4) Instrument and material for the solid-supported synthesis of oligonucleotides

Oligonucleotide synthesis on solid support was performed on an Applied Biosystems DNA/RNA Synthesizer, model ABI 392 (8 monomer bottles) equipped with conductivity monitoring of stepwise yields and operated using a computer with the *OligoNet 1.0.1*™ software. The dry solvents (CH_3CN and DCM) were from Sigma-Aldrich (DNA synthesis grade). The synthesizer-related chemicals, solutions and solid supports (crystalline ETT activator, Cap Mix A: THF/lutidine/acetic anhydride 8/1/1, Cap Mix B: 10% NMI/THF, Deblock Mix: 3% TCA/DCM and Oxidiser: 0.02 M iodine in THF/pyridine/water 7/2/1, chemical 5'-phosphorylation reagent (CPR), CE-phosphoramidites (dmf-G and U) and 10 µmole-scale

columns filled with CPG 1000 solid-support-bound 3’-terminal nucleoside or phosphate were all purchased from Link Technologies.

1)5) MS analyses

Low-resolution Mass Spectrometry (MS) was carried out on a Bruker AmaZon SL ion trap spectrometer with an electrospray ionization (ESI) source. Both positive and negative ion modes could be used.

Ultra-High-Performance Liquid Chromatography/Mass Spectrometry (UHPLC/MS) was carried out a U3000 system (Thermo Scientific), hyphenated to a Bruker Impact II quadrupole/time of flight (Q-TOF) high-resolution mass spectrometer. For ESI-MS both positive and negative ion modes were used.

Matrix assisted Laser-desorption (MALDI)-MS was carried out on a Bruker Autoflex speed MALDI-TOF spectrometer operated in positive ion mode. Each sample (0.5 µL) was mixed with a matrix solution (0.5 µl) consisting of 2’,4’,6’-trihydroxy acetophenone and diammonium citrate, spotted on a stainless-steel plate then dried at room temperature.

1)6) NMR analyses

All deuterated solvents originated from Eurisotop. NMR spectra were recorded on Bruker Advance spectrometers: 300 MHz (for ^{1}H) or 400 MHz (for ^{1}H).

2) Supplementary information to: Production and characterization of mixtures of random peptides

2)1) NMR characterizations of the random peptides generated in GAVLPSAA mixtures

Glycine (Fig. S1), L-Alanine (Fig. S2), L-Valine (Fig. S3) and L-Leucine (Fig. S4) in D_2O were independently added in different concentrations to 2.5 corresponding equivalents of solid CDI and left for different times under agitation. The ^{1}H NMR (300 MHz) spectra were recorded A) before and B) after addition of CDI.

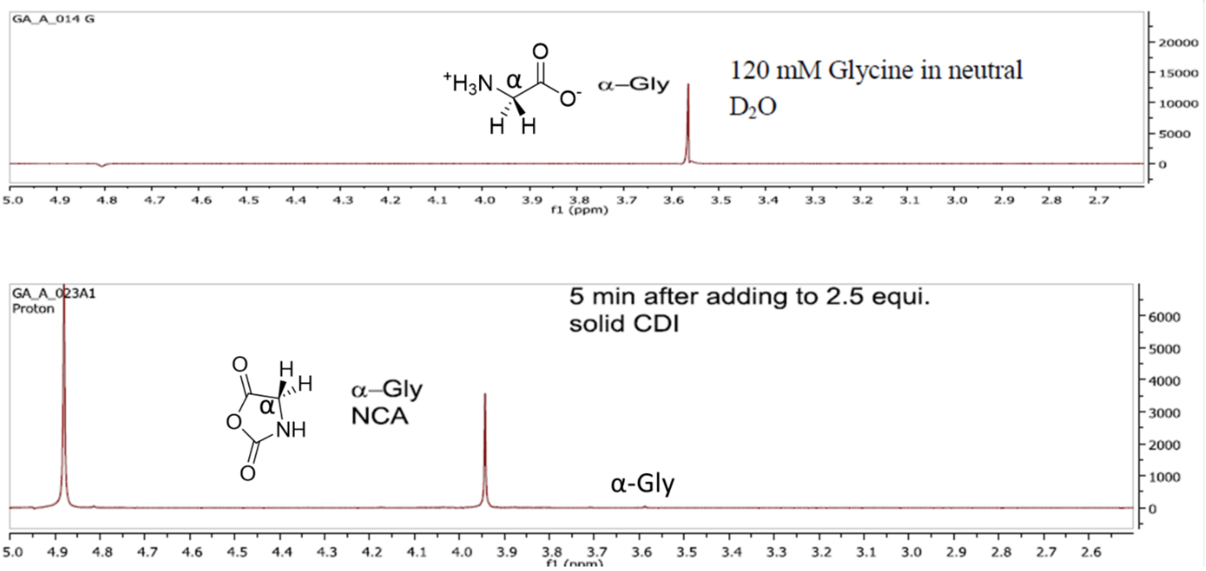

Figure S1. ^{1}H NMR (300 MHz) of 120 mM glycine in D_2O recorded before (upper spectrum) and 5 minutes under agitation after the addition to 2.5 equivalents of solid CDI (lower spectrum). Residual HDO at δ_H = 4.88 ppm.

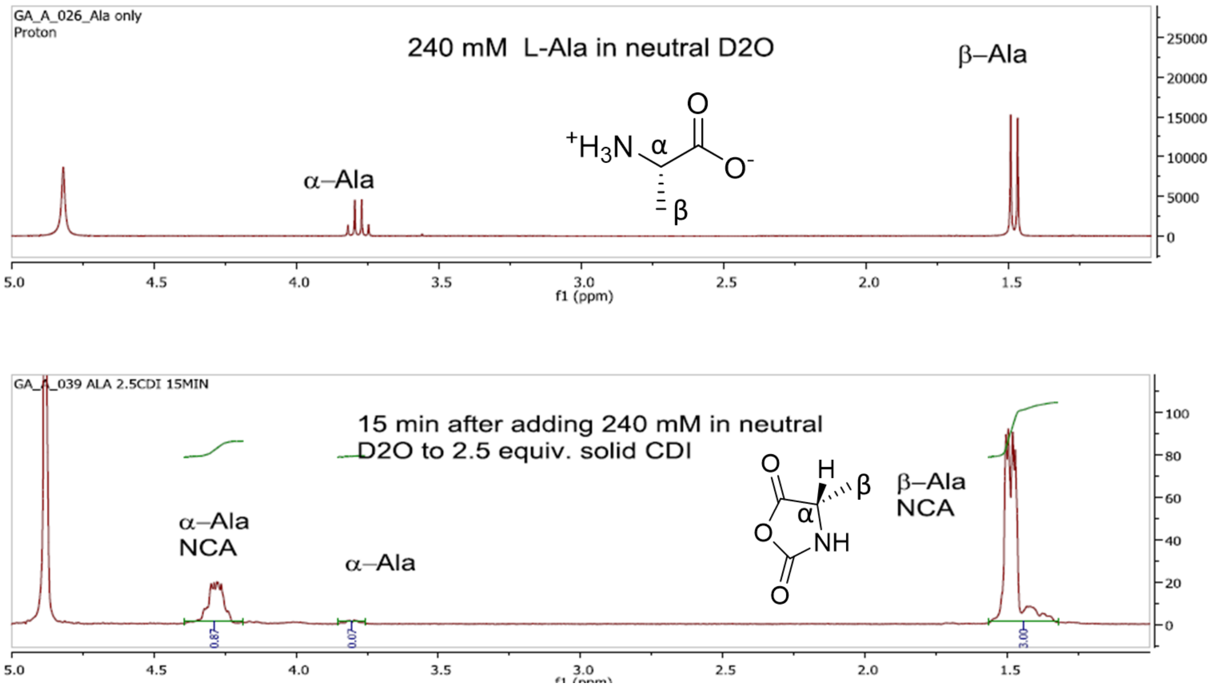

Figure S2. ^{1}H NMR (300 MHz) of 240 mM L-alanine in D_2O recorded before (upper spectrum) and 15 minutes under agitation after the addition to 2.5 equivalents of solid CDI (lower spectrum).

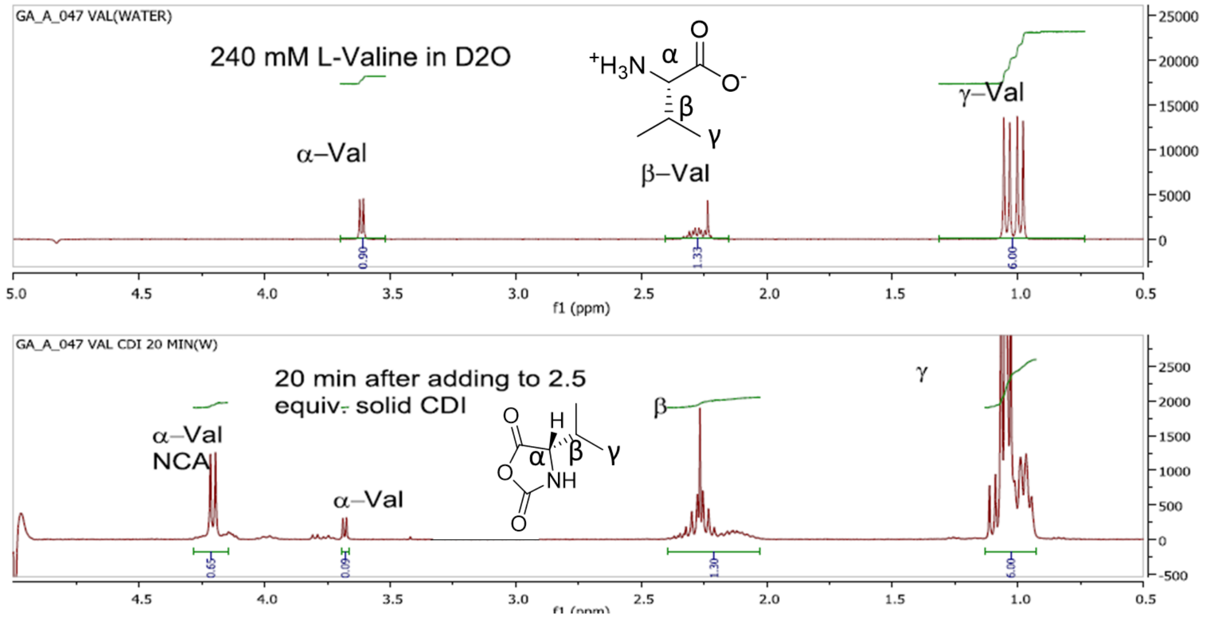

Figure S3. ^{1}H NMR (300 MHz) of 240 mM L-Valine in D_2O recorded before (upper spectrum) and 20 minutes under agitation after the addition to 2.5 equivalents of solid CDI (lower spectrum).

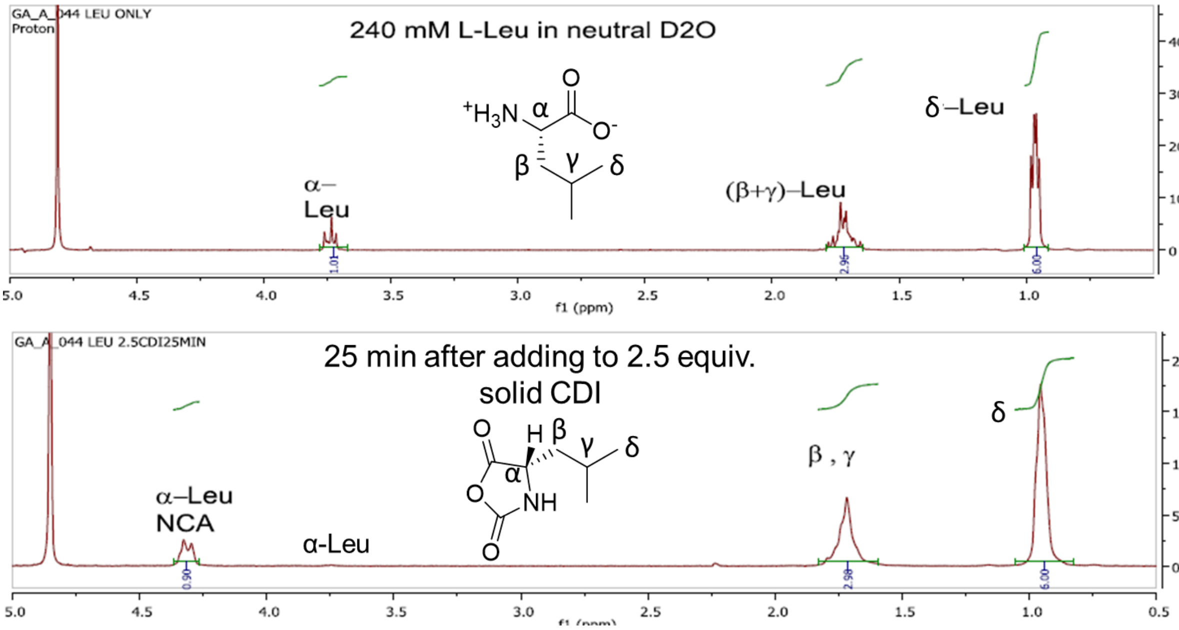

Figure S4. ^{1}H NMR (300 MHz) of 240 mM L-leucine in D_2O recorded before (upper spectrum) and 25 minutes under agitation after the addition to 2.5 equivalents of solid CDI (lower spectrum).

General method of peptide production (GMPP): For the following experiments, a solution containing 200 mM amino acids in various proportions was solubilized in H_2O/D_2O 9:1. After 30 min at room temperature, the sample was analysed using NMR spectroscopy. Then, the sample was cooled down in an ice/water bath for 30 min prior to be added on cold and dry, solid CDI (final concentration of 500 mM). The reaction was left for 15 min under agitation in an ice/water bath prior to be analysed using NMR spectroscopy. Then, the reaction was left for 64 h under agitation at room temperature prior another analysis using NMR spectroscopy (Scheme S1).

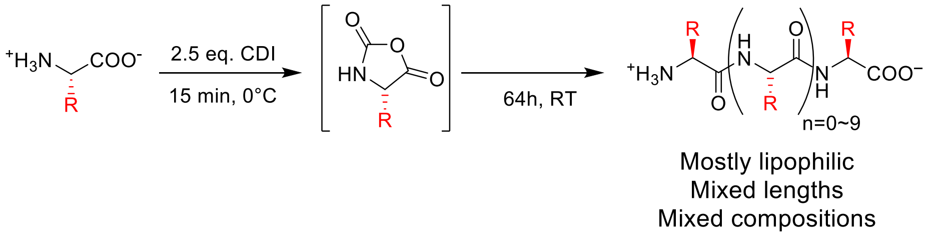

Scheme S1. GMPP: Random peptides (in length and composition) were produced from a pool of 200 mM amino acids under activation with CDI yielding NCAs that could polymerise to yield short peptides.

For several combinations, a pellet was separated from the supernatant after the polymerization. For that, the sample was vortexed for 1 min and centrifuged for 5 min at 10,000 G. The supernatant was then separated from the pellet. For the precipitation extent experiments, both parts were lyophilized overnight (around 16 h). Then, both samples (supernatant and pellet) were weighed and compared (Fig. S5).

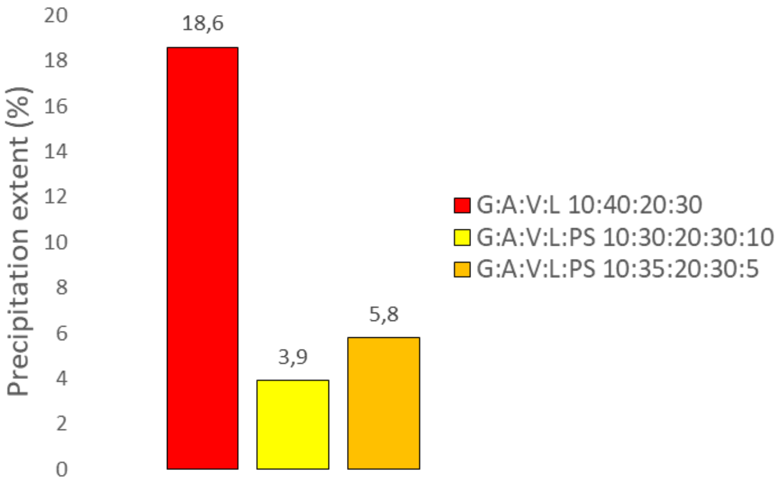

Figure S5. Precipitation extents of peptides generated from several combinations of amino acids: Glycine (G), alanine (A), valine (V), leucine (L) and *O*-phospho-serine (PS) (ratios given in molar percentage). In a combination containing only neutral and lipophilic amino acids (G:A:V:L) a relatively high extent of precipitation was observed for the peptides formed (around 19 %). The addition of a polar and charged amino acid (PS) at random positions allowed to decrease the precipitation extent.

Following the GMPP, the activations of 12 mM *O*-phospho-L-serine (PS) (Fig. S6) and 12 mM azido-L-alanine (AA) (Fig. S7) into their NCAs by 2.5 equivalents of CDI were assessed using ^{1}H NMR spectroscopy.

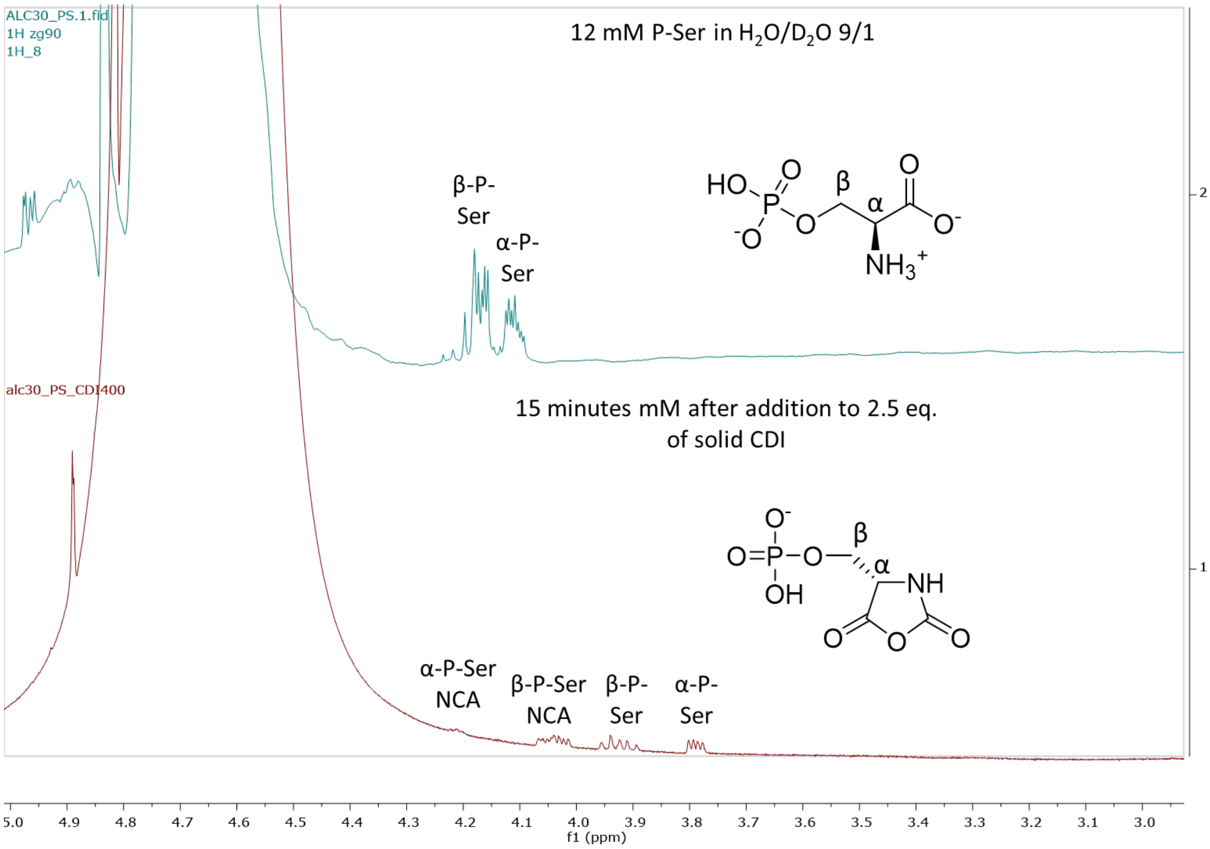

Figure S6. ^{1}H NMR at 300 MHz and 400 MHz of 12 mM *O*-phospho-L-serine in H_2O/D_2O 9:1 recorded before (upper spectrum) and 15 minutes under agitation after the addition of 2.5 equivalents of solid CDI (lower spectrum).

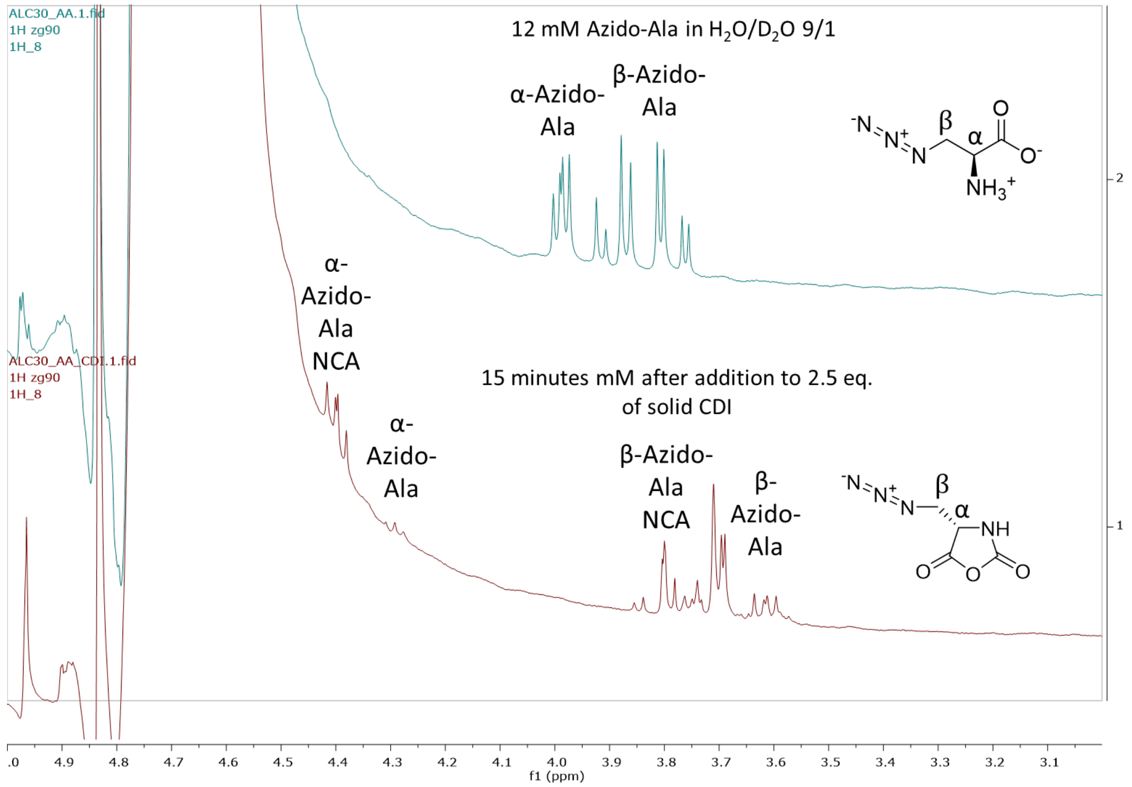

Figure S7. ^{1}H NMR (300 MHz) of 12 mM azido-L-alanine in H_2O/D_2O 9:1 recorded before (upper spectrum) and 15 minutes under agitation after the addition of 2.5 equivalents of solid CDI (lower spectrum).

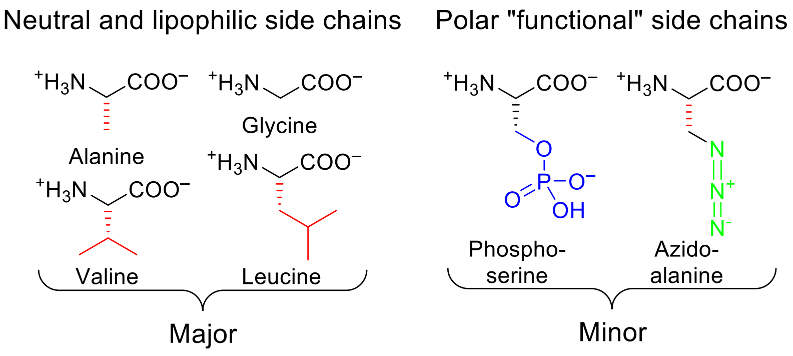

Figure S8. Structures of the amino acids selected for the production of lipophilic and prebiotic peptides.

In order to attribute the peaks found using NMR after activation, the NMR spectra were recorded before and after activation (Fig. S9). This information, in addition to the standards (Fig. S1-4, S6-7), enabled us to determine the origin of each peak in the region of interest. Then, the integration to calculate the activation and the oligomerisation extents could be performed (Fig. S10).

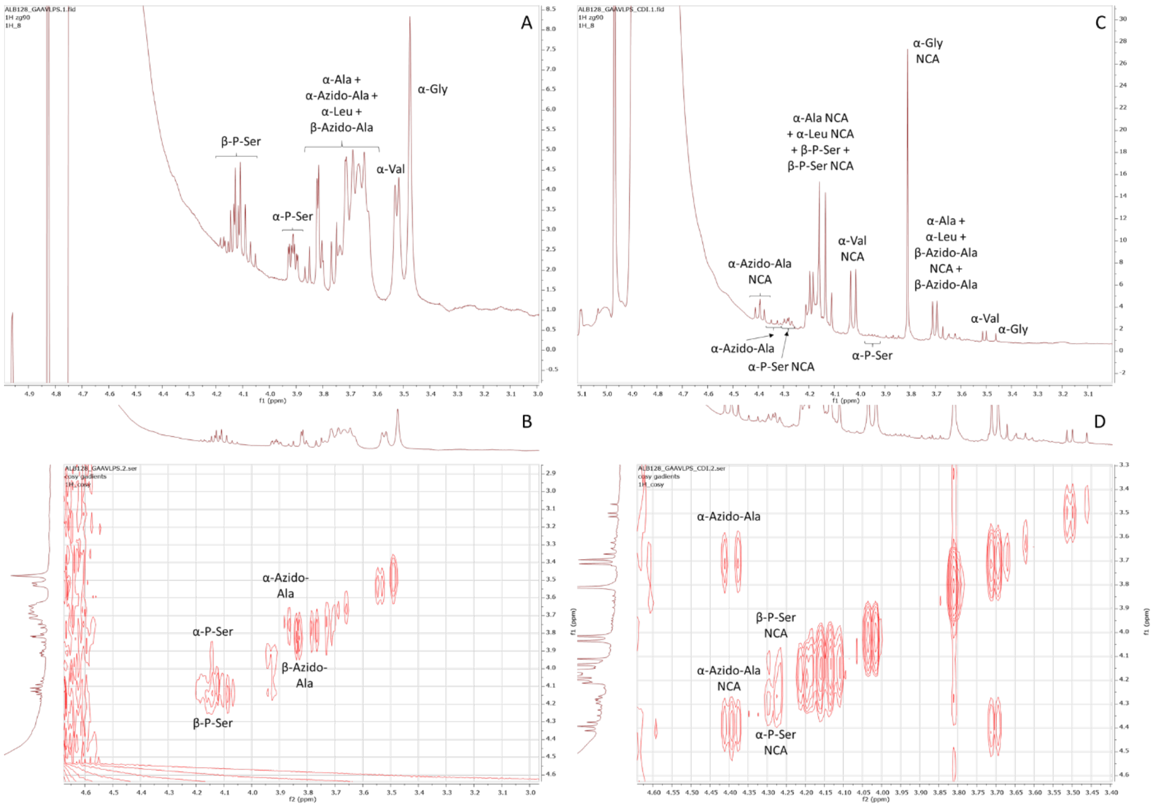

Figure S9. ^{1}H NMR and COSY spectra (300 MHz) allowing the attribution of the peaks found after activation with CDI. **A**) Example of ^{1}H NMR spectrum obtained from the GAVLPSAA combination before activation with CDI. The origin of all the peaks in the region of interest could be determined thanks to the standards (Fig. S1-4 and S6-7) and **B**) the corresponding COSY. **C**) Example of ^{1}H NMR spectrum obtained from the GAVLPSAA combination after activation with CDI. The most important peaks in the region of interest could be attributed thanks to the standards (Fig. S1-4 and S6-7) and **D**) the corresponding COSY.

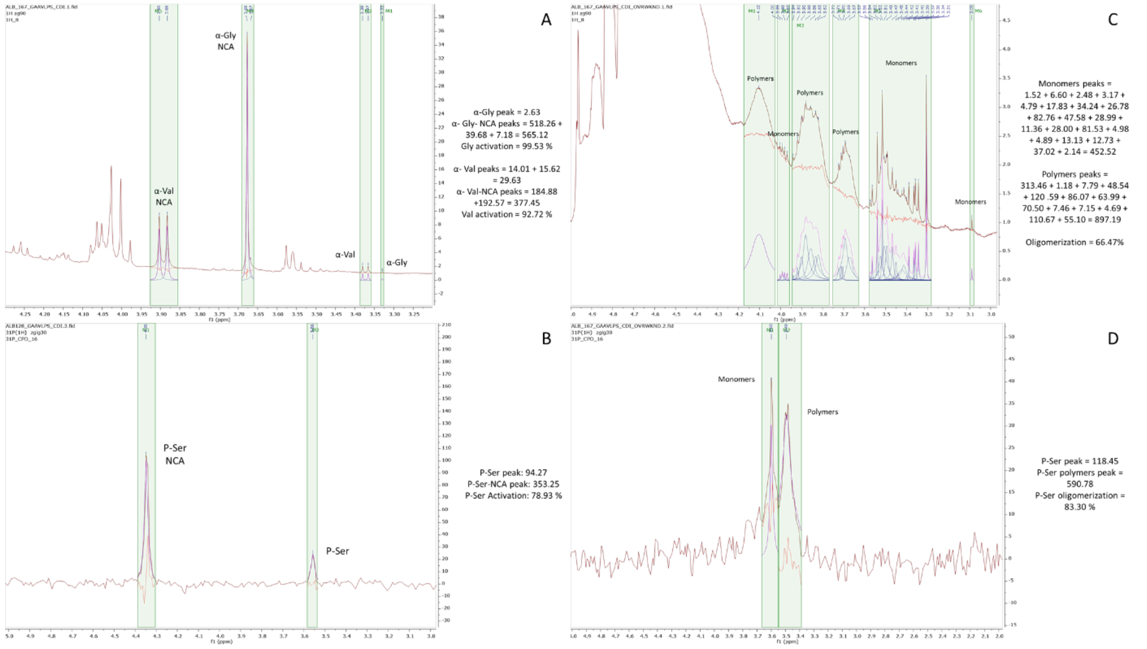

Figure S10. Examples of NMR spectra (**A**, **C**: ^{1}H, 300 MHz and B, D: ^{31}P, 120 MHz) and integration (area values) of deconvoluted peaks (Mestre software) used to calculate the (**A**, **B**) activation and (**C**, **D**) oligomerization extents of the different amino acids of the GAVLPSAA combinations.

2)2) MS characterizations of the random peptides generated in GAVLPSAA mixtures

The GAVLPSAA peptides were analysed using reversed-phase LC-ESI-MS (positive ion mode) with a C18 *Gemini*^{TM} column (5 µm, 250 × 4.6 mm, *Phenomenex*®). Eluants A (5 mM NH_4OAc in H_2O) and B (CH_3CN/A 95:5) were used with various linear binary gradients operating from 2 % B to 50 % B in 30 min at 1.0 mL/min (Fig. S11, see below and the following 3 pages).

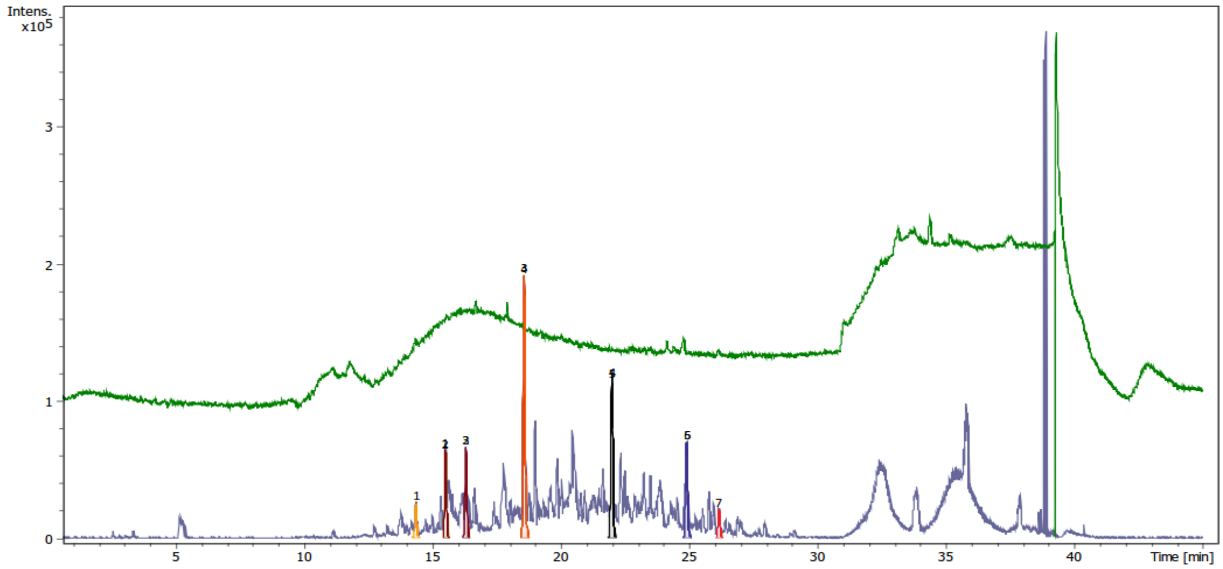

- Composé 1 - Tr = 14.5 min

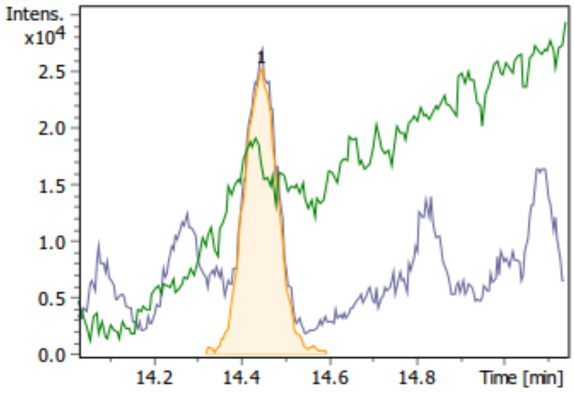

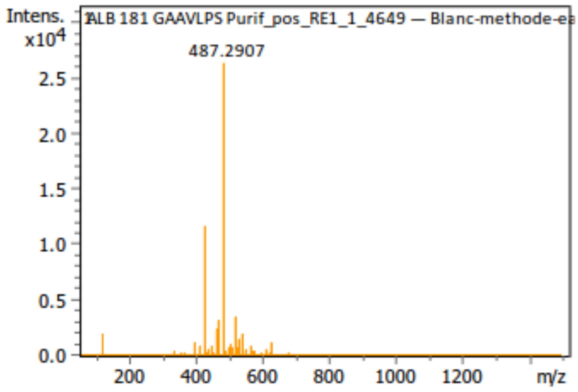

Unidentified compound

- Composé 2 - Tr = 15.6 min

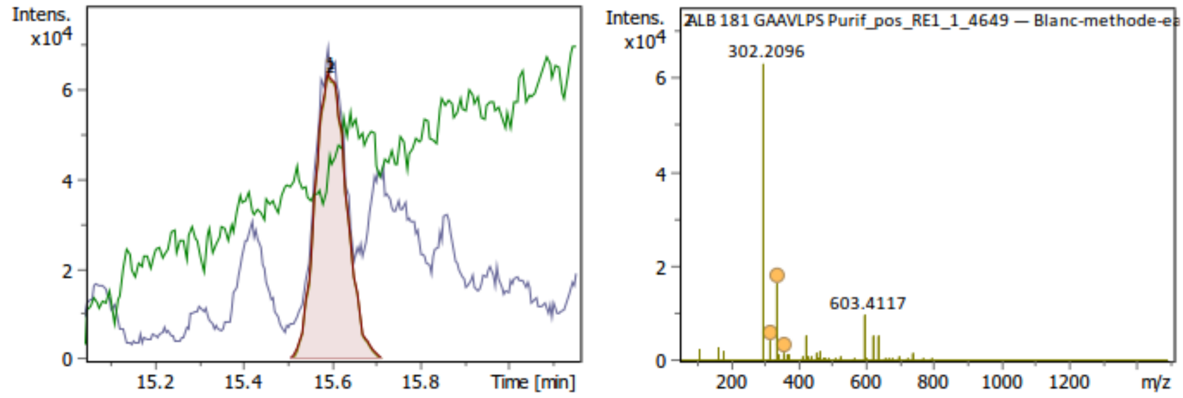

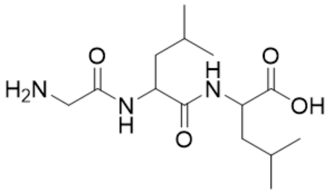

Chemical Formula: $C_{14}H_{27}N_3O_4$
Exact Mass: 301,20

Gly-Leu-Leu (or Leu-Gly-Leu or Leu-Leu-Gly)

- Composé 3 - Tr = 16.4 min

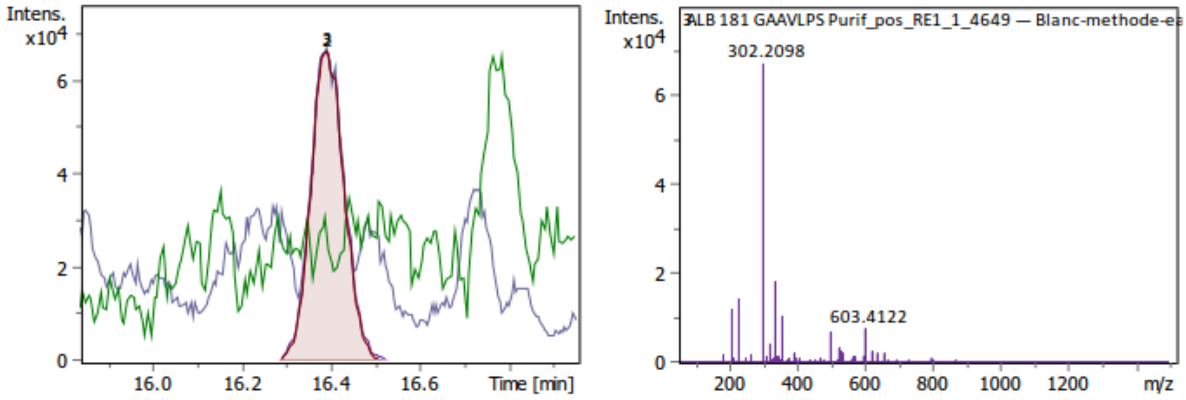

Gly-Leu-Leu (or Leu-Gly-Leu or Leu-Leu-Gly)

- Composé 4 - Tr = 18.7 min

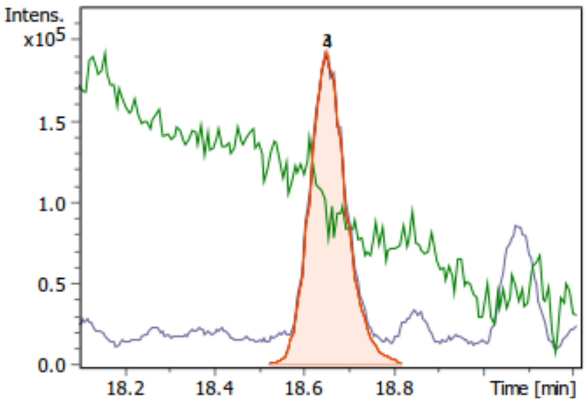

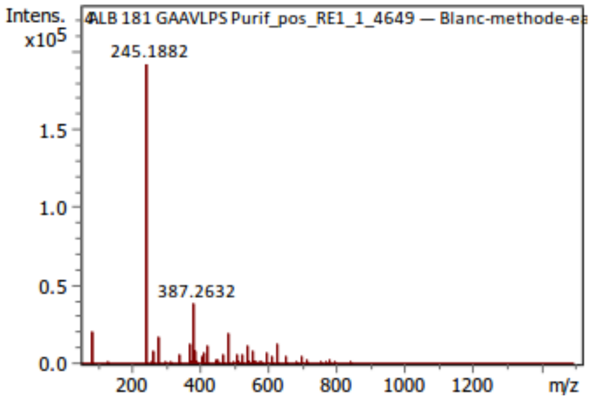

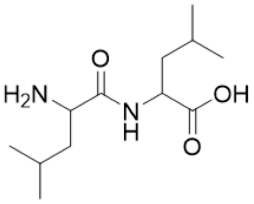

Chemical Formula: $C_{12}H_{24}N_2O_3$
Exact Mass: 244,18

Leu-Leu

- Composé 5 - Tr = 22.1 min

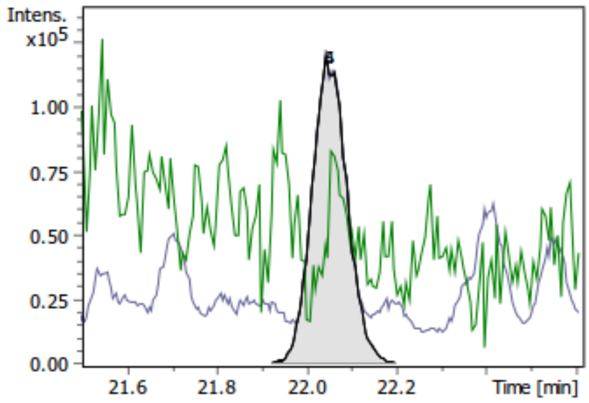

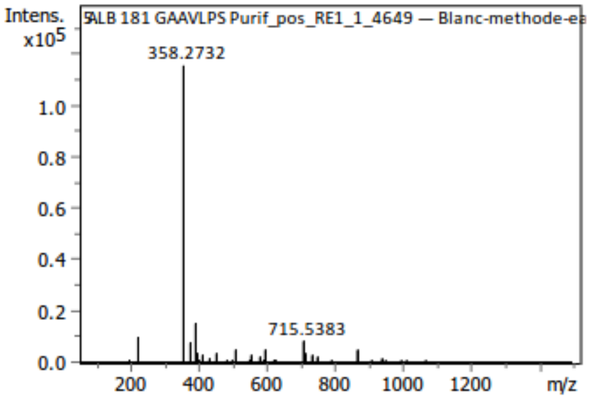

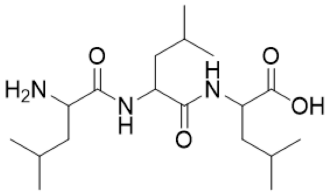

Chemical Formula: $C_{18}H_{35}N_3O_4$
Exact Mass: 357,26

Leu-Leu-Leu

- Composé 6 - Tr = 25.0 min

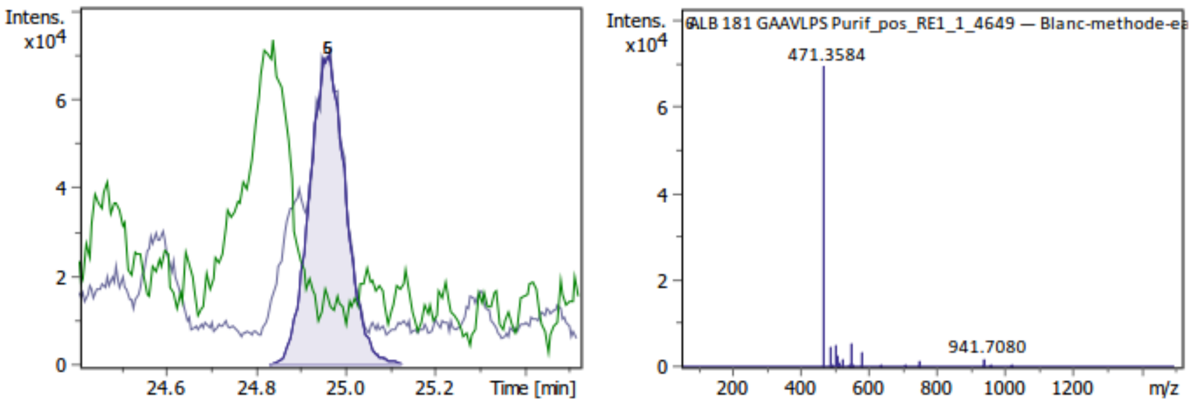

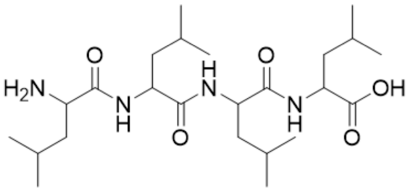

Chemical Formula: $C_{24}H_{46}N_4O_5$
Exact Mass: 470,35

Leu-Leu-Leu-Leu

The size of the peptides present was obtained using ion-extraction of the LC-ESI-MS integrating the area of ten intervals of masses that represented the estimated average masses of the peptides generated from GAVLPSAA mixtures (Fig. S12).

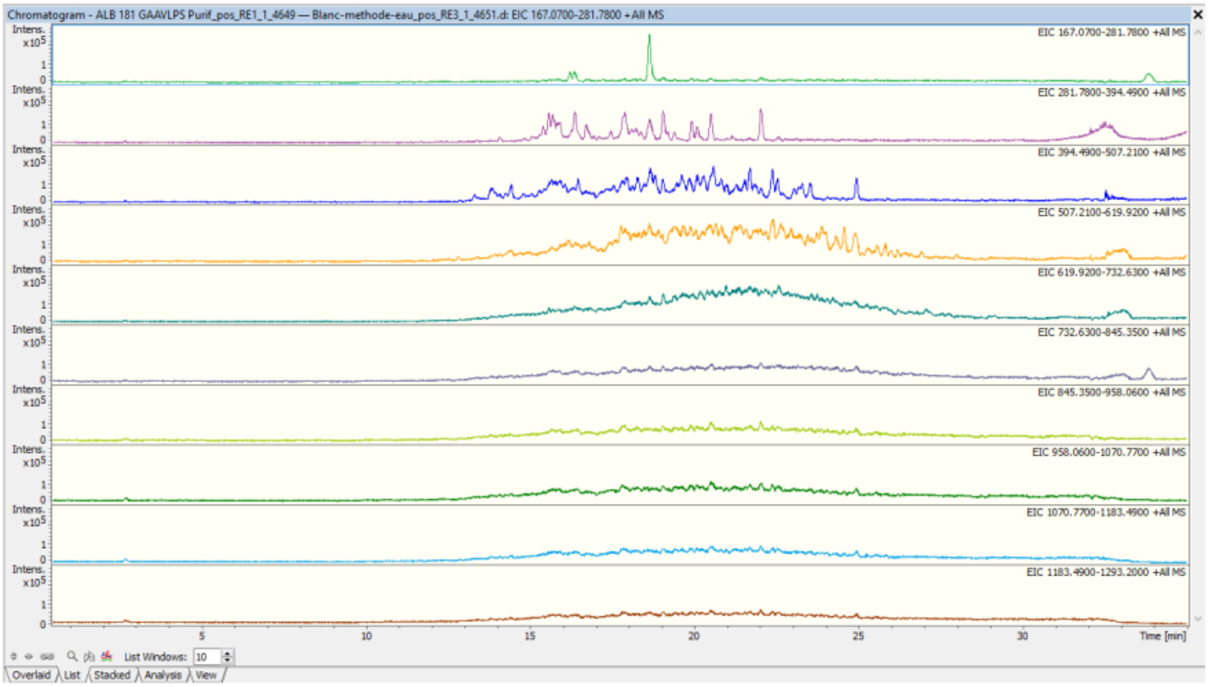

Figure S12. LC-ESI-MS in positive ion mode: extracted ion chromatograms (EICs) for ten ranges of extracted masses representing the estimated average mono-charged masses of short peptides generated from GAVLPSAA combinations; from average-molecular-weight 2-mers (top EIC) to average-MW 11-mers (bottom EIC). The integrated areas of each mass range, cf. upper right {*m/z* range, area} values for each EIC range, are depicted in a histogram in Fig. 3B.

GAVLPSAA mixtures were also analysed using MALDI-ToF-MS. For such analyses, most of the imidazole was separated from peptides using chromatography using a Sephadex G-10 column. The supernatant of the GAVLPSAA mixture was analysed as such, whereas the pellet was first dissolved in pure trifluoroacetic acid (1 mg pellet in 10 µL TFA). Then, these 10 µL were added to 990 µL of H_2O for the MS analysis (Fig. S13).

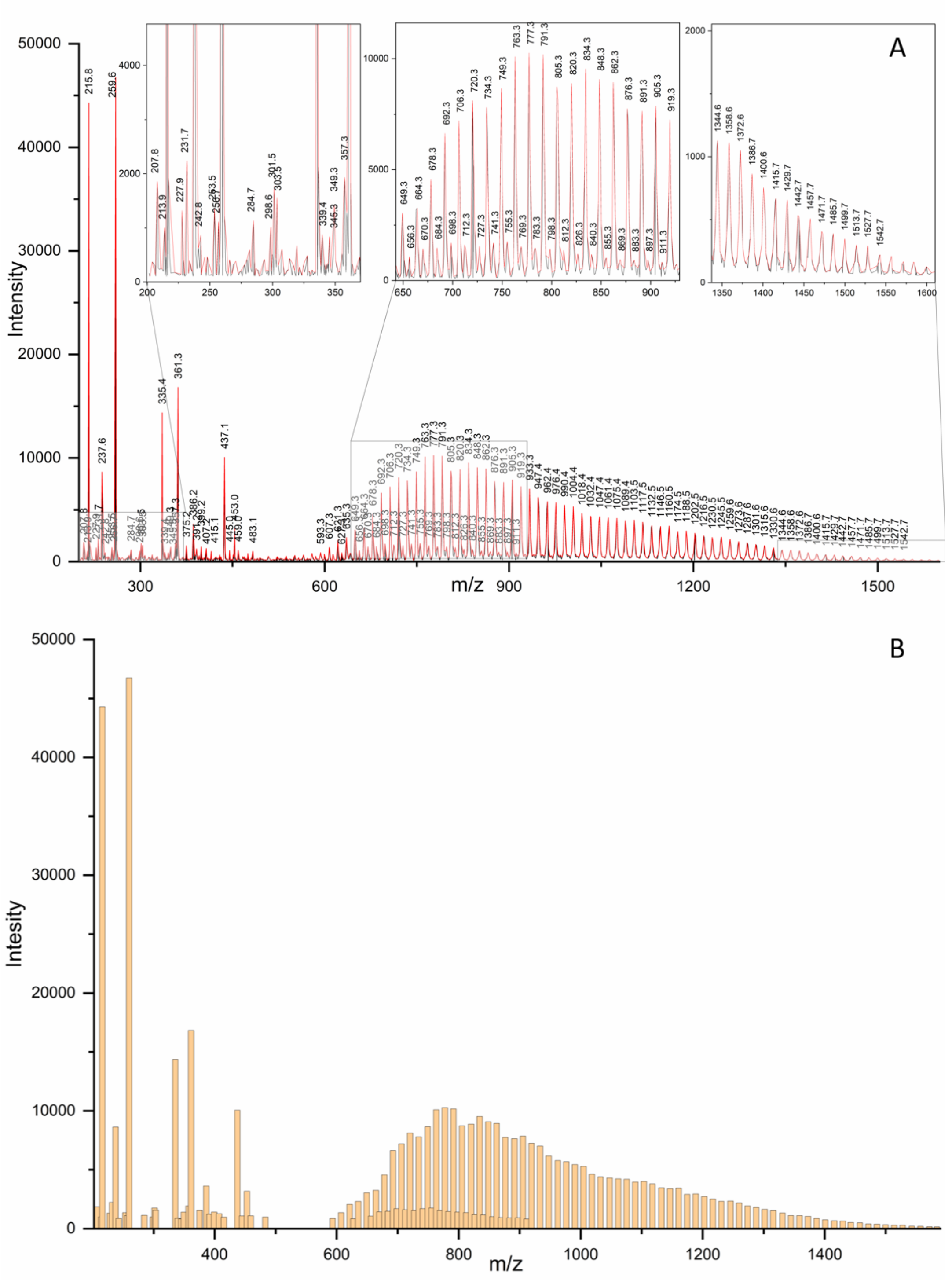

Figure S13. MALDI-ToF-MS analysis was performed on GAVLPSAA mixtures on the pellet isolated using centrifugation, after polymerization mediated by CDI and purification through a Sephadex G-10 column. The raw data (black line in inserts) was convoluted to depict **A**) groups of single peaks each within 5 Da (red lines) and **B**) a histogram gathering two sub-populations of peaks in a same range.

2)3) HPLC characterizations of the random peptides generated in GAVLPSAA mixtures (click reactions)

In order to assess the presence of azido-alanine in the random GAVLPSAA peptides, clicked reactions with cyclooctyne-containing fluorophores (DBCO-AF488 and DBCO-PEG$_4$-5/6-FAM) were performed, but first we carried out control reactions with pure azido-alanine (Scheme S2).

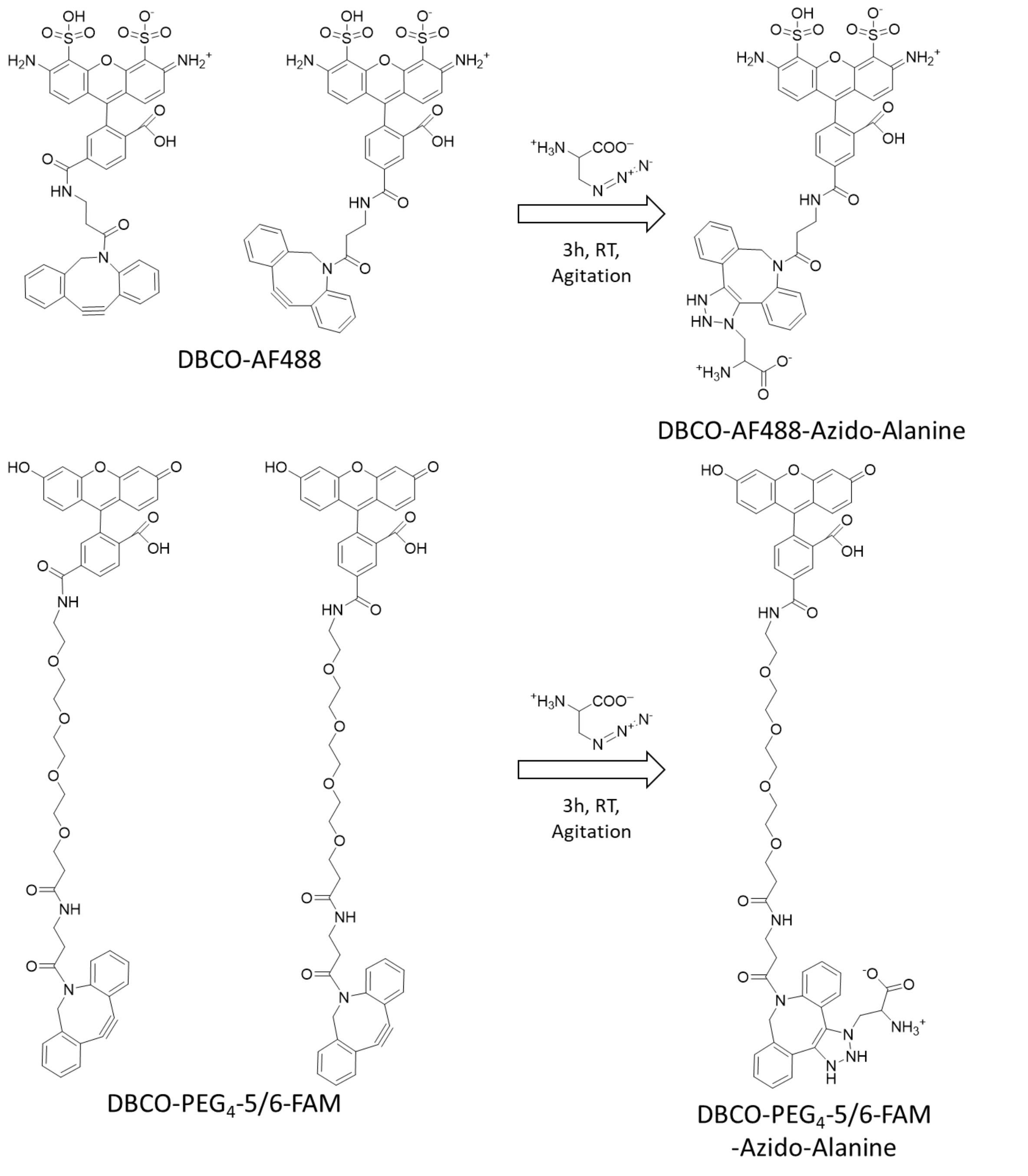

Scheme S2. Click reactions between two fluorescent clickable hydrophilic probes: DBCO-AF488 and DBCO-PEG$_4$-5/6-FAM (isomer mixtures shown only for the unclicked) and azido-alanine yielding a fluorescent modified amino acid. If azido-alanine is present inside peptides (as in GAVLPSAA mixtures), then fluorescent peptides can be yielded in this way. These reactions were performed with 50 µM fluorescent probe and 1 mM azido-alanine during 3 h at room temperature under slow agitation (300 RPM).

The result of the reaction and the other click-reactions was analysed by RP-HPLC using a *Gemini*^{Tm} C18 column (250 × 4.6 mm, 5 µm, *Phenomenex®*). The fluorescent compounds were separated with a binary linear gradient from 2 to 56 % B in 27 min at 1.0 mL/min using eluants A) 5 mM NH_4OAc pH 8.0 and B) CH_3CN/A 95:5 and detected using fluorescence at 517 nm emission wavelength (excited at 493 nm).

Both probes were diluted 20 times in 1 mL of HPLC eluent A from concentrated solutions in DMSO (1 mM, 1 mg in 1.26 mL DMSO (DBCO-AF488) or 1 mg in 1.13 mL DMSO (DBCO-PEG_4-5/6-FAM)) (50 µM final concentration after dilution). Then, they were separately injected as HPLC references. Azido-alanine was added through a 10-fold dilution in eluent A (10 mM, 1.30 mg in 1 mL) (1 mM final concentration after dilution) to these solutions to yield the clicked fluorescent amino acid. After 3 h at RT and under slow agitation (300 rpm), these solutions were injected and analysed similarly using HPLC (Fig. 4A). Moreover, both probes (50 µM each) were separately and independently incubated in similar conditions with around 20 mM GAVLPSAA solution (thus, the concentration of azido-alanine should be more or less of 1 mM), in order to generate clicked peptides. To estimate the concentration of peptides in solution after polymerisation with CDI, the amount of salts, imidazole, non-polymerised amino acids (obtained with the oligomerisation extent of all amino acids previously determined (Fig. 2F) and the average masses of the obtained peptides (≈ 600 Da, Fig. 3) were all taken into account. In the end, 9.70 mg lyophilised GAVLPSAA mixtures were dissolved in 1 mL eluent A to yield a solution of about 50 mM and diluted 2.5 times when added to the probes in 1 mL eluent A (20 mM final concentration in 1 mL, final volume). The reaction was left for 3 h at RT under slow agitation (300 rpm). In order to highlight the diversity of the clicked peptides generated, spiking experiments were performed. To do so, 5 µL of the previous reaction solutions (GAVLPSAA + probes) were injected with 0.5 µL of the probes alone (50 µM) or with 0.5 µL of the probes clicked with azido-alanine (50 µM and 1 mM respectively) (Fig. S14).

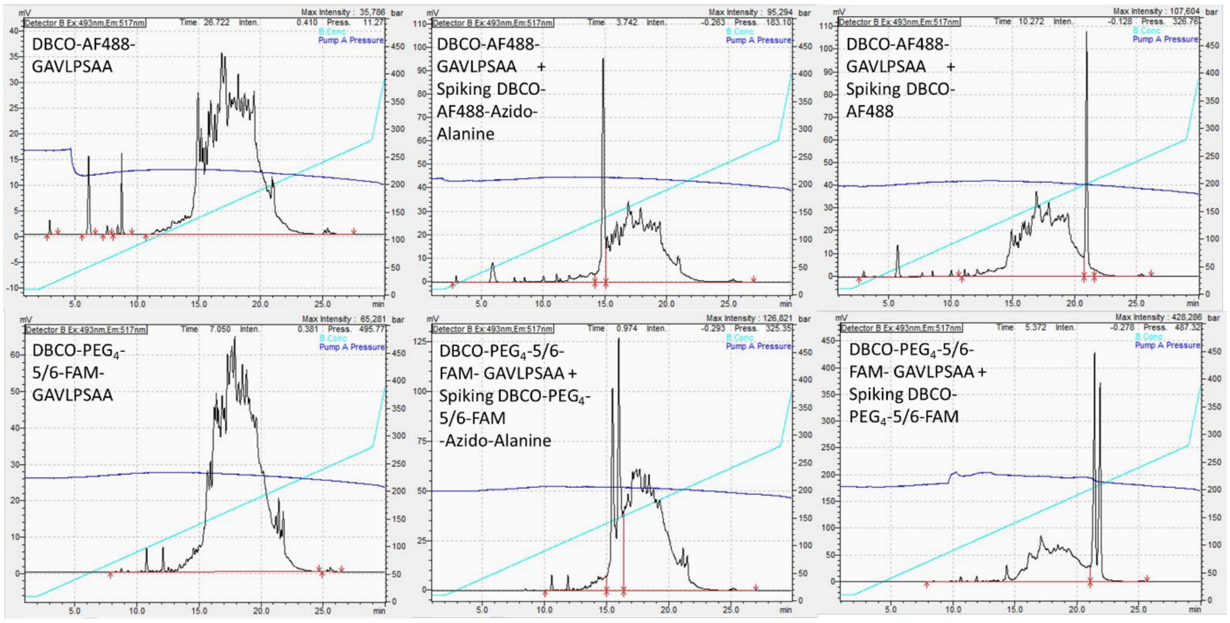

Figure S14. HPLC chromatograms obtained by fluorescence detection (493/517 nm excit/em) from spiking injections after reaction of GAVLPSAA with the clickable fluorescent probes (left column). Spiking with azido-alanine clicked to the probe of interest (central column) and with the probe of interest alone (right column) showed that many GAVLPSAA peptides contained azido-alanine and were able to click to the fluorescent probes.

After the reactions of GAVLPSAA mixture with the fluorescent probes, 50 µM of both fluorescent probes were separately added from concentrated solutions and a second click reaction was left overnight at room temperature under slow agitation (300 rpm). The day after, both solutions were

injected using HPLC and showed that more azido-alanine-containing peptides were still available for the click reaction to occur (Fig. S15).

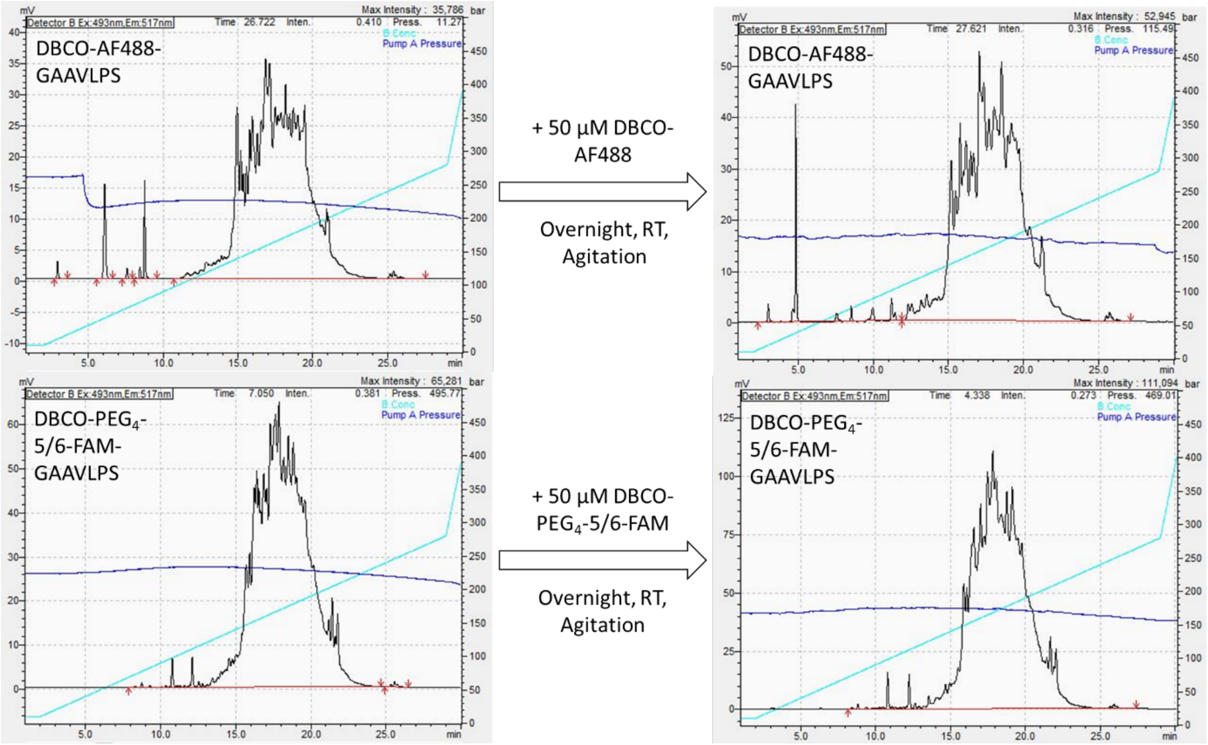

Figure S15. HPLC chromatograms obtained using fluorescence detection (493/517 nm excit/em) before and after a second click reaction. Additional fluorescent probes (50 µM) were added to clicked GAVLPSAA mixtures (left part) and the reaction was left overnight at RT under slow agitation (300 rpm). Then, the resulting mixtures were analysed using HPLC (right part). All the additional added probes were consumed yielding more GAVLPSAA fluorescent peptides.

3) Supplementary information to: Coupling attempts of peptides with oligonucleotides in prebiotic conditions compatible with the presence of lipidic giant vesicles

The prebiotic coupling reaction investigated corresponded to the formation of peptido-oligo-nucleotides after the activation of the terminal phosphate of oligonucleotides with the action of EDC. The additional step involving the formation of another intermediate after addition of the nucleophilic catalyst was omitted for simplicity (Scheme S3).

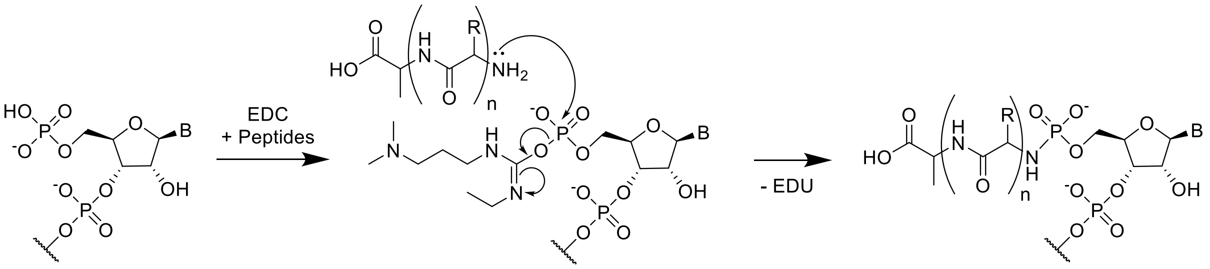

Scheme S3. The formation of peptide-oligonucleotides, as vectors of peptides and oligonucleotides to get inside lipidic giant vesicles, was studied in the case of peptido-oligonucleotides with 5'-phosphorylated oligonucleotides. Such conjugates (chimeras) should be obtained after activation of the 5'- oligonucleotide phosphate by a coupling agent such as EDC. B stands for nucleobase.

3)1) pUGGU RNA production and analysis

First of all, it was necessary to produce the 5'-phosphorylated 4-mer RNA pUGGU used as a standard for all coupling experiments. The solid-supported synthesis of the oligonucleotide was achieved with a default program (DMT-OFF) containing small modifications allowing to increase the stepwise and overall yields. The standard 10 µmole scale-cycle was used on an amount of commercial solid support that was charged with 6 µmol of the first (3'-terminal) nucleoside and using a 10 µmole-scale column in order to increase the overall yields. With the same aim, an additional capping step was introduced after the aqueous iodine oxidation step, making the solid-support more anhydrous for the subsequent detritylation and coupling. Additional washings with DCM were also added before and after detritylation to minimize the chances of detritylating added phosphoramidite monomers before they would couple to the solid-support. Besides, the waiting times were increased to 15 min for the activation/coupling of monomers.

The de-immobilizations and deprotections were performed on a 2 µmole scale (1 eq.). The corresponding amount of resin obtained after solid-phase synthesis was taken and 0.5 mL of aqueous ammonia solution (35 % *v/v*) then 0.5 mL of ethanolic methylamine solution (33 % *v/v*) were added (around 500 eq. each) [1]. The deprotection reaction was performed in closed Eppendorf vials placed in a thermomixer heating at 65°C and 400 rpm during 20 min. Then, the Eppendorf vial was cooled down for 30 min in an ice bath and the supernatant containing the free RNA was collected. In order to recover most of the deprotected and de-immobilized oligonucleotides, the resin was washed 4 times (500 µL of ultrapure water added, brief vortex and supernatant collected). Next, the Eppendorf was placed in a Speedvac for 20 min at 43°C to get rid of the organic solvents. Thereafter, the residual water was removed by an overnight (around 16 h) lyophilization. 0.7 mL of Et_3N-3HF was added (around 500 eq.) on the lyophilised pellet and the sample was treated in a thermomixer at 65°C for 1.5 h [2]. Again, the Eppendorf vial was cooled down in an ice bath for 30 min. The sample liquid was added to 5 mL neat methoxytrimethylsilane to remove all excess fluoride as gaseous trimethylsilyl fluoride (and methanol) formed [3]. Afterwards, the sample was vortexed for 2 min and the organic volatiles (Et_3N, MeOH) were evaporated for 20 min in a Speedvac at 43°C. Finally, the yellow pellet was dissolved in 1 mL of ultrapure water and lyophilised overnight to get rid of any residual organic volatile leftovers. The lyophilised pellets were stored at –80°C before purification.

Clarity Oligo-RP™ columns from *Phenomenex*® (5 µm, 250×10 mm, for the semi-preparative column and 5 µm, 250×4.6 mm for the analytical column) were used for the separation or the purity analysis of the oligonucleotide. It was purified using semi-preparative RP-HPLC at 5.0 mL/min and 60°C column temperature using a binary gradient of A) 10 mM NH_4OAc pH 8.0 and B) CH_3CN/A 95:5. The purity of the oligonucleotide HPLC fractions were assessed using analytical RP-HPLC at 1.0 mL/min and 60°C using 260 nm UV detection. The gradients used varied from 2 to 8 % of B in 8 min. The yield of the oligonucleotide was calculated using the optical density at 260 nm. For that, the absorbance A_{260} of the pure sample obtained after HPLC separation was measured and the corresponding yield was calculated with OligoCalc™ (Fig. S16).

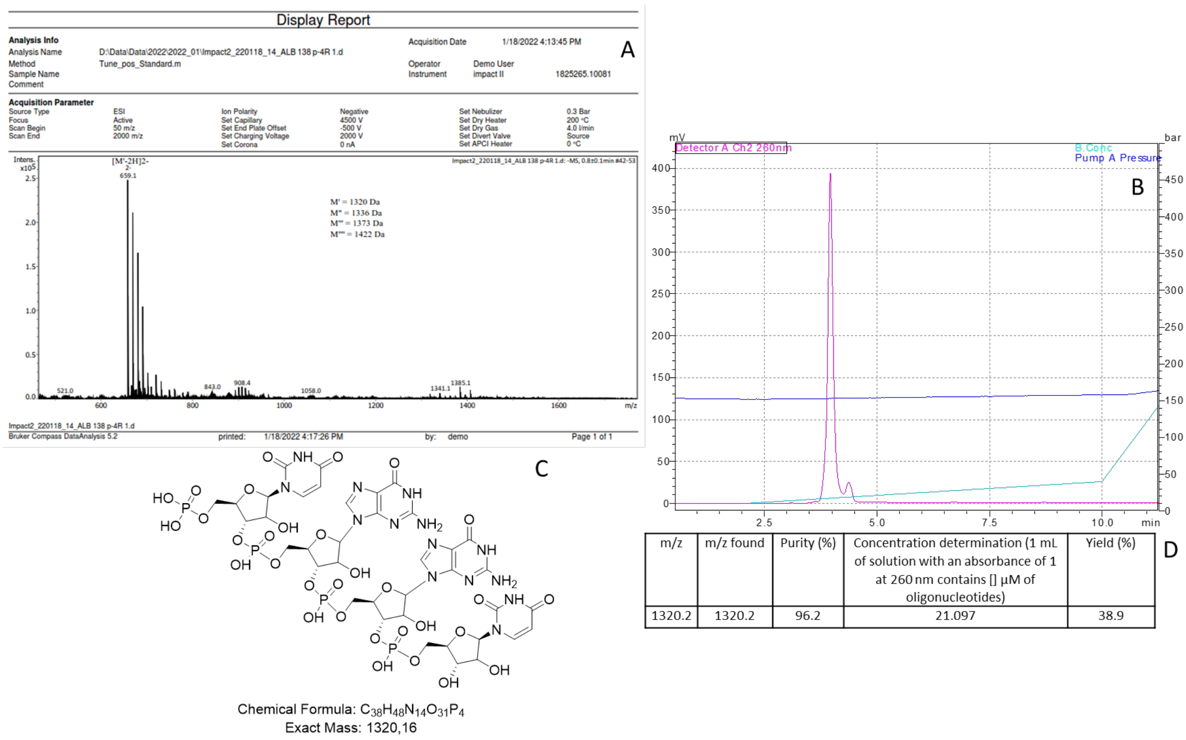

m/z	m/z found	Purity (%)	Concentration determination (1 mL of solution with an absorbance of 1 at 260 nm contains [] µM of oligonucleotides)	Yield (%)
1320.2	1320.2	96.2	21.097	38.9

Figure S16. Chemical analysis of the RNA 4-mer pUGGU produced. **A**) MS analysis (negative ion mode, main compound found as dianion), **B**) UV chromatogram (260 nm) and **C**) chemical structure, formula and molecular weight of the main compound. **D**) Table showing *m/z* ratio calculated for the main oligonucleotide, *m/z* of the product found by MS analysis (both for $z = -1$ (mono-anion)), the purity (determined from the UV chromatogram), the micromolar concentration of an oligonucleotide solution in 1 mL to have an absorption $A_{260} = 1.000$ through 1 cm path length (calculated with OligoCalc™ and the yield calculated from the absorption coefficient and the A_{260}.

3)2) Coupling attempts and vesicles production in the presence of EDC and EtIm

The coupling experiments between the RNA pUGGU and GAVLPSAA peptides were performed in 2 steps:

Activation step: The previously synthesized RNA pUGGU (50 µM, 1 eq, concentration calculated from the UV absorbance at 260 nm and the online service OligoCalc™) was added to 7.5 equivalents of EtIm (375 µM, 3.60 mg in 1 mL of ultrapure water for a concentrated solution of 37.5 mM later diluted 100 times to reach the desired concentration) in ultrapure water. Then, the pH was adjusted to 7.5 (A1 experiment) or 8.0 (A2 experiment) adding 100 mM HCl. Afterwards, 20 equivalents of EDC (1 mM, A1 experiment) or 40 equivalents of EDC (2 mM, A2 experiment) were added from a concentrated solution (10 mM, 1.55 mg in 1 mL). Eventually, the pH was adjusted to 7.5 (A1 experiment) or 8.0 (A2 experiment) by adding 100 mM NaOH. The activation reaction was left for 2 h (A1 experiment) or 192 h (A2 experiment).

Incubation step: After activation, 20 "equivalents" (1 mM, A1 experiment) or 60 "equivalents" (3 mM, A2 experiment) of the GAVLPSAA mixture were added from a concentrated solution (50 mM, 9.70 mg of lyophilized GAVLPSAA mixtures in 1 mL of ultrapure water) to the activated oligonucleotides. Importantly, the concentration of added peptides refers to an estimation. The amount of peptides in solution after polymerization with CDI could be estimated taking into account the amount of salts, imidazole, non-polymerized amino acids (obtained with the oligomerization extent of all amino acids

previously determined (Fig. 1F)) and the average masses of the peptides yielded (≈ 600 Da, Fig. 2). The pH was then adjusted to 7.5 (A1 experiment) or 8.0 (A2 experiment) adding 100 mM HCl. In the A2 experiment, 1 mM EDC was also added with the second step (from a concentrated solution, similarly as described previously) in order to favor the reaction. The reactions were then left in an Eppendorf vial for 120 h (A1 experiment) or 360 h (A2 experiment) at ambient temperature and without shaking. The pH was checked regularly and remained stable around the pH set after the addition of GAVLPSAA mixtures. 50 µL of the solutions were sampled during the reaction or at least the end of the incubation for LC-MS analysis.

All the products of reaction were analyzed using LC-MS using a *Gemini*™ C18 column (250 × 4.6 mm, 5 µm, *Phenomenex*®). The oligonucleotides were separated at 1.0 mL/min using a binary linear gradient from 2 to 44 % B in 28 min using eluants A) 5 mM NH_4OAc pH 8.0 and B) CH_3CN/A 95/5 (Fig. S17-20).

A1 experiment – 4 h:

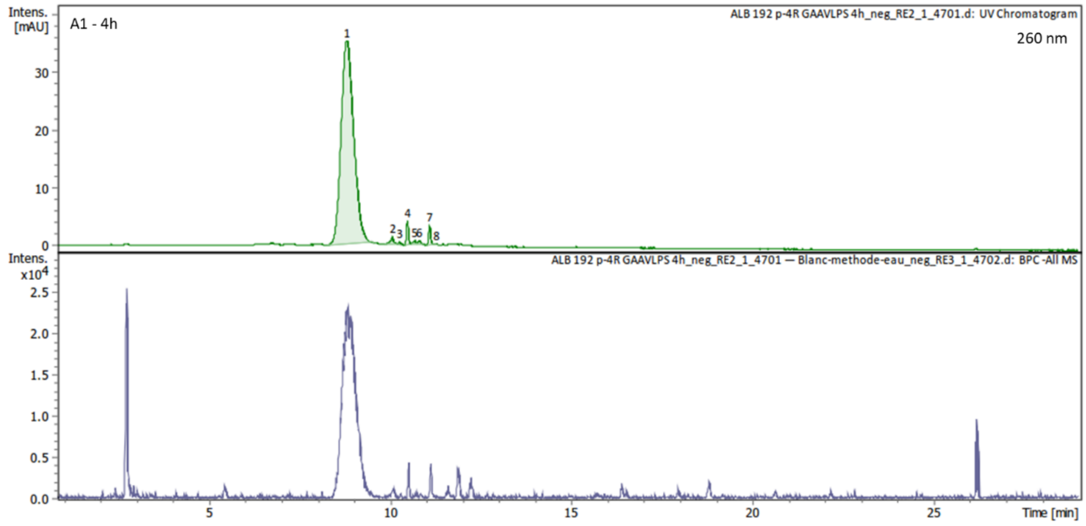

- Composé 1 - Tr = 8.8 min

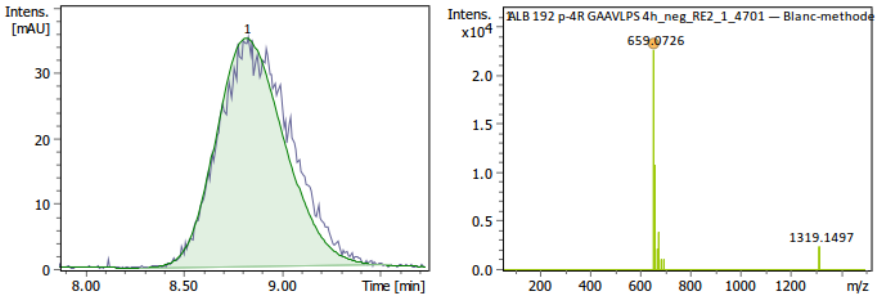

Meas. m/z	Ion Formula	Sum Formula	m/z	err [ppm]	mSigma	Adduct	z
659.0726	C38H46N14O31P4	C38H48N14O31P4	659.0707	-2.8	3.9	M-H	2-

→ Le spectre de masse met en évidence la présence des adduits correspondant à un produit dont la formule brute est la suivante : $\mathbf{C_{38}H_{48}N_{14}O_{31}P_4}$.

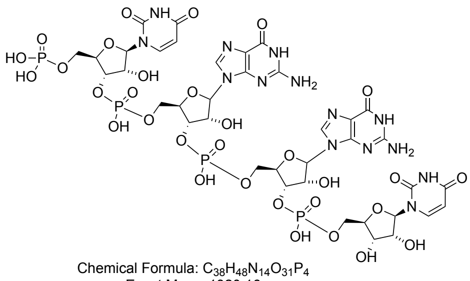

Chemical Formula: $C_{38}H_{48}N_{14}O_{31}P_4$
Exact Mass: 1320,16

pUGGU

- Composé 2 - Tr = 10.1 min

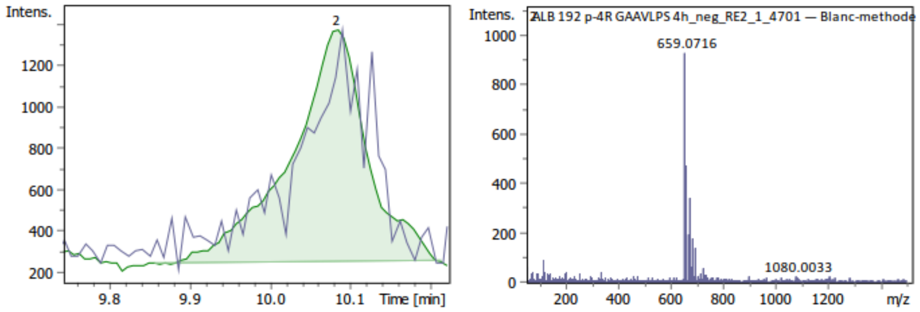

pUGGU

- Composé 3 - Tr = 10.3 min

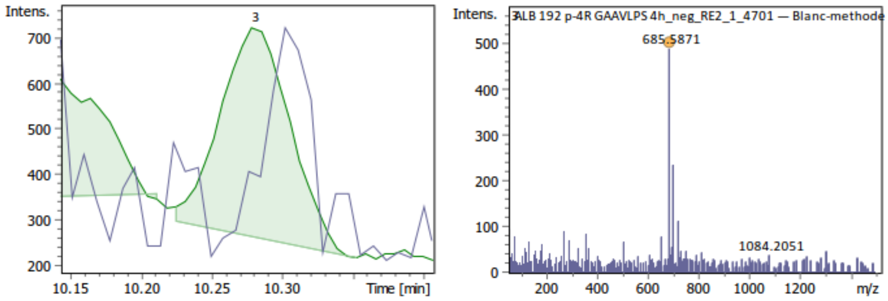

Soit M = 685.5871*2+2 = 1373.1742 Da

Unidentified derivative

- Composé 4 - Tr = 10.5 min

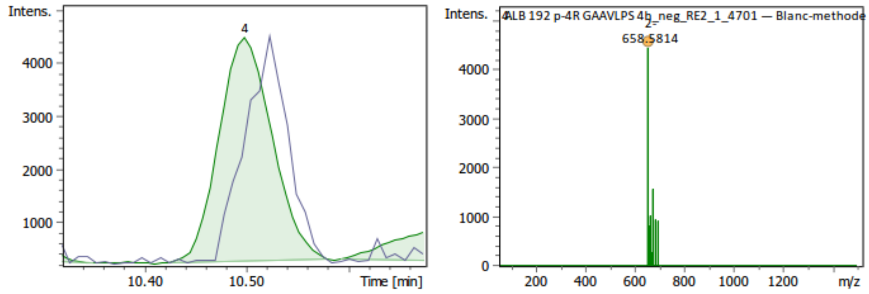

Meas. m/z	Ion Formula	Sum Formula	m/z	err [ppm]	mSigma	Adduct	z
658.5814	C38H47N15O30P4	C38H49N15O30P4	658.5787	-4	16.6	M-H	2-

→ Le spectre de masse met en évidence la présence des adduits correspondant à un produit dont la formule brute est la suivante : **$C_{38}H_{49}N_{15}O_{30}P_4$**.

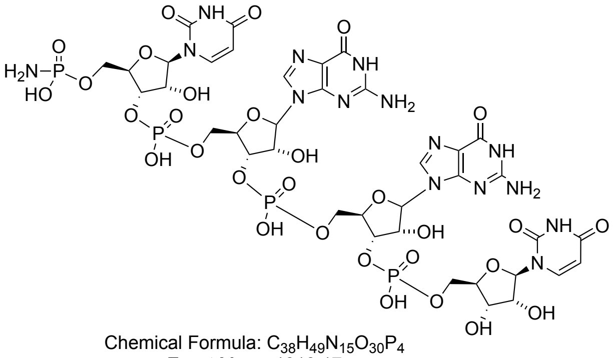

Chemical Formula: $C_{38}H_{49}N_{15}O_{30}P_4$
Exact Mass: 1319,17

H₂N-pUGGU

- Composé 5 - Tr = 10.7 min

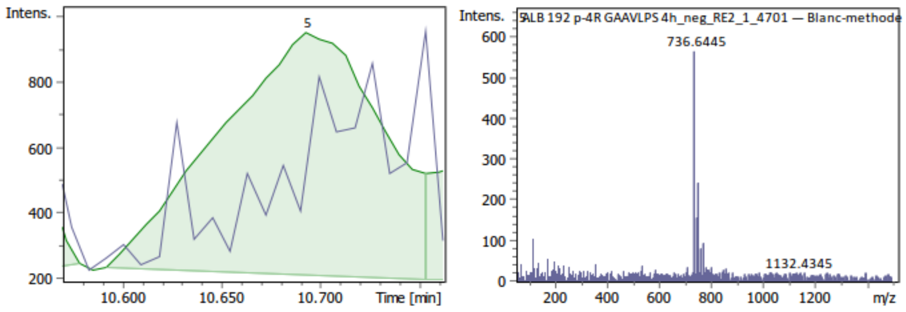

Soit M = 736.6445*2+2 = 1475.2890 Da

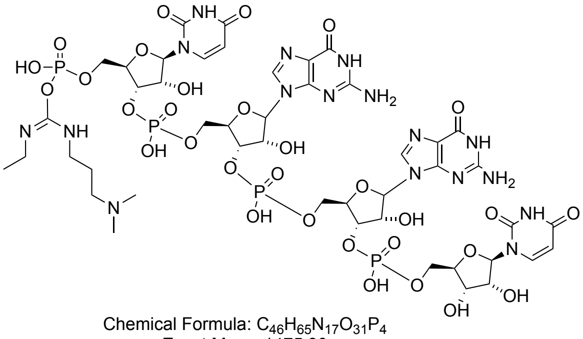

Chemical Formula: $C_{46}H_{65}N_{17}O_{31}P_4$
Exact Mass: 1475,30

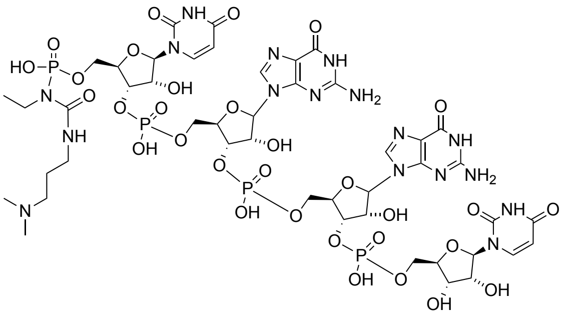

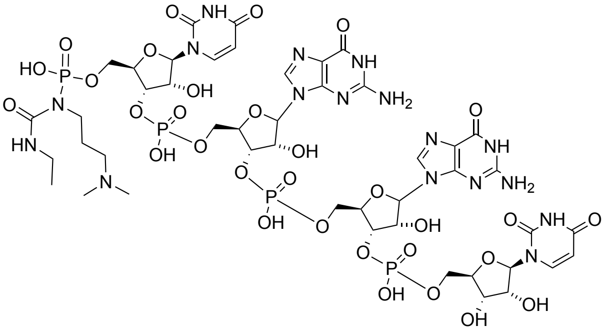

EDC-pUGGU

- Composé 6 - Tr = 10.8 min

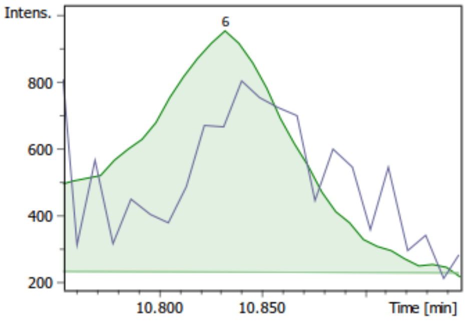

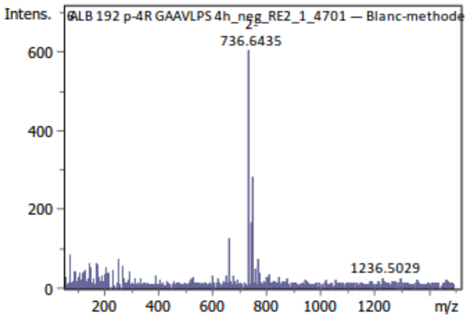

Soit M = 736.6435*2+2 = 1475.2870 Da

EDC-pUGGU

- Composé 7 - Tr = 11.1 min

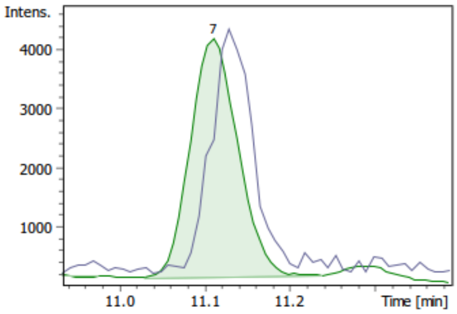

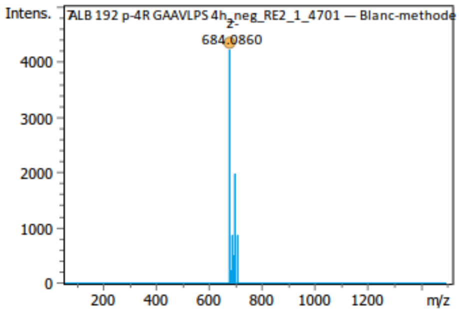

Meas. m/z	Ion Formula	Sum Formula	m/z	err [ppm]	mSigma	Adduct	z
684.086	C41H48N16O30P4	C41H50N16O30P4	684.0842	-2.7	16.3	M-H	2-

→ Le spectre de masse met en évidence la présence des adduits correspondant à un produit dont la formule brute est la suivante : **$C_{41}H_{50}N_{16}O_{30}P_4$**.

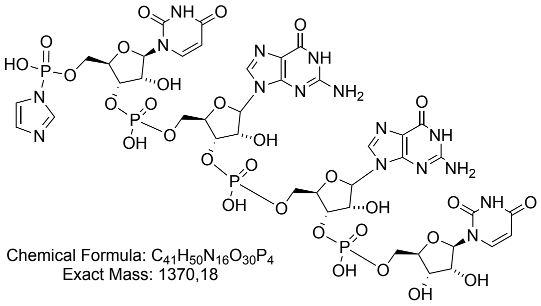

Im-pUGGU

- Composé 8 - Tr = 11.3 min

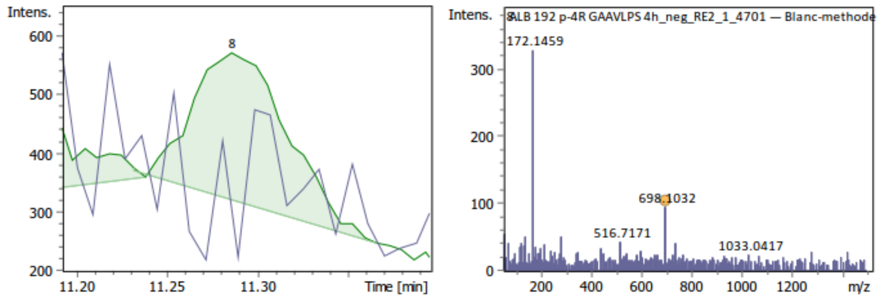

Meas. m/z	Ion Formula	Sum Formula	m/z	err [ppm]	mSigma	Adduct	z
698.1032	C43H52N16O30P4	C43H54N16O30P4	698.0998	-4.8	111.8	M-H	2-

→ Le spectre de masse met en évidence la présence des adduits correspondant à un produit dont la formule brute est la suivante : **$C_{43}H_{54}N_{16}O_{30}P_4$**.

C'est une supposition, l'intensité est vraiment faible, on ne discerne pas de massif isotopique très clair ce qui rend l'identification compliquée.

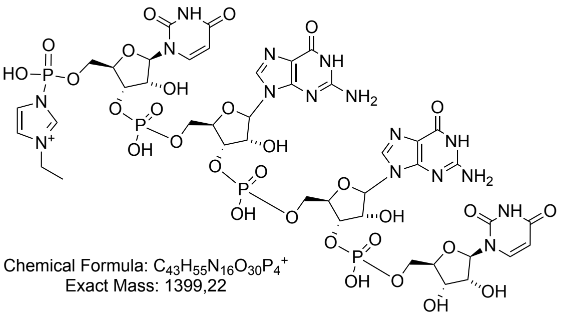

EtIm-pUGGU

Figure S17. UV (260 nm) and LC-MS chromatograms (negative ion mode) with UV peaks and associated compounds obtained for A1 experiment after 4h of reaction with GAVLPSAA mixture.

A1 experiment – 24 h:

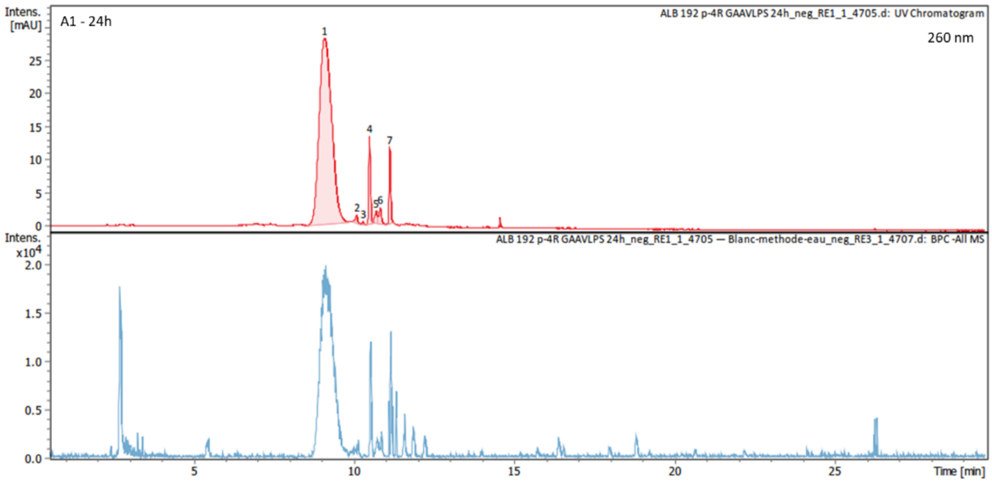

- Composé 1 - Tr = 9.1 min

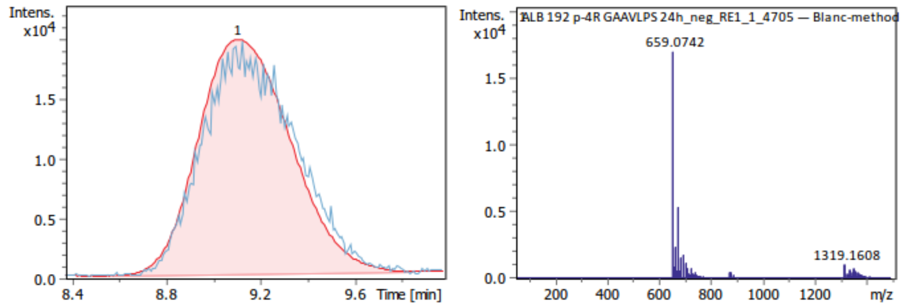

Meas. m/z	Ion Formula	Sum Formula	m/z	err [ppm]	mSigma	Adduct	z
659.0742	C38H46N14O31P4	C38H48N14O31P4	659.0707	-5.2	8.9	M-H	2-

→ Le spectre de masse met en évidence la présence des adduits correspondant à un produit dont la formule brute est la suivante : $\mathbf{C_{38}H_{48}N_{14}O_{31}P_4}$.

pUGGU

- Composé 2 - Tr = 10.1 min

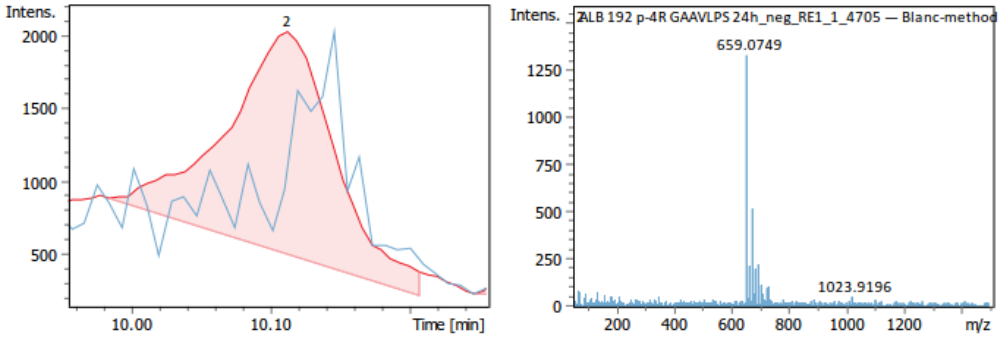

pUGGU

- Composé 3 - Tr = 10.3 min

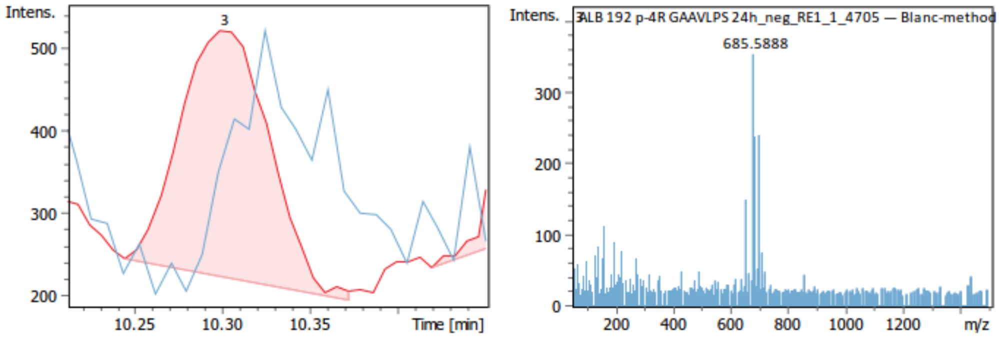

Soit M = 685.5888*2+2 = 1373.1776 Da

Unidentified derivative

- Composé 4 - Tr = 10.5 min

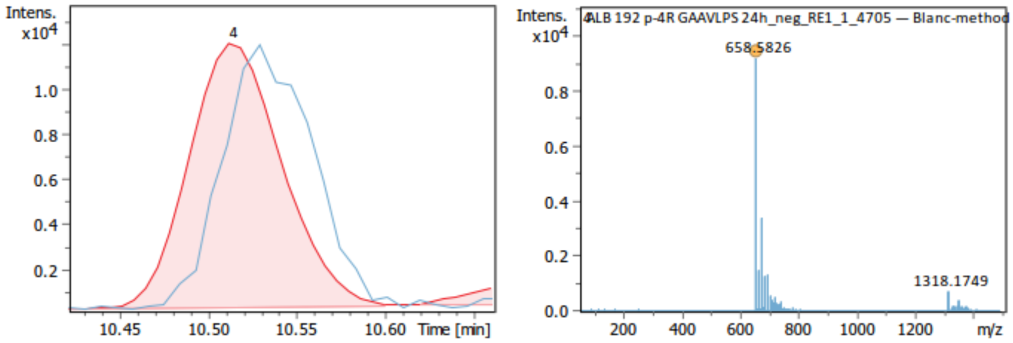

Soit M = 658.5836*2+2 = 1319.1672 Da

H_2N-pUGGU

- Composé 5 - Tr = 10.7 min

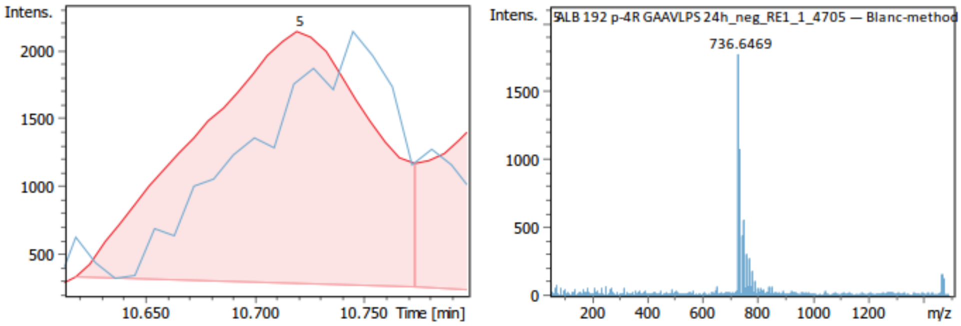

Soit M = 736.6459*2+2 = 1475.2918 Da

EDC-pUGGU

- Composé 6 - Tr = 10.8 min

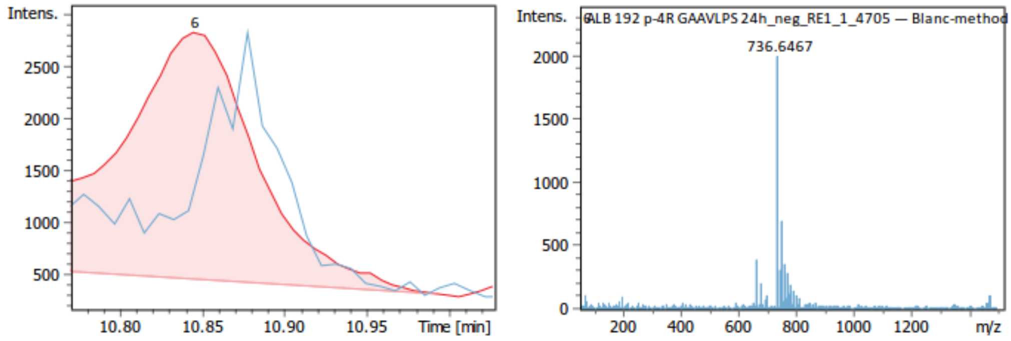

Soit M = 736.6467*2+2 = 1475.2934 Da

EDC-pUGGU

- Composé 7 - Tr = 11.1 min

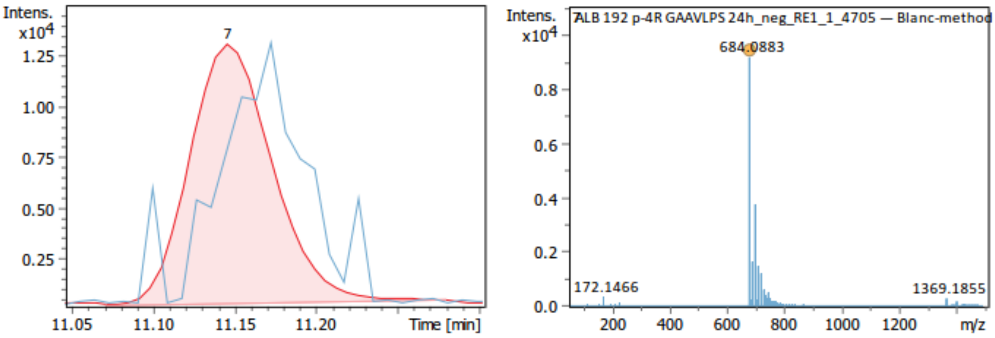

Meas. m/z	Ion Formula	Sum Formula	m/z	err [ppm]	mSigma	Adduct	z
684.0883	C41H48N16O30P4	C41H50N16O30P4	684.0842	-6.1	6.6	M-H	2-

→ Le spectre de masse met en évidence la présence des adduits correspondant à un produit dont la formule brute est la suivante : $\mathbf{C_{41}H_{50}N_{16}O_{30}P_4}$.

Im-pUGGU

Figure S18. UV (260 nm) and LC-MS chromatograms (negative ion mode) with UV peaks and associated compounds obtained for A1 experiment after 24 h of reaction with GAVLPSAA mixture.

A1 experiment – 120 h:

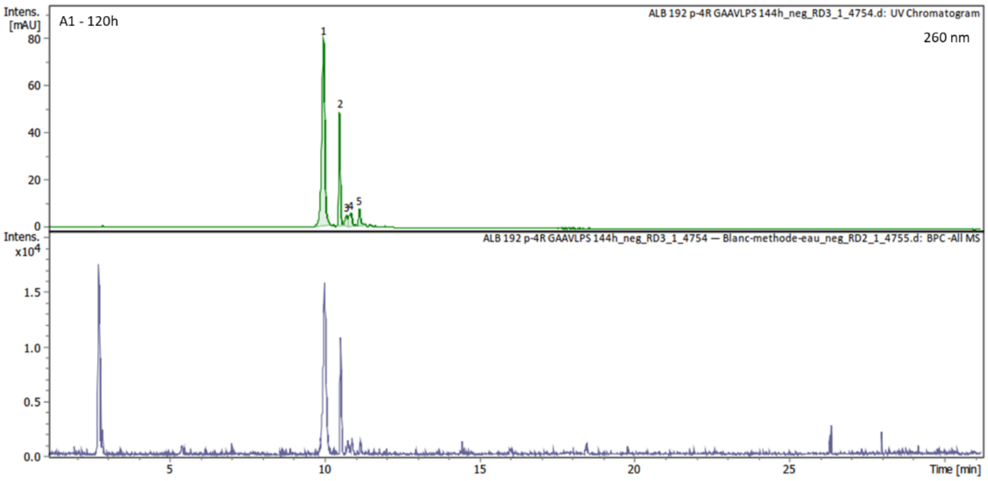

- Composé 1 - Tr = 10.0 min

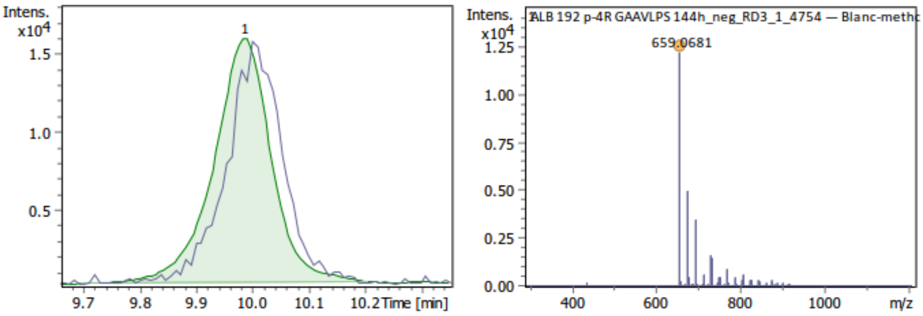

Meas. m/z	Ion Formula	Sum Formula	m/z	err [ppm]	mSigma	Adduct	z
659.0681	C38H46N14O31P4	C38H48N14O31P4	659.0707	4	14.6	M-H	2-

→ Le spectre de masse met en évidence la présence des adduits correspondant à un produit dont la formule brute est la suivante : $\mathbf{C_{38}H_{48}N_{14}O_{31}P_4}$.

pUGGU

- Composé 2 - Tr = 10.5 min

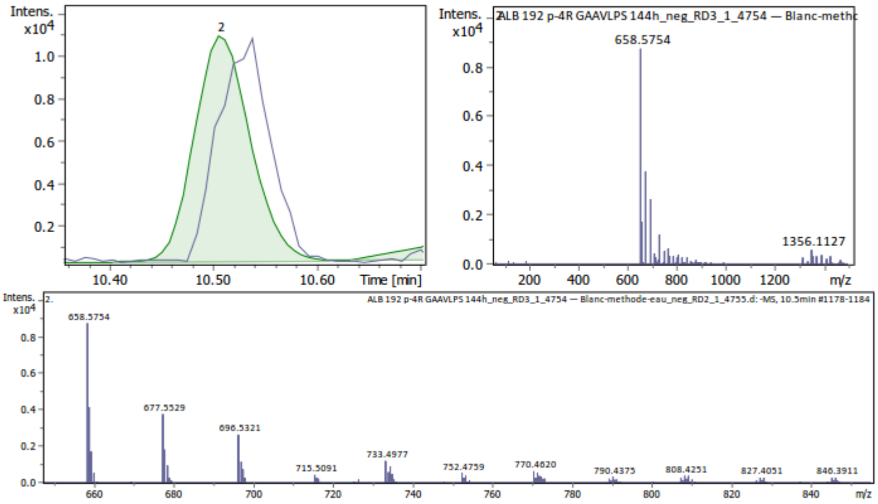

Soit M = 658.5754*2+2 = 1319.1508 Da

H_2N-pUGGU

- Composé 3 - Tr = 10.7 min

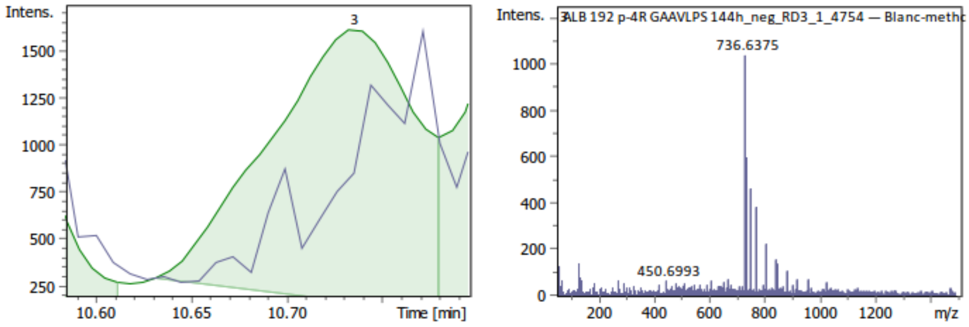

Soit M = 736.6375*2+2 = 1475.275 Da

EDC-pUGGU

- Composé 4 - Tr = 10.9 min

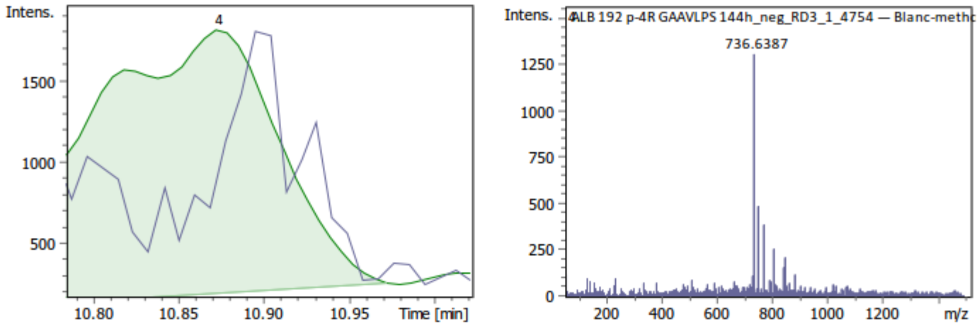

Soit M = 736.6387*2+2 = 1475.2774 Da

EDC-pUGGU

- Composé 5 - Tr = 11.1 min

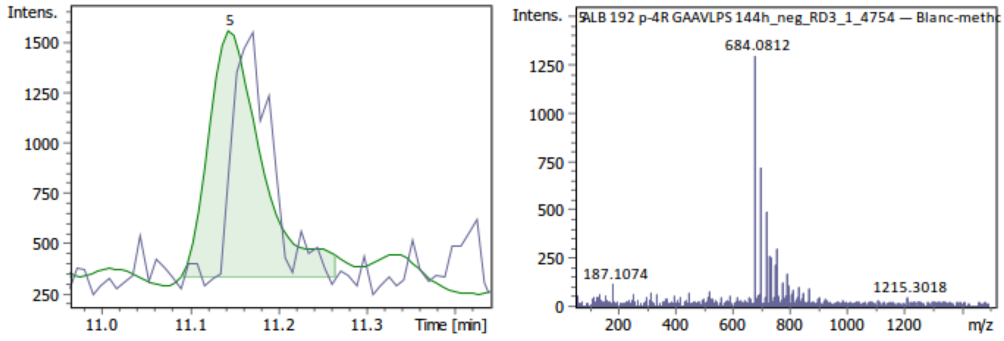

Soit M = 684.0812*2+2 = 1370.1624 Da

Im-pUGGU

Figure S19. UV (260 nm) and LC-MS chromatograms (negative ion mode) with UV peaks and associated compounds obtained for A1 experiment after 124 h of reaction with GAVLPSAA mixture.

A2 experiment – 360 h:

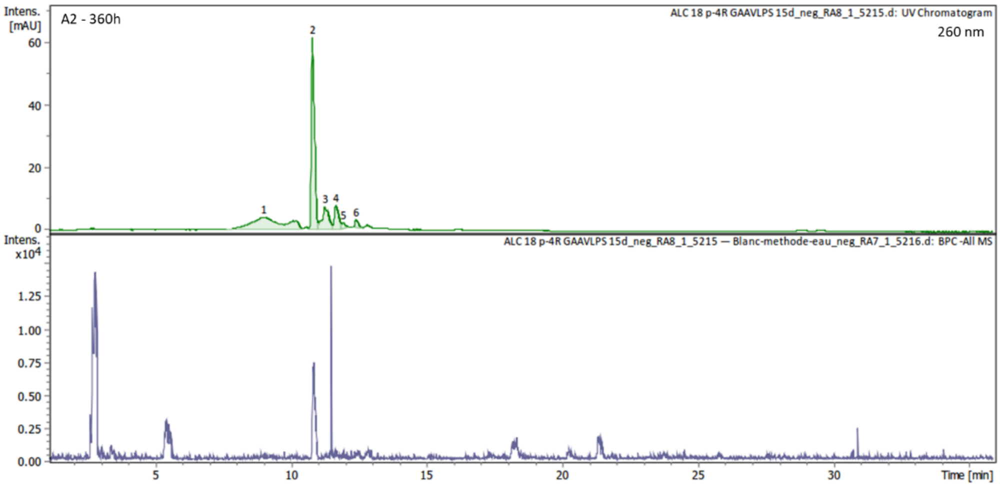

- Composé 1 - Tr = 9.0 min

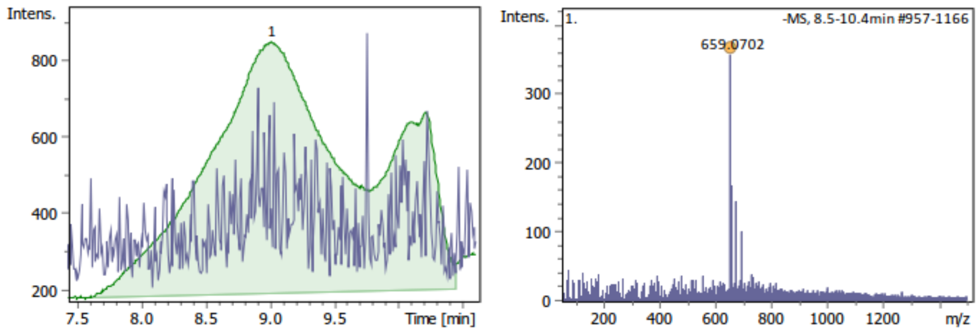

Meas. m/z	Ion Formula	Sum Formula	m/z	err [ppm]	mSigma	Adduct	z
659.0702	C38H46N14O31P4	C38H48N14O31P4	659.0707	0.8	32.1	M-H	2-

→ Le spectre de masse met en évidence la présence des adduits correspondant à un produit dont la formule brute est la suivante : $\mathbf{C_{38}H_{48}N_{14}O_{31}P_4}$.

pUGGU

- Composé 2 - Tr = 10.8 min

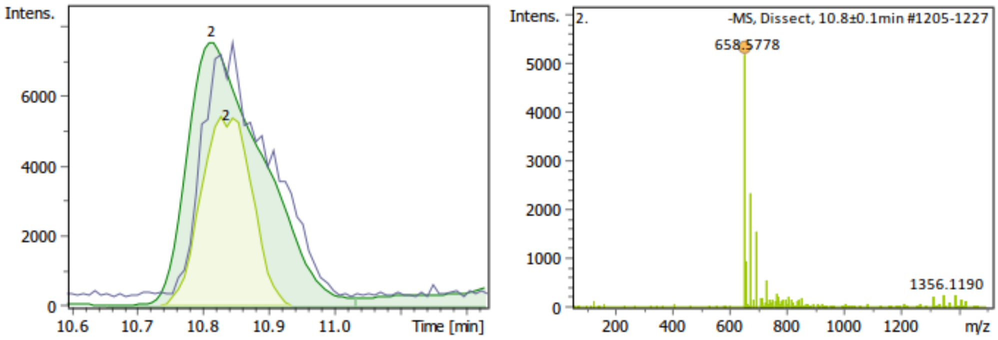

Meas. m/z	Ion Formula	Sum Formula	m/z	err [ppm]	mSigma	Adduct	z
658.5778	C38H47N15O30P4	C38H49N15O30P4	658.5787	1.5	15	M-H	2-

→ Le spectre de masse met en évidence la présence des adduits correspondant à un produit dont la formule brute est la suivante : $\mathbf{C_{38}H_{49}N_{15}O_{30}P_4}$.

H_2N-pUGGU

- Composé 3 - Tr = 11.3 min

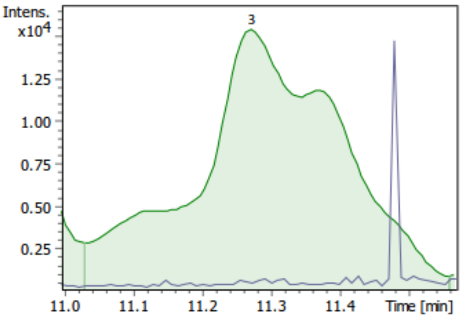

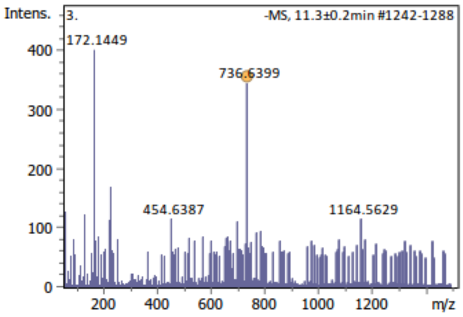

Meas. m/z	Ion Formula	Sum Formula	m/z	err [ppm]	mSigma	Adduct	z
736.6399	C46H63N17O31P4	C46H65N17O31P4	736.6419	2.6	140.1	M-H	2-

→ Le spectre de masse met en évidence la présence des adduits correspondant à un produit dont la formule brute est la suivante : $\mathbf{C_{46}H_{65}N_{17}O_{31}P_4}$.

EDC-pUGGU

- Composé 4 - Tr = 11.7 min

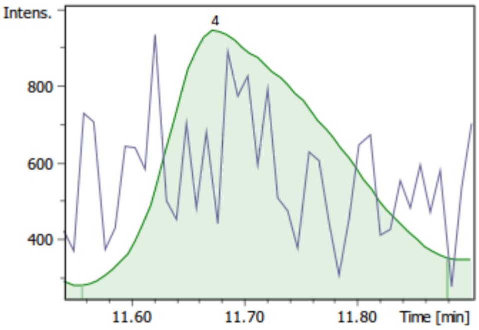

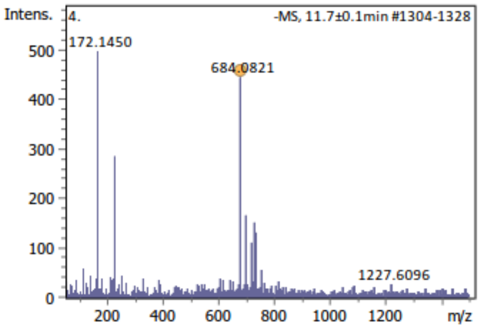

Meas. m/z	Ion Formula	Sum Formula	m/z	err [ppm]	mSigma	Adduct	z
684.0821	C41H48N16O30P4	C41H50N16O30P4	684.0842	3	177.7	M-H	2-

→ Le spectre de masse met en évidence la présence des adduits correspondant à un produit dont la formule brute est la suivante : $\mathbf{C_{41}H_{50}N_{16}O_{30}P_4}$.

Im-pUGGU

- Composé 5 - Tr = 12.0 min

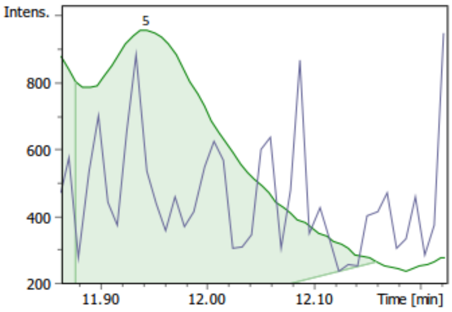

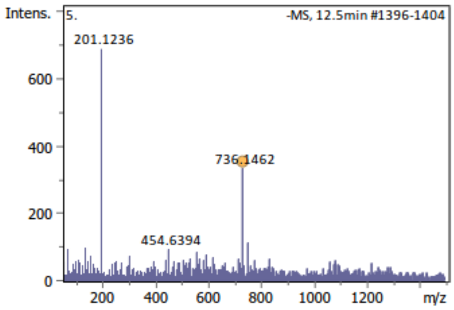

Meas. m/z	Ion Formula	Sum Formula	m/z	err [ppm]	mSigma	Adduct	z
736.1462	C46H64N18O30P4	C46H66N18O30P4	736.1499	5	67.7	M-H	2-

→ Le spectre de masse met en évidence la présence des adduits correspondant à un produit dont la formule brute est la suivante : $\mathbf{C_{46}H_{66}N_{18}O_{30}P_4}$.

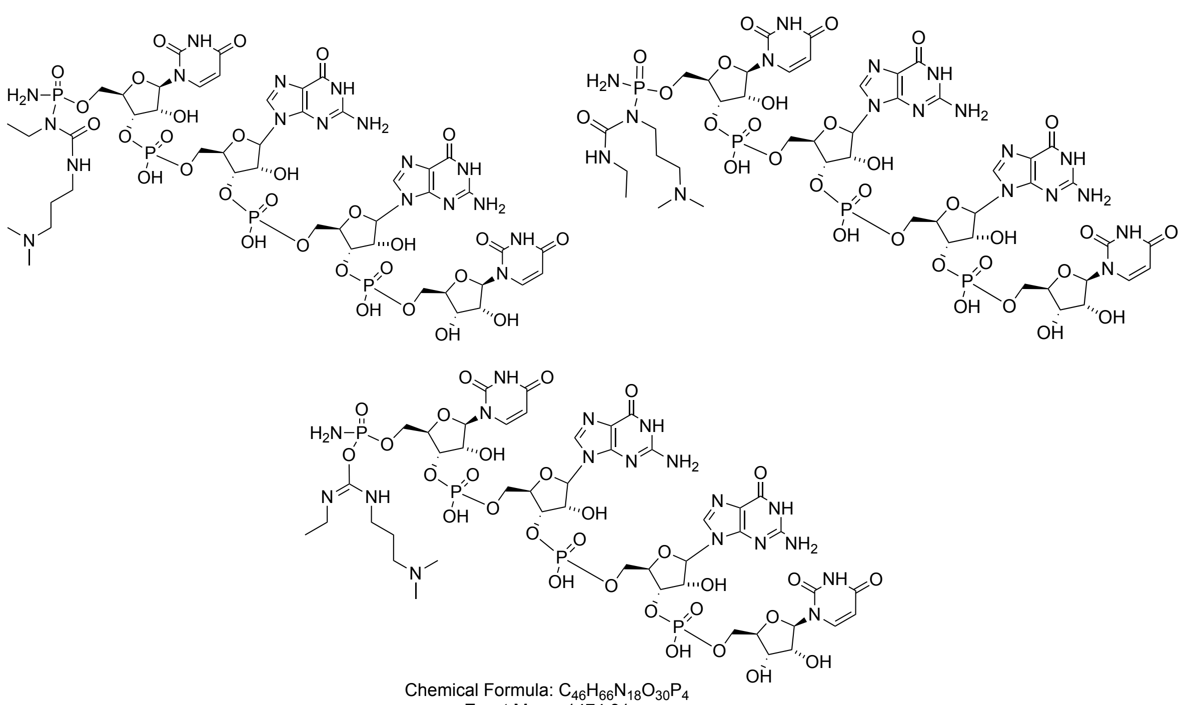

Chemical Formula: $C_{46}H_{66}N_{18}O_{30}P_4$
Exact Mass: 1474,31

EDC-H$_2$N-pUGGU

- Composé 6 - Tr = 12.5 min

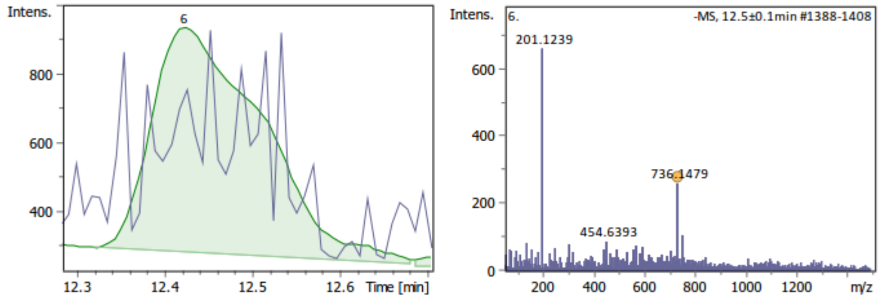

Meas. m/z	Ion Formula	Sum Formula	m/z	err [ppm]	mSigma	Adduct	z
736.1479	C46H64N18O30P4	C46H66N18O30P4	736.1499	2.6	72.2	M-H	2-

→ Le spectre de masse met en évidence la présence des adduits correspondant à un produit dont la formule brute est la suivante : **$C_{46}H_{66}N_{18}O_{30}P_4$**.

EDC-H$_2$N-pUGGU

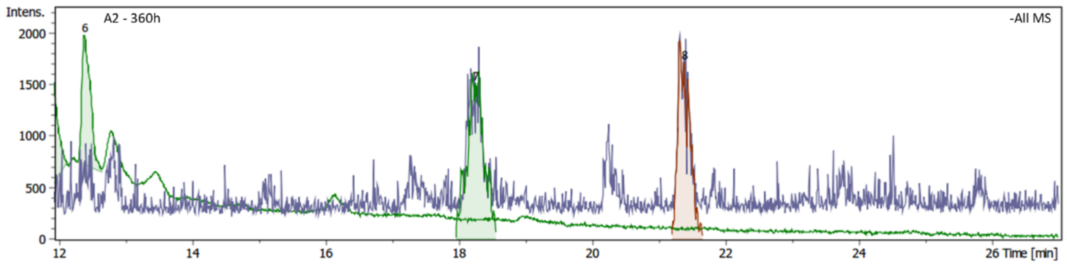

Pic 7

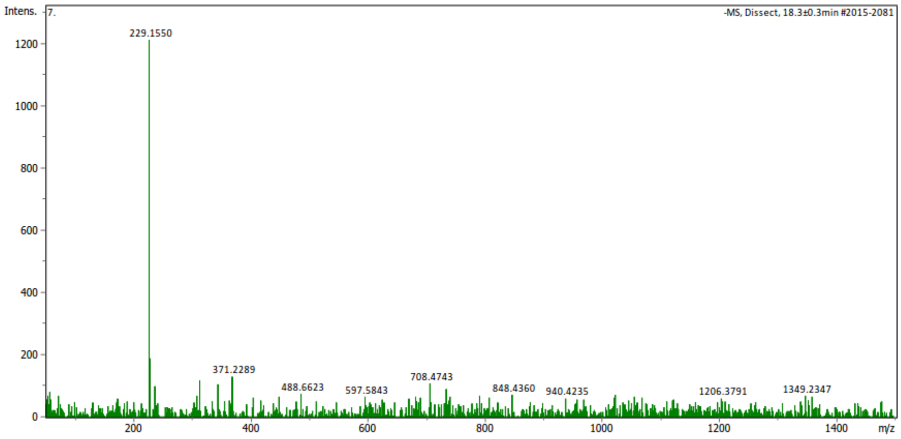

Azidoalaninyl-valine or valinyl-azidoalanine

Pic 8

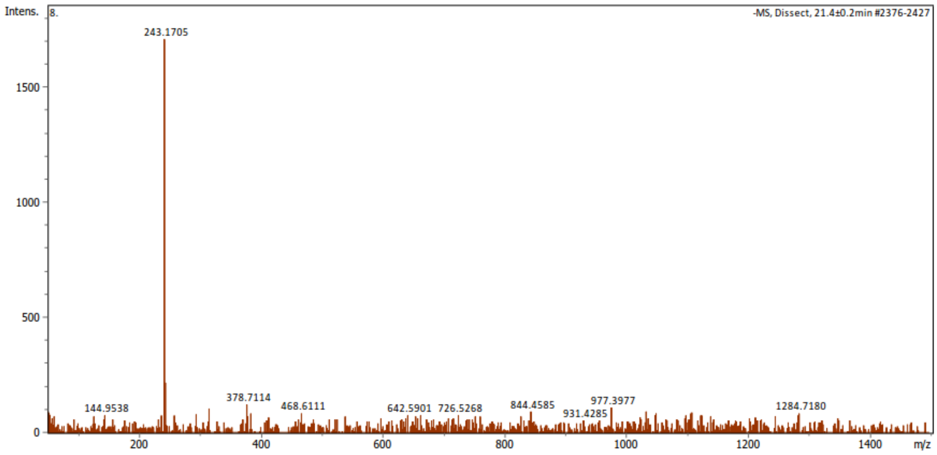

Azidoalaninyl-leucine or leucinyl-azidoalanine

Figure S20. UV (260 nm) and LC-MS chromatograms (negative ion mode) with UV peaks and associated compounds obtained for A2 experiment after 360 h of reaction with GAVLPSAA mixture.

In order to see if our conditions were compatible with the presence of prebiotic lipidic vesicles, their formation was tested by gentle hydration from dried lipid films. For that, 0.5 mL of a methanolic concentrated mixture of lipids containing 10 mM LA:MLG:DLPA (1:1:1) (6.66 mg of LA, 10.9 mg of MLG and 17.1 mg of DLPA in 10 mL of methanol) was diluted in a flask with 0.5 mL of MeOH (5 mM final concentration + 0.02 mol% of DOPE-Rhodamine). The solvent was evaporated using a rotavapor and a bath at 40°C to form a thin lipidic film. After dehydration overnight, the film was rehydrated overnight at room temperature with 1 mL of a buffer solution containing 1 or 2 mM EDC (A1 or A2 experiment) and 375 µM Et-Im at pH 6.5 (adjusted with 100 mM HCl) prepared as previously for the coupling experiments. After hydration over 16 h at room temperature, the vesicles were observed using epifluorescence microscopy. Then, 1 or 3 mM of GAVLPSAA mixture (A1 or A2 experiment) were added from a concentrated solution of 50 mM prepared as described previously. These concentrations were only estimation, as detailed thereover. For the A2 experiment, 1 mM EDC was also added at that point from a concentrated solution of 10 mM prepared as described previously. In both cases, the samples were adjusted to pH 8.0 adding 100 mM NaOH. After incubation over 16 h at room temperature, the vesicles were observed using epifluorescence microscopy.

3)3) Coupling attempts and vesicles production in the presence of EDC and DMAP

These experiments were performed very similarly to the previous ones:

Activation step: The previously synthesized RNA pUGGU (50 µM, 1 eq, concentration calculated with the UV absorbance at 260 nm and the online service OligoCalc™) was added to 20 equivalents of DMAP (1 mM, 1.22 mg in 1 mL of ultrapure water for a concentrated solution of 10 mM later diluted 10 times to reach the desired concentration) in ultrapure water. Then, the pH was adjusted to 8.0 adding 100 mM HCl. Afterwards, 20 equivalents of EDC (1 mM, B1 experiment) or 40 equivalents of EDC (2 mM, B2 experiment) were added from a concentrated solution (10 mM, 1.55 mg in 1 mL). Eventually, the pH was adjusted to 8.0 adding 100 mM NaOH. The activation reaction was left for 2 h (B1 experiment) or 192 h (B2 experiment).

Incubation step: After activation, 20 "equivalents" (1 mM, B1 experiment) or 40 "equivalents" (2 mM, B2 experiment) of the GAVLPSAA mixture were added from a concentrated solution (50 mM, 9.70 mg of lyophilized GAVLPSAA mixtures in 1 mL of ultrapure water) to the activated oligonucleotides. Importantly, the concentration of peptides added referred to an estimation. The amount of peptides in solution after polymerization with CDI could be estimated when taking into account the amount of salts, imidazole, non-polymerized amino acids (obtained with the oligomerization extent of all amino acids previously determined (Fig. 2F)) and the average molecular mass of the peptides (≈ 600 Da, Fig. 3). The pH was then adjusted to 8.0 adding 100 mM HCl. The reactions were then left in an Eppendorf vial for 120 h (B1 experiment) or 312 h (B2 experiment) at ambient temperature and without shaking. The pH was checked regularly and remained stable around the pH set after the addition of GAVLPSAA mixtures. 50 µL of the solutions were sampled during the reaction or at least the end of the incubation for LC-MS analysis.

After the incubation and 120 h additional hours, the sample of the B1.1 experiment was concentrated twice by heating at 60°C under argon flow. 50 µL of this sample were taken for LC-MS analysis. A second evaporation was performed similarly after 5 h and 50 µL were sampled for LC-MS analysis.

All the products of reaction were analysed using LC-MS with a *Gemini*™ C18 column (250 × 4.6 mm, 5 µm, *Phenomenex®*). The oligonucleotides were separated at 1.0 mL/min using a binary linear gradient from 2 to 44 % B in 28 min using eluants A) 5 mM NH_4OAc pH 8.0 and B) CH_3CN/A 95:5 (Figs. S21-S26).

B1 experiment – 0 h:

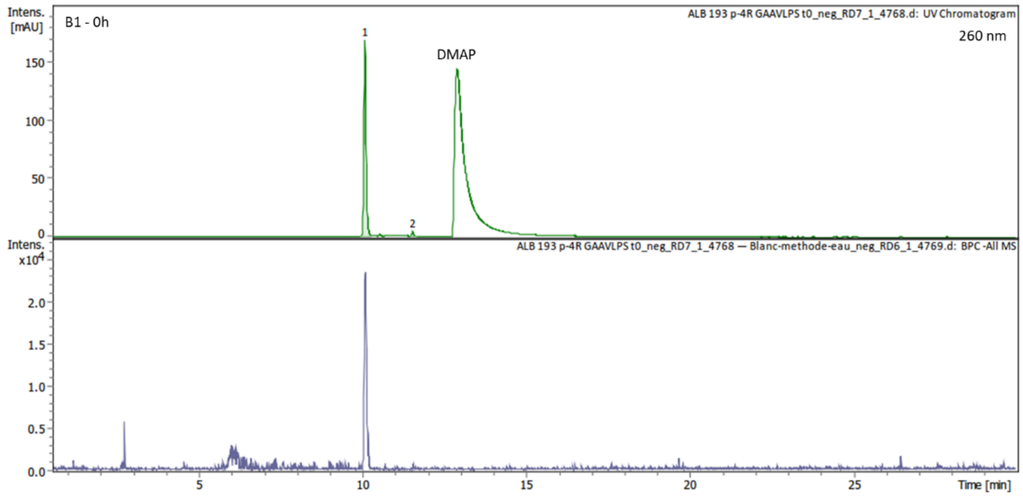

- Composé 1 - Tr = 10.1 min

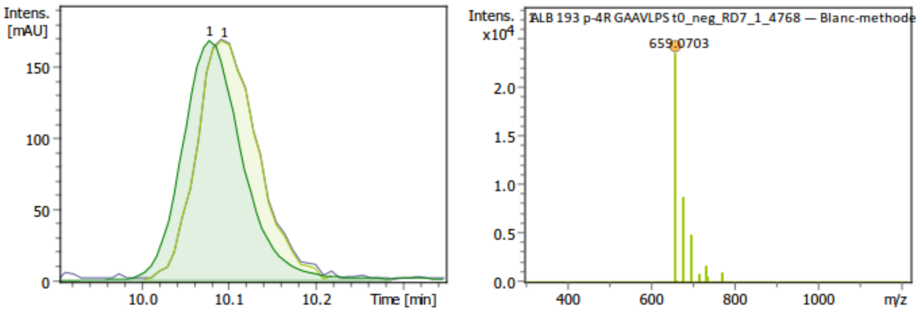

Meas. m/z	Ion Formula	Sum Formula	m/z	err [ppm]	mSigma	Adduct	z
659.0703	C38H46N14O31P4	C38H48N14O31P4	659.0707	0.6	12.8	M-H	2-

→ Le spectre de masse met en évidence la présence des adduits correspondant à un produit dont la formule brute est la suivante : $\mathbf{C_{38}H_{48}N_{14}O_{31}P_4}$.

pUGGU

- Composé 2 - Tr = 11.6 min

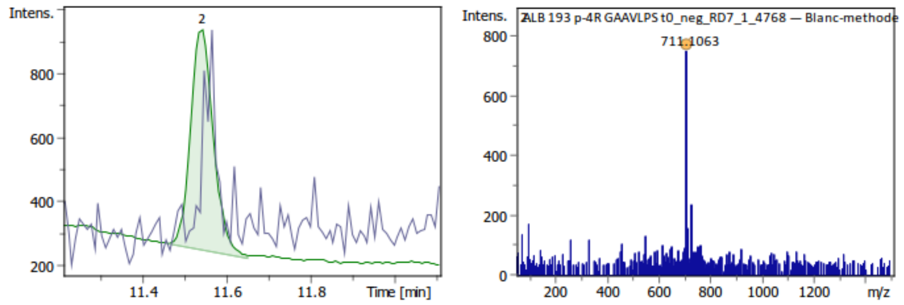

Meas. m/z	Ion Formula	Sum Formula	m/z	err [ppm]	mSigma	Adduct	z
711.1063	C45H54N16O30P4	C45H56N16O30P4	711.1077	2	156.9	M-H	2-

→ Le spectre de masse met en évidence la présence des adduits correspondant à un produit dont la formule brute est la suivante : $\mathbf{C_{45}H_{56}N_{16}O_{30}P_4}$.

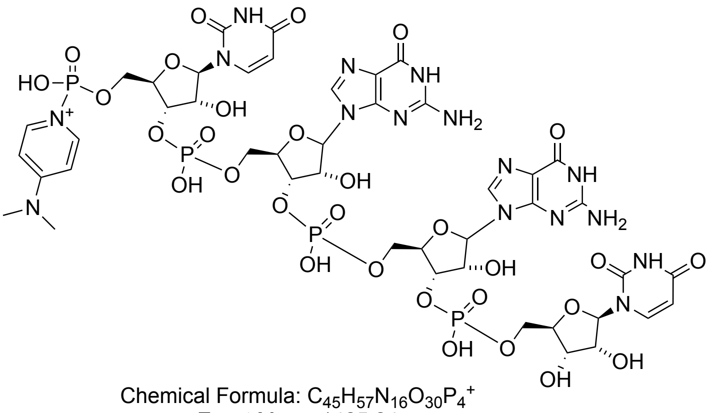

Chemical Formula: $C_{45}H_{57}N_{16}O_{30}P_4^+$
Exact Mass: 1425,24

DMAP-pUGGU

Figure S21. UV (260 nm) and LC-MS chromatograms (negative ion mode), UV peaks and associated compounds obtained for B1 experiment before the reaction with GAVLPSAA (0 h).

B1.1 experiment – 120 h:

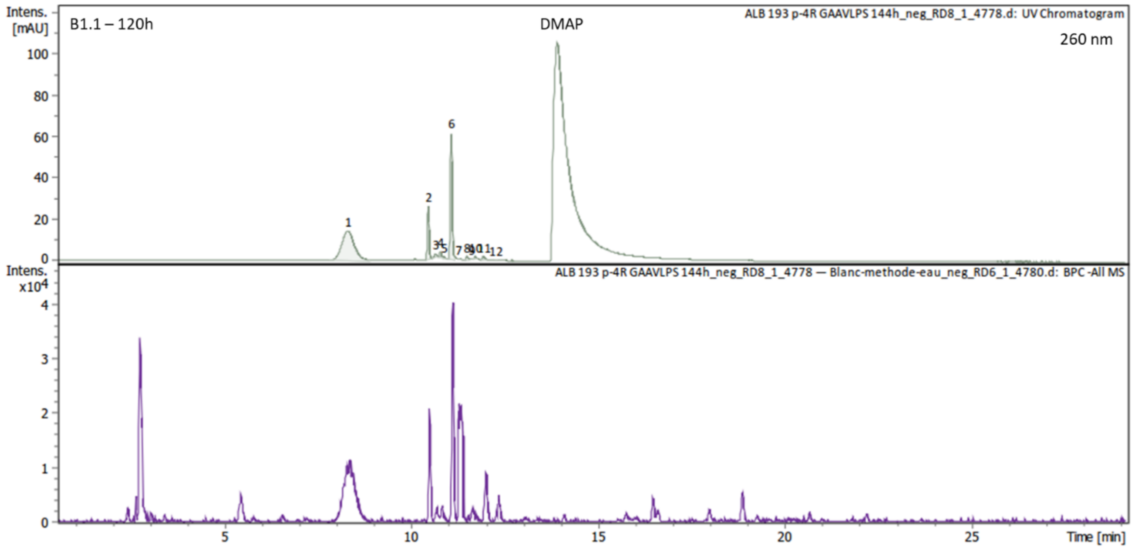

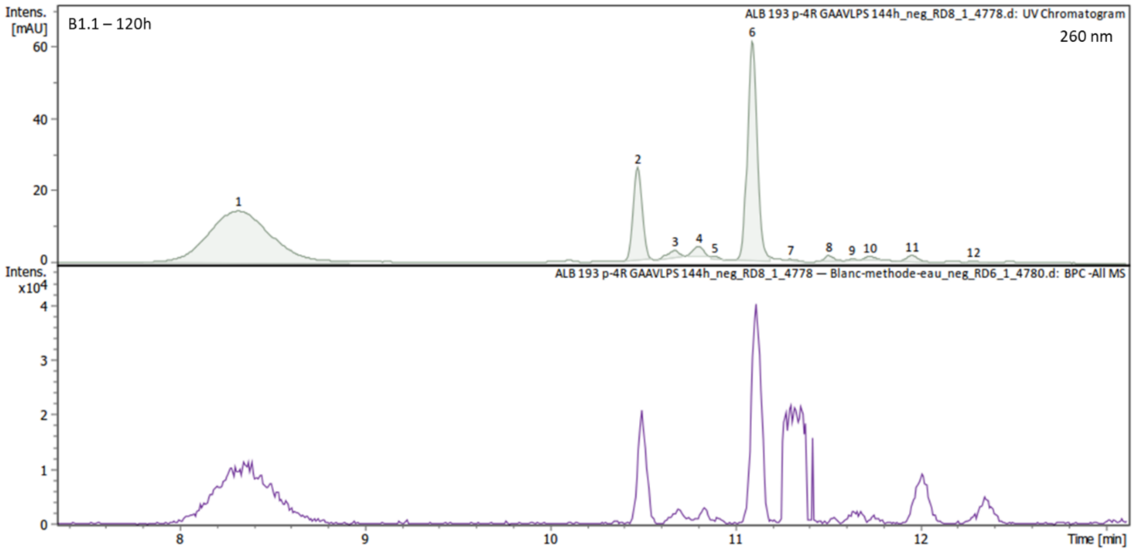

- Composé 1 - Tr = 8.3 min

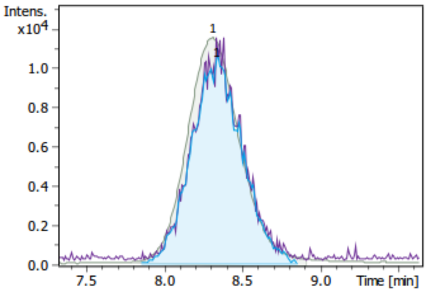

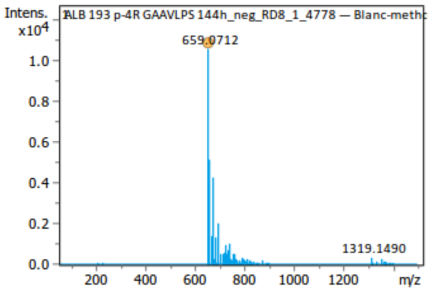

Meas. m/z	Ion Formula	Sum Formula	m/z	err [ppm]	mSigma	Adduct	z
659.0712	C38H46N14O31P4	C38H48N14O31P4	659.0707	-0.6	4.7	M-H	2-

→ Le spectre de masse met en évidence la présence des adduits correspondant à un produit dont la formule brute est la suivante : **$C_{38}H_{48}N_{14}O_{31}P_4$**

pUGGU

- Composé 2 - Tr = 10.5 min

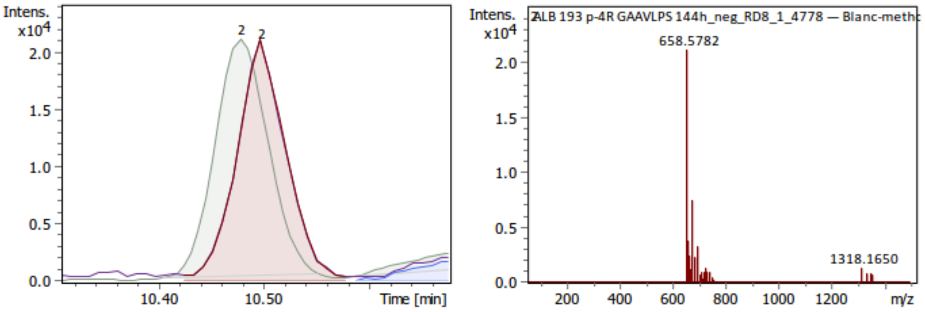

Meas. m/z	Ion Formula	Sum Formula	m/z	err [ppm]	mSigma	Adduct	z
658.5782	C38H47N15O30P4	C38H49N15O30P4	658.5787	0.8	4.8	M-H	2-

→ Le spectre de masse met en évidence la présence des adduits correspondant à un produit dont la formule brute est la suivante : $\mathbf{C_{38}H_{49}N_{15}O_{30}P_4}$

H$_2$N-pUGGU

- Composé 3 - Tr = 10.7 min

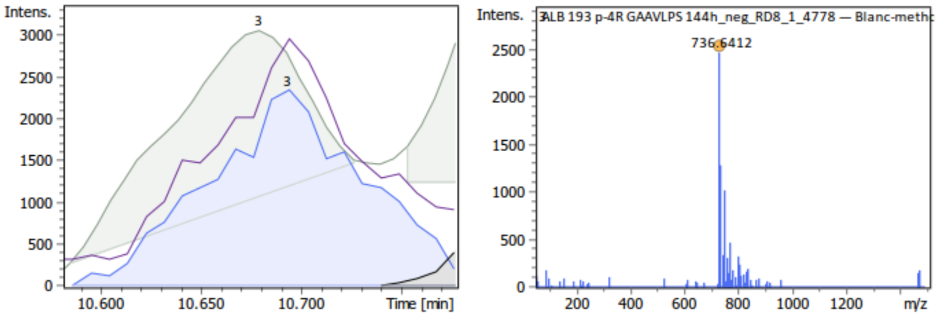

Meas. m/z	Ion Formula	Sum Formula	m/z	err [ppm]	mSigma	Adduct	z
736.6412	C46H63N17O31P4	C46H65N17O31P4	736.6419	0.9	33.8	M-H	2-

→ Le spectre de masse met en évidence la présence des adduits correspondant à un produit dont la formule brute est la suivante : $\mathbf{C_{46}H_{65}N_{17}O_{31}P_4}$

EDC-pUGGU

- Composé 4 - Tr = 10.8 min

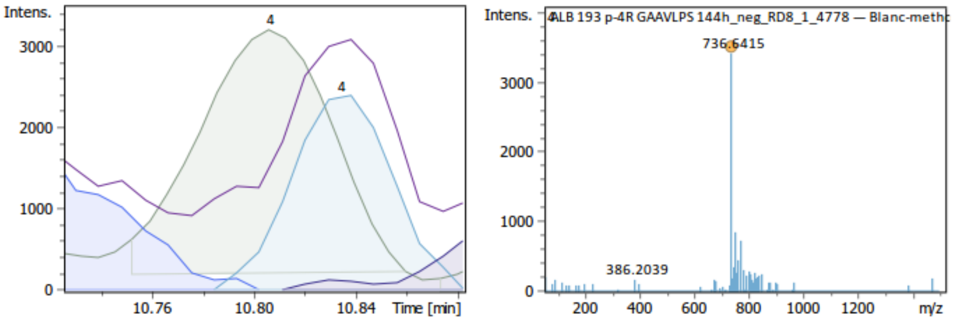

Meas. m/z	Ion Formula	Sum Formula	m/z	err [ppm]	mSigma	Adduct	z
736.6415	C46H63N17O31P4	C46H65N17O31P4	736.6419	0.5	28.3	M-H	2-

→ Le spectre de masse met en évidence la présence des adduits correspondant à un produit dont la formule brute est la suivante : $\mathbf{C_{46}H_{65}N_{17}O_{31}P_4}$

EDC-pUGGU

- Composé 5 - Tr = 10.9 min

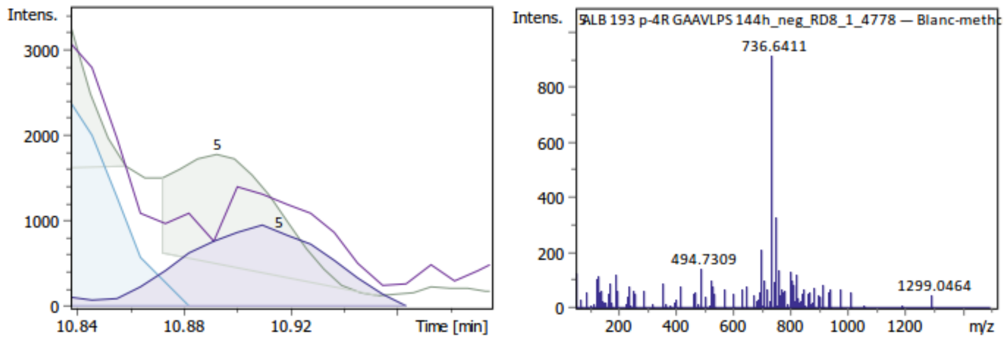

Meas. m/z	Ion Formula	Sum Formula	m/z	err [ppm]	mSigma	Adduct	z
736.6411	C46H63N17O31P4	C46H65N17O31P4	736.6419	1	51.9	M-H	2-

→ Le spectre de masse met en évidence la présence des adduits correspondant à un produit dont la formule brute est la suivante : **$C_{46}H_{65}N_{17}O_{31}P_4$**

EDC-pUGGU

- Composé 6 - Tr = 11.1 min

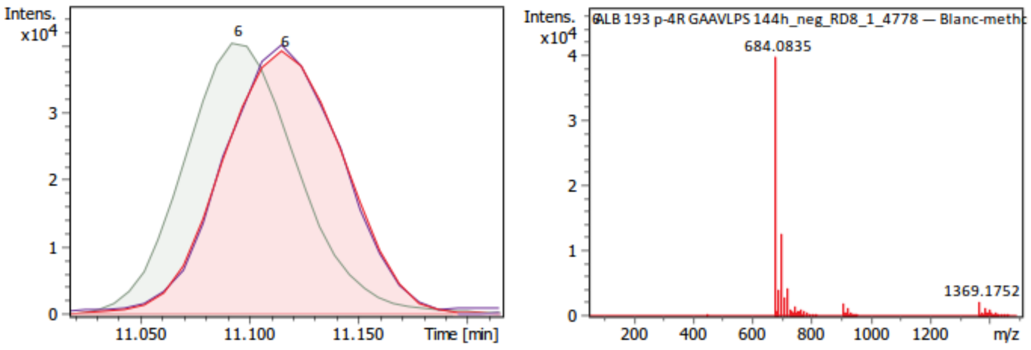

Meas. m/z	Ion Formula	Sum Formula	m/z	err [ppm]	mSigma	Adduct	z
684.0835	C42H44N20O26P4	C42H46N20O26P4	684.0849	2	7.4	M-H	2-
	C45H52N10O32P4	C45H54N10O32P4	684.0855	3	9.9	M-H	2-
	C41H48N16O30P4	C41H50N16O30P4	684.0842	1	13	M-H	2-
	C46H48N14O28P4	C46H50N14O28P4	684.0862	4	15.1	M-H	2-
	C40H52N12O34P4	C40H54N12O34P4	684.0835	0.1	22.6	M-H	2-
	C37H44N22O28P4	C37H46N22O28P4	684.0828	-0.9	23.6	M-H	2-
	C36H48N18O32P4	C36H50N18O32P4	684.0822	-1.9	33.6	M-H	2-
	C33H40N28O26P4	C33H42N28O26P4	684.0815	-2.9	35.4	M-H	2-
	C35H52N14O36P4	C35H54N14O36P4	684.0815	-2.9	43.8	M-H	2-
	C32H44N24O30P4	C32H46N24O30P4	684.0808	-3.9	45.2	M-H	2-
	C31H44N26O29P4	C31H46N26O29P4	684.0864	4.4	47.5	M-H	2-
	C34H56N10O40P4	C34H58N10O40P4	684.0808	-3.8	54.1	M-H	2-
	C31H48N20O34P4	C31H50N20O34P4	684.0802	-4.8	55.2	M-H	2-
	C33H56N12O39P4	C33H58N12O39P4	684.0865	4.4	56.2	M-H	2-
	C30H48N22O33P4	C30H50N22O33P4	684.0858	3.4	57.5	M-H	2-

→ Le spectre de masse met en évidence la présence des adduits correspondant à un produit dont la formule brute est la suivante : **Voir colonne « Sum Formula ».**

Im-pUGGU

- Composé 7 - Tr = 11.3 min

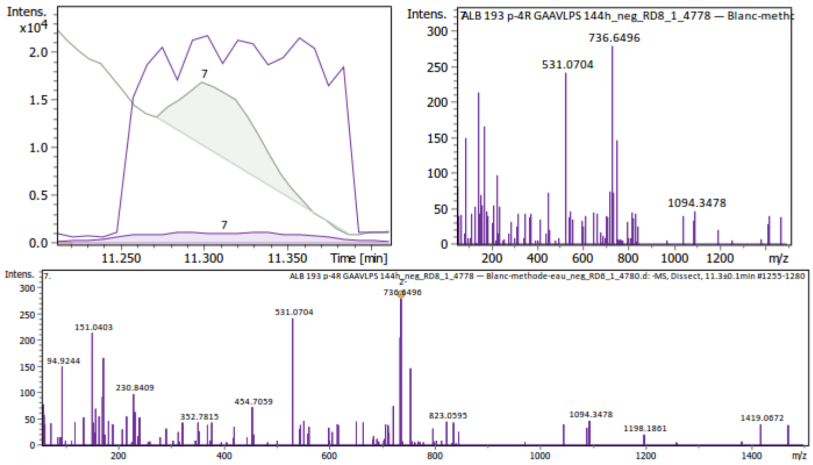

EDC-pUGGU

- Composé 8 - Tr = 11.5 min

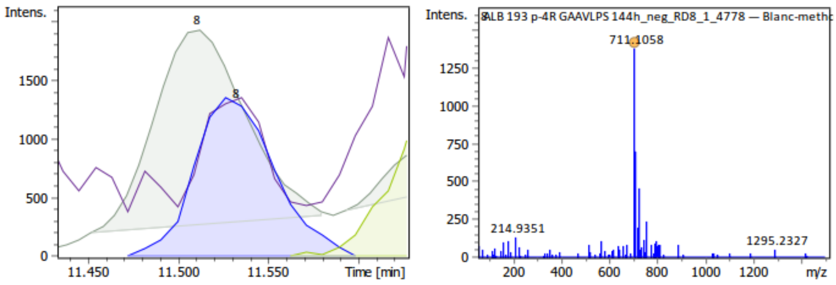

Meas. m/z	Ion Formula	Sum Formula	m/z	err [ppm]	mSigma	Adduct	z
711.1058	C45H54N16O30P4	C45H56N16O30P4	711.1077	2.6	33.9	M-H	2-

→ Le spectre de masse met en évidence la présence des adduits correspondant à un produit dont la formule brute est la suivante : $\mathbf{C_{45}H_{56}N_{16}O_{30}P_4}$

DMAP-pUGGU

- Composé 9 - Tr = 11.7 min

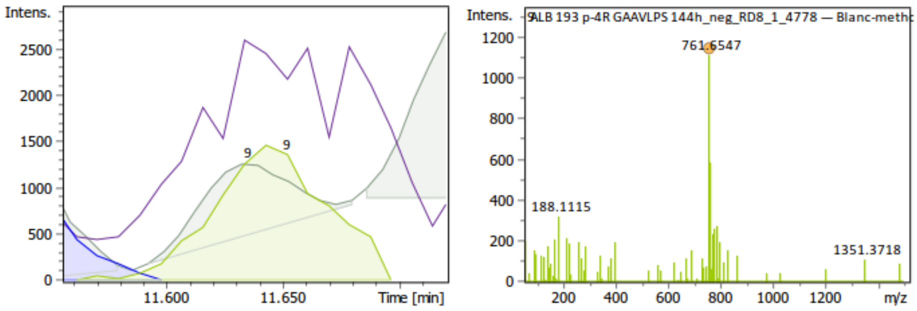

Meas. m/z	Ion Formula	Sum Formula	m/z	err [ppm]	mSigma	Adduct	z
761.6547	C35H57N35O27P4	C35H59N35O27P4	761.6562	2	17.8	M-H	2-
	C31H53N41O25P4	C31H55N41O25P4	761.6549	0.2	19.4	M-H	2-
	C34H61N31O31P4	C34H63N31O31P4	761.6556	1.1	20.6	M-H	2-
	C38H65N25O33P4	C38H67N25O33P4	761.6569	2.9	21.2	M-H	2-
	C39H65N23O34P4	C39H67N23O34P4	761.6513	-4.5	22.6	M-H	2-
	C37H69N21O37P4	C37H71N21O37P4	761.6562	2	23.4	M-H	2-
	C39H61N29O29P4	C39H63N29O29P4	761.6576	3.7	24.1	M-H	2-
	C41H73N15O39P4	C41H75N15O39P4	761.6576	3.7	25.7	M-H	2-
	C40H61N27O30P4	C40H63N27O30P4	761.652	-3.6	26.2	M-H	2-
	C30H57N37O29P4	C30H59N37O29P4	761.6542	-0.7	26.8	M-H	2-
	C40H77N11O43P4	C40H79N11O43P4	761.6569	2.9	27.2	M-H	2-
	C42H73N13O40P4	C42H75N13O40P4	761.652	-3.6	27.3	M-H	2-
	C33H65N27O35P4	C33H67N27O35P4	761.6549	0.2	27.5	M-H	2-
	C42H69N19O35P4	C42H71N19O35P4	761.6582	4.6	28.5	M-H	2-
	C36H73N17O41P4	C36H75N17O41P4	761.6556	1.1	29.4	M-H	2-
	C43H69N17O36P4	C43H71N17O36P4	761.6526	-2.8	30.7	M-H	2-
	C40H57N33O25P4	C40H59N33O25P4	761.6582	4.6	30.7	M-H	2-
	C41H57N31O26P4	C41H59N31O26P4	761.6526	-2.8	33.1	M-H	2-
	C32H69N23O39P4	C32H71N23O39P4	761.6542	-0.7	36.1	M-H	2-
	C44H65N21O32P4	C44H67N21O32P4	761.6533	-1.9	37	M-H	2-
	C35H77N13O45P4	C35H79N13O45P4	761.6549	0.2	37.5	M-H	2-
	C47H73N11O38P4	C47H75N11O38P4	761.654	-1	41.5	M-H	2-
	C45H61N25O28P4	C45H63N25O28P4	761.654	-1	45.1	M-H	2-
	C31H73N19O43P4	C31H75N19O43P4	761.6536	-1.5	45.5	M-H	2-
	C48H69N15O34P4	C48H71N15O34P4	761.6546	-0.1	49.1	M-H	2-
	C30H77N15O47P4	C30H79N15O47P4	761.6529	-2.4	55.3	M-H	2-
	C49H65N19O30P4	C49H67N19O30P4	761.6553	0.8	57.8	M-H	2-
	C50H61N23O26P4	C50H63N23O26P4	761.656	1.6	67.3	M-H	2-

→ Le spectre de masse met en évidence la présence des adduits correspondant à un produit dont la formule brute est la suivante : **Voir colonne « Sum Formula ».**

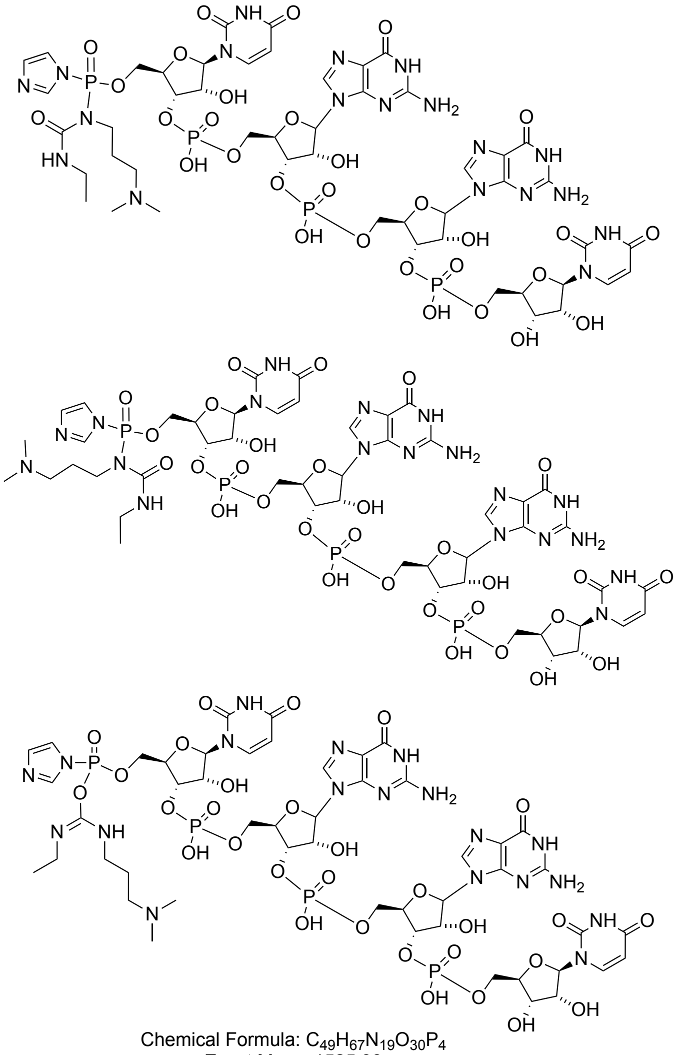

Chemical Formula: $C_{49}H_{67}N_{19}O_{30}P_4$
Exact Mass: 1525,33

EDC-Im-pUGGU

- Composé 10 - Tr = 11.8 min

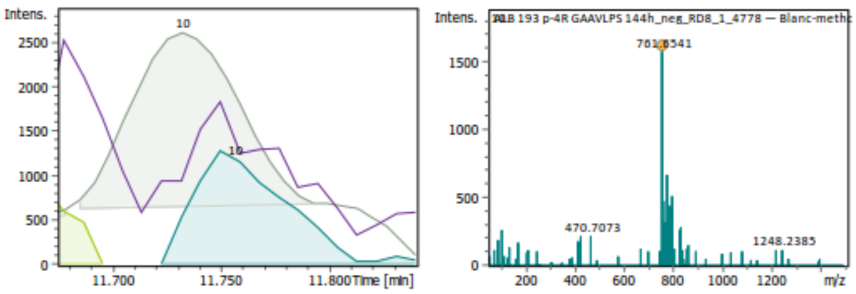

EDC-Im-pUGGU

- Composé 11 - Tr = 12.0 min

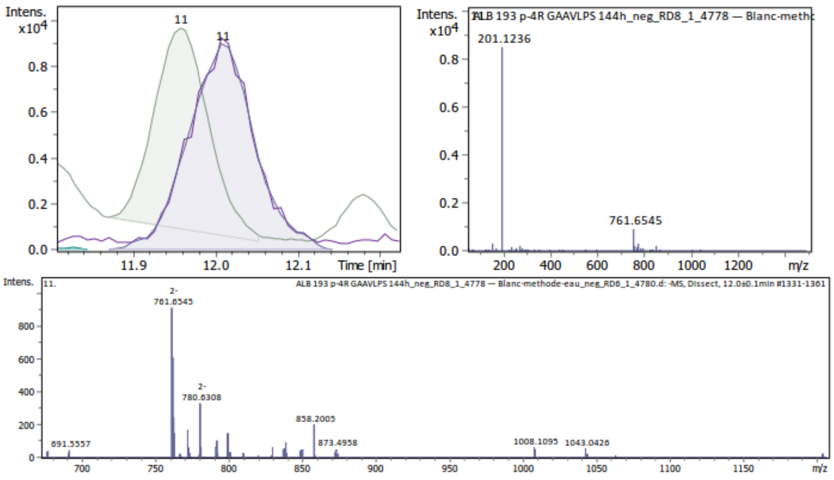

Unidentified derivative

- Composé 12 - Tr = 12.4 min

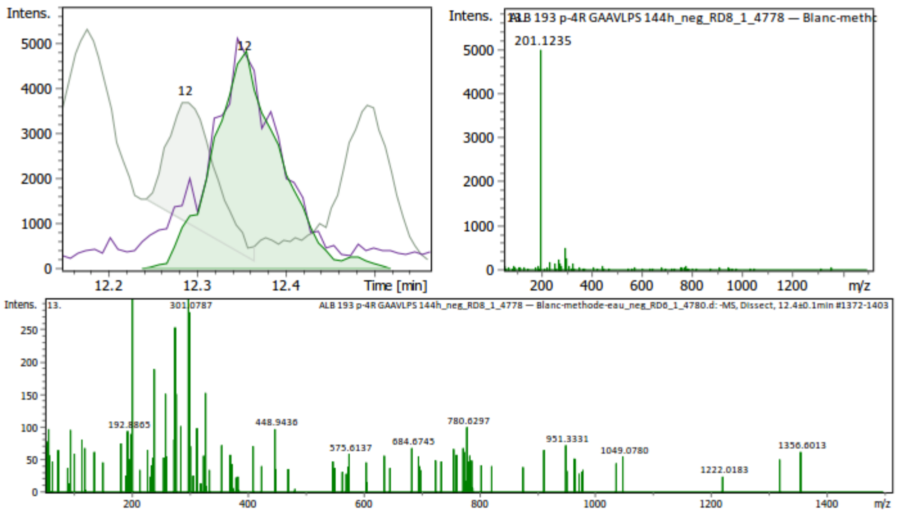

Unidentified derivative

Figure S22. UV (260 nm) and LC-MS chromatogram (negative ion mode), UV peaks and associated compounds obtained for B1 experiment after 120 h of reaction with GAVLPSAA (B1.1).

B1.2 experiment - 235h:

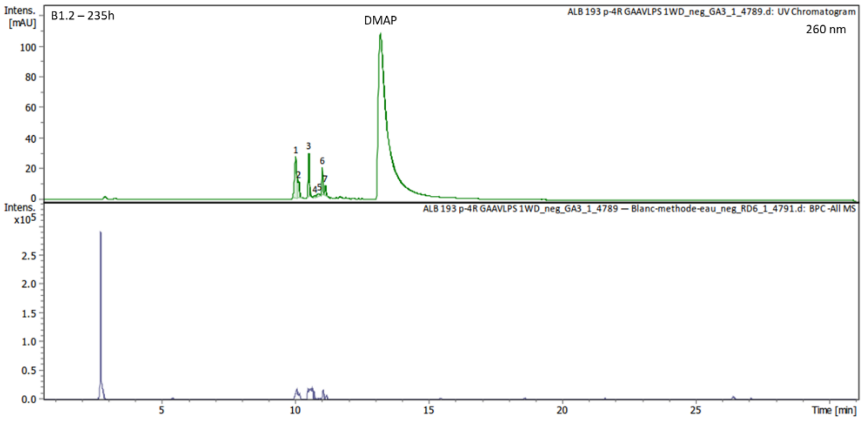

- Composé 1 - Tr = 10.0 min

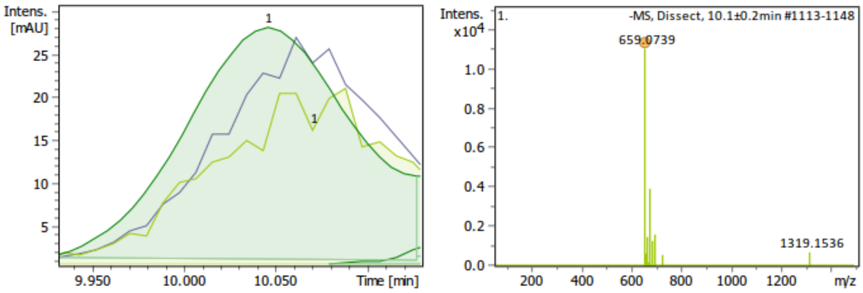

Meas. m/z	Ion Formula	Sum Formula	m/z	err [ppm]	mSigma	Adduct	z
659.0739	C38H46N14O31P4	C38H48N14O31P4	659.0707	-4.7	12.7	M-H	2-

→ Le spectre de masse met en évidence la présence des adduits correspondant à un produit dont la formule brute est la suivante : $\mathbf{C_{38}H_{48}N_{14}O_{31}P_4}$

pUGGU

- Composé 2 - Tr = 10.2 min

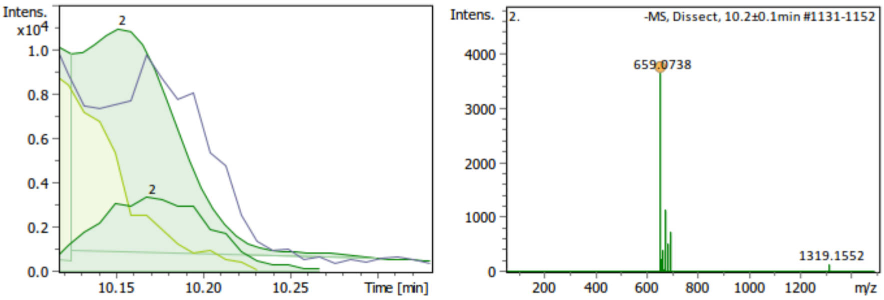

Meas. m/z	Ion Formula	Sum Formula	m/z	err [ppm]	mSigma	Adduct	z
659.0738	C38H46N14O31P4	C38H48N14O31P4	659.0707	-4.7	20.6	M-H	2-

→ Le spectre de masse met en évidence la présence des adduits correspondant à un produit dont la formule brute est la suivante : $\mathbf{C_{38}H_{48}N_{14}O_{31}P_4}$

pUGGU

- Composé 3 - Tr = 10.5 min

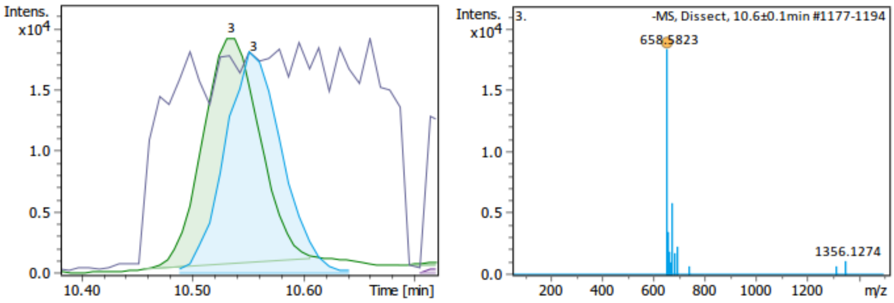

Meas. m/z	Ion Formula	Sum Formula	m/z	err [ppm]	mSigma	Adduct	z
658.5823	C38H47N15O30P4	C38H49N15O30P4	658.5787	-5.4	14.3	M-H	2-

→ Le spectre de masse met en évidence la présence des adduits correspondant à un produit dont la formule brute est la suivante : **$C_{38}H_{49}N_{15}O_{30}P_4$**

H_2N-pUGGU

- Composé 4 - Tr = 10.8 min

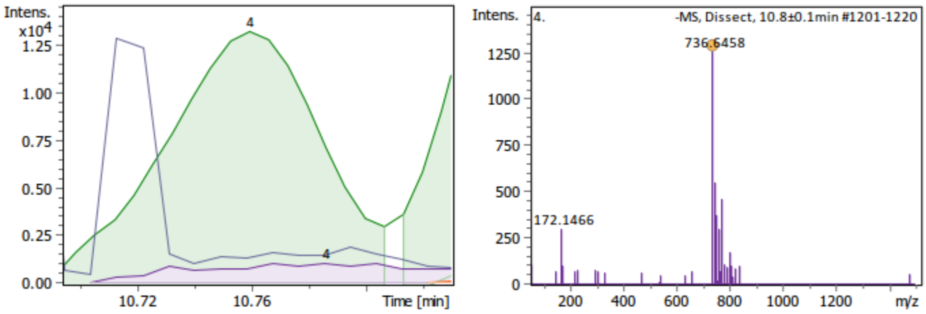

Meas. m/z	Ion Formula	Sum Formula	m/z	err [ppm]	mSigma	Adduct	z
736.6458	C46H63N17O31P4	C46H65N17O31P4	736.6419	-5.4	67.1	M-H	2-

→ Le spectre de masse met en évidence la présence des adduits correspondant à un produit dont la formule brute est la suivante : **$C_{46}H_{65}N_{17}O_{31}P_4$**

EDC-pUGGU

- Composé 5 - Tr = 10.9 min

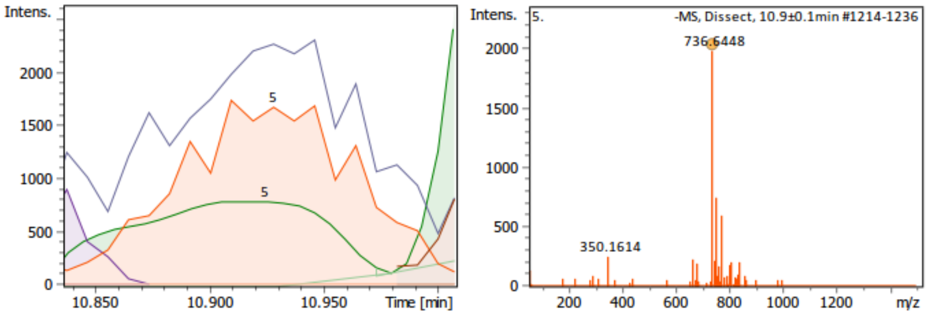

Meas. m/z	Ion Formula	Sum Formula	m/z	err [ppm]	mSigma	Adduct	z
736.6448	C46H63N17O31P4	C46H65N17O31P4	736.6419	-4	30	M-H	2-

→ Le spectre de masse met en évidence la présence des adduits correspondant à un produit dont la formule brute est la suivante : **$C_{46}H_{65}N_{17}O_{31}P_4$**

EDC-pUGGU

- Composé 6 - Tr = 11.0 min

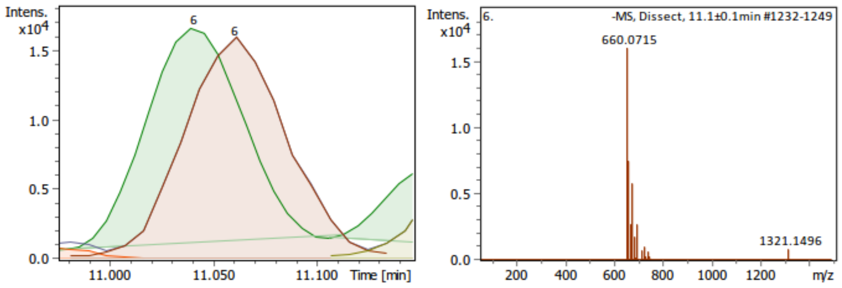

Soit M = 660.0715*2+2 = 1322.143 Da

Unidentified derivative

- Composé 7 - Tr = 11.2 min

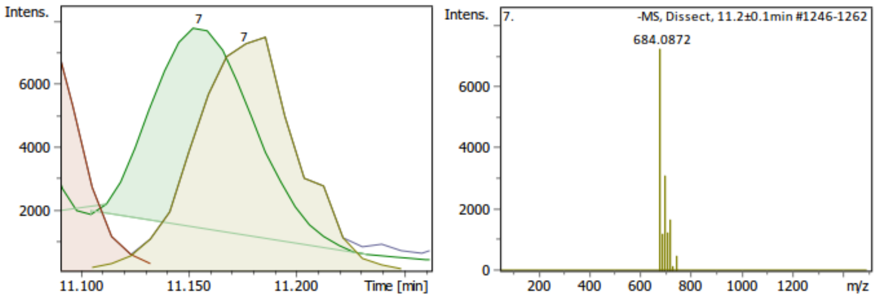

Meas. m/z	Ion Formula	Sum Formula	m/z	err [ppm]	mSigma	Adduct	z
684.0872	C41H48N16O30P4	C41H50N16O30P4	684.0842	-4.5	10.5	M-H	2-

→ Le spectre de masse met en évidence la présence des adduits correspondant à un produit dont la formule brute est la suivante : **$C_{41}H_{50}N_{16}O_{30}P_4$**

Im-pUGGU

Figure S23. UV (260 nm) and LC-MS chromatogram (negative ion mode), UV peaks and associated compounds obtained for B1 experiment after 240h of reaction with GAVLPSAA and 1 concentration (B1.2).

B1.3 experiment – 240 h:

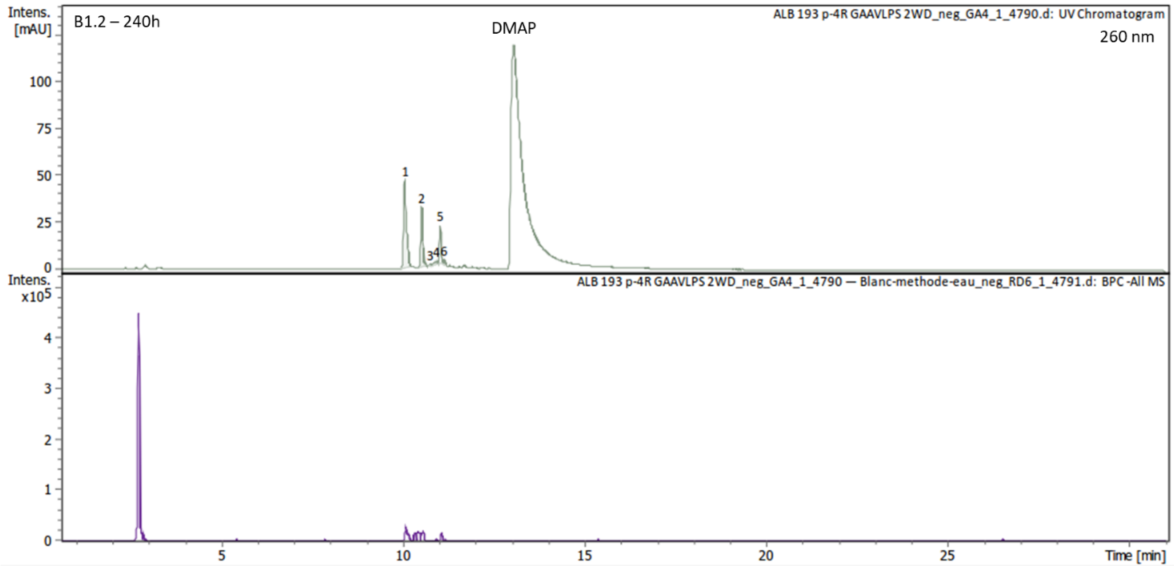

- Composé 1 - Tr = 10.0 min

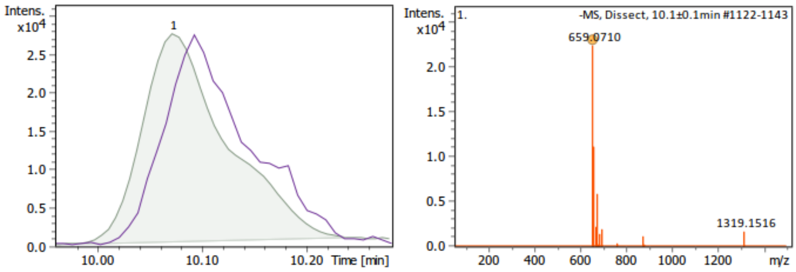

Meas. m/z	Ion Formula	Sum Formula	m/z	err [ppm]	mSigma	Adduct	z
659.071	C38H46N14O31P4	C38H48N14O31P4	659.0707	-0.4	8	M-H	2-

→ Le spectre de masse met en évidence la présence des adduits correspondant à un produit dont la formule brute est la suivante : $\mathbf{C_{38}H_{48}N_{14}O_{31}P_4}$

pUGGU

- Composé 2 - Tr = 10.6 min

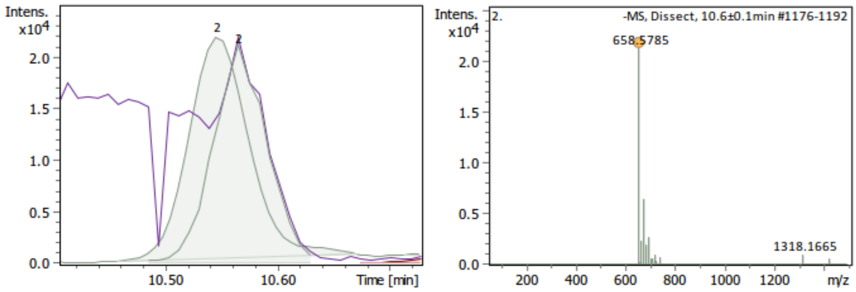

Meas. m/z	Ion Formula	Sum Formula	m/z	err [ppm]	mSigma	Adduct	z
658.5785	C38H47N15O30P4	C38H49N15O30P4	658.5787	0.4	18.2	M-H	2-

→ Le spectre de masse met en évidence la présence des adduits correspondant à un produit dont la formule brute est la suivante : $\mathbf{C_{38}H_{49}N_{15}O_{30}P_4}$

H_2N-pUGGU

- Composé 3 - Tr = 10.8 min

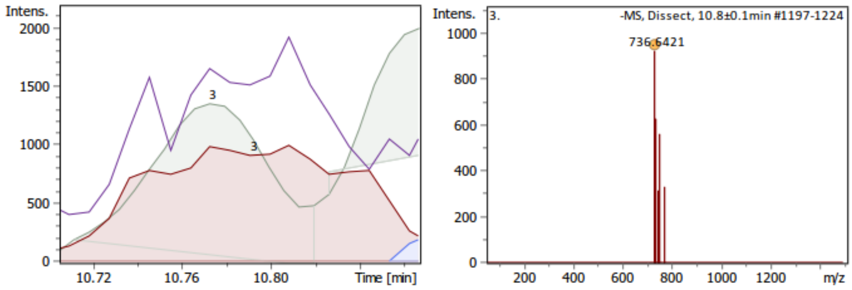

Meas. m/z	Ion Formula	Sum Formula	m/z	err [ppm]	mSigma	Adduct	z
736.6421	C46H63N17O31P4	C46H65N17O31P4	736.6419	-0.4	54.3	M-H	2-

→ Le spectre de masse met en évidence la présence des adduits correspondant à un produit dont la formule brute est la suivante : $\mathbf{C_{46}H_{65}N_{17}O_{31}P_4}$

EDC-pUGGU

- Composé 4 - Tr = 10.9 min

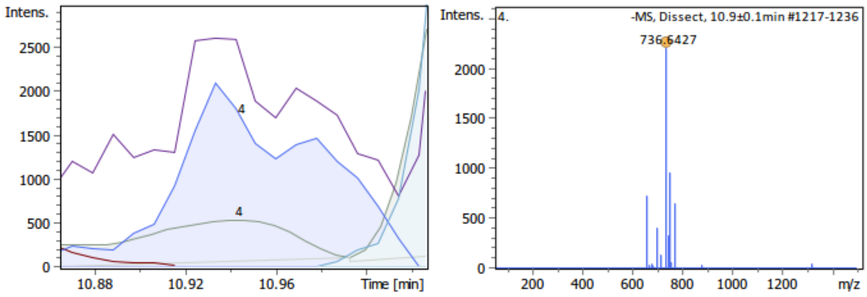

Meas. m/z	Ion Formula	Sum Formula	m/z	err [ppm]	mSigma	Adduct	z
736.6427	C46H63N17O31P4	C46H65N17O31P4	736.6419	-1.1	24.4	M-H	2-

→ Le spectre de masse met en évidence la présence des adduits correspondant à un produit dont la formule brute est la suivante : **$C_{46}H_{65}N_{17}O_{31}P_4$**

EDC-pUGGU

- Composé 5 - Tr = 11.1 min

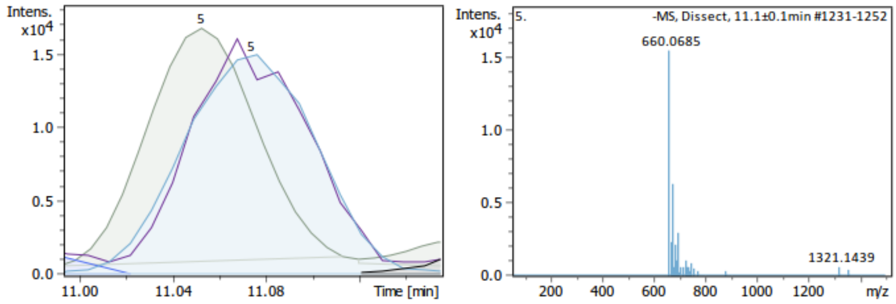

Soit M = 660.0685*2+2 = 1322.137 Da

Unidentified derivative

- Composé 6 - Tr = 11.2 min

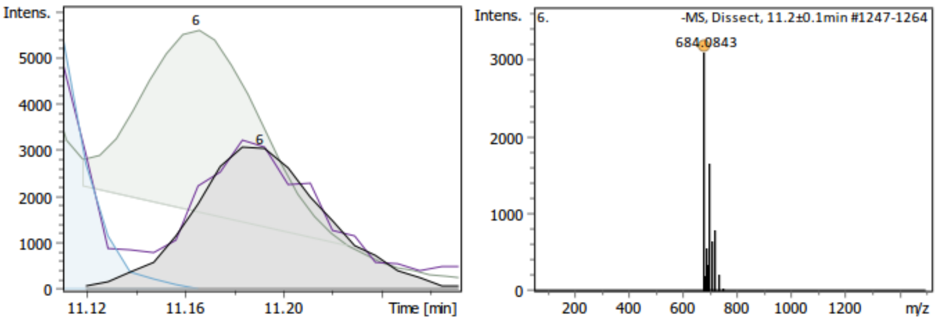

Meas. m/z	Ion Formula	Sum Formula	m/z	err [ppm]	mSigma	Adduct	z
684.0843	C41H48N16O30P4	C41H50N16O30P4	684.0842	-0.1	30	M-H	2-

→ Le spectre de masse met en évidence la présence des adduits correspondant à un produit dont la formule brute est la suivante : **$C_{41}H_{50}N_{16}O_{30}P_4$**

Im-pUGGU

Figure S24. UV (260 nm) and LC-MS chromatogram (negative ion mode), UV peaks and associated compounds obtained for B1 experiment after 245 h of reaction with GAVLPSAA and 2 concentrations (B1.3).

B2 experiment – 0 h:

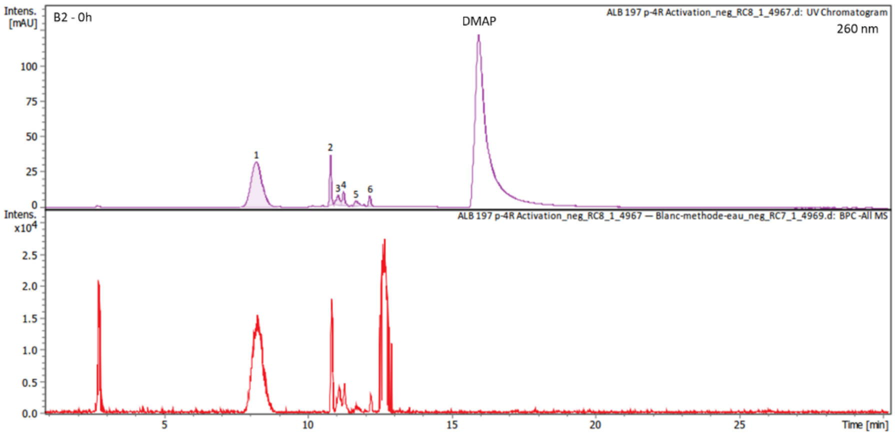

- Composé 1 - Tr = 8.3 min

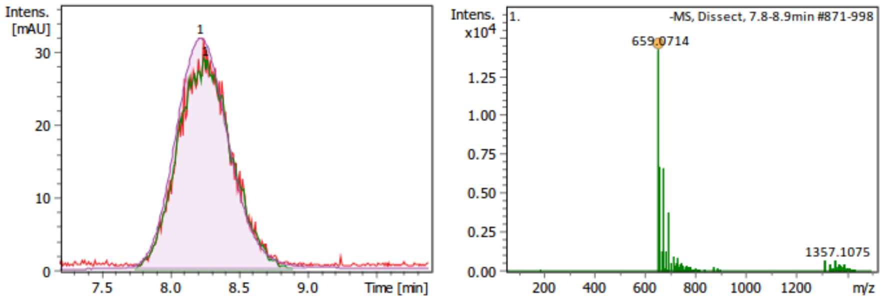

Meas. m/z	Ion Formula	Sum Formula	m/z	err [ppm]	mSigma	Adduct	z
659.0714	C38H46N14O31P4	C38H48N14O31P4	659.0707	-0.9	6	M-H	2-

→ Le spectre de masse met en évidence la présence des adduits correspondant à un produit dont la formule brute est la suivante : **$C_{38}H_{48}N_{14}O_{31}P_4$**.

pUGGU

- Composé 2 - Tr = 10.8 min

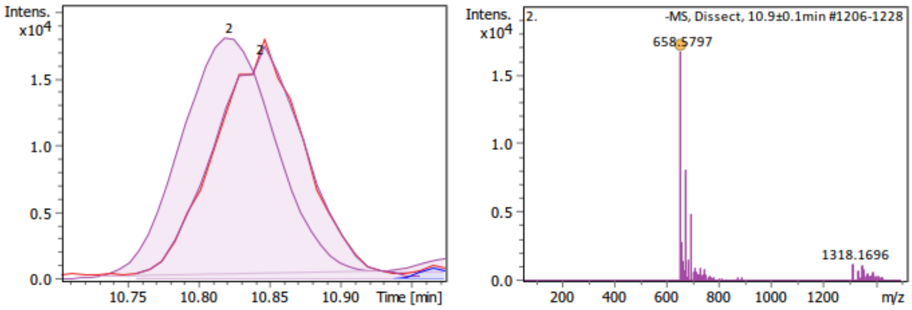

Meas. m/z	Ion Formula	Sum Formula	m/z	err [ppm]	mSigma	Adduct	z
658.5797	C38H47N15O30P4	C38H49N15O30P4	658.5787	-1.5	6.1	M-H	2-

→ Le spectre de masse met en évidence la présence des adduits correspondant à un produit dont la formule brute est la suivante : **$C_{38}H_{49}N_{15}O_{30}P_4$**.

H_2N-pUGGU

- Composé 3 - Tr = 11.1 min

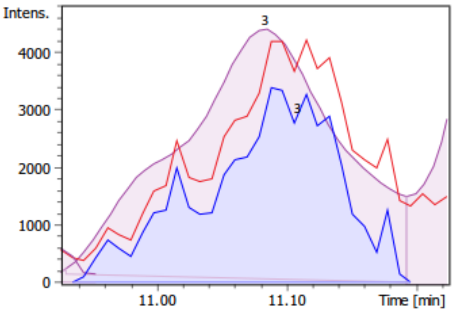

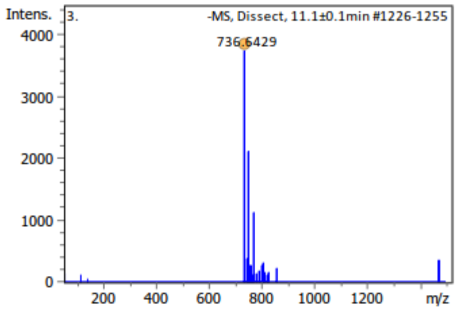

Meas. m/z	Ion Formula	Sum Formula	m/z	err [ppm]	mSigma	Adduct	z
736.6429	C46H63N17O31P4	C46H65N17O31P4	736.6419	-1.4	38.6	M-H	2-

→ Le spectre de masse met en évidence la présence des adduits correspondant à un produit dont la formule brute est la suivante : **$C_{46}H_{65}N_{17}O_{31}P_4$**.

EDC-pUGGU

- Composé 4 - Tr = 11.3 min

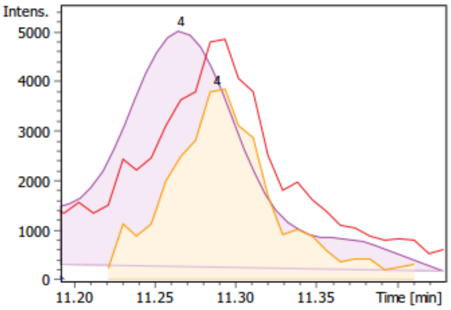

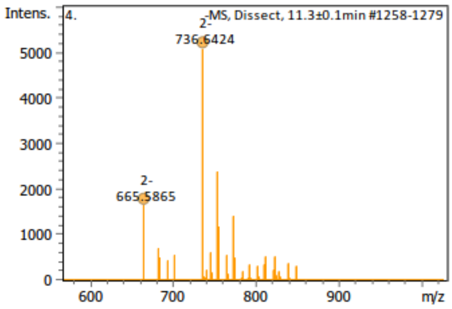

Meas. m/z	Ion Formula	Sum Formula	m/z	err [ppm]	mSigma	Adduct	z
665.5865	C46H45N15O25P4	C46H47N15O25P4	665.5836	-4.3	44.3	M-H	2-
	C40H45N19O26P4	C40H47N19O26P4	665.5872	1.1	45.6	M-H	2-
	C39H49N15O30P4	C39H51N15O30P4	665.5866	0.1	52.8	M-H	2-
736.6424	C46H63N17O31P4	C46H65N17O31P4	736.6419	-0.7	10.7	M-H	2-

→ Le spectre de masse met en évidence la présence des adduits correspondant à un produit dont la formule brute est la suivante : **Voir colonne « Sum Formula ».**

Major compound: EDC-pUGGU

- Composé 5 - Tr = 11.7 min

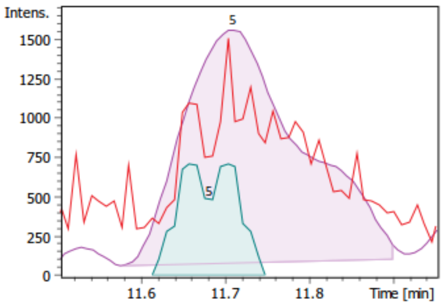

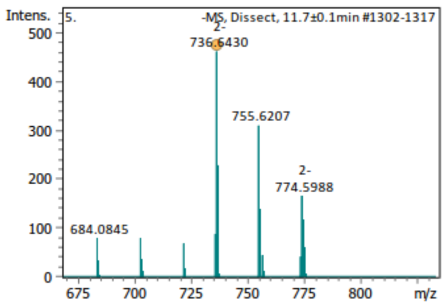

Meas. m/z	Ion Formula	Sum Formula	m/z	err [ppm]	mSigma	Adduct	z
684.0845	C41H48N16O30P4	C41H50N16O30P4	684.0842	-0.5	76	M-H	2-
736.643	C46H63N17O31P4	C46H65N17O31P4	736.6419	-1.5	50.7	M-H	2-

→ Le spectre de masse met en évidence la présence des adduits correspondant à un produit dont la formule brute est la suivante : **Voir colonne « Sum Formula ».**

Major compound: EDC-pUGGU

- Composé 6 - Tr = 12.2 min

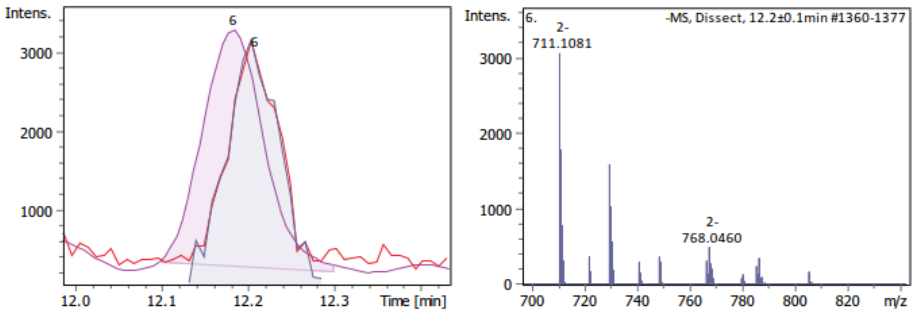

Meas. m/z	Ion Formula	Sum Formula	m/z	err [ppm]	mSigma	Adduct	z
711.1081	C46H50N20O26P4	C46H52N20O26P4	711.1083	0.4	24.2	M-H	2-
	C45H54N16O30P4	C45H56N16O30P4	711.1077	-0.6	26.6	M-H	2-
	C41H50N22O28P4	C41H52N22O28P4	711.1063	-2.5	37.3	M-H	2-
	C40H54N18O32P4	C40H56N18O32P4	711.1056	-3.4	44.3	M-H	2-
	C39H54N20O31P4	C39H56N20O31P4	711.1113	4.5	46.9	M-H	2-
	C38H58N16O35P4	C38H60N16O35P4	711.1106	3.6	55	M-H	2-

→ Le spectre de masse met en évidence la présence des adduits correspondant à un produit dont la formule brute est la suivante : **Voir colonne « Sum Formula ».**

Major compound: DMAP-pUGGU

Figure S25. UV (260 nm) and LC-MS chromatograms (negative ion mode), UV peaks and associated compounds obtained for C3 experiment before the reaction with GAVLPSAA (0 h).

B2 experiment – 312 h:

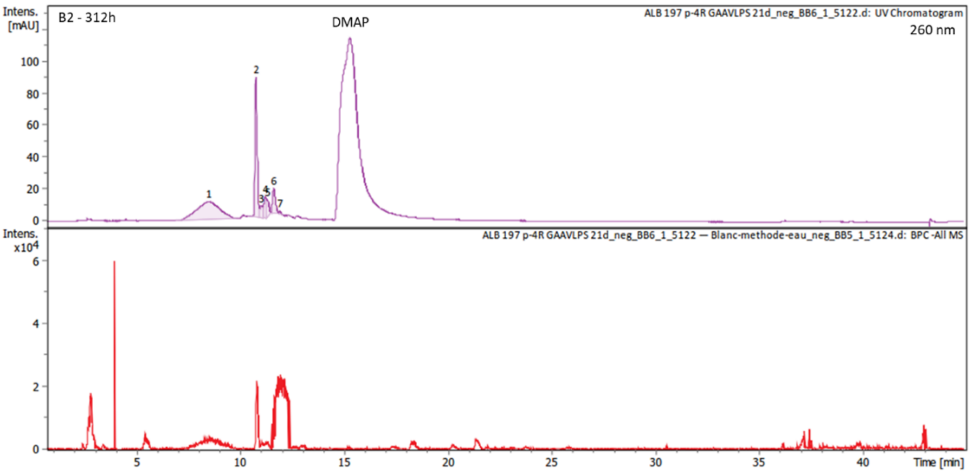

- Composé 1 - Tr = 8.5 min

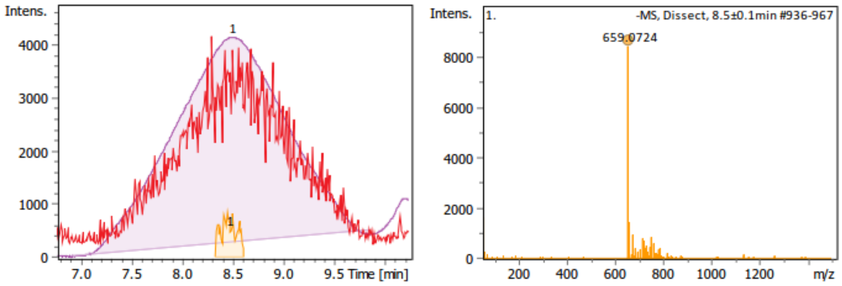

Meas. m/z	Ion Formula	Sum Formula	m/z	err [ppm]	mSigma	Adduct	z
659.0724	C38H46N14O31P4	C38H48N14O31P4	659.0707	-2.4	147.2	M-H	2-

→ Le spectre de masse met en évidence la présence des adduits correspondant à un produit dont la formule brute est la suivante : **$C_{38}H_{48}N_{14}O_{31}P_4$.**

pUGGU

- Composé 2 - Tr = 10.8 min

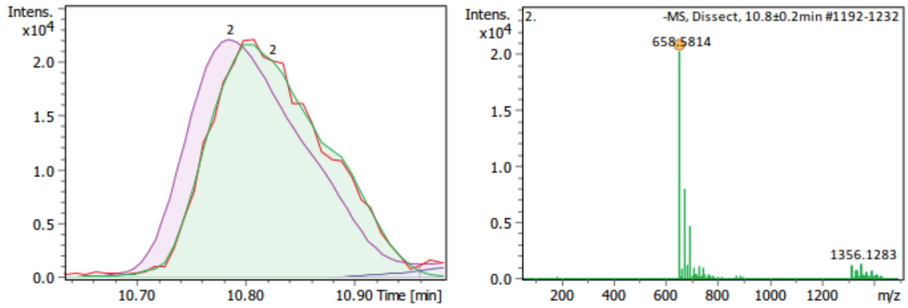

Meas. m/z	Ion Formula	Sum Formula	m/z	err [ppm]	mSigma	Adduct	z
658.5814	C38H47N15O30P4	C38H49N15O30P4	658.5787	-4.1	7.4	M-H	2-

→ Le spectre de masse met en évidence la présence des adduits correspondant à un produit dont la formule brute est la suivante : $\mathbf{C_{38}H_{49}N_{15}O_{30}P_4}$.

H_2N-pUGGU

- Composé 3 - Tr = 11.1 min

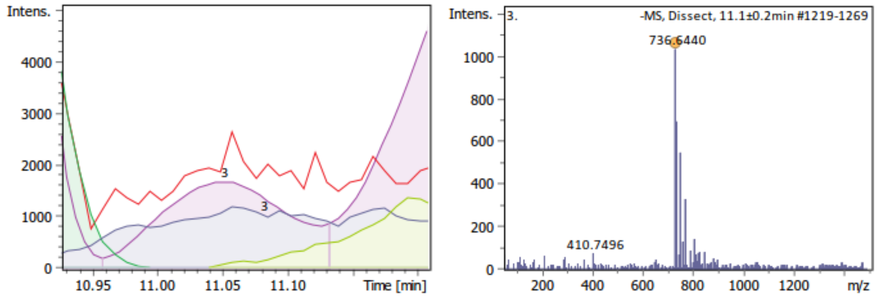

Meas. m/z	Ion Formula	Sum Formula	m/z	err [ppm]	mSigma	Adduct	z
736.644	C46H63N17O31P4	C46H65N17O31P4	736.6419	-2.9	49.6	M-H	2-

→ Le spectre de masse met en évidence la présence des adduits correspondant à un produit dont la formule brute est la suivante : $\mathbf{C_{46}H_{65}N_{17}O_{31}P_4}$.

EDC-pUGGU

- Composé 4 - Tr = 11.3 min

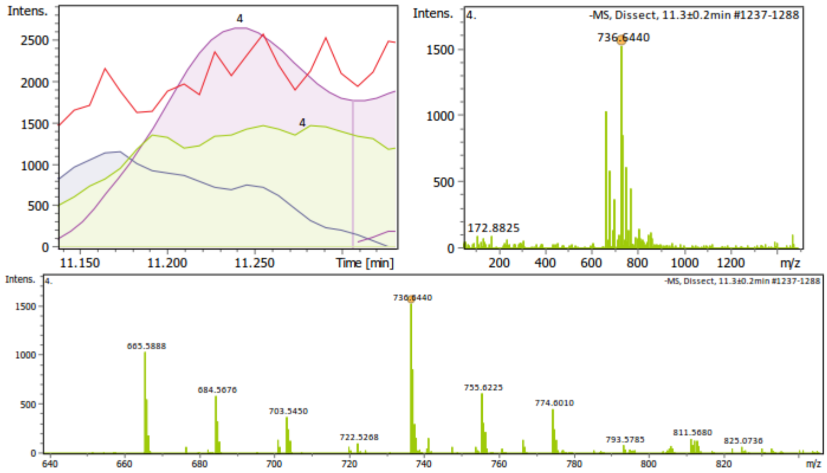

Meas. m/z	Ion Formula	Sum Formula	m/z	err [ppm]	mSigma	Adduct	z
736.644	C46H63N17O31P4	C46H65N17O31P4	736.6419	-2.9	22.7	M-H	2-

→ Le spectre de masse met en évidence la présence des adduits correspondant à un produit dont la formule brute est la suivante : $\mathbf{C_{46}H_{65}N_{17}O_{31}P_4}$.
Ainsi qu'une autre espèce, $[M\text{-}H]^-$ = 665.5888Da

Major compound: EDC-pUGGU

- Composé 5 - Tr = 11.4 min

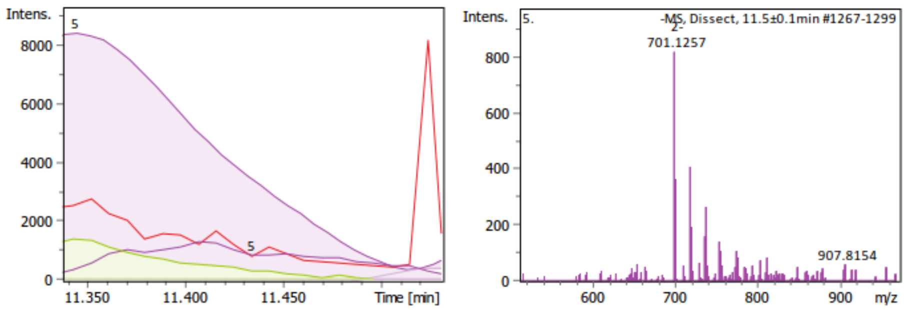

Unidentified derivative

- Composé 6 - Tr = 11.7 min

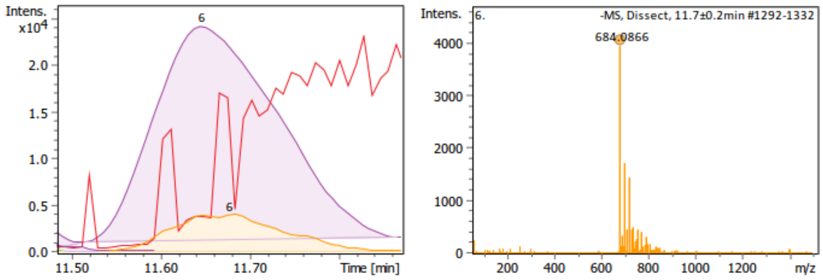

Meas. m/z	Ion Formula	Sum Formula	m/z	err [ppm]	mSigma	Adduct	z
684.0866	C41H48N16O30P4	C41H50N16O30P4	684.0842	-3.5	8.9	M-H	2-

→ Le spectre de masse met en évidence la présence des adduits correspondant à un produit dont la formule brute est la suivante : **$C_{41}H_{50}N_{16}O_{30}P_4$**.

Im-pUGGU

- Composé 7 - Tr = 12.1 min

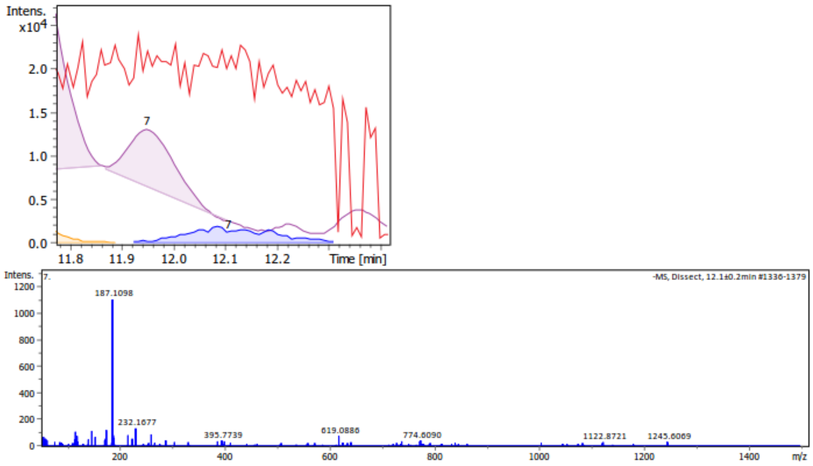

Unidentified derivative

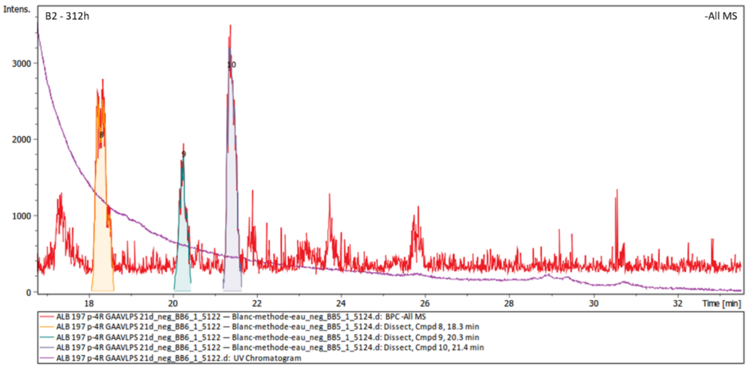

PIC 8

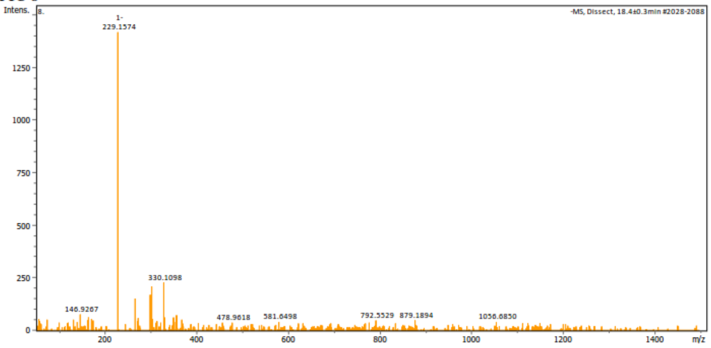

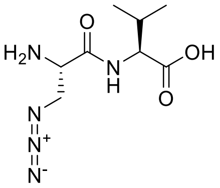

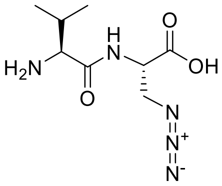

Chemical Formula: $C_8H_{15}N_5O_3$ Exact Mass: 229,12

Chemical Formula: $C_8H_{15}N_5O_3$ Exact Mass: 229,12

Azidoalaninyl-valine (left) or valinyl-azidoalanine (right)

PIC 9

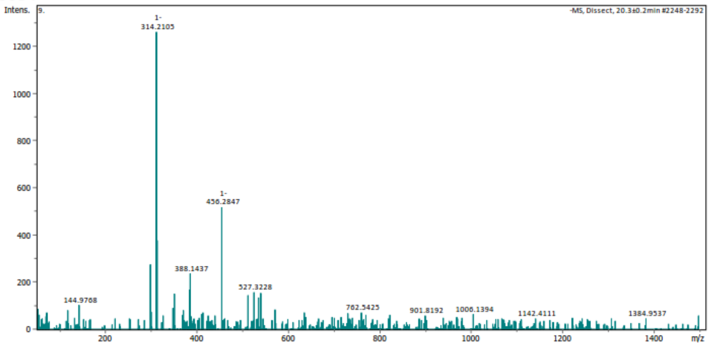

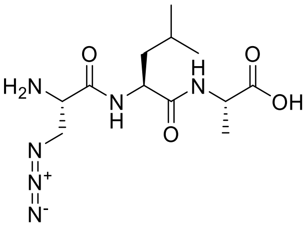

Chemical Formula: $C_{12}H_{22}N_6O_4$

Exact Mass: 314,17

Azidoalaninyl-alaninyl-leucine (or sequence isomers)

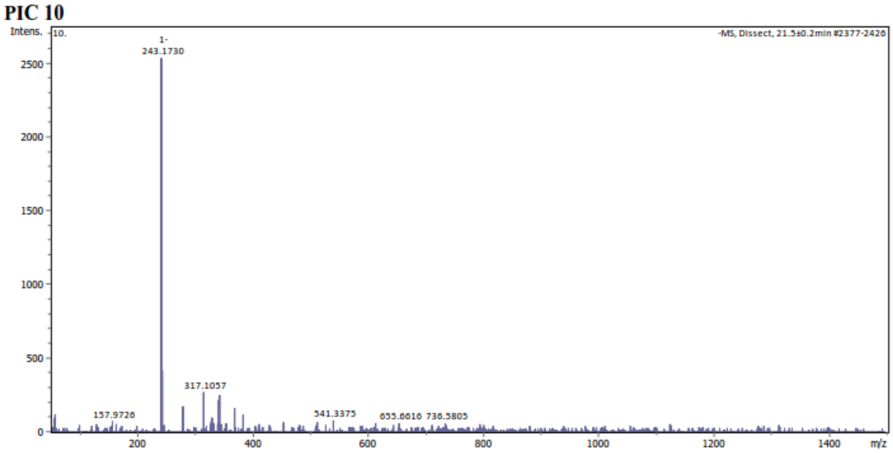

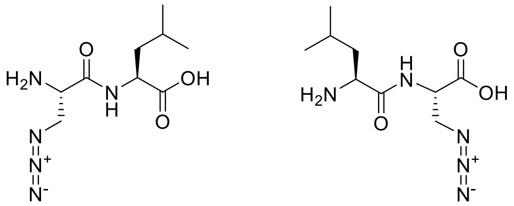

Azidoalaninyl-leucine (left) or leucinyl-azidoalanine (right)

Figure S26. UV (260 nm) and LC-MS chromatograms (negative ion mode), UV peaks, MS peaks and associated compounds obtained for B2 experiment after reaction with GAVLPSAA (312 h).

In order to see if our conditions were compatible with the presence of prebiotic lipidic vesicles, their formation was tested by gentle hydration from dried lipids. For that, 0.5 mL of a methanolic concentrated mixture of lipids containing 10 mM LA:MLG:DLPA (1:1:1) (6.66 mg of LA, 10.9 mg of MLG and 17.1 mg of DLPA in 10 mL of methanol) was diluted in a flask with 0.5 mL of MeOH (5 mM final concentration + 0.02 mol% of DOPE-Rhodamine). The solvent was evaporated using a rotavapor and a bath at 40°C to form a thin lipidic film. After dehydration overnight, the film was rehydrated overnight at room temperature with 1 mL of a buffer solution containing 1 mM EDC and 1 mM DMAP at pH 6.5 (adjusted with 100 mM HCl) prepared as previously for the coupling experiments. After hydration over 16 sh at room temperature, the samples were observed using epifluorescence microscopy but no GV were detected.

3)4) Coupling attempts with NCA in the presence of EDC and EtIm

These experiments were performed very similarly but in one step:

The previously synthesized RNA pUGGU (50 µM, 1 eq, concentration calculated from the UV absorbance at 260 nm and the online service OligoCalc™) was added to 7.5 or 75 equivalents of EtIm (375 µM or 3.75 mM) (C1 or C2 experiment). For that, 3.60 mg of EtIm was dissolved in 1 mL of ultrapure water for a concentrated solution of 37.5 mM later diluted 100 or 10 times (C1 or C2 experiment) to reach the desired concentration in ultrapure water. Then, the pH was adjusted to 8.0 adding 100 mM HCl. Afterwards, 20 or 200 equivalents of EDC (1 mM or 10 mM) (C1 or C2 experiment) were added from a concentrated solution (100 mM, 15.5 mg in 1 mL). The pH was adjusted to 8.0 adding 100 mM NaOH. 20 “equivalents” (950 µM, C1 experiment) or 200 “equivalents” (9.5 mM, C2 experiment) of GAVLAA

NCA were added from a concentrated solution (200 mM) prepared as described previously (Scheme S1). The pH was then adjusted to 8.0 adding 100 mM HCl. The reactions were then left in an Eppendorf vial for 336 h at ambient temperature and without shaking. The pH was checked regularly and remained stable around the pH set after the addition of GAVLAA NCA. 50 µL of the solutions were sampled during the reaction or at least the end of the incubation for LC-MS analysis.

All the products of reaction were analysed using LC-MS with a *Gemini*™ C18 column (250 × 4.6 mm, 5 µm, *Phenomenex*®). The oligonucleotides were separated at 1.0 mL/min using a binary linear gradient from 2 to 44 % B in 28 min using eluants A) 5 mM NH_4OAc pH 8.0 and B) CH_3CN/A 95/5 (Fig. S27-28).

C1 experiment – 336 h:

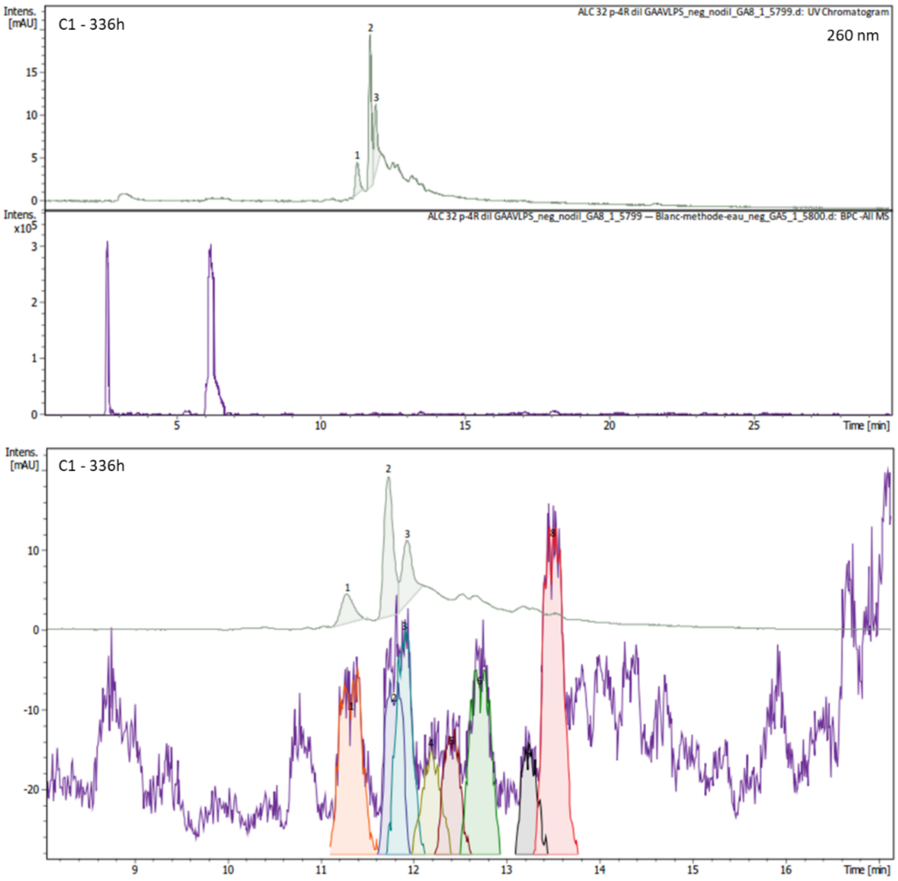

- Composé 1 - Tr = 11.3 min

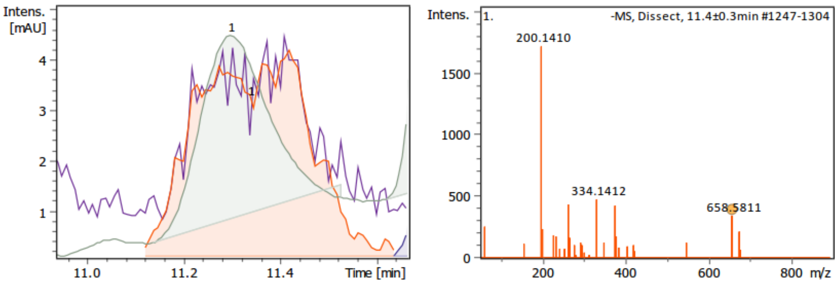

Meas. m/z	Ion Formula	Sum Formula	m/z	err [ppm]	mSigma	Adduct	z
658.5811	C38H47N15O30P4	C38H49N15O30P4	658.5787	-3.5	90.5	M-H	2-

→ Le spectre de masse met en évidence la présence des adduits correspondant à un produit dont la formule brute est la suivante : **$C_{38}H_{49}N_{15}O_{30}P_4$**.

H_2N-pUGGU

- Composé 2 - Tr = 11.8 min

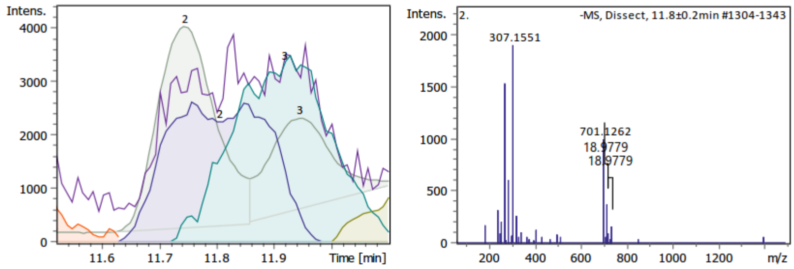

Meas. m/z	Ion Formula	Sum Formula	m/z	err [ppm]	mSigma	Adduct	z
701.1262	C40H63N16O30P5	C40H65N16O30P5	701.1298	5	9.7	M-H	2-
	C43H58N16O30P4	C43H60N16O30P4	701.1233	-4.2	21.5	M-H	2-

→ Le spectre de masse met en évidence la présence des adduits correspondant à un produit dont la formule brute est la suivante : **Voir colonne « Sum Formula ».**

Unidentified derivative

- Composé 3 - Tr = 11.9 min

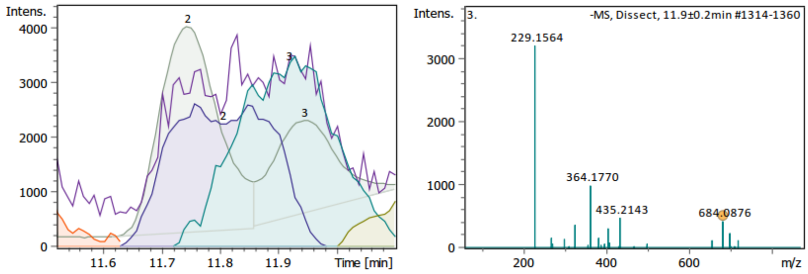

Meas. m/z	Ion Formula	Sum Formula	m/z	err [ppm]	mSigma	Adduct	z
684.0876	C41H48N16O30P4	C41H50N16O30P4	684.0842	-5	36.9	M-H	2-

→ Le spectre de masse met en évidence la présence des adduits correspondant à un produit dont la formule brute est la suivante : **$C_{41}H_{50}N_{16}O_{30}P_4$**.

Im-pUGGU

- Composé 4 - Tr = 12.2 min

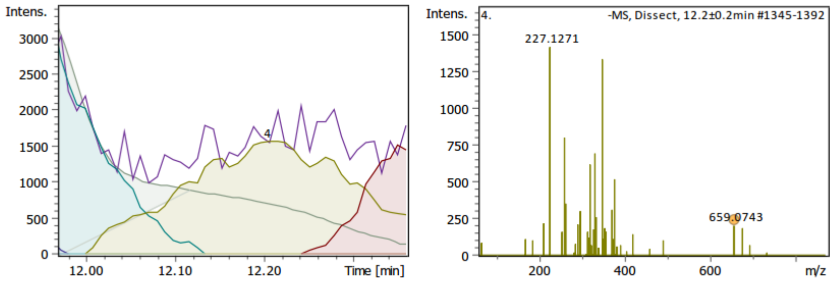

Meas. m/z	Ion Formula	Sum Formula	m/z	err [ppm]	mSigma	Adduct	z
659.0743	C38H46N14O31P4	C38H48N14O31P4	659.0707	-5.5	151.1	M-H	2-

→ Le spectre de masse met en évidence la présence des adduits correspondant à un produit dont la formule brute est la suivante : **$C_{38}H_{48}N_{14}O_{31}P_4$**.

H_2N-pUGGU

- Composé 5 - Tr = 12.7 min

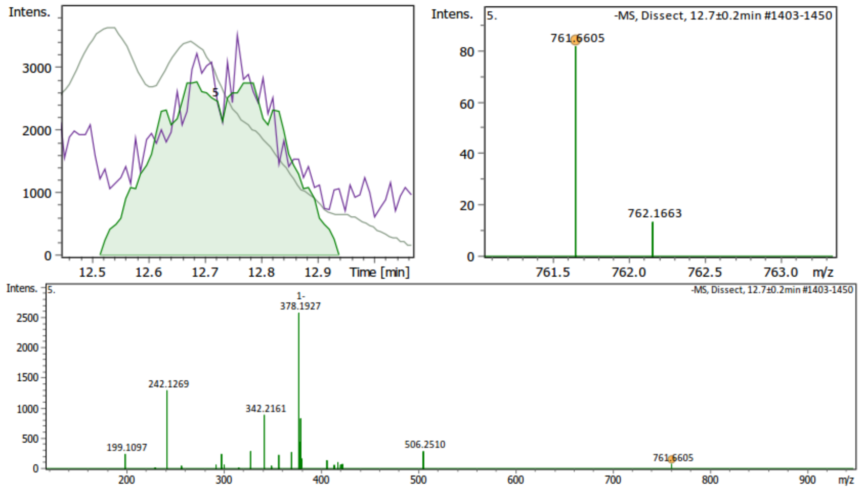

EDC-Im-pUGGU

- Composé 6 - Tr = 13.2 min

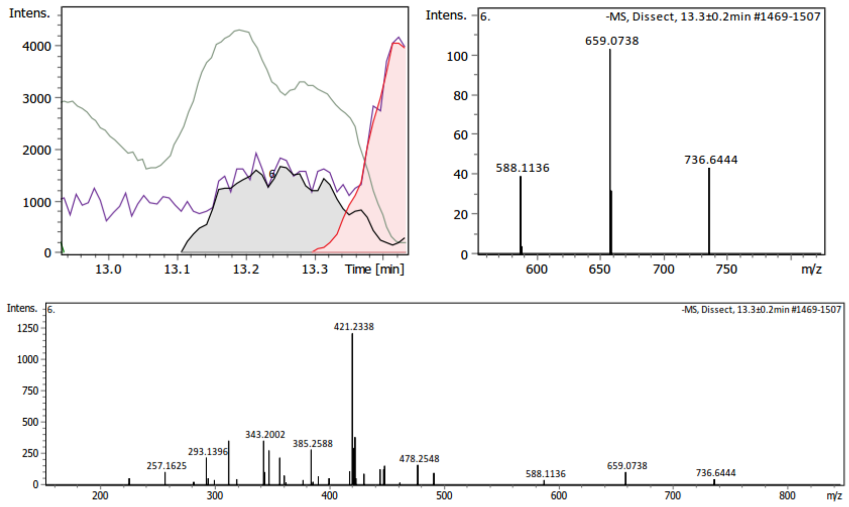

pUGGU and EDC-pUGGU

Figure S27. UV (260 nm) and LC-MS chromatograms (negative ion mode), UV peaks, MS peaks and associated compounds obtained for C1 experiment after reaction with GAVLAA NCA (336 h).

C2 experiment – 336 h:

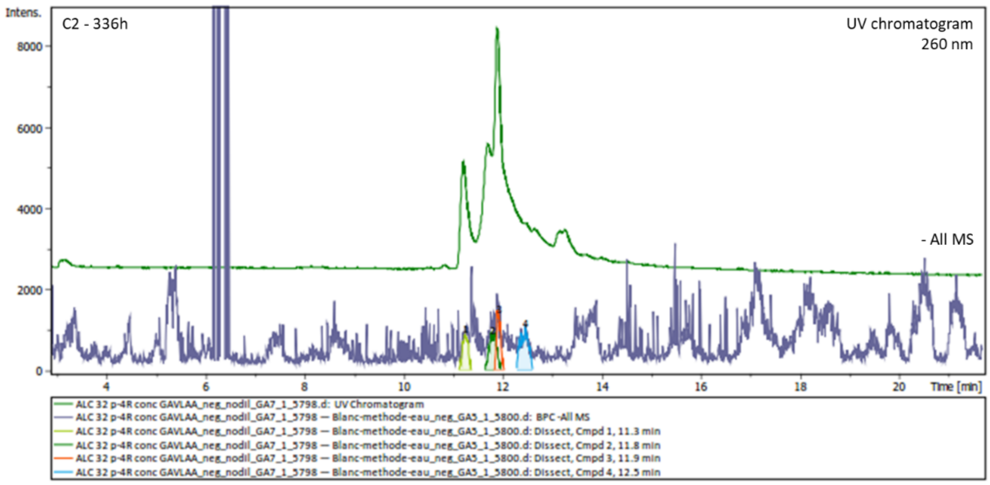

- Composé 1 - Tr = 11.3 min

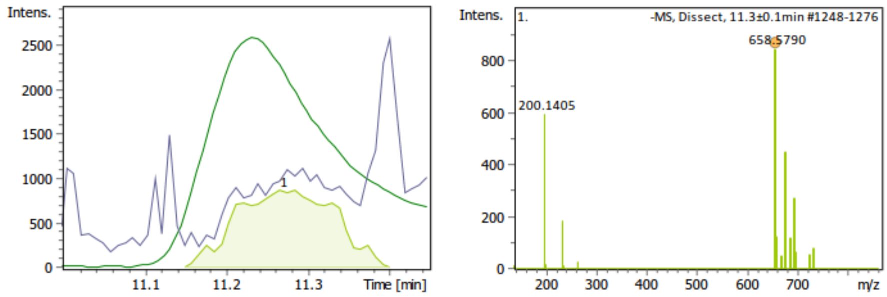

Meas. m/z	Ion Formula	Sum Formula	m/z	err [ppm]	mSigma	Adduct	z
658.579	C38H47N15O30P4	C38H49N15O30P4	658.5787	-0.4	22.7	M-H	2-

→ Le spectre de masse met en évidence la présence des adduits correspondant à un produit dont la formule brute est la suivante : **$C_{38}H_{49}N_{15}O_{30}P_4$**.

H_2N-pUGGU

- Composé 2 - Tr = 11.8 min

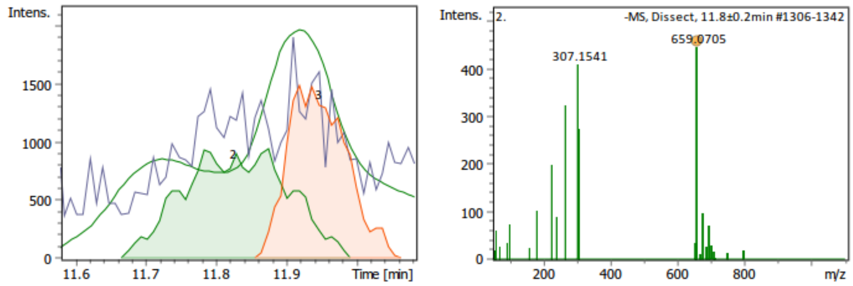

Meas. m/z	Ion Formula	Sum Formula	m/z	err [ppm]	mSigma	Adduct	z
659.0705	C38H46N14O31P4	C38H48N14O31P4	659.0707	0.4	121.9	M-H	2-

→ Le spectre de masse met en évidence la présence des adduits correspondant à un produit dont la formule brute est la suivante : **$C_{38}H_{48}N_{14}O_{31}P_4$**.

pUGGU

- Composé 3 - Tr = 11.9 min

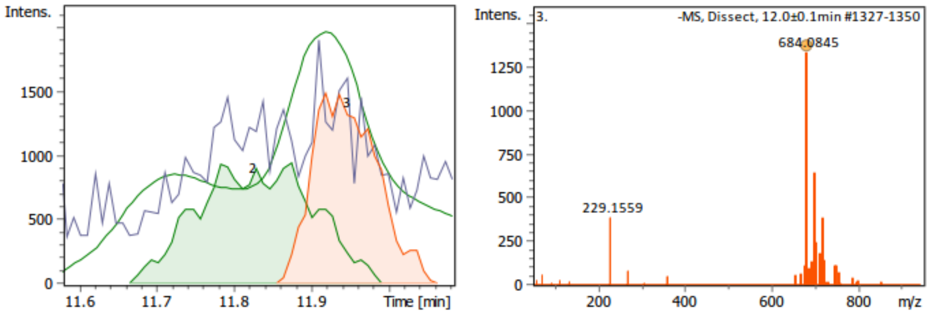

Meas. m/z	Ion Formula	Sum Formula	m/z	err [ppm]	mSigma	Adduct	z
684.0845	C41H48N16O30P4	C41H50N16O30P4	684.0842	-0.4	22.2	M-H	2-

→ Le spectre de masse met en évidence la présence des adduits correspondant à un produit dont la formule brute est la suivante : **$C_{41}H_{50}N_{16}O_{30}P_4$**.

Im-pUGGU

- Composé 4 - Tr = 12.5 min

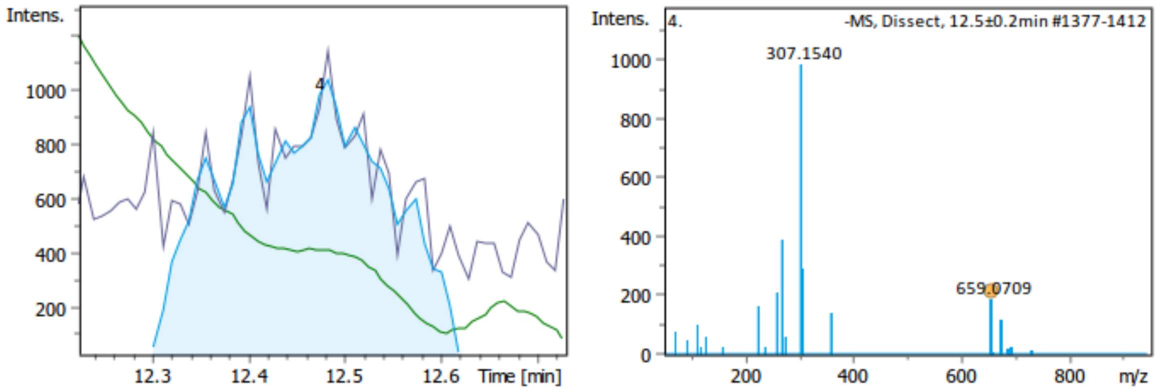

Meas. m/z	Ion Formula	Sum Formula	m/z	err [ppm]	mSigma	Adduct	z
659.0709	C38H46N14O31P4	C38H48N14O31P4	659.0707	-0.2	61.4	M-H	2-

→ Le spectre de masse met en évidence la présence des adduits correspondant à un produit dont la formule brute est la suivante : **$C_{38}H_{48}N_{14}O_{31}P_4$**.

H_2N-pUGGU

Figure S28. UV (260 nm) and LC-MS chromatograms (negative ion mode), UV peaks, MS peaks and associated compounds obtained for C2 experiment after reaction with GAVLAA NCA (336 h).

4) Supplementary information to: Ligation attempts between activated complementary oligonucleotides in prebiotic conditions compatible with the presence of lipidic giant vesicles

The prebiotic ligation reaction investigated corresponded to the formation of longer oligonucleotides after the activation of the terminal phosphate of oligonucleotides into 2’,3’-cyclophosphate with the action of EDC. The additional step involving the formation of another intermediate after addition of the nucleophilic catalyst was omitted for simplicity (Scheme S4).

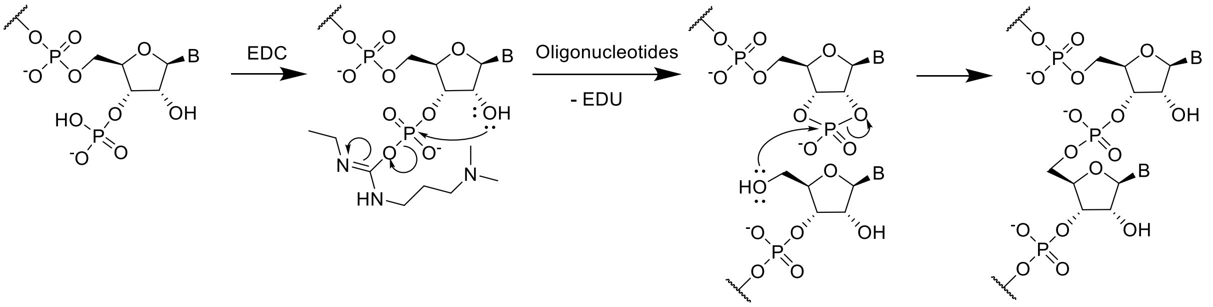

Scheme S4. The formation of oligonucleotides by ligation was studied in the case of activated 2',3'-cyclophosphates oligonucleotides. Such intermediate should be obtained after activation of the 3'-phosphate with a coupling agent such as EDC. B stands for nucleobase and EDU for *N*-ethyl-*N'*-dimethylaminopropylurea. Only the natural 3',5'-dinucleotide linkage is shown, but the 2',5'-linkage can also be formed.

4)1) Oligonucleotides production and analyses

Similarly to the oligonucleotide pUGGU, all the oligonucleotides were generated using solid-support synthesis with the same adapted program and quantities (see 3)1)). The same deprotection steps were also performed without the addition of Et_3N-3HF for oligonucleotides containing only DNA nucleotides (see 3)1)). For the HPLC purification, the same protocols were applied. The only difference was the gradient used that depended on the oligonucleotide (see Fig. S29 for the gradient used with each oligonucleotide). The yields of the oligonucleotides were calculated using the optical density at 260 nm with the absorbance A_{260} of the pure samples obtained after HPLC separation and the online service OligoCalc™ for each oligonucleotide. Hereunder are the chemical analyses of all the oligonucleotides synthesized (Fig. S29).

4Dp oligonucleotide:

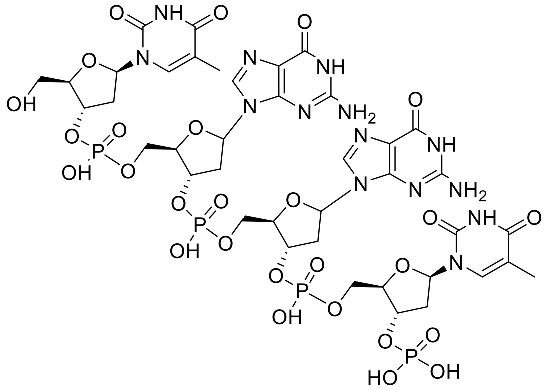

Chemical Formula: $C_{40}H_{52}N_{14}O_{27}P_4$
Molecular Weight: 1284,82

4Dp MS analysis and UV analytical HPLC chromatogram (260 nm):

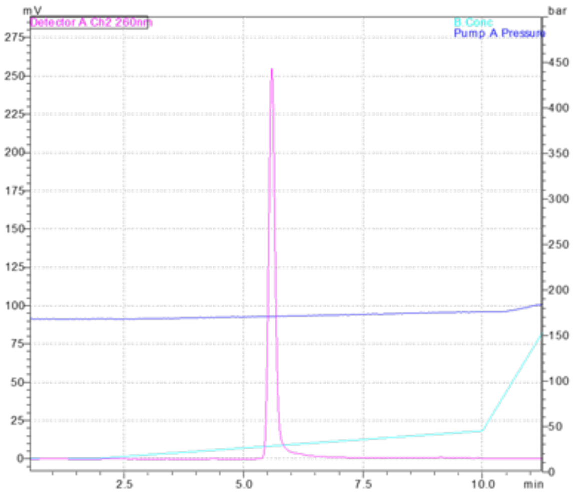

Display Report

Analysis Info

Analysis Name	D:\Data\Data\2021\2021_09\Impact2_2100902_01_AL B 100 4D-p 1.d	Acquisition Date	9/2/2021 1:45:11 PM
Method	Tune_pos_Standard.m	Operator	Demo User
Sample Name		Instrument	impact II 1825265.10081
Comment			

Acquisition Parameter

Source Type	ESI	Ion Polarity	Negative	Set Nebulizer	0.3 Bar
Focus	Active	Set Capillary	4500 V	Set Dry Heater	200 °C
Scan Begin	50 m/z	Set End Plate Offset	-500 V	Set Dry Gas	4.0 l/min
Scan End	2500 m/z	Set Charging Voltage	2000 V	Set Divert Valve	Source
		Set Corona	0 nA	Set APCI Heater	0 °C

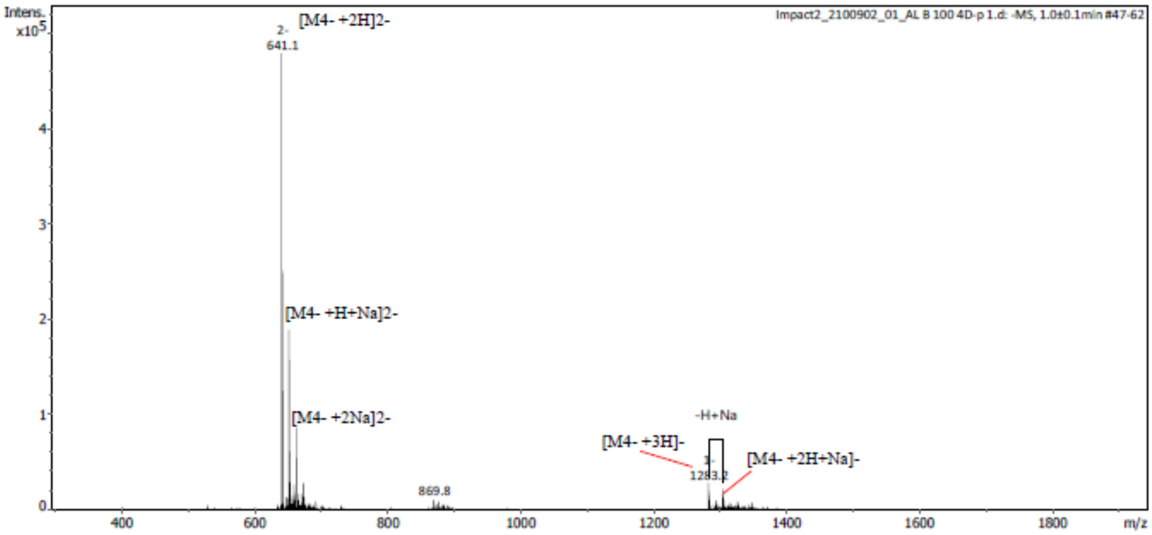

Impact2_2100902_01_AL B 100 4D-p 1.d

Bruker Compass DataAnalysis 5.2 printed: 9/2/2021 1:50:35 PM by: demo Page 1 of 1

p4D oligonucleotide:

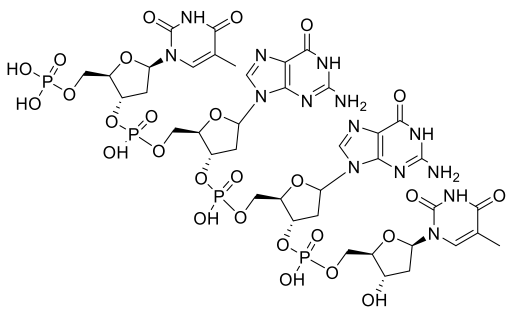

Chemical Formula: $C_{40}H_{52}N_{14}O_{27}P_4$

Molecular Weight: 1284,82

p4D MS analysis and UV analytical HPLC chromatogram (260 nm):

Display Report

Analysis Info

Analysis Name	D:\Data\Data\2021\2021_11\Impact2_211116_07_ALB 124 p-4D 1.d	Acquisition Date	11/16/2021 4:50:43 PM
Method	Tune_pos_Standard.m	Operator	Demo User
Sample Name		Instrument	impact II 1825265.10081
Comment			

Acquisition Parameter

Source Type	ESI	Ion Polarity	Negative	Set Nebulizer	0.3 Bar
Focus	Active	Set Capillary	4500 V	Set Dry Heater	200 °C
Scan Begin	50 m/z	Set End Plate Offset	-500 V	Set Dry Gas	4.0 l/min
Scan End	3000 m/z	Set Charging Voltage	2000 V	Set Divert Valve	Source
		Set Corona	0 nA	Set APCI Heater	0 °C

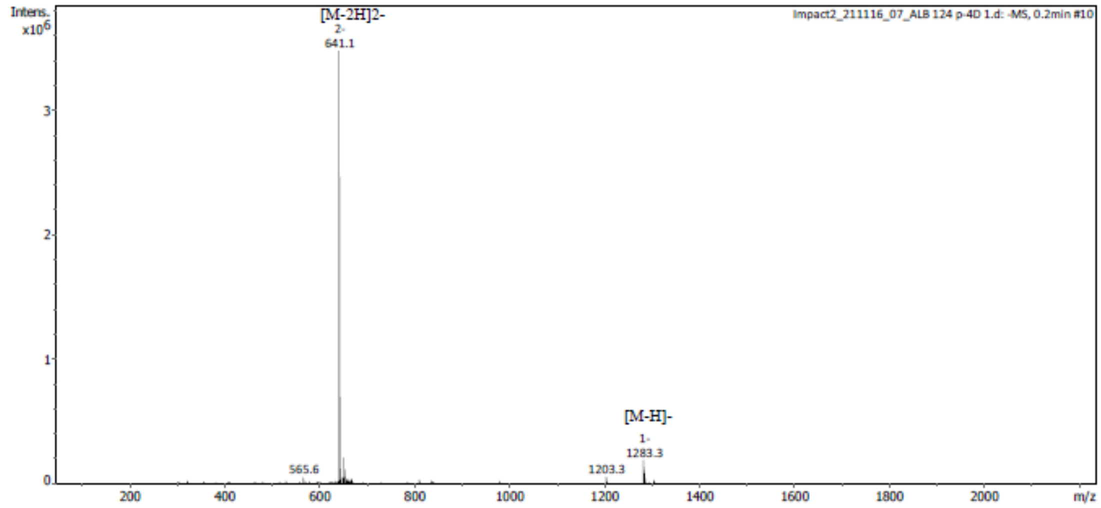

Impact2_211116_07_ALB 124 p-4D 1.d

Bruker Compass DataAnalysis 5.2 printed: 11/16/2021 4:53:49 PM by: demo Page 1 of 1

Datafile Name:p-4D purity OD measures.lcd
Sample Name:p-4D purity OD measures

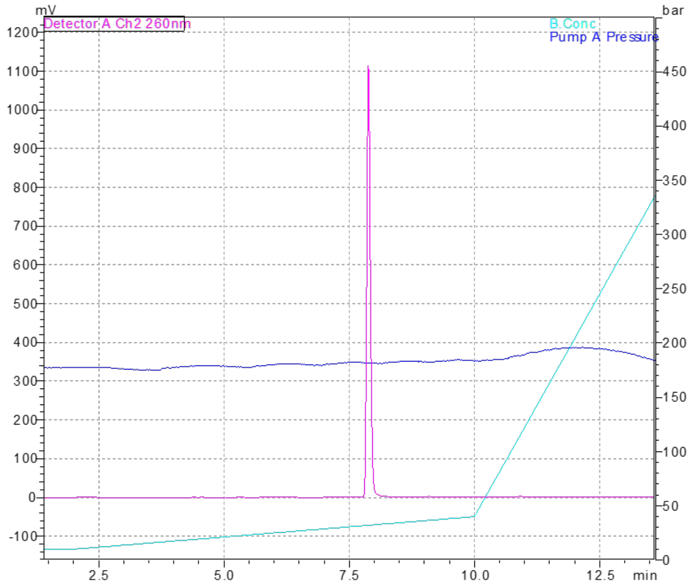

4Hp oligonucleotide:

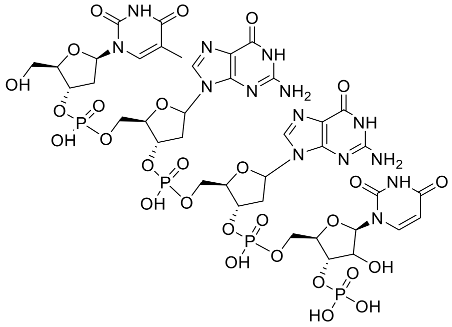

Chemical Formula: $C_{39}H_{50}N_{14}O_{28}P_4$
Molecular Weight: 1286,79

4Hp MS analysis and UV analytic HPLC chromatogram (260 nm):

Display Report

Analysis Info

Analysis Name	D:\Data\Data\2021\2021_09\Impact2_2100902_02_AL B 101 4H 1.d	Acquisition Date	9/2/2021 2:50:52 PM
Method	Tune_pos_Standard.m	Operator	Demo User
Sample Name		Instrument	Impact II 1825265.10081
Comment			

Acquisition Parameter

Source Type	ESI	Ion Polarity	Negative	Set Nebulizer	0.3 Bar
Focus	Active	Set Capillary	4500 V	Set Dry Heater	200 °C
Scan Begin	50 m/z	Set End Plate Offset	-500 V	Set Dry Gas	4.0 l/min
Scan End	2500 m/z	Set Charging Voltage	2000 V	Set Divert Valve	Source
		Set Corona	0 nA	Set APCI Heater	0 °C

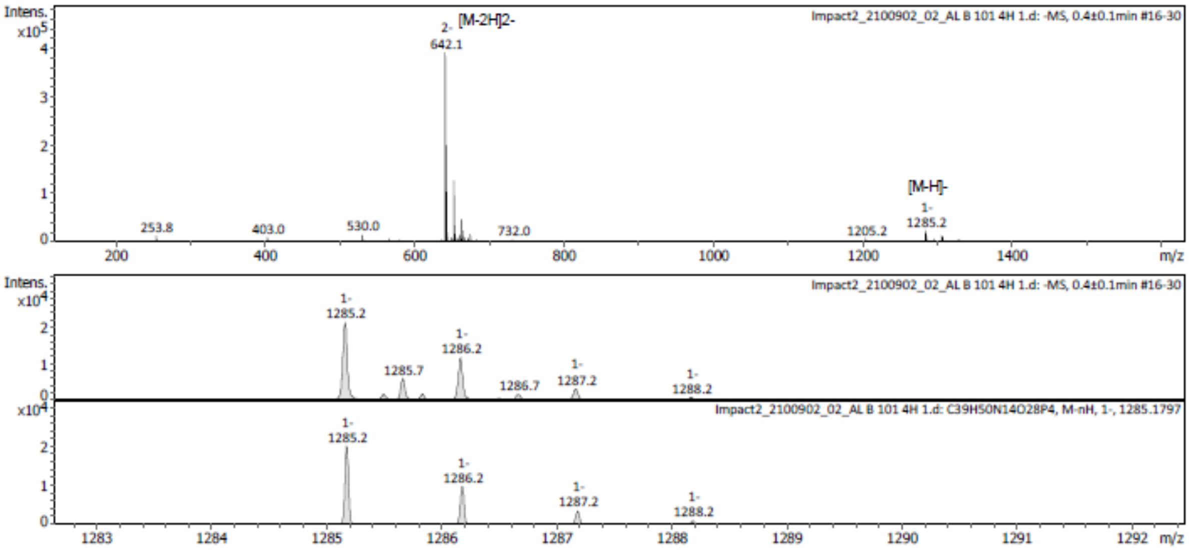

Impact2_2100902_02_AL B 101 4H 1.d
Bruker Compass DataAnalysis 5.2 printed: 9/2/2021 2:53:20 PM by: demo Page 1 of 1

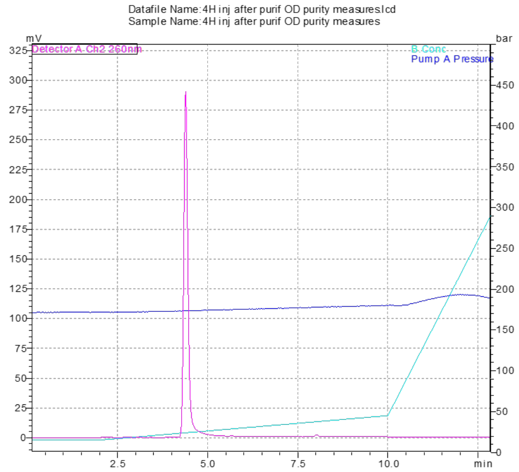

4Rp oligonucleotide:

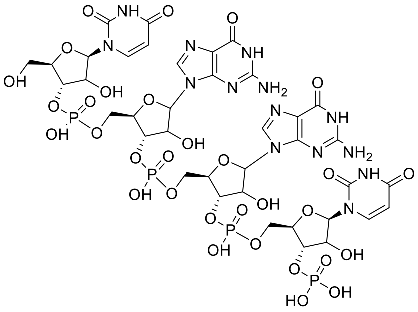

Chemical Formula: $C_{38}H_{48}N_{14}O_{31}P_4$
Molecular Weight: 1320,76

4Rp MS analysis and UV analytical HPLC chromatogram (260 nm):

Display Report

Analysis Info

Analysis Name	D:\Data\Data\2022\2022_01\Impact2_220118_03_ALB 137 4R-p.d	Acquisition Date	1/18/2022 8:53:20 AM
Method	Tune_pos_Standard.m	Operator	Demo User
Sample Name		Instrument	impact II 1825265.10081
Comment			

Acquisition Parameter

Source Type	ESI	Ion Polarity	Negative	Set Nebulizer	0.3 Bar
Focus	Active	Set Capillary	4500 V	Set Dry Heater	200 °C
Scan Begin	50 m/z	Set End Plate Offset	-500 V	Set Dry Gas	4.0 l/min
Scan End	1500 m/z	Set Charging Voltage	2000 V	Set Divert Valve	Source
		Set Corona	0 nA	Set APCI Heater	0 °C

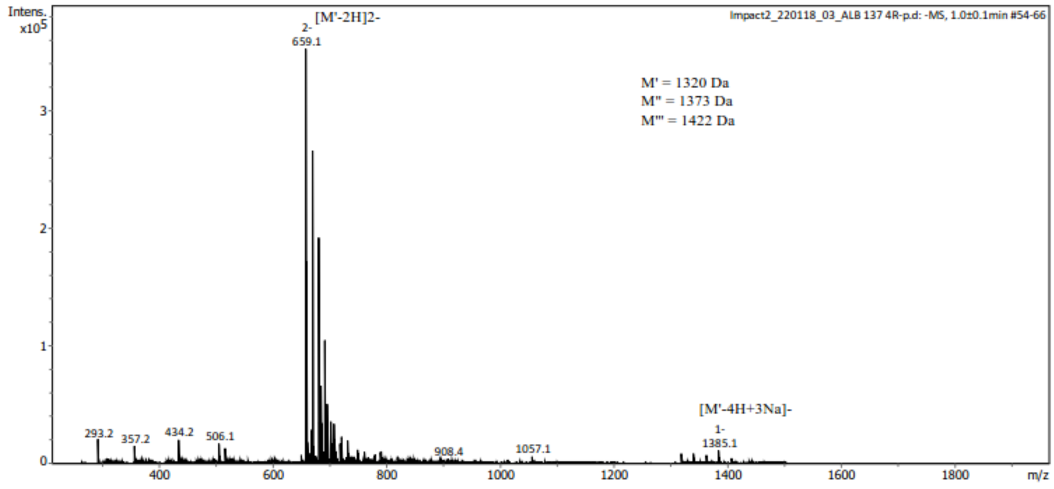

Impact2_220118_03_ALB 137 4R-p.d

Bruker Compass DataAnalysis 5.2 printed: 1/18/2022 9:17:47 AM by: demo Page 1 of 1

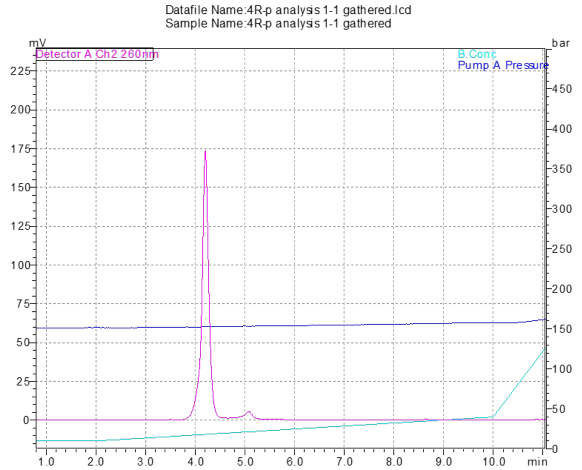

5Dp oligonucleotide:

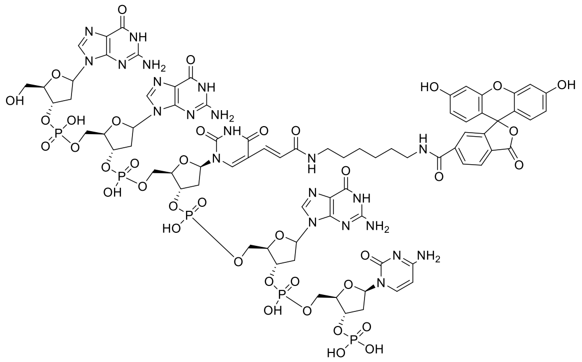

Chemical Formula: $C_{78}H_{87}N_{22}O_{39}P_5$
Molecular Weight: 2111,54

5Dp MS analysis, UV (260 nm) and fluorescence (490/510 nm) analytical HPLC chromatograms:

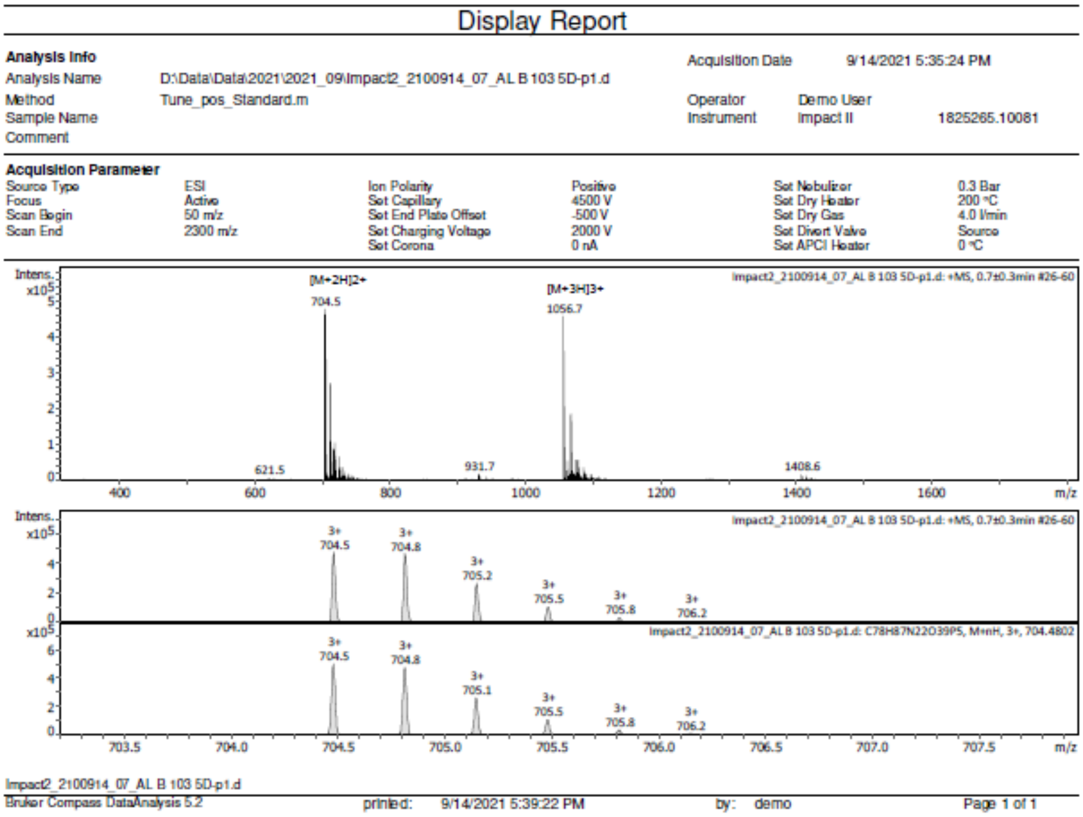

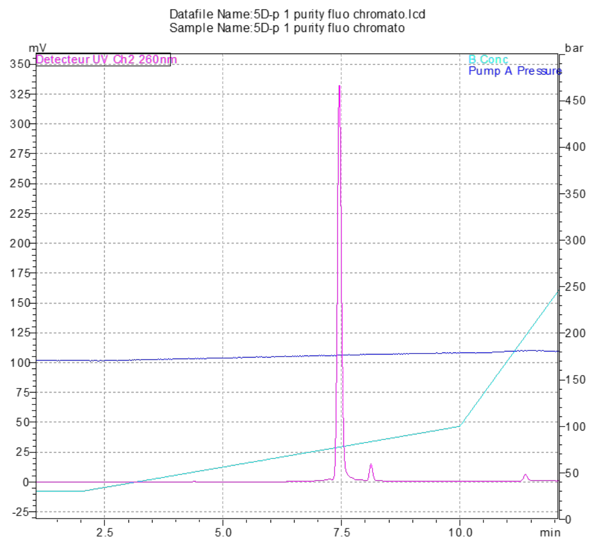

Datafile Name:5D-p 1 purity fluo chromato.lcd
Sample Name:5D-p 1 purity fluo chromato
Detecteur UV Ch2 260nm
B.Conc.
Pump A Pressure
mV
bar
min

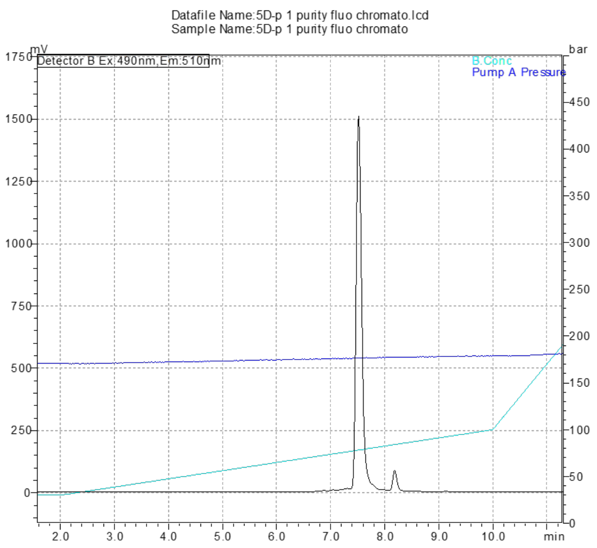

Datafile Name:5D-p 1 purity fluo chromato.lcd
Sample Name:5D-p 1 purity fluo chromato
Detector B Ex:490nm,Em:510nm
B.Conc.
Pump A Pressure
mV
bar
min

p5D oligonucleotide:

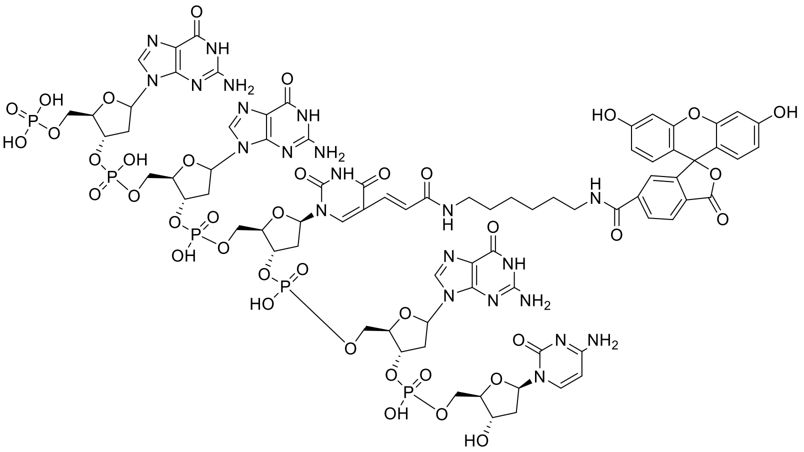

Chemical Formula: $C_{78}H_{87}N_{22}O_{39}P_5$
Molecular Weight: 2111,54

p5D MS analysis, UV (260 nm) and fluorescence (490/510 nm) analytical HPLC chromatograms:

Display Report

Analysis Info

Analysis Name	D:\Data\Data\2021\2021_11\Impact2_211104_06_ALB 120 p-5D 1.d	Acquisition Date	11/4/2021 2:47:29 PM
Method	Tune_pos_Standard.m	Operator	Demo User
Sample Name		Instrument	impact II 1825265.10081
Comment			

Acquisition Parameter

Source Type	ESI	Ion Polarity	Negative	Set Nebulizer	0.3 Bar
Focus	Active	Set Capillary	4500 V	Set Dry Heater	200 °C
Scan Begin	50 m/z	Set End Plate Offset	-500 V	Set Dry Gas	4.0 l/min
Scan End	2500 m/z	Set Charging Voltage	2000 V	Set Divert Valve	Source
		Set Corona	0 nA	Set APCI Heater	0 °C

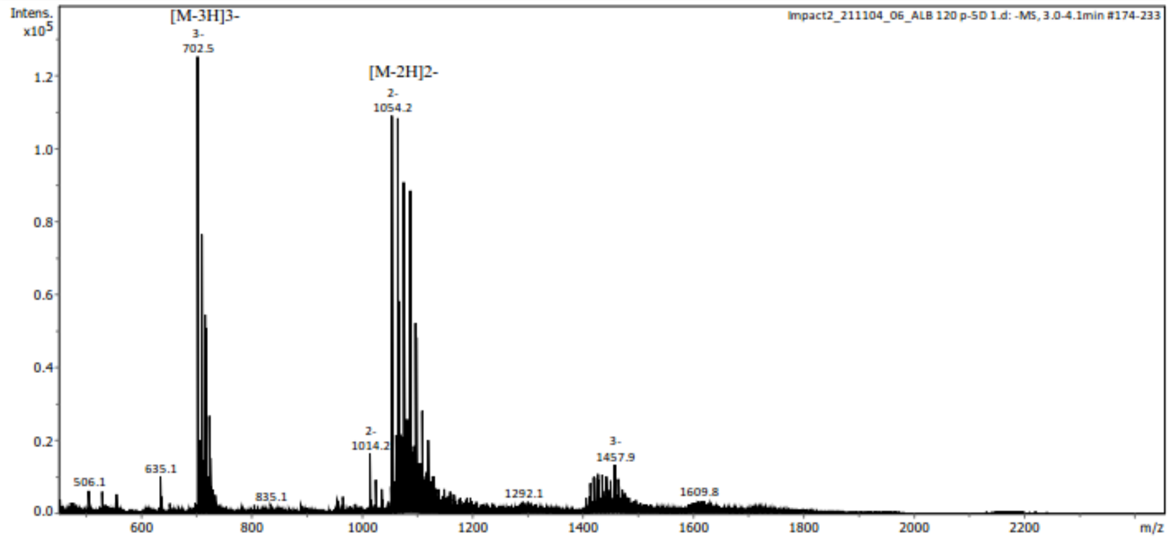

Impact2_211104_06_ALB 120 p-5D 1.d
Bruker Compass DataAnalysis 5.2 printed: 11/4/2021 2:54:22 PM by: demo Page 1 of 1

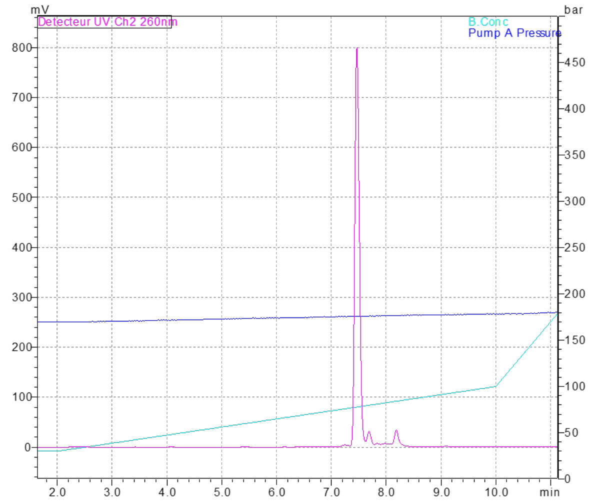

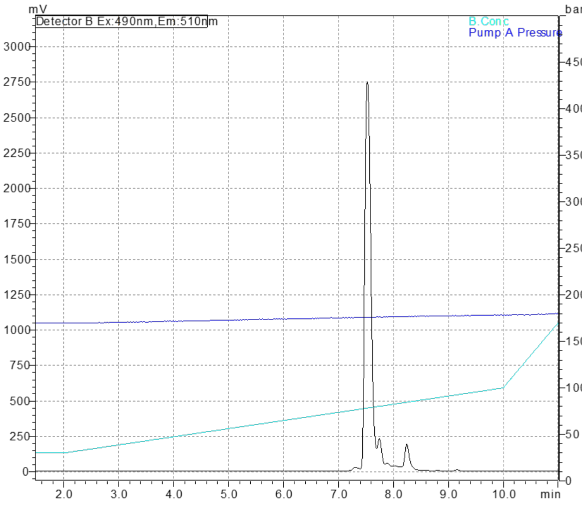

5Hp oligonucleotide:

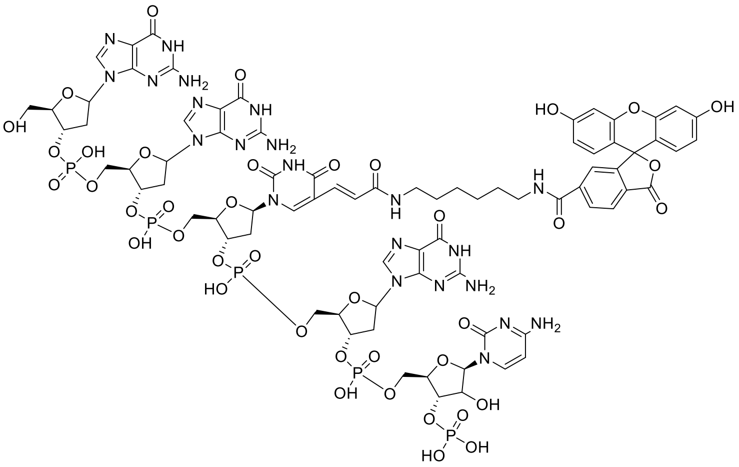

Chemical Formula: $C_{78}H_{87}N_{22}O_{40}P_5$
Molecular Weight: 2127,54

5Hp MS analysis, UV (260 nm) and fluorescence (490/510 nm) analytical HPLC chromatograms:

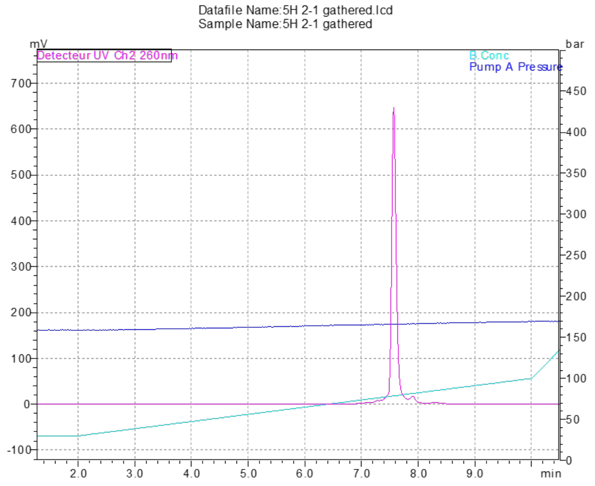

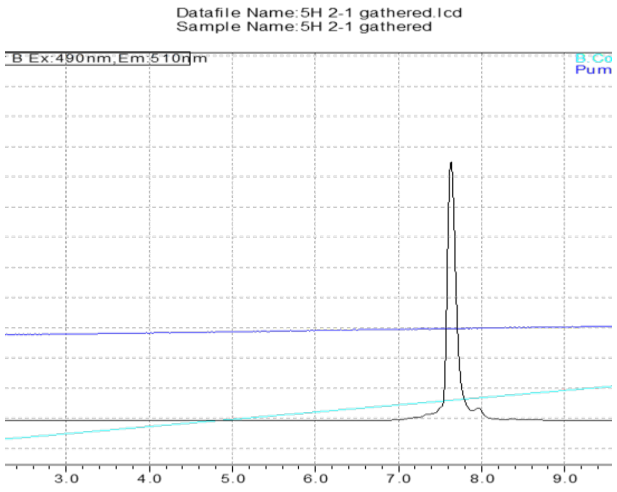

Display Report

Analysis Info

Analysis Name	D:\Data\Data\2021\2021_12\Impact2_211214_02_ALB 133 5H 2.d	Acquisition Date	12/14/2021 7:50:45 AM
Method	Tune_pos_Standard.m	Operator	Demo User
Sample Name		Instrument	impact II 1825265.10081
Comment			

Acquisition Parameter

Source Type	ESI	Ion Polarity	Negative	Set Nebulizer	0.3 Bar
Focus	Active	Set Capillary	4500 V	Set Dry Heater	200 °C
Scan Begin	100 m/z	Set End Plate Offset	-500 V	Set Dry Gas	4.0 l/min
Scan End	3000 m/z	Set Charging Voltage	2000 V	Set Divert Valve	Source
		Set Corona	0 nA	Set APCI Heater	0 °C

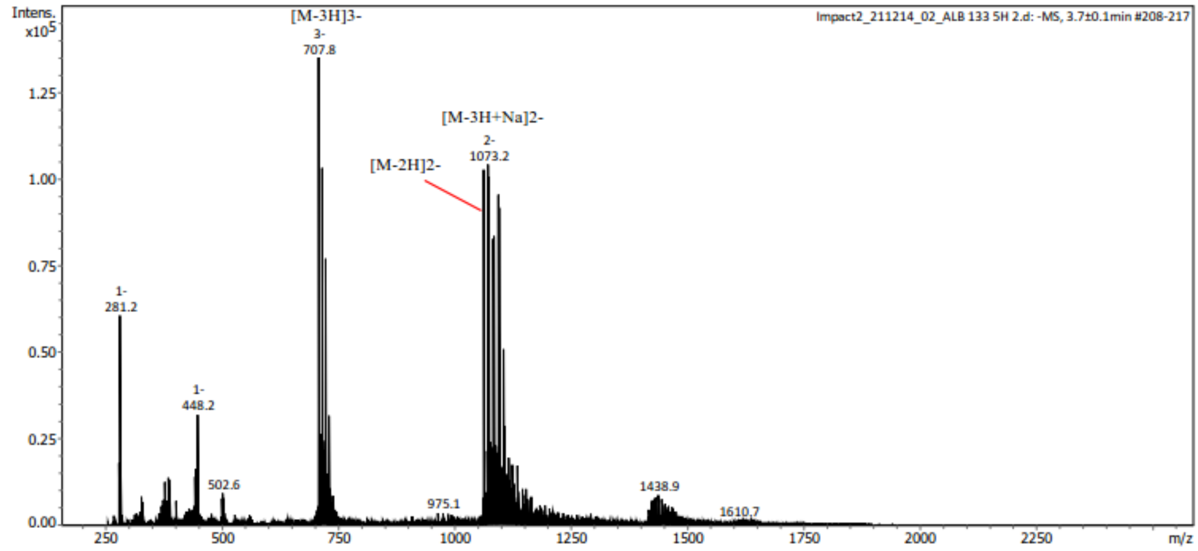

Impact2_211214_02_ALB 133 5H 2.d

Bruker Compass DataAnalysis 5.2 printed: 12/14/2021 8:11:18 AM by: demo Page 1 of 1

5Rp oligonucleotide:

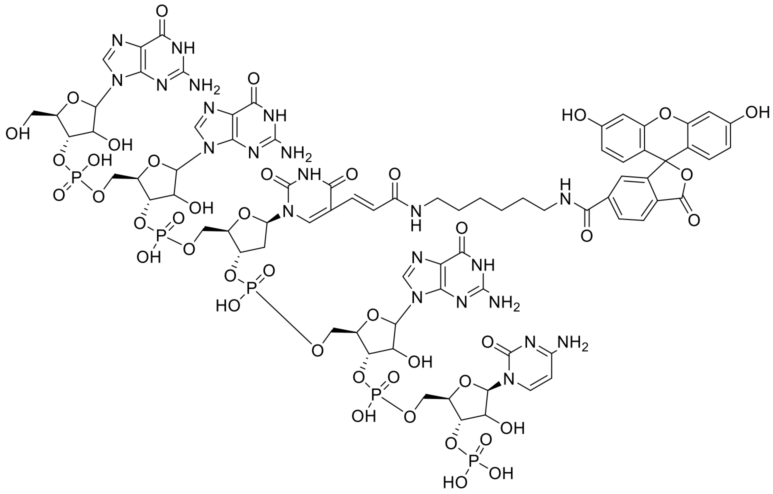

Chemical Formula: $C_{78}H_{87}N_{22}O_{43}P_5$

Molecular Weight: 2175,53

5Rp MS analysis, UV (260 nm) and fluorescence (490/510 nm) analytical HPLC chromatograms:

Display Report

Analysis Info

Analysis Name	D:\Data\Data\2022\2022_01\Impact2_220106_04_ALB 136 5R-p.d	Acquisition Date	1/6/2022 1:06:34 PM
Method	Tune_pos_Standard.m	Operator	Demo User
Sample Name		Instrument	impact II 1825265.10081
Comment			

Acquisition Parameter

Source Type	ESI	Ion Polarity	Negative	Set Nebulizer	0.3 Bar
Focus	Active	Set Capillary	4500 V	Set Dry Heater	200 °C
Scan Begin	50 m/z	Set End Plate Offset	-500 V	Set Dry Gas	4.0 l/min
Scan End	3000 m/z	Set Charging Voltage	2000 V	Set Divert Valve	Source
		Set Corona	0 nA	Set APCI Heater	0 °C

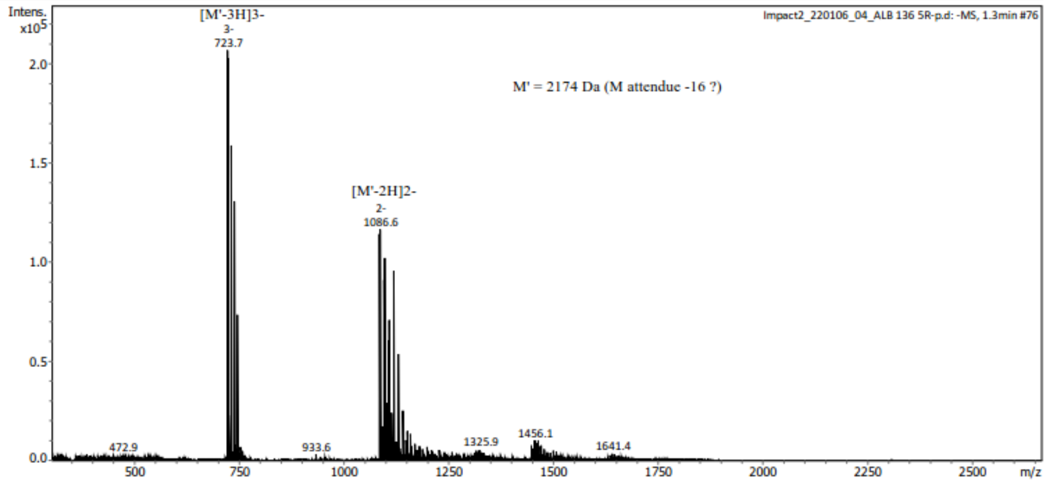

Impact2_220106_04_ALB 136 5R-p.d

Bruker Compass DataAnalysis 5.2 printed: 1/6/2022 1:15:12 PM by: demo Page 1 of 1

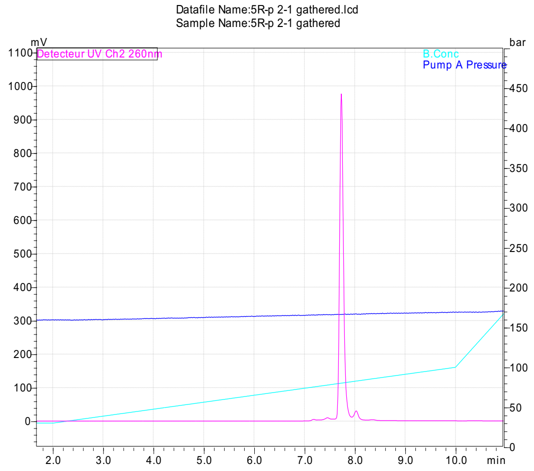

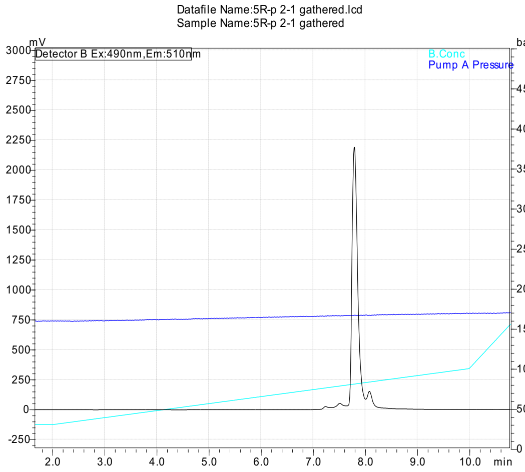

p5R oligonucleotide:

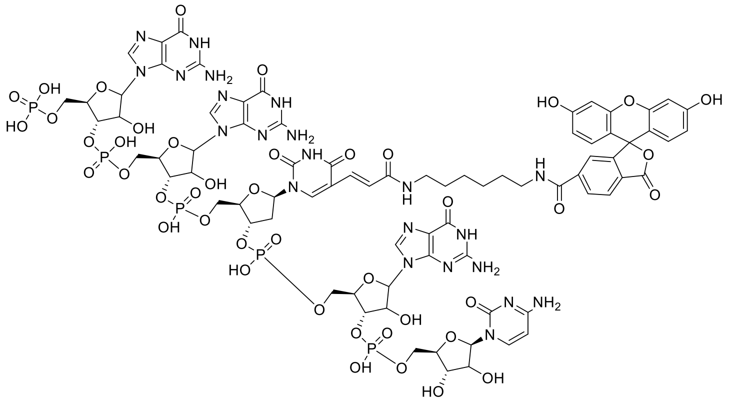

Chemical Formula: $C_{78}H_{87}N_{22}O_{43}P_5$
Molecular Weight: 2175,53

p5R MS analysis, UV (260 nm) and fluorescence (490/510 nm) analytical HPLC chromatograms:

Display Report

Analysis Info

Analysis Name	D:\Data\Data\2022\2022_01\Impact2_220125_01_ALB 139 p-5R 2.d	Acquisition Date	1/25/2022 8:20:43 AM
Method	Tune_pos_Standard.m	Operator	Demo User
Sample Name		Instrument	impact II 1825265.10081
Comment			

Acquisition Parameter

Source Type	ESI	Ion Polarity	Negative	Set Nebulizer	0.3 Bar
Focus	Active	Set Capillary	4500 V	Set Dry Heater	200 °C
Scan Begin	50 m/z	Set End Plate Offset	-500 V	Set Dry Gas	4.0 l/min
Scan End	2500 m/z	Set Charging Voltage	2000 V	Set Divert Valve	Source
		Set Corona	0 nA	Set APCI Heater	0 °C

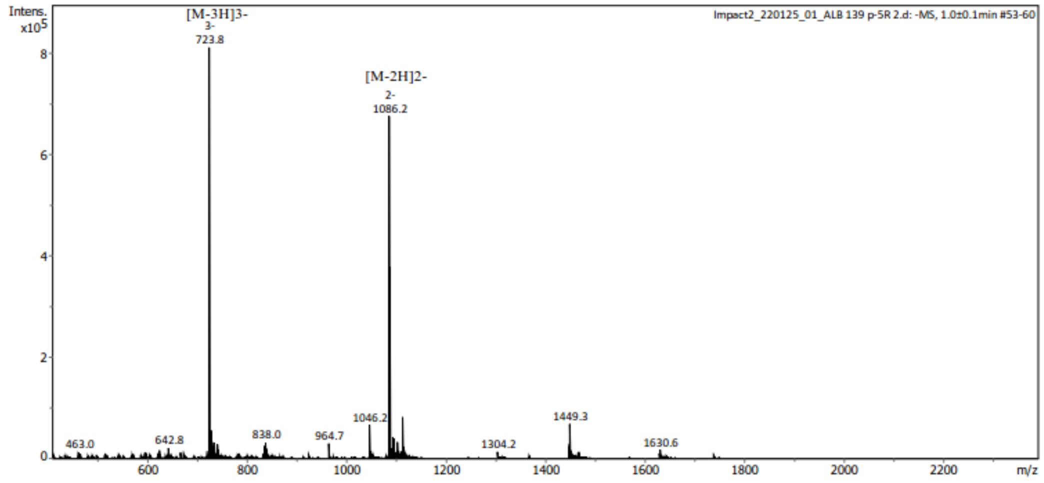

Impact2_220125_01_ALB 139 p-5R 2.d
Bruker Compass DataAnalysis 5.2 printed: 1/25/2022 8:33:36 AM by: demo Page 1 of 1

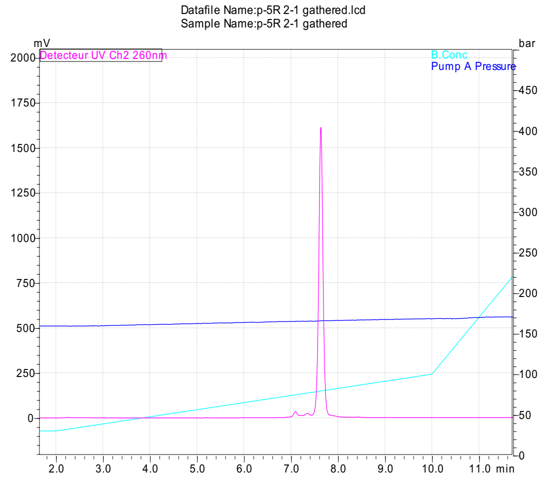

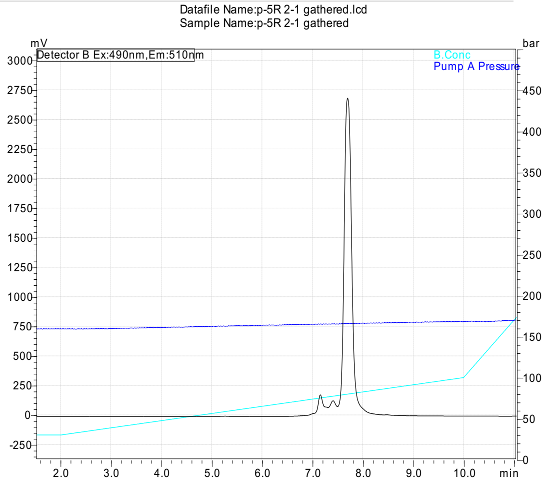

9D oligonucleotide:

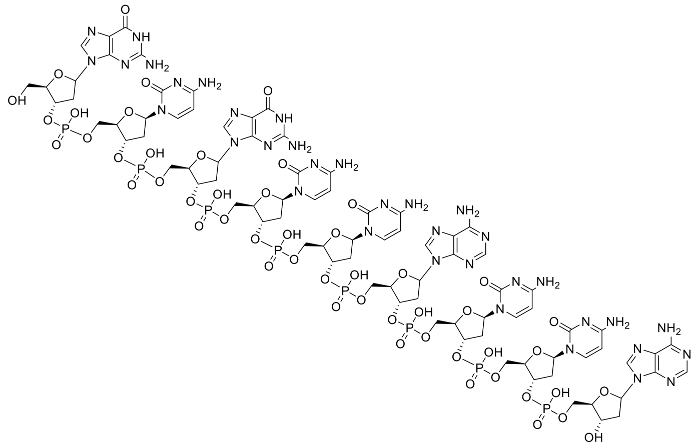

Chemical Formula: $C_{85}H_{109}N_{35}O_{50}P_8$
Molecular Weight: 2668,79

9D MS analysis and UV analytic HPLC chromatogram (260 nm):

Display Report

Analysis Info

Analysis Name	D:\Data\Data\2021\2021_09\Impact2_2100902_04_AL B 101 9D 1.d	Acquisition Date	9/2/2021 4:14:52 PM
Method	Tune_pos_Standard.m	Operator	Demo User
Sample Name		Instrument	Impact II 1825265.10081
Comment			

Acquisition Parameter

Source Type	ESI	Ion Polarity	Negative	Set Nebulizer	0.3 Bar
Focus	Active	Set Capillary	4500 V	Set Dry Heater	200 °C
Scan Begin	50 m/z	Set End Plate Offset	-500 V	Set Dry Gas	4.0 l/min
Scan End	3500 m/z	Set Charging Voltage	2000 V	Set Divert Valve	Source
		Set Corona	0 nA	Set APCI Heater	0 °C

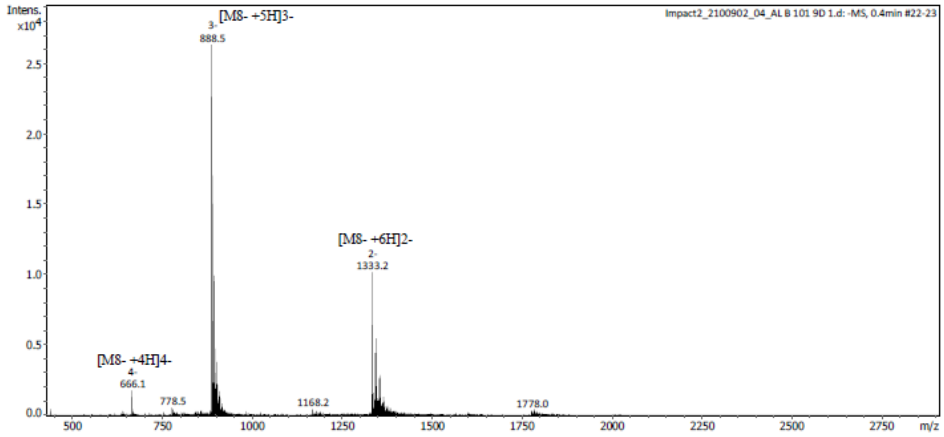

Impact2_2100902_04_AL B 101 9D 1.d
Bruker Compass DataAnalysis 5.2 printed: 9/2/2021 4:20:45 PM by: demo Page 1 of 1

Datafile Name:9D purity measure pH 8.0.lcd
Sample Name:9D purity measure pH 8.0

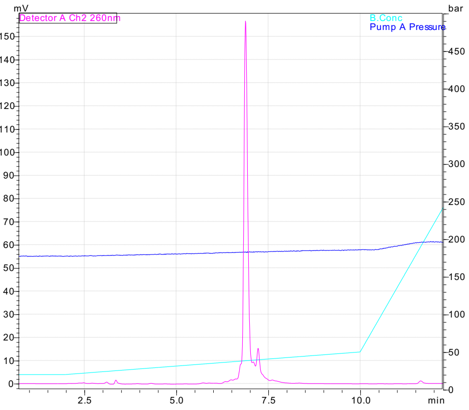

9R oligonucleotide:

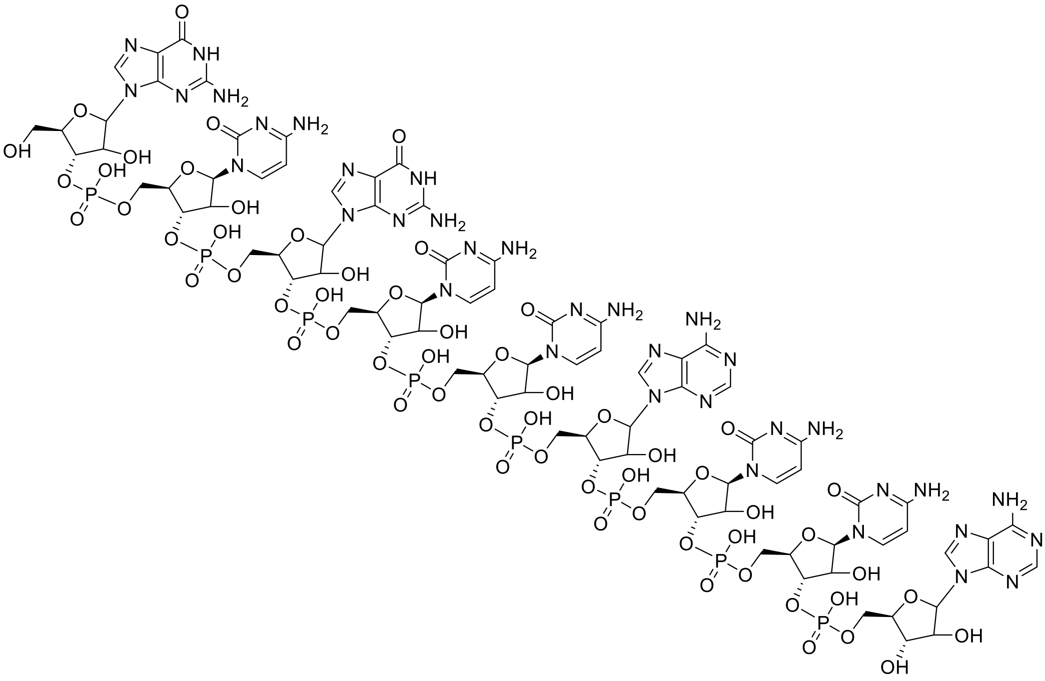

Chemical Formula: $C_{85}H_{109}N_{35}O_{59}P_8$
Molecular Weight: 2812,78

9R MS analysis and UV analytic HPLC chromatogram (260 nm):

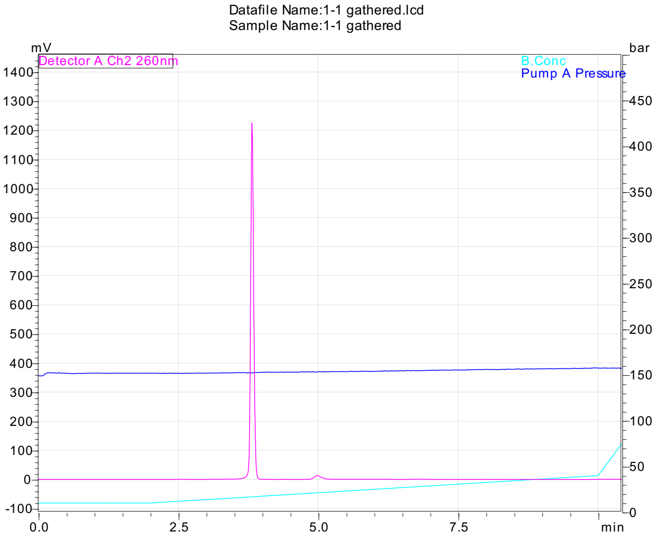

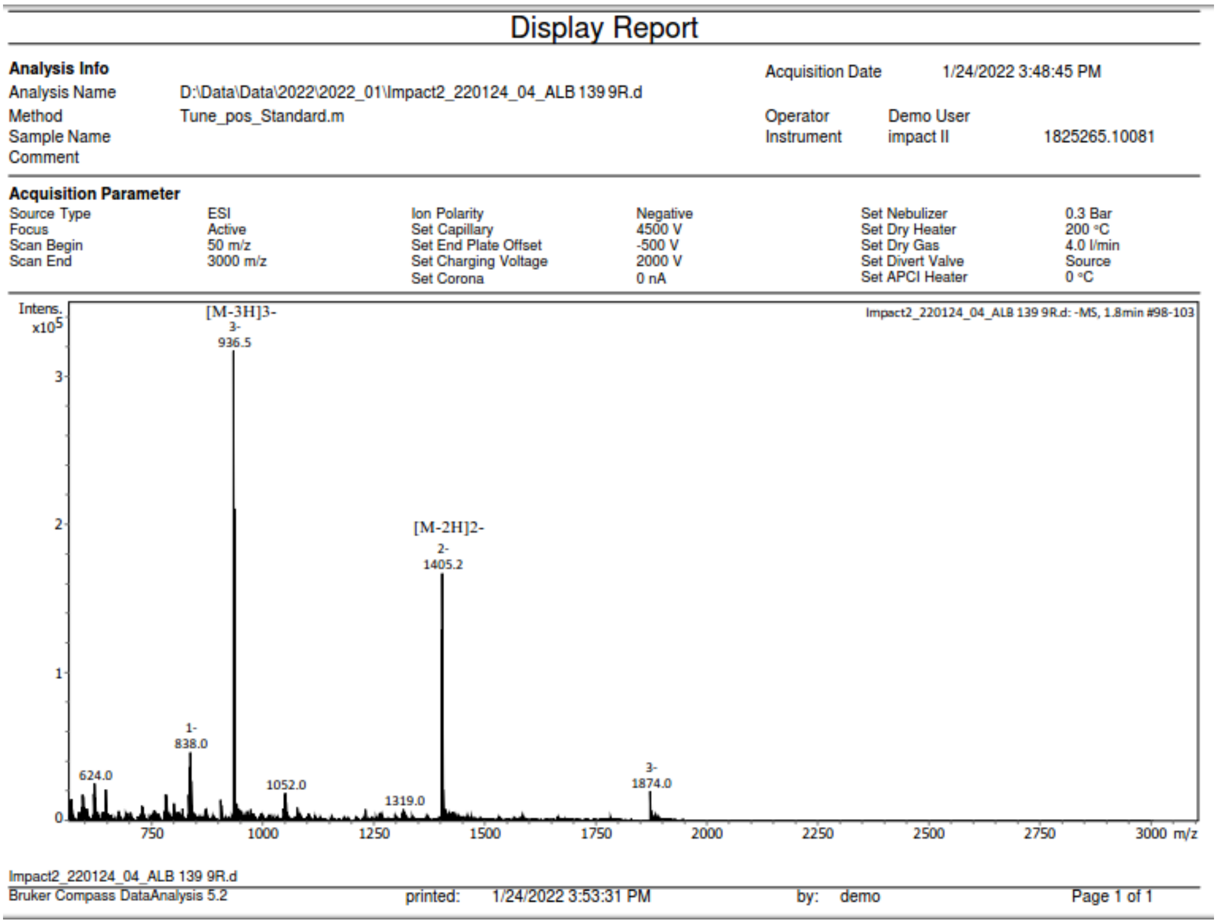

Sequence	Gradient used	Purity (%)	Concentration determination (1 mL of solution with an absorbance of 1 at 260 nm contains [] µM of oligonucleotides)	Yield (%)	m/z	m/z found
4Dp d(TGGT)-P	3 to 9% B in 8 min	99.8	24.498	8.7	1284.8	1284.2
p4D P-d(TGGT)	3 to 9% B in 8 min	99.6	24.498	39.5	1284.8	1284.2
4Hp d(TGG)U-P	3 to 9% B in 8 min	99.6	23.685	11.4	1286.8	1286.2
4Rp UGGU-P	2 to 8% B in 8 min	97.1	21.097	12.4	1320.8	1320.2
p4R P-UGGU	2 to 8% B in 8 min	96.2	21.097	38.9	1320.8	1320.2
5Dp d(GGfTGC)-P	6 to 20% B in 8 min	95.4	15.342	8.8	2111.5	2111.5
p5D P-d(GGfTGC)	6 to 20% B in 8 min	95.3	15.342	25.1	2111.5	2110.5
5Hp d(GGfTG)C-P	6 to 20% B in 8 min	98.7	15.342	19.7	2127.5	2126.4
5Rp GGd(fT)GC-P	6 to 20% B in 8 min	97.3	13.661	21.1	2175.5	2174.2
p5R P-GGd(fT)GC	6 to 20% B in 8 min	98.2	13.661	28.8	2175.5	2174.4
9D d(GCGCCACCA)	5 to 11% B in 8 min	96.5	11.152	2.2	2668.8	2668.5
9R GCGCCACCA	4 to 10% B in 8 min	97.5	9.69	6.2	2812.8	2812.5

Figure S29. For each oligonucleotide, the MS analysis (negative ion mode), the UV chromatogram (260 nm) (and the fluorescence chromatogram (490/510 nm) for 5-mers only) are displayed. As a summary, the table gives for each sequence, the HPLC gradient used for the separation, the purity (calculated with the UV chromatogram displayed), the micromolar concentration of an oligonucleotide solution in 1 mL to have an absorption A_{260} = 1.000 through 1 cm path length (calculated with OligoCalc™), the yield calculated from the measured A_{260}, the *m/z* ratio of the oligonucleotide and the *m/z* ratio of the product found by MS analysis, both for *z* = –1 (mono-anion). Fluorescent thymidylate = fT.

4)2) Templated ligation attempts performed in two steps and vesicle production in the presence of EDC and sulfo-NHS

For the first attempts (from D1 to D4 experiments), the templated ligations were performed through two separate steps:

Cyclisation step: 10 equivalents of EDC (1 mM) were added to 1 equivalent (100 µM) of the oligonucleotide able to form cyclic phosphate (4-mer alone or 4-mer + 5-mer) in 1 mL [4]. When both 4-mer and 5-mer were used, a 9-mer (0.4 (D1 experiment) or 0.2 equivalents (D2, D3 and D4 experiments)) was also added to allow the templated ligation to occur. The pH was adjusted to 5.5 adding 100 mM HCl and the cyclisation reaction was left for 4 h at room temperature. After that, 200 µL were sampled and frozen at –80°C for further analysis. For the cyclisation test performed on 4Rp alone the experiment was stopped here.

Reaction step: The pH value was brought to 6.0 and the sample was left for 64 h at 5°C for the ligation attempts. After that, 200 µL were sampled and frozen at –80°C for further analyses.

Several modifications were brought depending on the experiments:
— In the D2 experiment, several incubations at 5°C were performed, separated by 30 min at 60°C under argon flow in order to evaporate the solvent and to concentrate twice the sample (between each incubation).
— In order to avoid the formation of the of EDC-derivative of the fluorescein tag on the 5-mers, 1.1 equivalent (1.1 mM) sulfo-NHS was added at the beginning of the reaction (D3) or 1 equivalent (1 mM) was added after the cyclization step (D4).
— In order to favour the hybridisation between the 9-mer and the other oligonucleotides, 1.0 M NaCl was added after the first incubation (D3) or 100 mM were used from the start of the experiment (D4).

The same HPLC method was used for all the following LC-MS analyses (4)2) and 4)3)). A *Luna Omega Polar*™ C18 column (150×3.0 mm, 5 µm, *Phenomenex*®) was used. The oligonucleotides were separated at 0.6 mL/min using a binary eluant gradient composed of A) 5 mM NH_4OAc pH 8.0 and B) CH_3CN/A 95:5 from 1 to 21 % B in 20 min.

As a test, a cyclisation experiment was performed on 4Rp alone (only the cyclisation step was done). The result was analysed using LC-MS (Fig. S30).

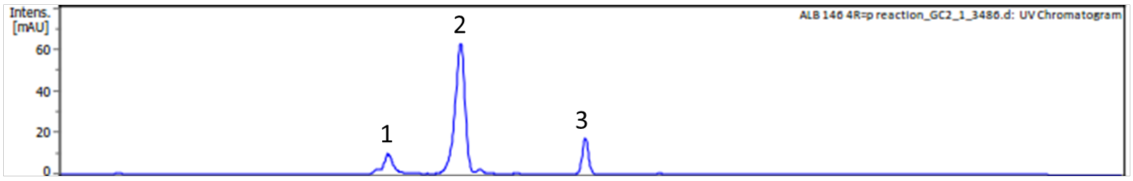

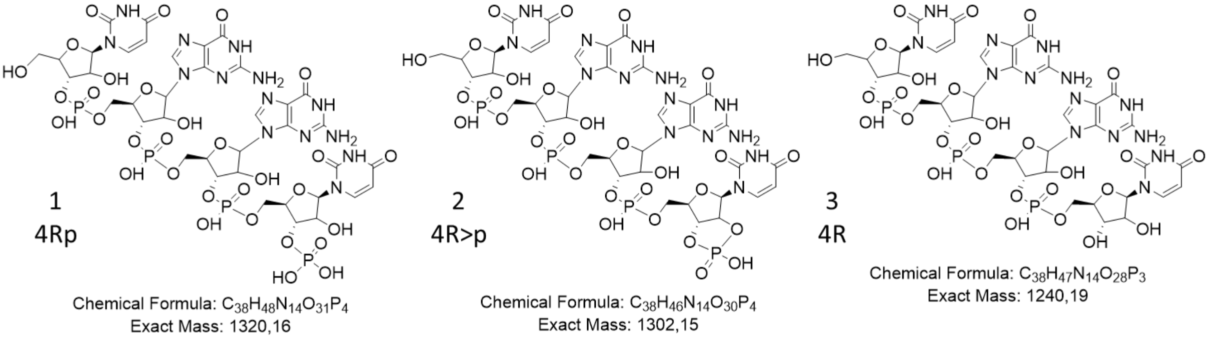

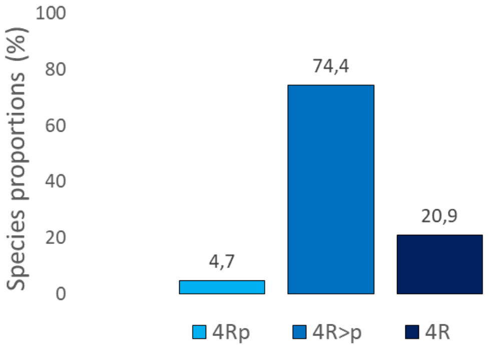

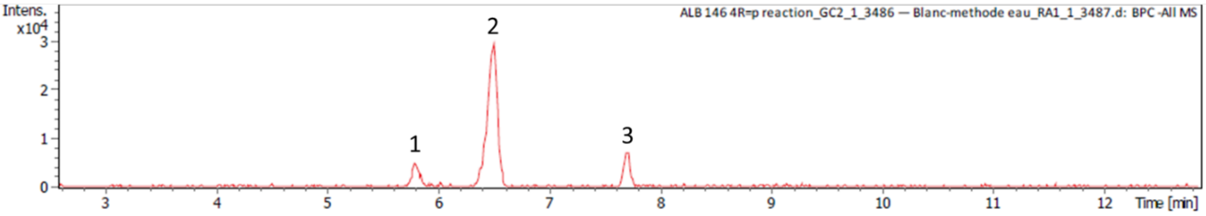

- Composé 1 - Tr = 5.8 min

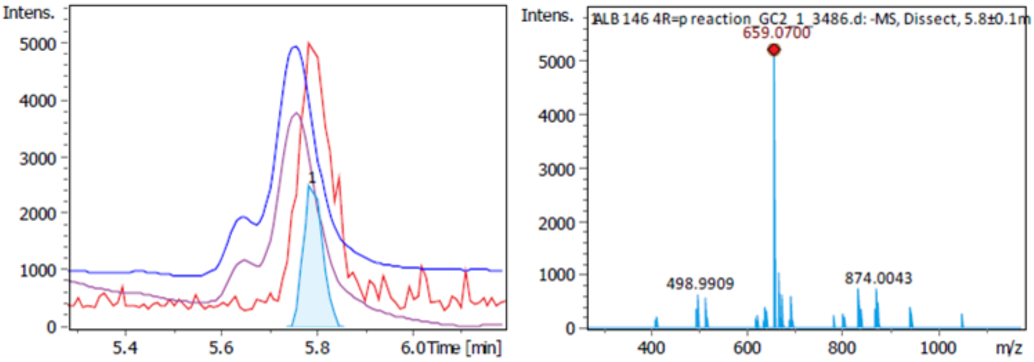

Meas. m/z	Ion Formula	Sum Formula	m/z	err [ppm]	mSigma	Adduct	z
659.07	C38H46N14O31P4	C38H48N14O31P4	659.0707	1.1	45.3	M-H	2-

→ Le spectre de masse met en évidence la présence des adduits correspondant à un produit dont la formule brute est la suivante : $\mathbf{C_{38}H_{48}N_{14}O_{31}P_4}$.

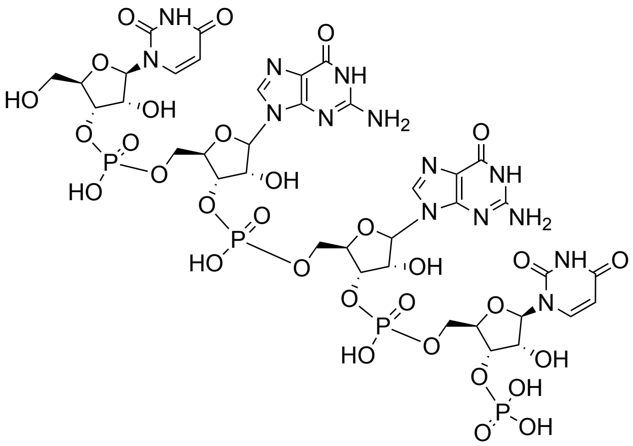

Chemical Formula: $C_{38}H_{48}N_{14}O_{31}P_4$
Exact Mass: 1320,16

4Rp

- Composé 2 - Tr = 6.5 min

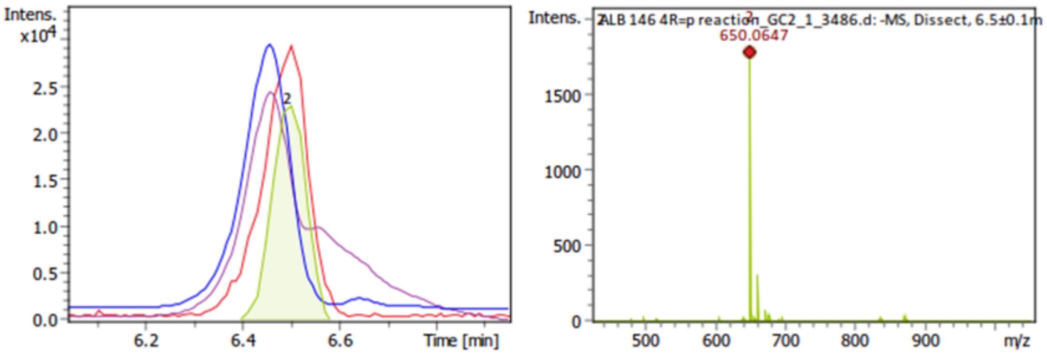

Meas. m/z	Ion Formula	Sum Formula	m/z	err [ppm]	mSigma	Adduct	z
650.0647	C38H44N14O30P4	C38H46N14O30P4	650.0655	1.2	25.4	M-H	2-

→ Le spectre de masse met en évidence la présence des adduits correspondant à un produit dont la formule brute est la suivante : $\mathbf{C_{38}H_{46}N_{14}O_{30}P_4}$.

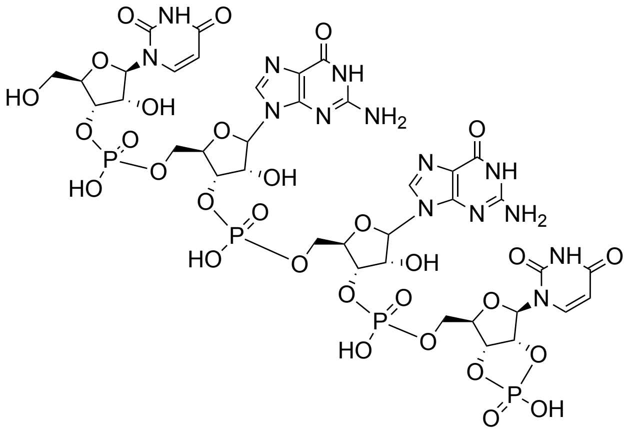

Chemical Formula: $C_{38}H_{46}N_{14}O_{30}P_4$
Exact Mass: 1302,15

4R>p

- Composé 3 - Tr = 7.7 min

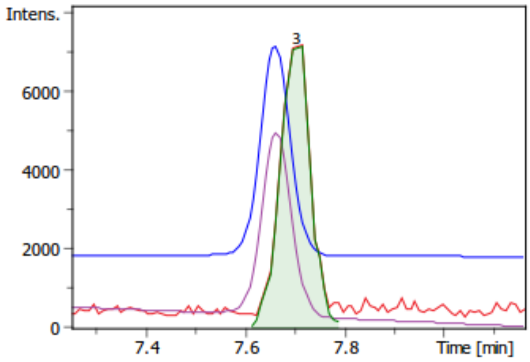

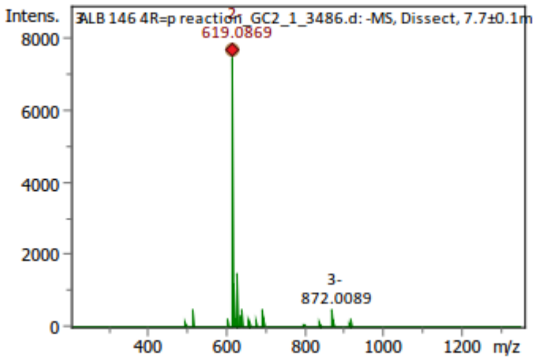

Meas. m/z	Ion Formula	Sum Formula	m/z	err [ppm]	mSigma	Adduct	z
619.0869	C38H45N14O28P3	C38H47N14O28P3	619.0876	1	21.3	M-H	2-

→ Le spectre de masse met en évidence la présence des adduits correspondant à un produit dont la formule brute est la suivante : **$C_{38}H_{47}N_{14}O_{28}P_3$**.

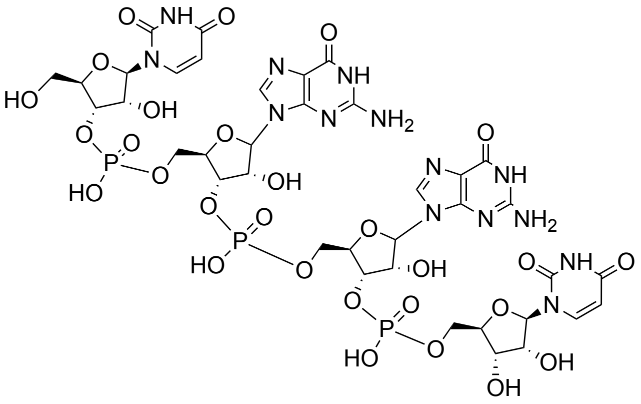

Chemical Formula: $C_{38}H_{47}N_{14}O_{28}P_3$
Exact Mass: 1240,19

4R

Figure S30. Results of the phosphate-cyclization experiment performed on 4Rp. UV chromatogram (260 nm) obtained from LC-MS analysis (negative mode) of the reaction products (RP elution with 5 mM NH_4OAc pH 8.0/CH_3CN:A 95:5) is shown with the compounds associated to the 3 main peaks (UV areas of the peaks were integrated to determine the proportions of the species present) characterized using LC-MS.

Subsequently, several experiments were performed with 4Rp, 5Rp and 9R (from D1 to D4 experiments) and the results were analysed using LC-MS as previously. Of note, out of all EDC derivatives found the structure of only one of several possible isomers is shown (Figs. S31-S37).

D1 experiment:

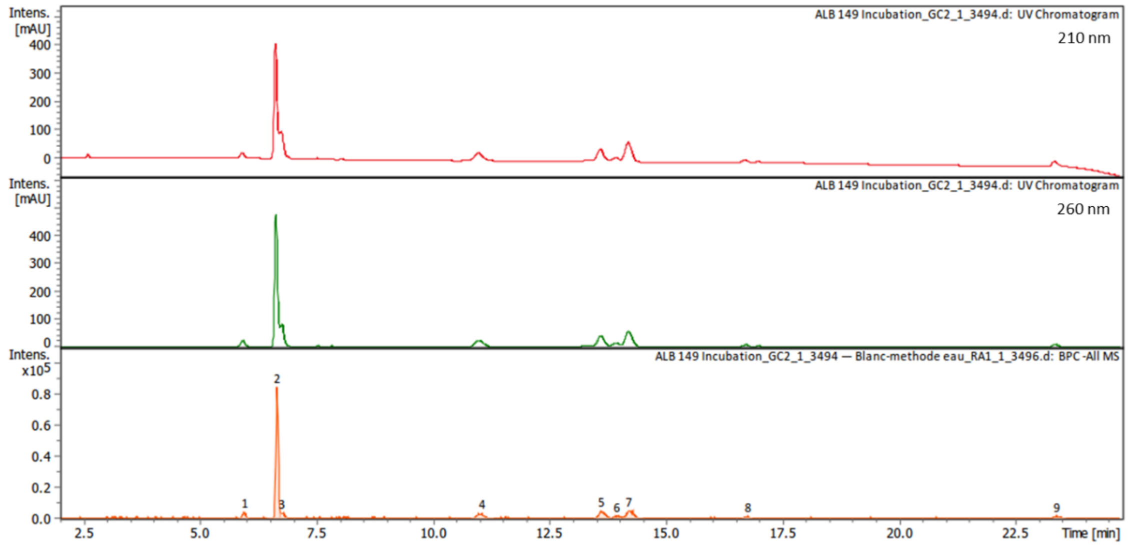

- Composé 1 - Tr = 6.0 min

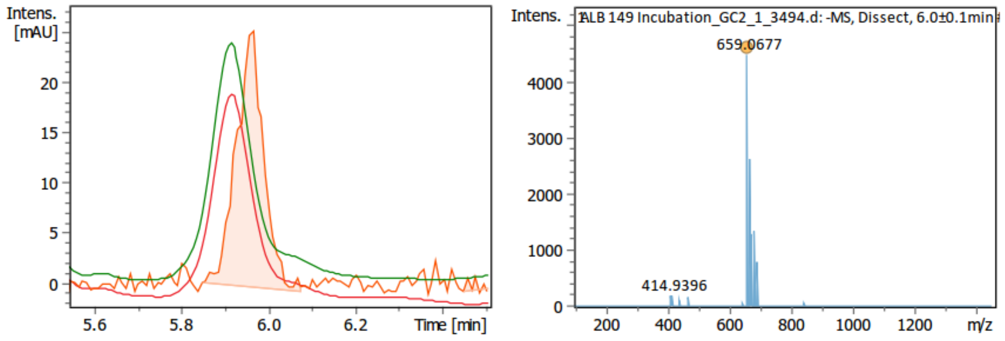

Meas. m/z	Ion Formula	Sum Formula	m/z	err [ppm]	mSigma	Adduct	z
659.0677	C38H46N14O31P4	C38H48N14O31P4	659.0707	4.6	57.2	M-H	2-

→ Le spectre de masse met en évidence la présence des adduits correspondant à un produit dont la formule brute est la suivante : **$C_{38}H_{48}N_{14}O_{31}P_4$**.

4Rp

- Composé 2 - Tr = 6.7 min

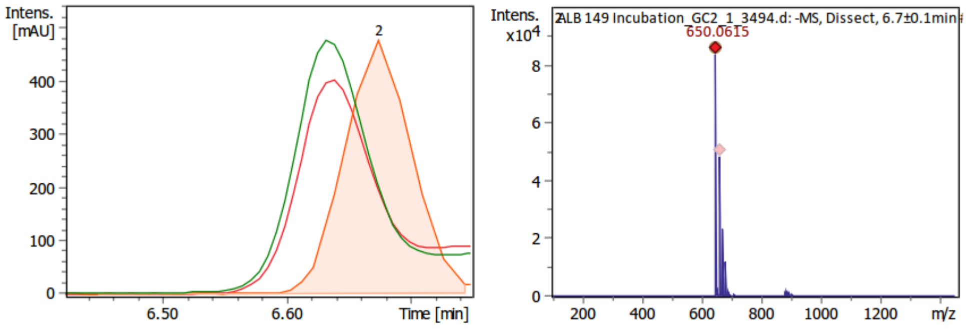

Meas. m/z	Ion Formula	Sum Formula	m/z	err [ppm]	mSigma	Adduct	z
650.0615	C38H44N14O30P4	C38H46N14O30P4	650.0655	6.1	18.5	M-H	2-

→ Le spectre de masse met en évidence la présence des adduits correspondant à un produit dont la formule brute est la suivante : **$C_{38}H_{46}N_{14}O_{30}P_4$**.

4R>p

- Composé 3 - Tr = 6.8 min

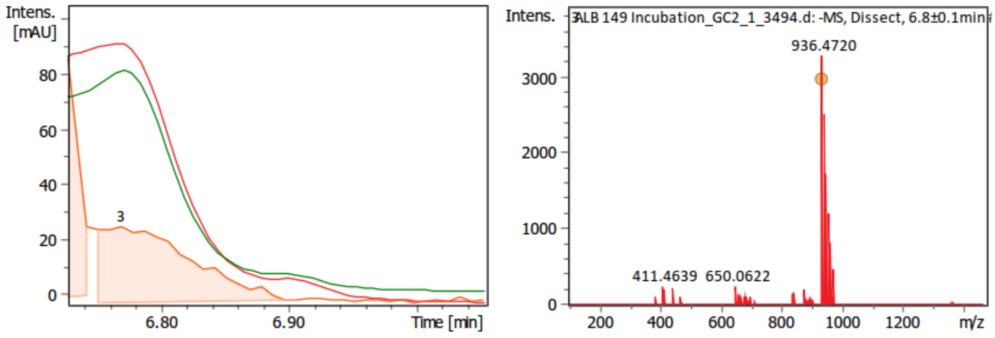

Meas. m/z	Ion Formula	Sum Formula	m/z	err [ppm]	mSigma	Adduct	z
936.1378	C85H106N35O59P8	C85H109N35O59P8	936.1429	5.5	22.5	M-H	3-

→ Le spectre de masse met en évidence la présence des adduits correspondant à un produit dont la formule brute est la suivante : **$C_{85}H_{109}N_{35}O_{59}P_8$**.

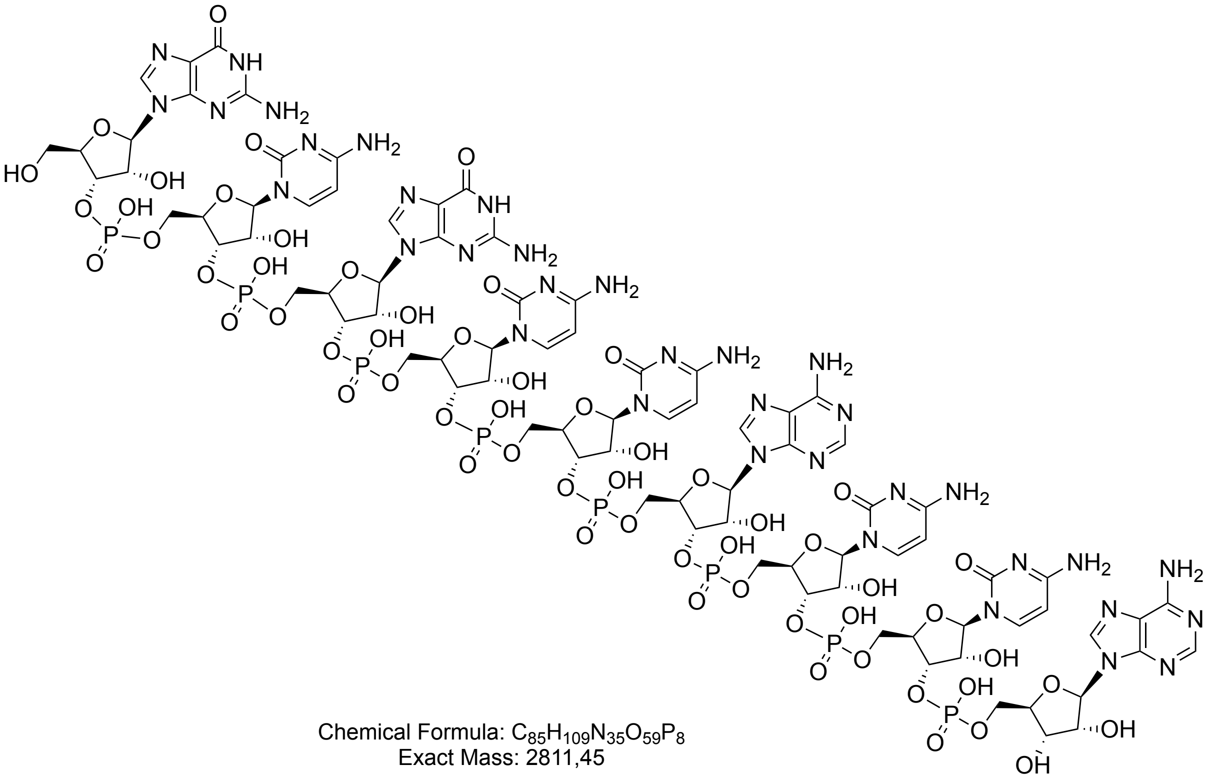

Chemical Formula: $C_{85}H_{109}N_{35}O_{59}P_8$
Exact Mass: 2811,45

- Composé 4 - Tr = 11.0 min

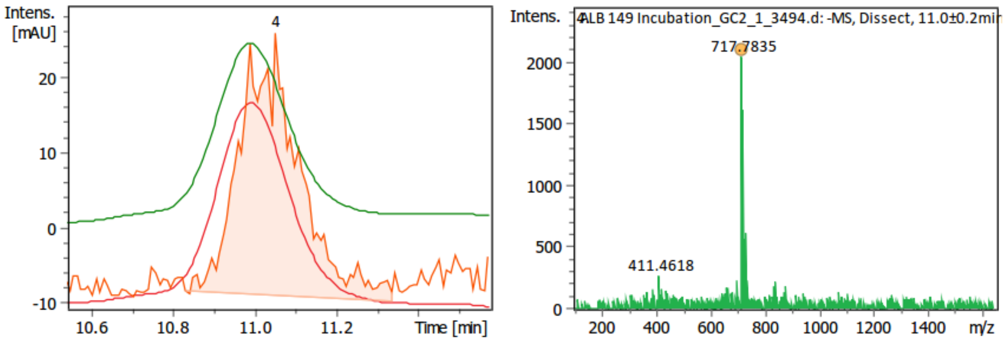

Meas. m/z	Ion Formula	Sum Formula	m/z	err [ppm]	mSigma	Adduct	z
717.7835	C78H82N22O42P5	C78H85N22O42P5	717.7887	7.3	16.7	M-H	3-

→ Le spectre de masse met en évidence la présence des adduits correspondant à un produit dont la formule brute est la suivante : **$C_{78}H_{85}N_{22}O_{42}P_5$**.

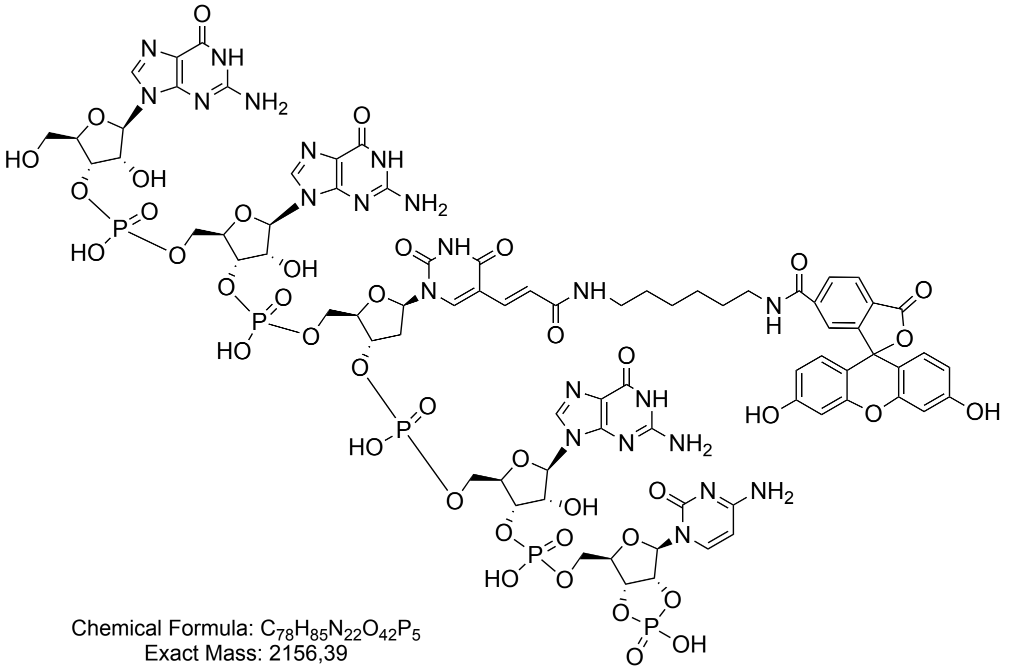

5R>p

- Composé 5 - Tr = 13.7 min

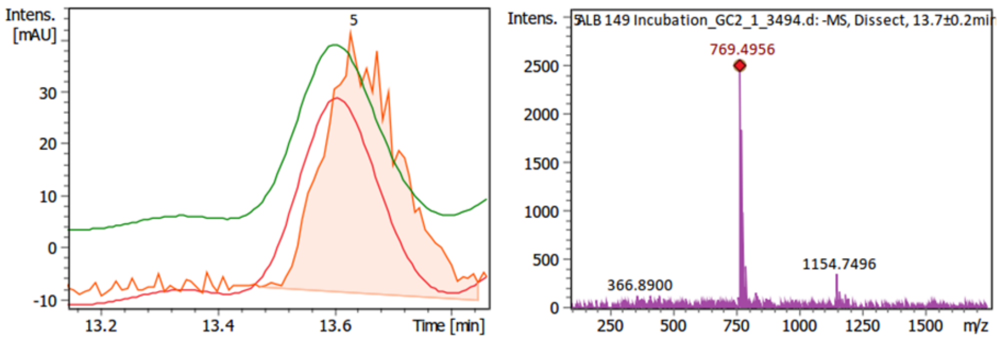

Meas. m/z	Ion Formula	Sum Formula	m/z	err [ppm]	mSigma	Adduct	z
769.4956	C86H99N25O42P5	C86H102N25O42P5	769.5028	9.3	15.8	M-H	3-

→ Le spectre de masse met en évidence la présence des adduits correspondant à un produit dont la formule brute est la suivante : **$C_{86}H_{102}N_{25}O_{42}P_5$**.

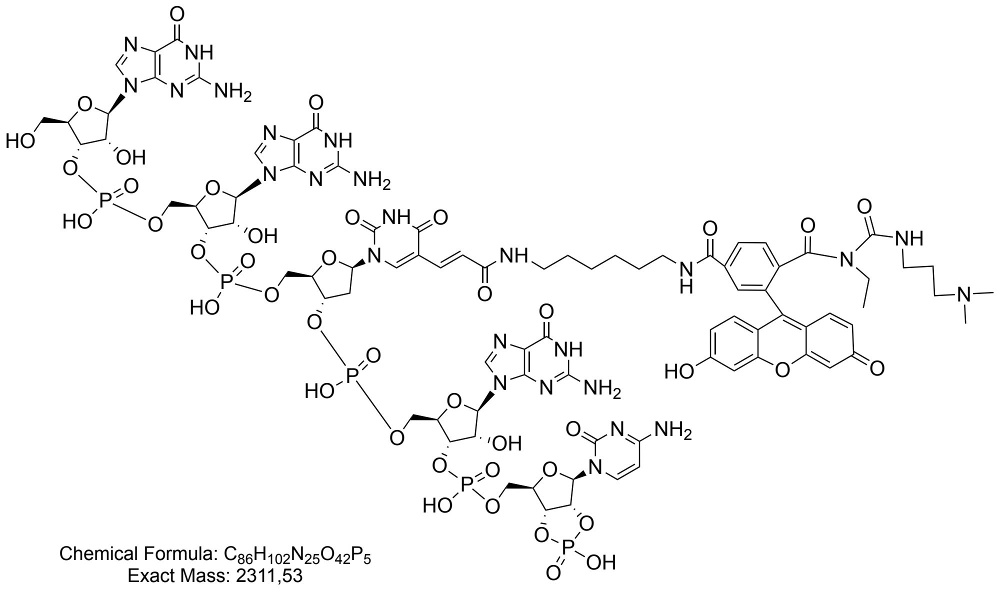

Chemical Formula: $C_{86}H_{102}N_{25}O_{42}P_5$
Exact Mass: 2311,53

5R>p-EDC

- Composé 6 - Tr = 14.0 min

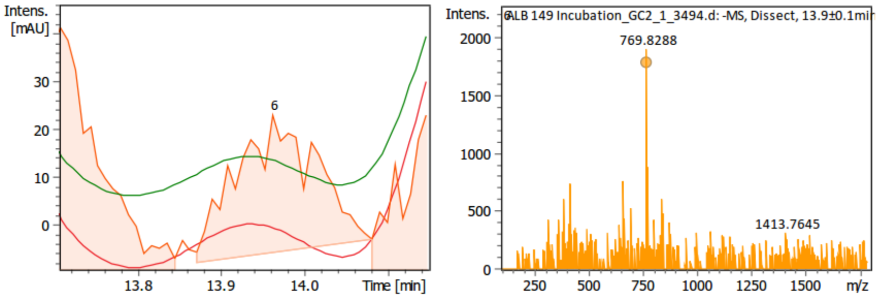

Meas. m/z	Ion Formula	Sum Formula	m/z	err [ppm]	mSigma	Adduct	z
769.4949	C86H99N25O42P5	C86H102N25O42P5	769.5028	10.2	180.9	M-H	3-

→ Le spectre de masse met en évidence la présence des adduits correspondant à un produit dont la formule brute est la suivante : **$C_{86}H_{102}N_{25}O_{42}P_5$**.

5R>p-EDC

- Composé 7 - Tr = 14.2 min

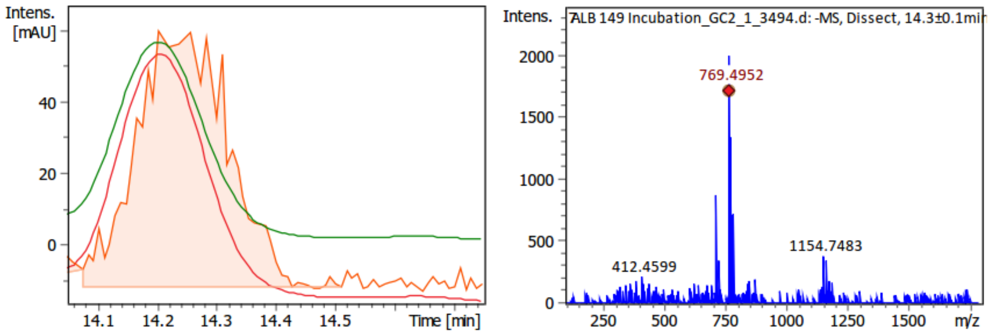

Meas. m/z	Ion Formula	Sum Formula	m/z	err [ppm]	mSigma	Adduct	z
769.4952	C86H99N25O42P5	C86H102N25O42P5	769.5028	9.9	56.1	M-H	3-

→ Le spectre de masse met en évidence la présence des adduits correspondant à un produit dont la formule brute est la suivante : **$C_{86}H_{102}N_{25}O_{42}P_5$**.

5R>p-EDC

- Composé 8 - Tr = 16.8 min

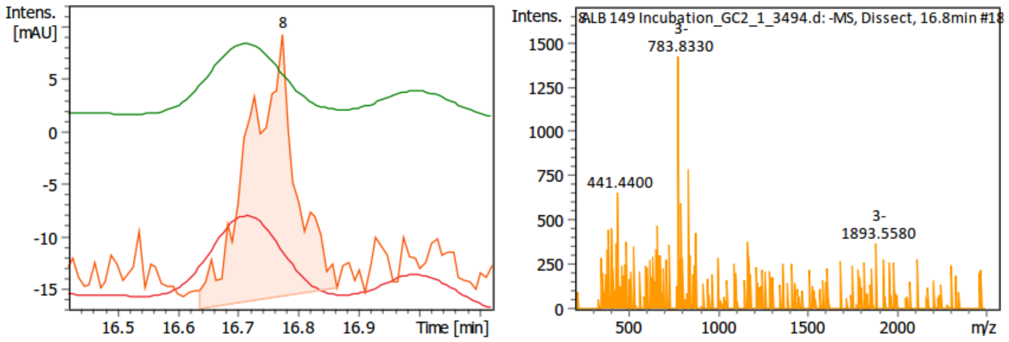

Soit M=783.8330*3+3= 2354.5Da

Unidentified derivative

- Composé 9 - Tr = 23.4 min

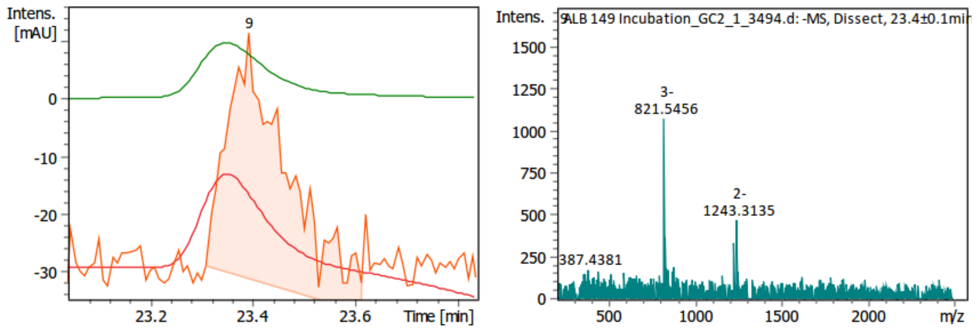

Soit M=821.5456*3+3=2467.6Da

Unidentified derivative

Figure S31. LC-MS chromatogram (negative ion mode), UV chromatograms (260 and 210 nm), peaks and associated compounds obtained for the D1 experiment.

D2.1 experiment:

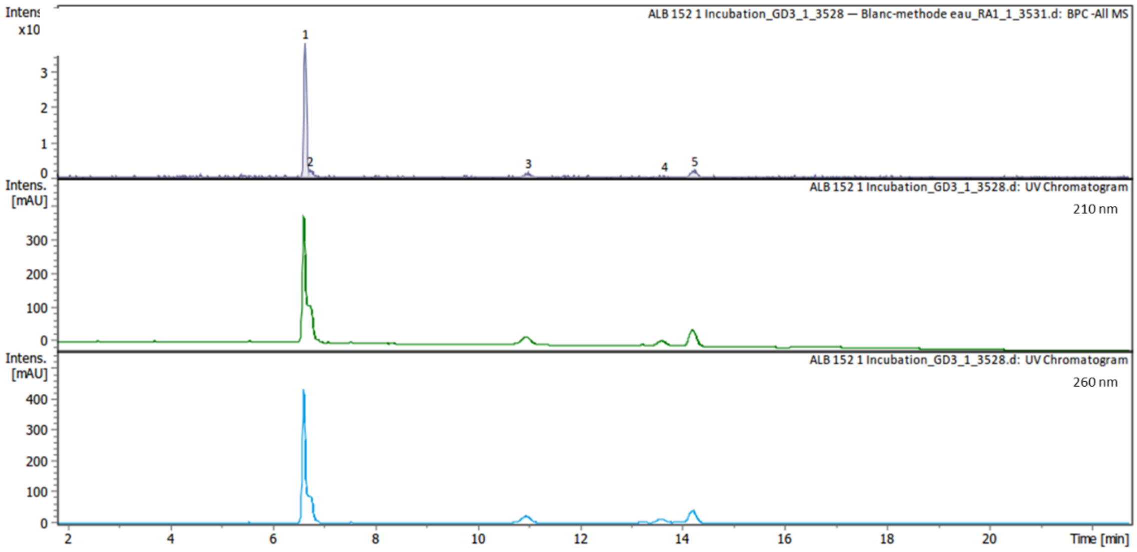

- Composé 1 - Tr = 6.7 min

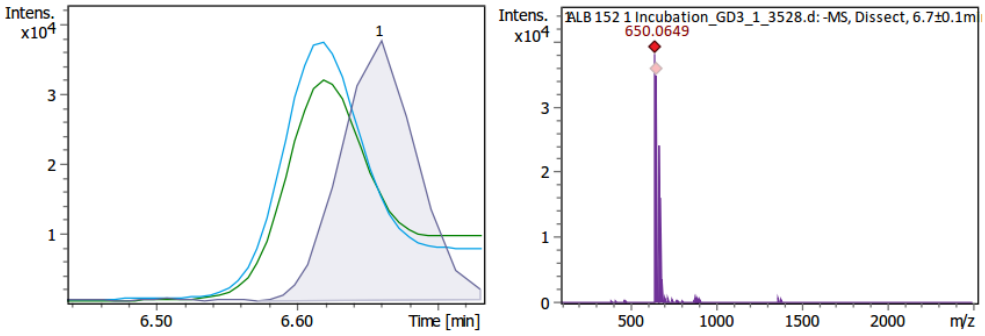

Meas. m/z	Ion Formula	Sum Formula	m/z	err [ppm]	mSigma	Adduct	z
650.0649	C38H44N14O30P4	C38H46N14O30P4	650.0655	0.8	8.7	M-H	2-

→ Le spectre de masse met en évidence la présence des adduits correspondant à un produit dont la formule brute est la suivante : $\mathbf{C_{38}H_{46}N_{14}O_{30}P_4}$.

4Rp

- Composé 2 - Tr = 6.7 min

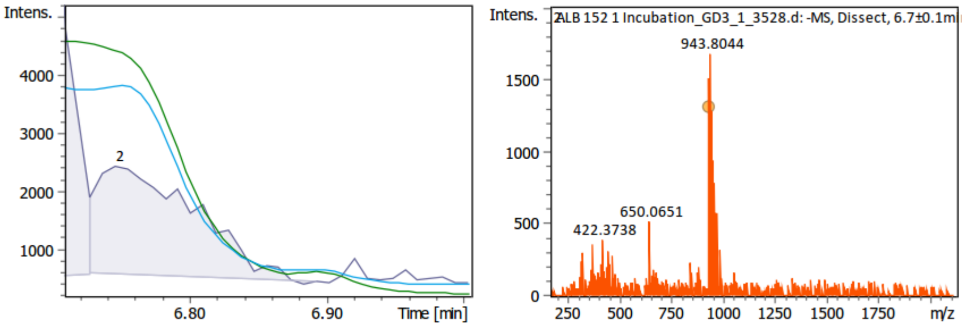

Meas. m/z	Ion Formula	Sum Formula	m/z	err [ppm]	mSigma	Adduct	z
936.1423	C85H106N35O59P8	C85H109N35O59P8	936.1429	0.7	39.6	M-H	3-

→ Le spectre de masse met en évidence la présence des adduits correspondant à un produit dont la formule brute est la suivante : $\mathbf{C_{85}H_{109}N_{35}O_{59}P_8}$.

9R

- Composé 3 - Tr = 11.0 min

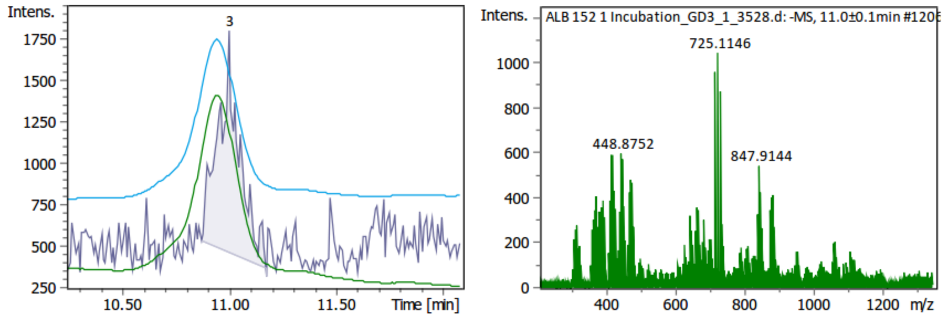

Meas. m/z	Ion Formula	Sum Formula	m/z	err [ppm]	mSigma	Adduct	z
717.7876	C78H82N22O42P5	C78H85N22O42P5	717.7887	1.6	40.6	M-H	3-

→ Le spectre de masse met en évidence la présence des adduits correspondant à un produit dont la formule brute est la suivante : $\mathbf{C_{78}H_{85}N_{22}O_{42}P_5}$.

5R>p

- Composé 4 - Tr = 13.6 min

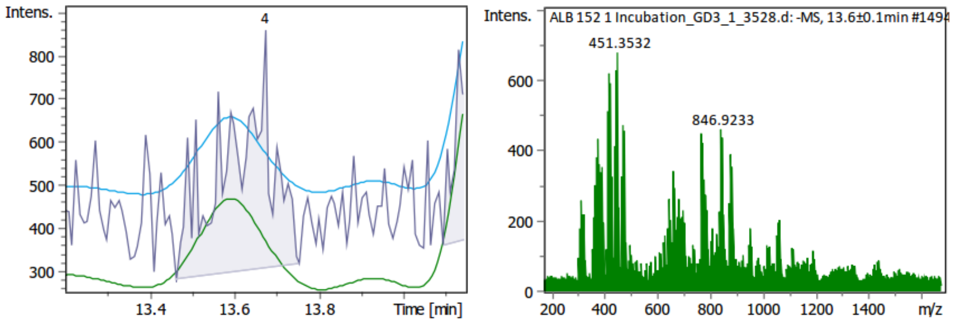

Meas. m/z	Ion Formula	Sum Formula	m/z	err [ppm]	mSigma	Adduct	z
769.5002	C86H99N25O42P5	C86H102N25O42P5	769.5028	3.4	71.6	M-H	3-

→ Le spectre de masse met en évidence la présence des adduits correspondant à un produit dont la formule brute est la suivante : $\mathbf{C_{86}H_{102}N_{25}O_{42}P_5}$.

5R>p-EDC

- Composé 5 - Tr = 14.2 min

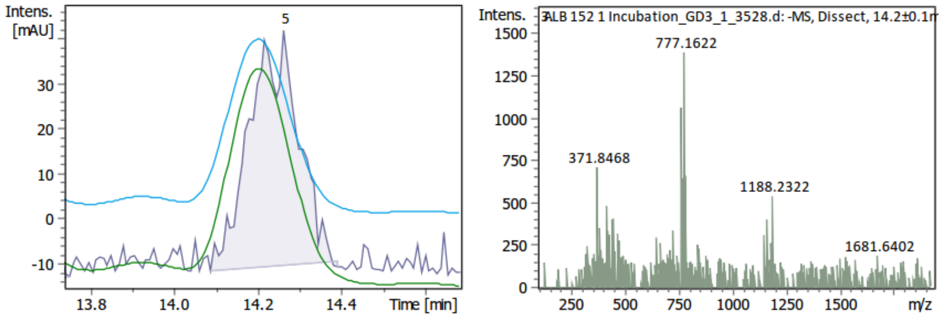

Meas. m/z	Ion Formula	Sum Formula	m/z	err [ppm]	mSigma	Adduct	z
769.5006	C86H99N25O42P5	C86H102N25O42P5	769.5028	2.9	58.9	M-H	3-

→ Le spectre de masse met en évidence la présence des adduits correspondant à un produit dont la formule brute est la suivante : $\mathbf{C_{86}H_{102}N_{25}O_{42}P_5}$.

5R>p-EDC

Figure S32. LC-MS chromatogram (negative mode), UV chromatograms (260 and 210 nm), peaks and associated compounds obtained for the D2.1 experiment.

D2.2 experiment:

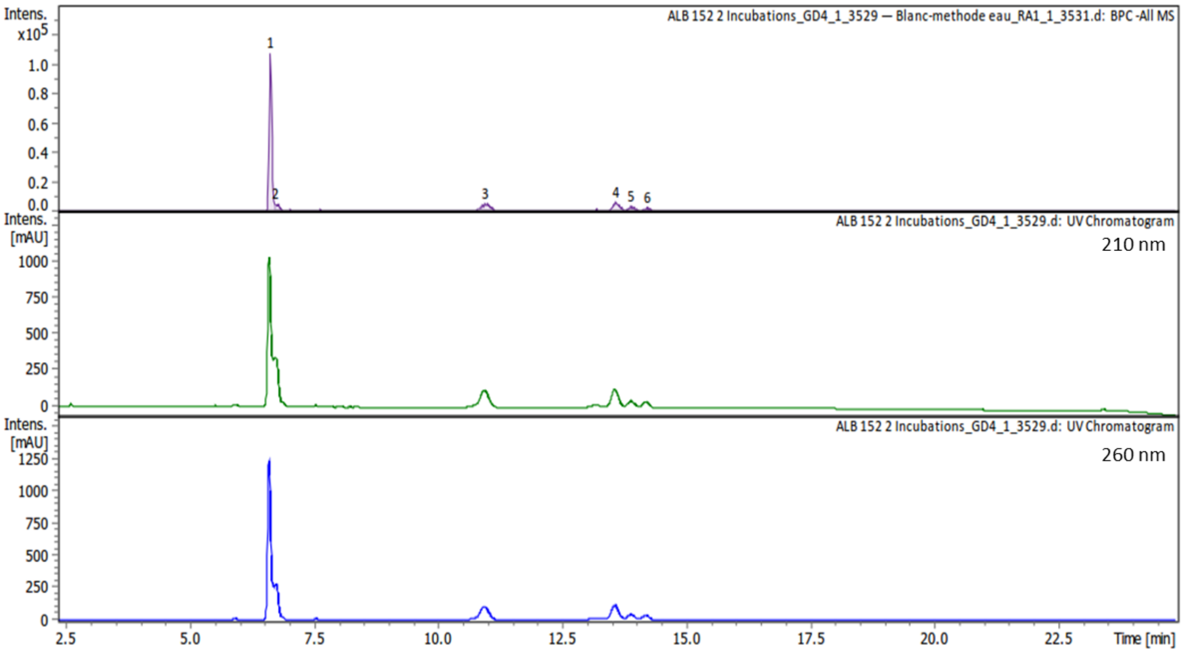

▪ Composé 1 - Tr = 6.6 min

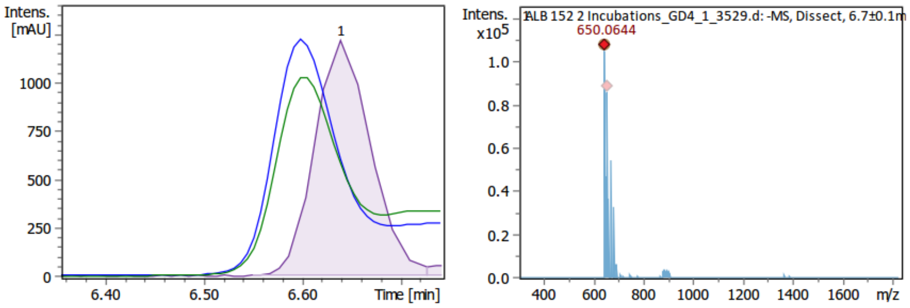

Meas. m/z	Ion Formula	Sum Formula	m/z	err [ppm]	mSigma	Adduct	z
650.0644	C38H44N14O30P4	C38H46N14O30P4	650.0655	1.6	21	M-H	2-

→ Le spectre de masse met en évidence la présence des adduits correspondant à un produit dont la formule brute est la suivante : **$C_{38}H_{46}N_{14}O_{30}P_4$**.

4R>p

▪ Composé 2 - Tr = 6.7 min

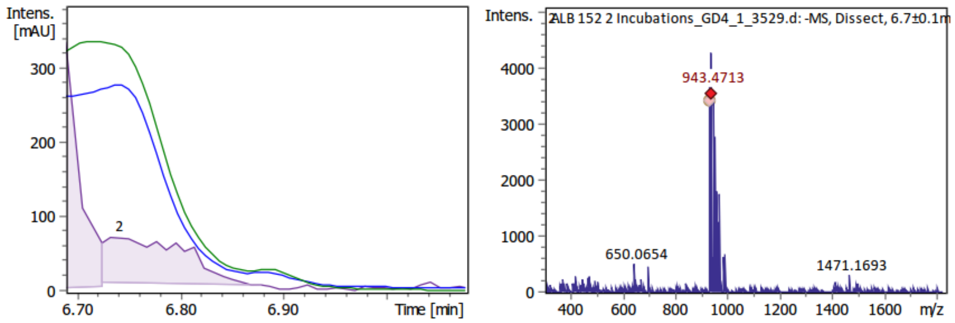

Meas. m/z	Ion Formula	Sum Formula	m/z	err [ppm]	mSigma	Adduct	z
936.1449	C85H106N35O59P8	C85H109N35O59P8	936.1429	-2.1	25.6	M-H	3-

→ Le spectre de masse met en évidence la présence des adduits correspondant à un produit dont la formule brute est la suivante : **$C_{85}H_{109}N_{35}O_{59}P_8$**.

9R

- Composé 3 - Tr = 11.0 min

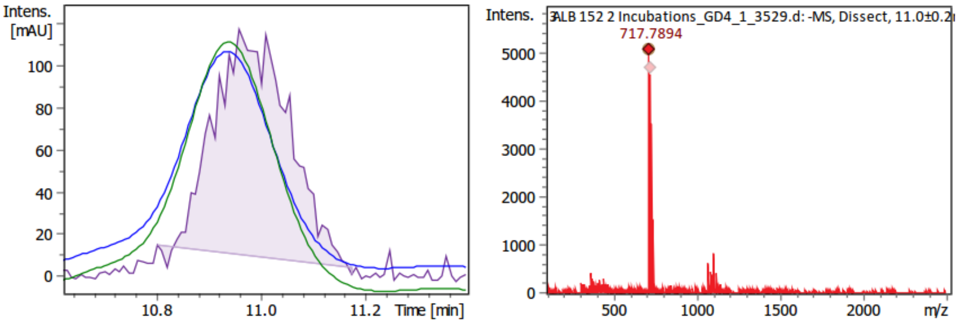

Meas. m/z	Ion Formula	Sum Formula	m/z	err [ppm]	mSigma	Adduct	z
717.7894	C78H82N22O42P5	C78H85N22O42P5	717.7887	-0.9	23.9	M-H	3-

→ Le spectre de masse met en évidence la présence des adduits correspondant à un produit dont la formule brute est la suivante : **$C_{78}H_{85}N_{22}O_{42}P_5$**.

5R>p

- Composé 4 - Tr = 13.2 min

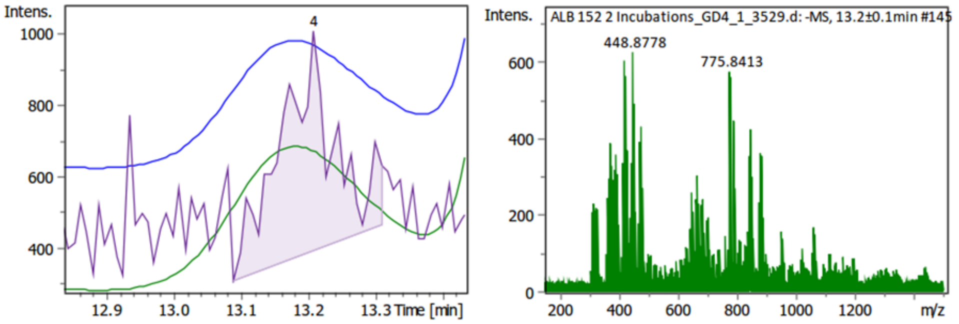

Meas. m/z	Ion Formula	Sum Formula	m/z	err [ppm]	mSigma	Adduct	z
775.5075	C86H101N25O43P5	C86H104N25O43P5	775.5063	-1.5	64.1	M-H	3-

→ Le spectre de masse met en évidence la présence des adduits correspondant à un produit dont la formule brute est la suivante : **$C_{86}H_{104}N_{25}O_{43}P_5$**.

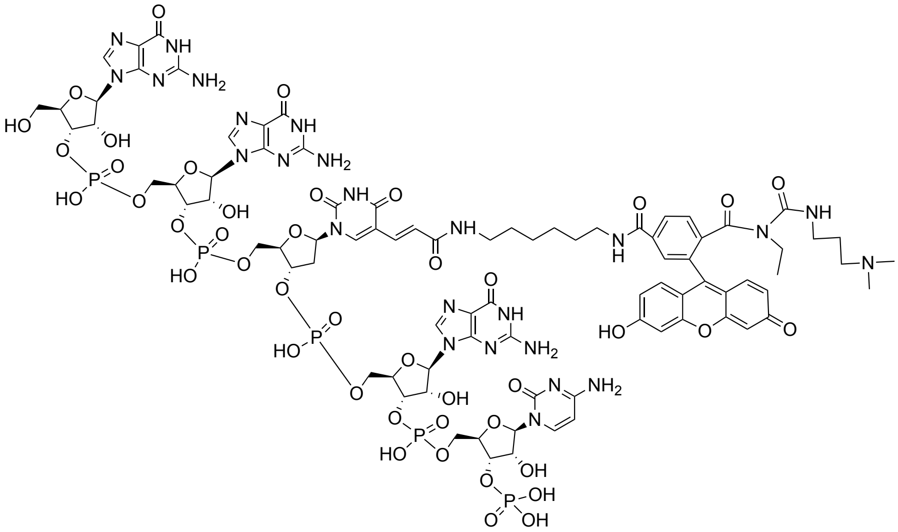

Chemical Formula: $C_{86}H_{104}N_{25}O_{43}P_5$
Exact Mass: 2329,54

5Rp-EDC

- Composé 5 - Tr = 13.6 min

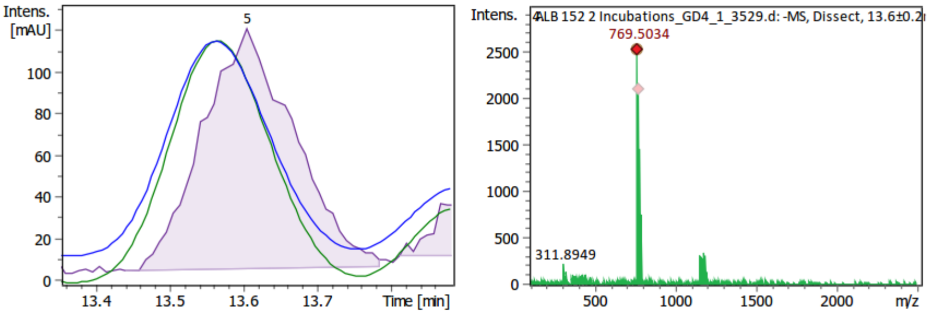

Meas. m/z	Ion Formula	Sum Formula	m/z	err [ppm]	mSigma	Adduct	z
769.5034	C86H99N25O42P5	C86H102N25O42P5	769.5028	-0.7	30.5	M-H	3-

→ Le spectre de masse met en évidence la présence des adduits correspondant à un produit dont la formule brute est la suivante : $\mathbf{C_{86}H_{102}N_{25}O_{42}P_{5}}$.

5R>p-EDC

- Composé 6 - Tr = 13.9 min

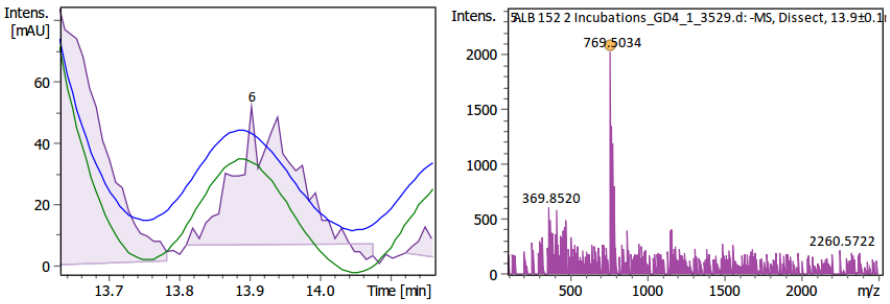

Meas. m/z	Ion Formula	Sum Formula	m/z	err [ppm]	mSigma	Adduct	z
769.5034	C86H99N25O42P5	C86H102N25O42P5	769.5028	-0.7	36	M-H	3-

→ Le spectre de masse met en évidence la présence des adduits correspondant à un produit dont la formule brute est la suivante : $\mathbf{C_{86}H_{102}N_{25}O_{42}P_{5}}$.

5R>p-EDC

Figure S33. LC-MS chromatogram (negative ion mode), UV chromatograms (260 and 210 nm), peaks and associated compounds obtained for the D2.2 experiment.

D2.3 experiment:

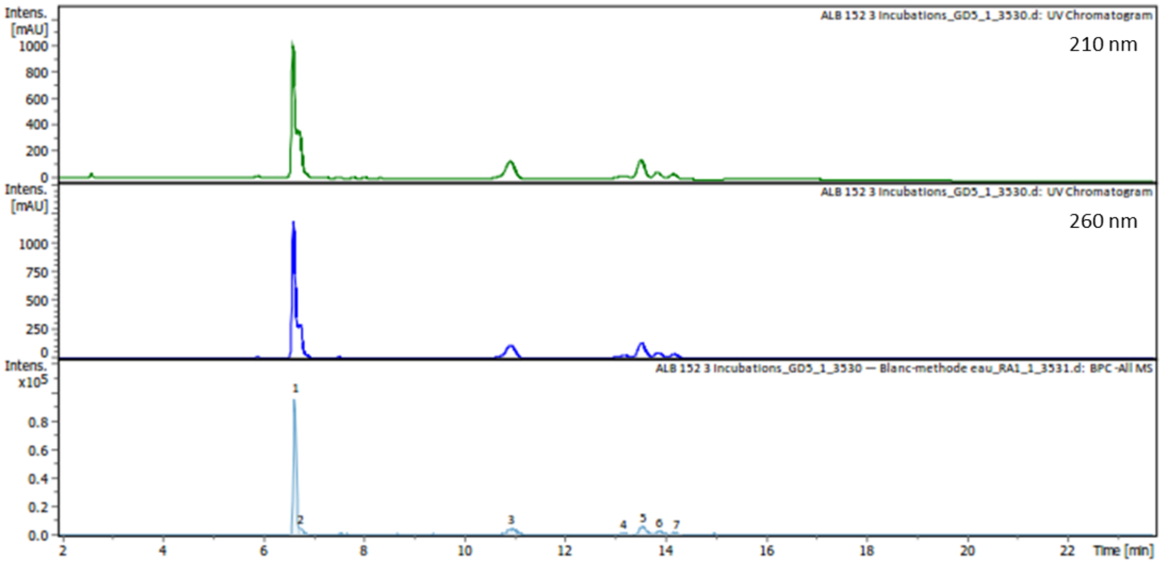

- Composé 1 - Tr = 6.6 min

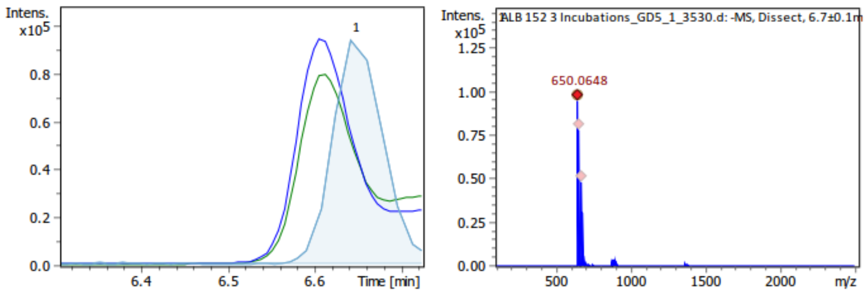

Meas. m/z	Ion Formula	Sum Formula	m/z	err [ppm]	mSigma	Adduct	z
650.0648	C38H44N14O30P4	C38H46N14O30P4	650.0655	1	25.7	M-H	2-

→ Le spectre de masse met en évidence la présence des adduits correspondant à un produit dont la formule brute est la suivante : **$C_{38}H_{46}N_{14}O_{30}P_4$**.

4R>p-EDC

- Composé 2 - Tr = 6.8 min

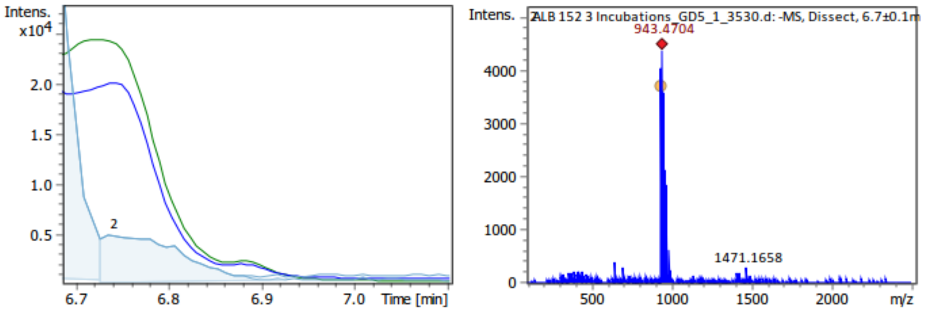

Meas. m/z	Ion Formula	Sum Formula	m/z	err [ppm]	mSigma	Adduct	z
936.1436	C85H106N35O59P8	C85H109N35O59P8	936.1429	-0.7	26.3	M-H	3-

→ Le spectre de masse met en évidence la présence des adduits correspondant à un produit dont la formule brute est la suivante : **$C_{85}H_{109}N_{35}O_{59}P_8$**.

- Composé 3 - Tr = 10.9 min

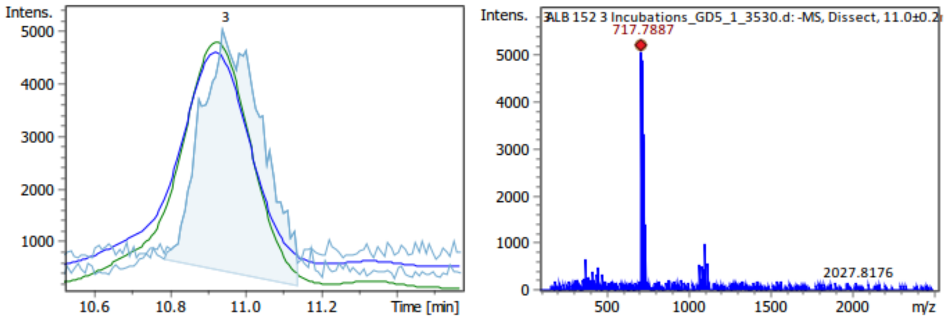

Meas. m/z	Ion Formula	Sum Formula	m/z	err [ppm]	mSigma	Adduct	z
717.7887	C78H82N22O42P5	C78H85N22O42P5	717.7887	0.1	16.7	M-H	3-

→ Le spectre de masse met en évidence la présence des adduits correspondant à un produit dont la formule brute est la suivante : **$C_{78}H_{85}N_{22}O_{42}P_5$**.

5R>p

- Composé 4 - Tr = 13.2 min

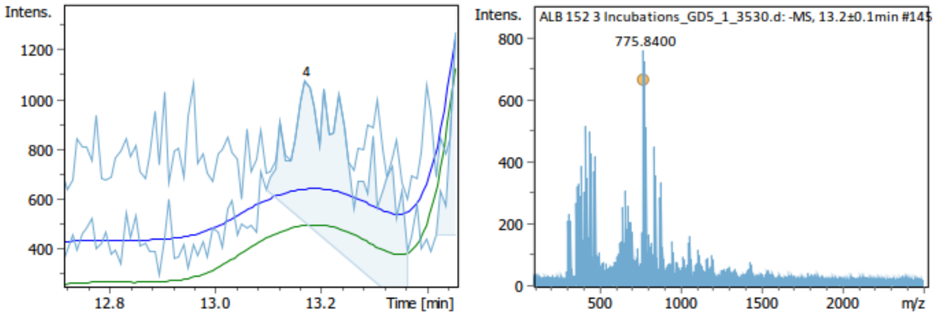

Meas. m/z	Ion Formula	Sum Formula	m/z	err [ppm]	mSigma	Adduct	z
775.5066	C86H101N25O43P5	C86H104N25O43P5	775.5063	-0.3	64.4	M-H	3-

→ Le spectre de masse met en évidence la présence des adduits correspondant à un produit dont la formule brute est la suivante : **$C_{86}H_{104}N_{25}O_{43}P_5$**.

5R-p-EDC

- Composé 5 - Tr = 13.6 min

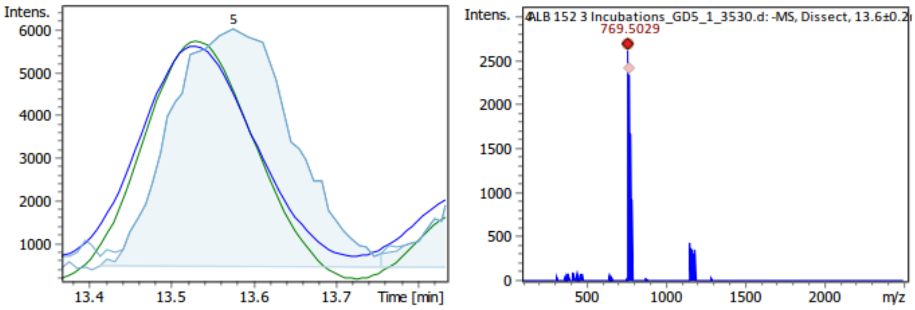

Meas. m/z	Ion Formula	Sum Formula	m/z	err [ppm]	mSigma	Adduct	z
769.5029	C86H99N25O42P5	C86H102N25O42P5	769.5028	-0.1	23.6	M-H	3-

→ Le spectre de masse met en évidence la présence des adduits correspondant à un produit dont la formule brute est la suivante : **$C_{86}H_{102}N_{25}O_{42}P_5$**.

5R>p-EDC

- Composé 6 - Tr = 13.9 min

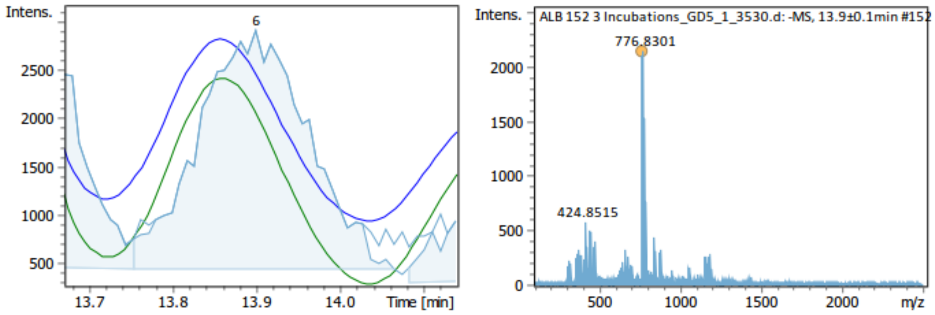

Meas. m/z	Ion Formula	Sum Formula	m/z	err [ppm]	mSigma	Adduct	z
769.5026	C86H99N25O42P5	C86H102N25O42P5	769.5028	0.3	26.7	M-H	3-

→ Le spectre de masse met en évidence la présence des adduits correspondant à un produit dont la formule brute est la suivante : **$C_{86}H_{102}N_{25}O_{42}P_5$**.

5R>p-EDC

- Composé 7 - Tr = 14.2 min

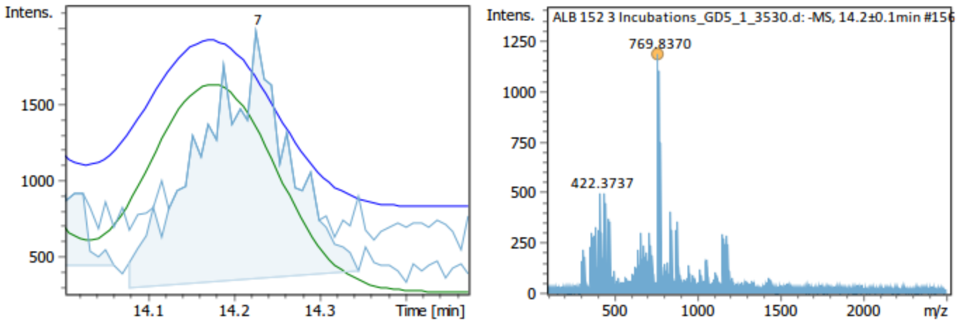

Meas. m/z	Ion Formula	Sum Formula	m/z	err [ppm]	mSigma	Adduct	z
769.5034	C86H99N25O42P5	C86H102N25O42P5	769.5028	-0.7	19.4	M-H	3-

→ Le spectre de masse met en évidence la présence des adduits correspondant à un produit dont la formule brute est la suivante : **$C_{86}H_{102}N_{25}O_{42}P_5$**.

5R>p-EDC

Figure S34. LC-MS chromatogram (negative ion mode), UV chromatograms (260 and 210 nm), peaks and associated compounds obtained for the D2.3 experiment.

A kinetic for the different species (coming from 4Rp and 5Rp) could be determine for the D2 experiments:

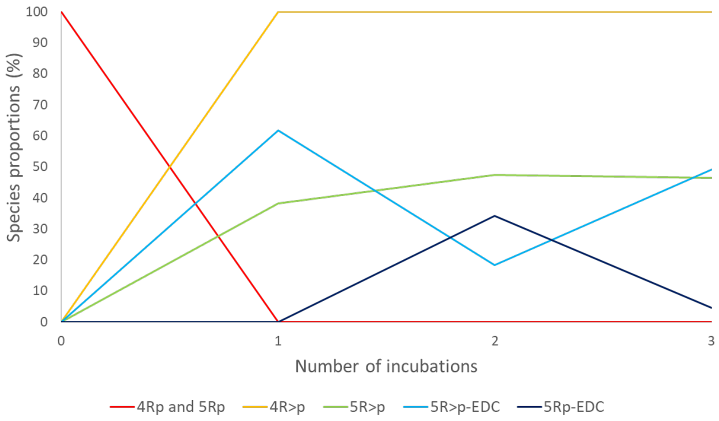

Figure S35. Proportions of the main species (others than 9R) found for D2.1, D2.2 and D2.3 experiments over the number of incubations. These ratios were calculated using the UV absorbance of the corresponding compound peaks detected by LC-MS analyses (RP elution with 5 mM NH_4OAc pH 8.0/ CH_3CN/A 95:5) (Fig. S32-34).

D3 experiment:

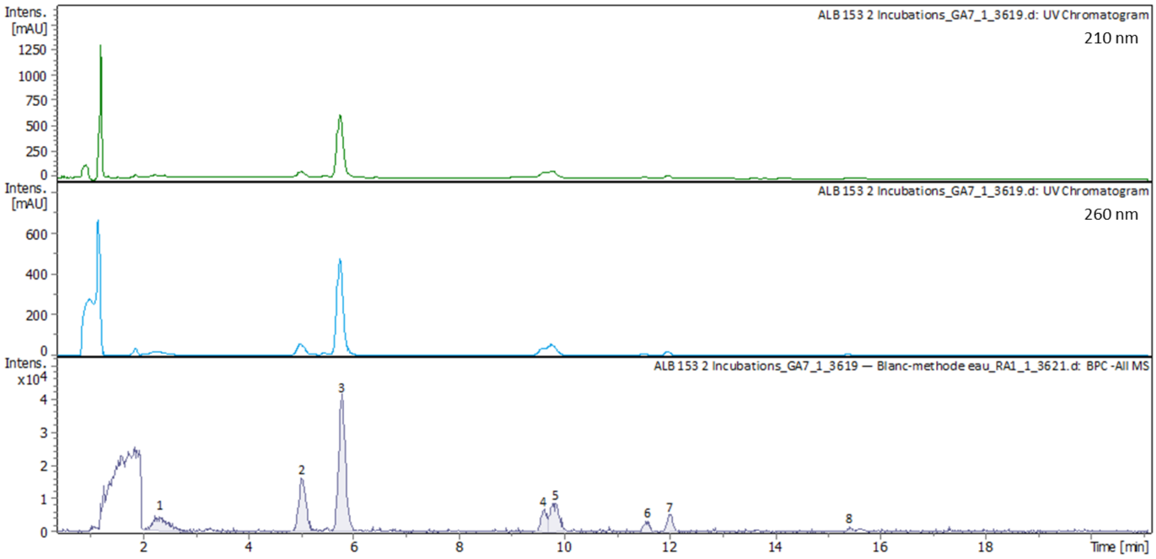

Ion extractions for the 2 possible products of ligation:
9Rfp ($C_{116}H_{133}N_{36}O_{73}P_9$) or 9Rf>p ($C_{116}H_{131}N_{36}O_{72}P_9$)

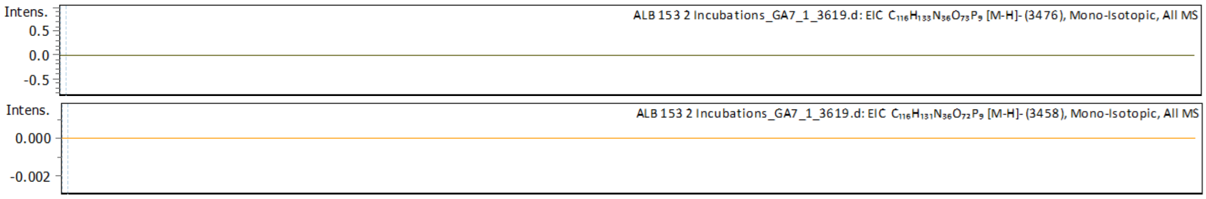

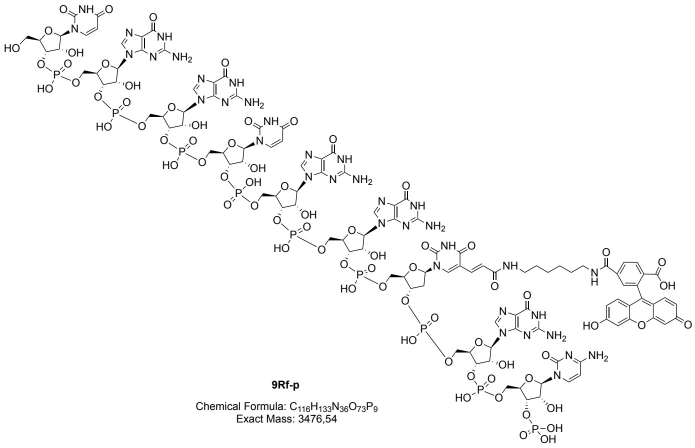

9Rf-p

Chemical Formula: $C_{116}H_{133}N_{36}O_{73}P_9$
Exact Mass: 3476,54

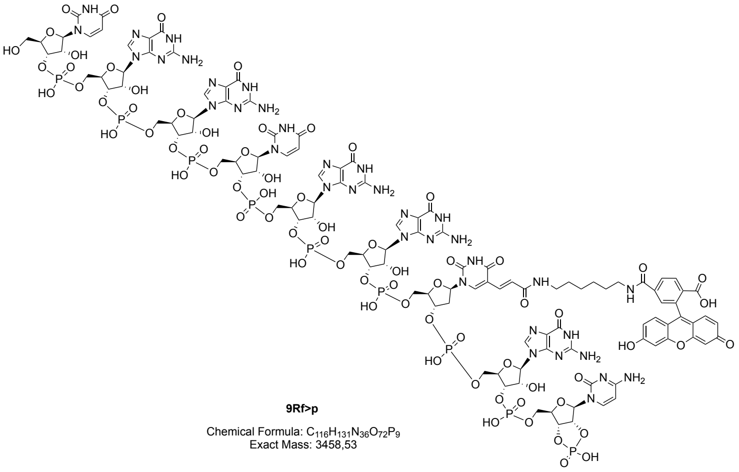

9Rf>p

Chemical Formula: $C_{116}H_{131}N_{36}O_{72}P_9$
Exact Mass: 3458,53

- Composé 1 - Tr = 2.3 min

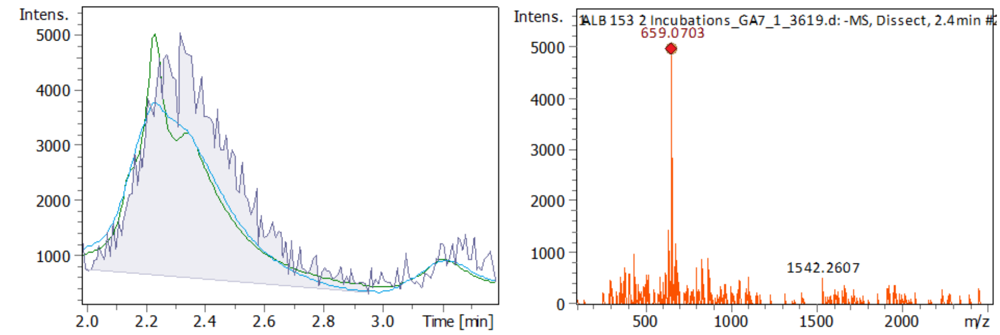

Meas. m/z	Ion Formula	Sum Formula	m/z	err [ppm]	mSigma	Adduct	z
659.0703	C38H46N14O31P4	C38H48N14O31P4	659.0707	0.7	40.1	M-H	2-

→ Le spectre de masse met en évidence la présence des adduits correspondant à un produit dont la formule brute est la suivante : $\mathbf{C_{38}H_{48}N_{14}O_{31}P_4}$.

4Rp

- Composé 2 - Tr = 5.0 min

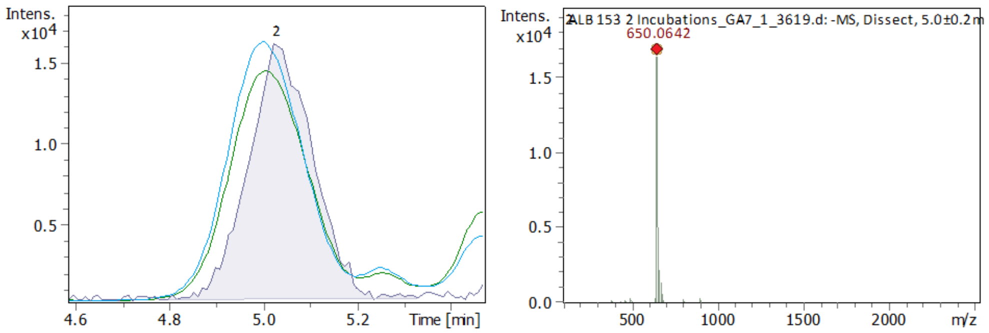

Meas. m/z	Ion Formula	Sum Formula	m/z	err [ppm]	mSigma	Adduct	z
650.0642	C38H44N14O30P4	C38H46N14O30P4	650.0655	2	18.5	M-H	2-

→ Le spectre de masse met en évidence la présence des adduits correspondant à un produit dont la formule brute est la suivante : $\mathbf{C_{38}H_{46}N_{14}O_{30}P_4}$.

4R>p

- Composé 3 - Tr = 5.8 min

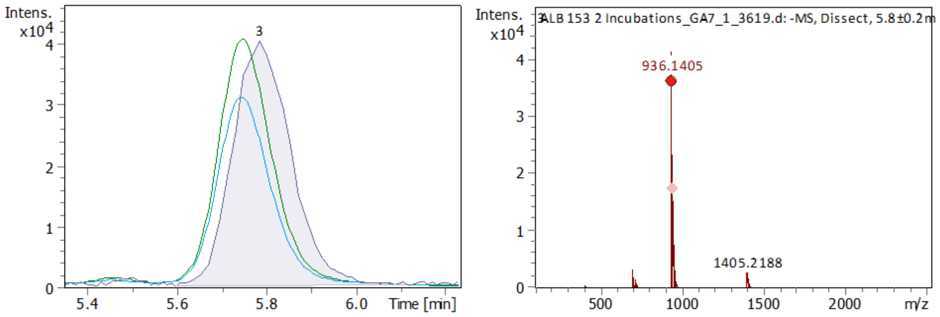

Meas. m/z	Ion Formula	Sum Formula	m/z	err [ppm]	mSigma	Adduct	z
936.1405	C85H106N35O59P8	C85H109N35O59P8	936.1429	2.6	32.1	M-H	3-

→ Le spectre de masse met en évidence la présence des adduits correspondant à un produit dont la formule brute est la suivante : $\mathbf{C_{85}H_{109}N_{35}O_{59}P_8}$.

9R

- Composé 4 - Tr = 9.6 min

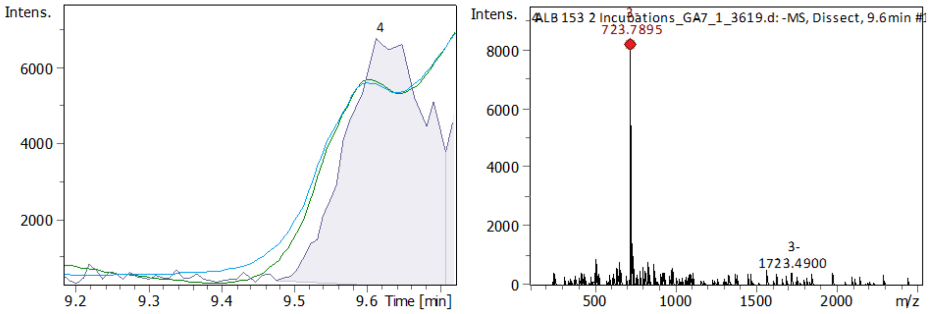

Meas. m/z	Ion Formula	Sum Formula	m/z	err [ppm]	mSigma	Adduct	z
723.7895	C78H84N22O43P5	C78H87N22O43P5	723.7922	3.8	149.6	M-H	3-

→ Le spectre de masse met en évidence la présence des adduits correspondant à un produit dont la formule brute est la suivante : **$C_{78}H_{87}N_{22}O_{43}P_5$**.

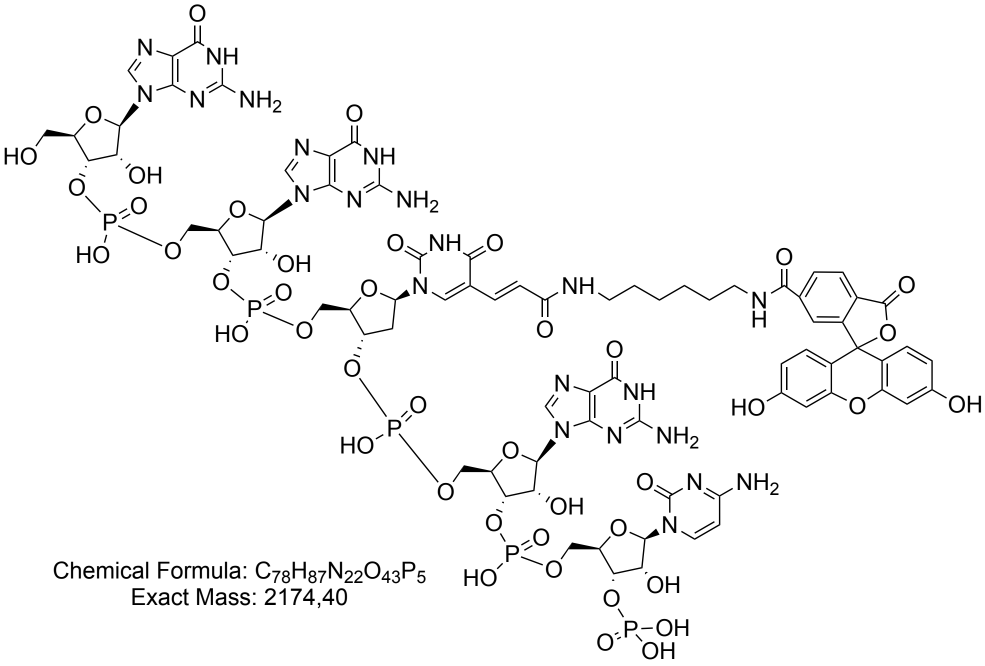

5Rp

- Composé 5 - Tr = 9.8 min

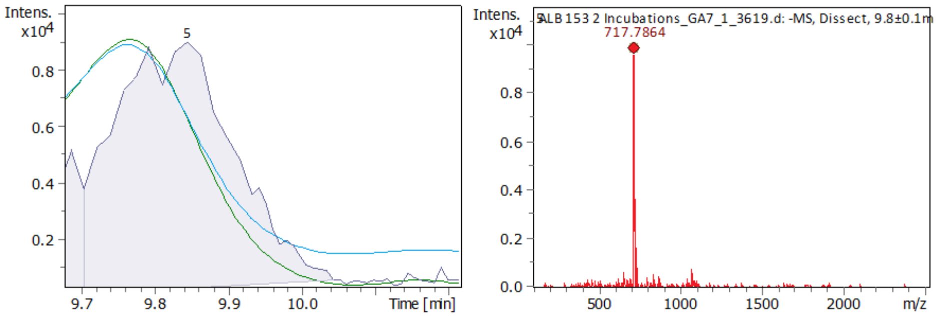

Meas. m/z	Ion Formula	Sum Formula	m/z	err [ppm]	mSigma	Adduct	z
717.7864	C78H82N22O42P5	C78H85N22O42P5	717.7887	3.3	5.2	M-H	3-

→ Le spectre de masse met en évidence la présence des adduits correspondant à un produit dont la formule brute est la suivante : **$C_{78}H_{85}N_{22}O_{42}P_5$**.

5R>p

- Composé 6 - Tr = 11.6 min

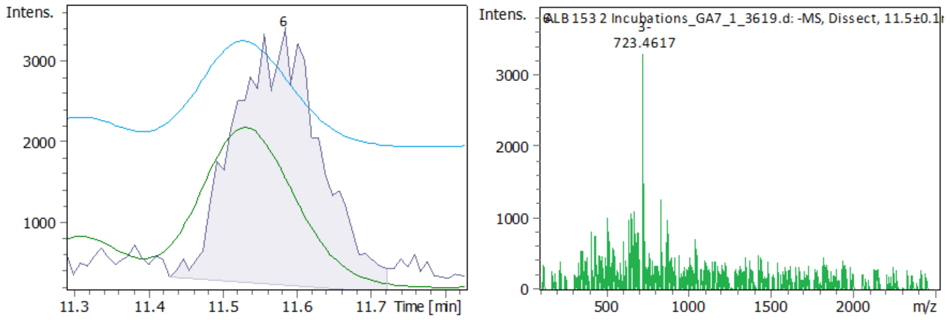

Meas. m/z	Ion Formula	Sum Formula	m/z	err [ppm]	mSigma	Adduct	z
723.4617	C78H85N23O42P5	C78H88N23O42P5	723.4642	3.5	94.8	M-H	3-

→ Le spectre de masse met en évidence la présence des adduits correspondant à un produit dont la formule brute est la suivante : $\mathbf{C_{78}H_{88}N_{23}O_{42}P_5}$.

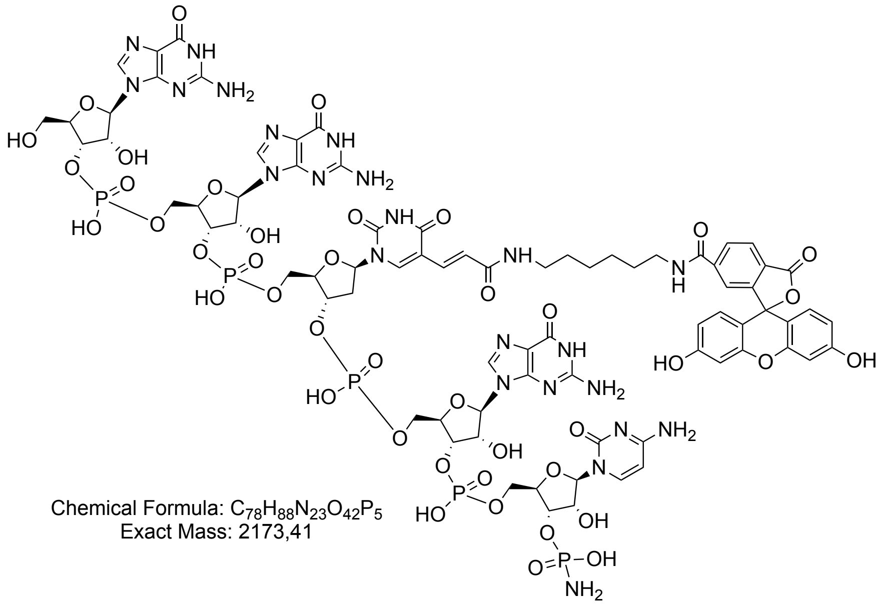

5Rp-NH$_2$

- Composé 7 - Tr = 12.0 min

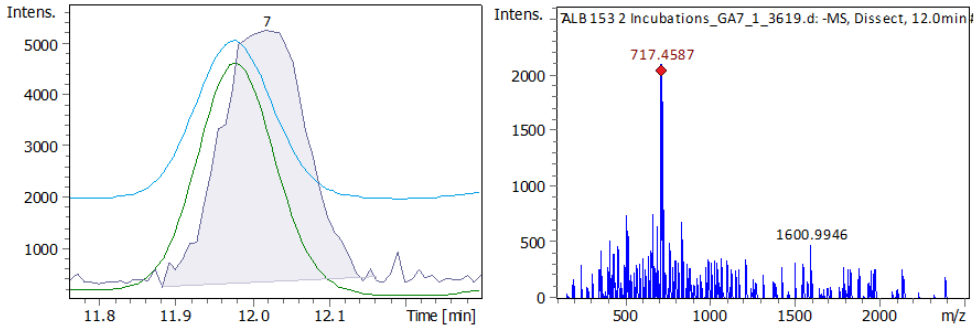

Meas. m/z	Ion Formula	Sum Formula	m/z	err [ppm]	mSigma	Adduct	z
717.4587	C78H83N23O41P5	C78H86N23O41P5	717.4607	2.8	49.7	M-H	3-

→ Le spectre de masse met en évidence la présence des adduits correspondant à un produit dont la formule brute est la suivante : $\mathbf{C_{78}H_{86}N_{23}O_{41}P_5}$.

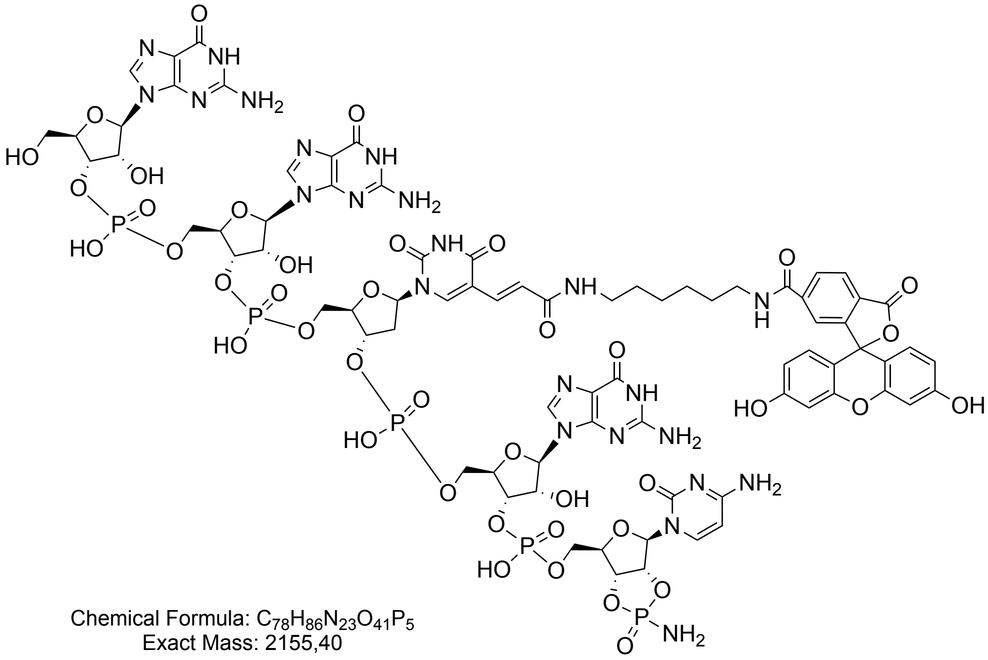

Chemical Formula: $C_{78}H_{86}N_{23}O_{41}P_5$
Exact Mass: 2155,40

5R>p-NH₂

- Composé 8 - Tr = 15.4 min

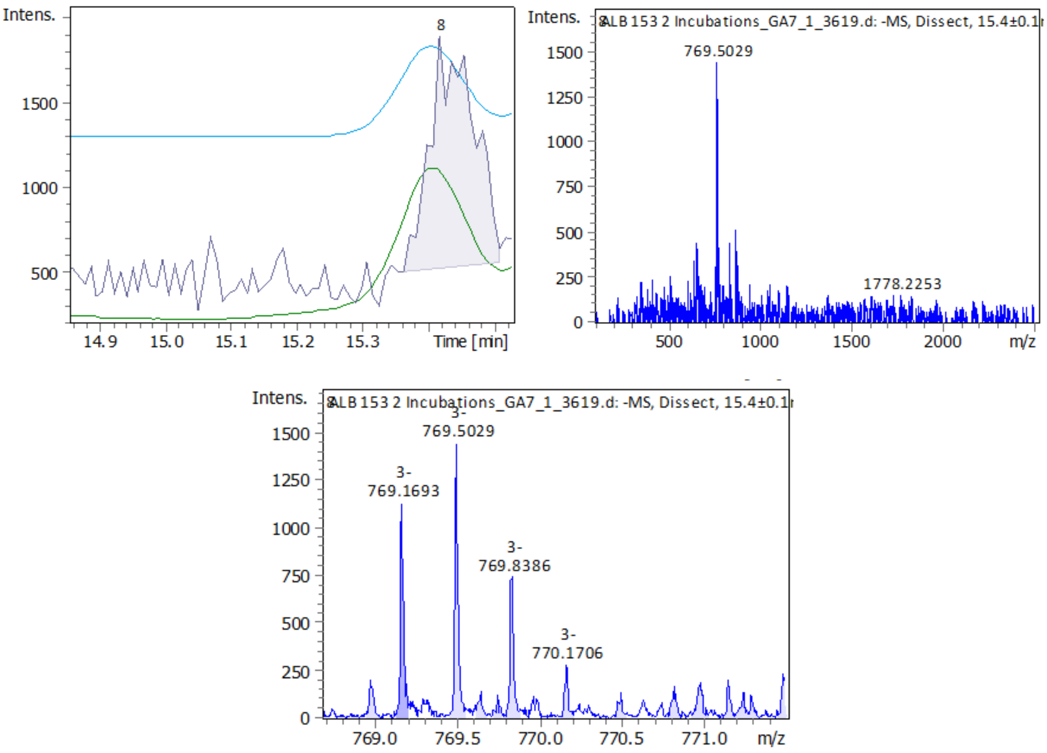

Mass observed: 2311.51

5R>p-EDC

Figure S36. LC-MS chromatogram (negative ion mode), UV chromatograms (260 and 210 nm), Ion extractions chromatograms for products of ligation, peaks and associated compounds obtained for the D3 experiment.

D4 experiment:

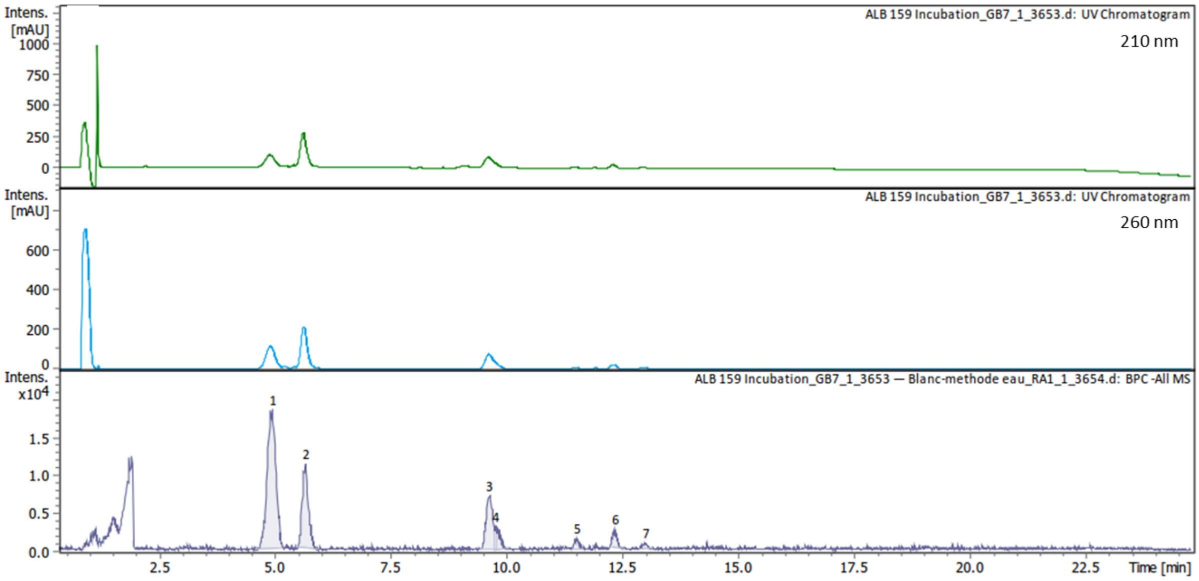

Extracted ion chromatograms for the 2 possible products of ligation:

9Rfp (red, $C_{116}H_{133}N_{36}O_{73}P_9$) or 9Rf>p (blue, $C_{116}H_{131}N_{36}O_{72}P_9$):

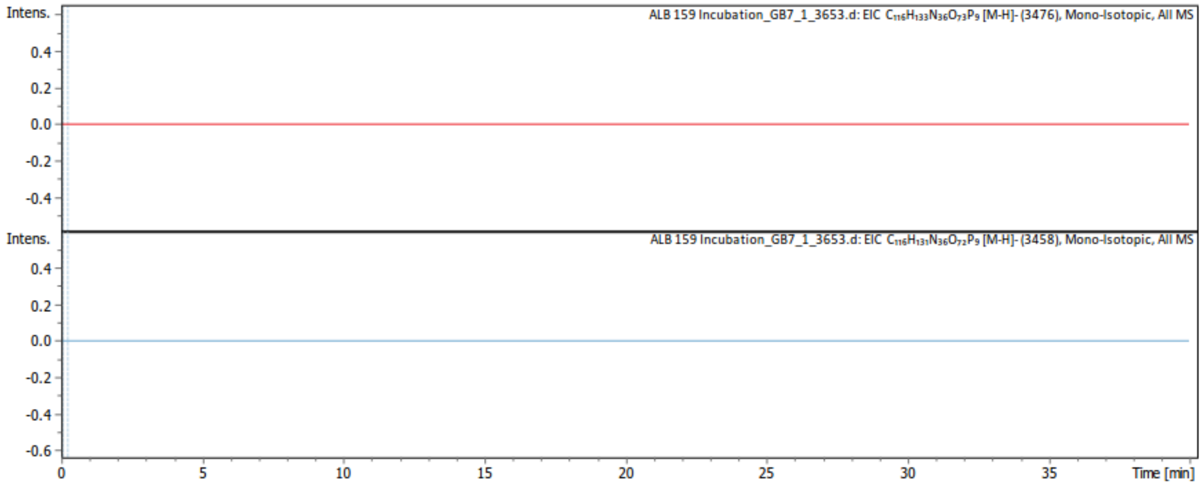

- Composé 1 - Tr = 4.9 min

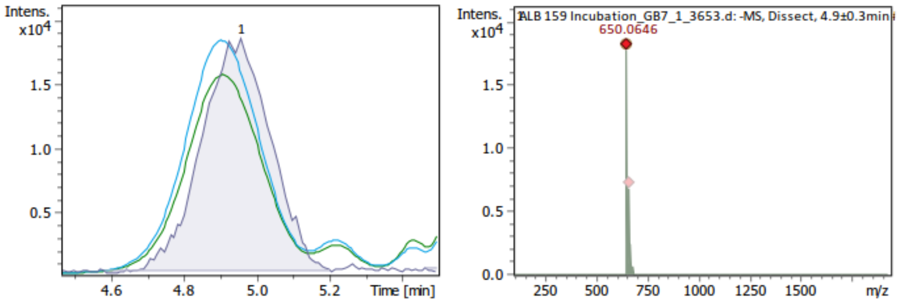

Meas. m/z	Ion Formula	Sum Formula	m/z	err [ppm]	mSigma	Adduct	z
650.0646	C38H44N14O30P4	C38H46N14O30P4	650.0655	1.4	6.6	M-H	2-

→ Le spectre de masse met en évidence la présence des adduits correspondant à un produit dont la formule brute est la suivante : **$C_{38}H_{46}N_{14}O_{30}P_4$**.

4R>P

- Composé 2 - Tr = 5.7 min

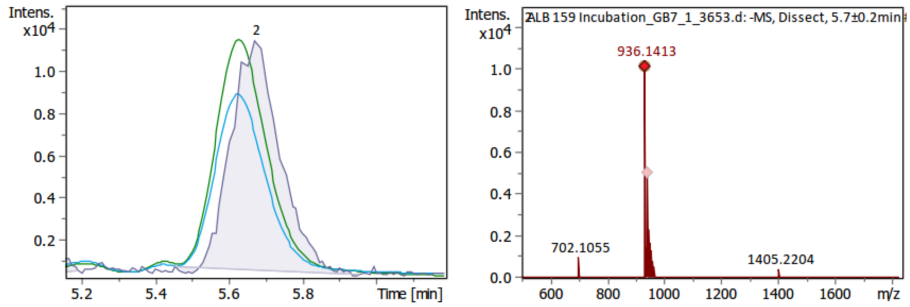

Meas. m/z	Ion Formula	Sum Formula	m/z	err [ppm]	mSigma	Adduct	z
936.1413	C85H106N35O59P8	C85H109N35O59P8	936.1429	1.7	8.7	M-H	3-

→ Le spectre de masse met en évidence la présence des adduits correspondant à un produit dont la formule brute est la suivante : $\mathbf{C_{85}H_{109}N_{35}O_{59}P_{8}}$.

9R

- Composé 3 - Tr = 9.7 min

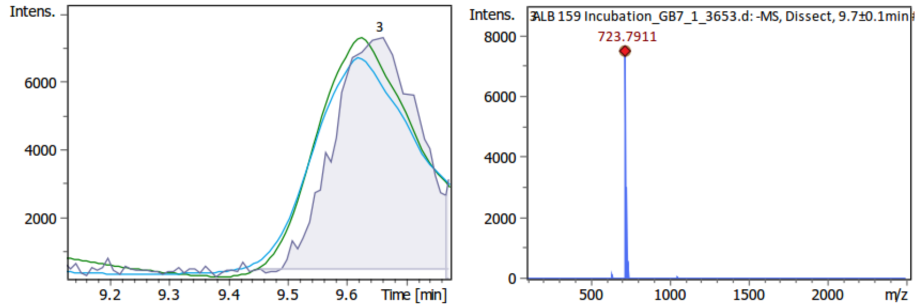

Meas. m/z	Ion Formula	Sum Formula	m/z	err [ppm]	mSigma	Adduct	z
723.7911	C78H84N22O43P5	C78H87N22O43P5	723.7922	1.5	25.8	M-H	3-

→ Le spectre de masse met en évidence la présence des adduits correspondant à un produit dont la formule brute est la suivante : $\mathbf{C_{78}H_{87}N_{22}O_{43}P_{5}}$.

5Rp

- Composé 4 - Tr = 9.8 min

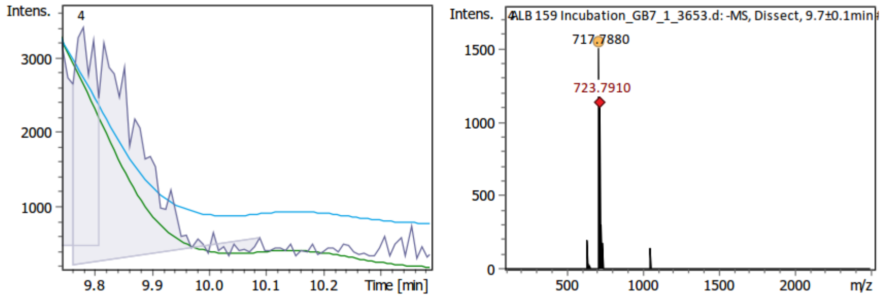

Meas. m/z	Ion Formula	Sum Formula	m/z	err [ppm]	mSigma	Adduct	z
717.788	C78H82N22O42P5	C78H85N22O42P5	717.7887	1	32.7	M-H	3-

→ Le spectre de masse met en évidence la présence des adduits correspondant à un produit dont la formule brute est la suivante : $\mathbf{C_{78}H_{85}N_{22}O_{42}P_{5}}$.

5R>p

- Composé 5 - Tr = 11.5 min

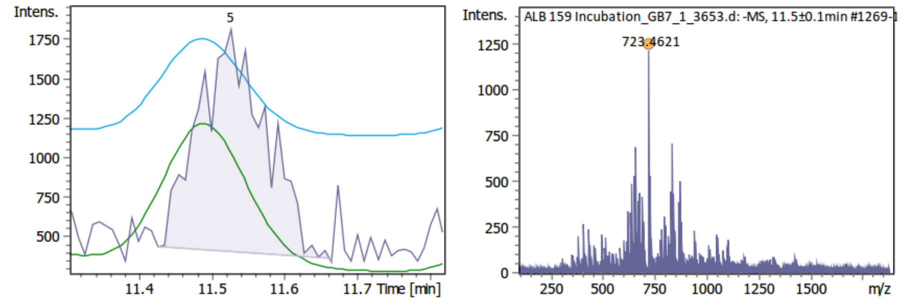

Meas. m/z	Ion Formula	Sum Formula	m/z	err [ppm]	mSigma	Adduct	z
723.4621	C78H85N23O42P5	C78H88N23O42P5	723.4642	3	38.6	M-H	3-

→ Le spectre de masse met en évidence la présence des adduits correspondant à un produit dont la formule brute est la suivante : $\mathbf{C_{78}H_{88}N_{23}O_{42}P_5}$.

5Rp-NH$_2$

- Composé 6 - Tr = 12.3 min

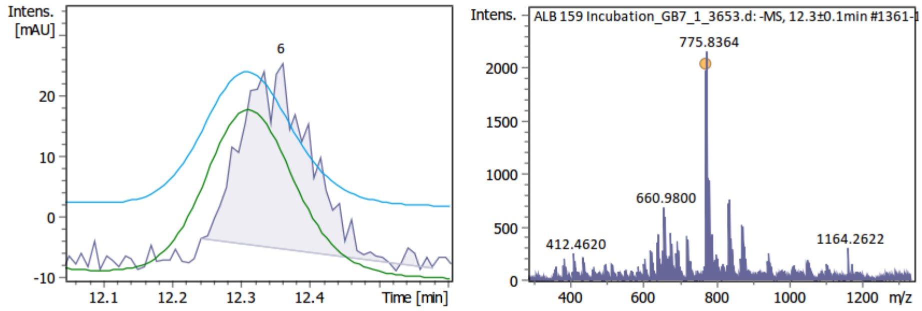

Meas. m/z	Ion Formula	Sum Formula	m/z	err [ppm]	mSigma	Adduct	z
775.502	C86H101N25O43P5	C86H104N25O43P5	775.5063	5.6	22.9	M-H	3-

→ Le spectre de masse met en évidence la présence des adduits correspondant à un produit dont la formule brute est la suivante : $\mathbf{C_{86}H_{104}N_{25}O_{43}P_5}$.

5Rp-EDC

- Composé 7 - Tr = 13.0 min

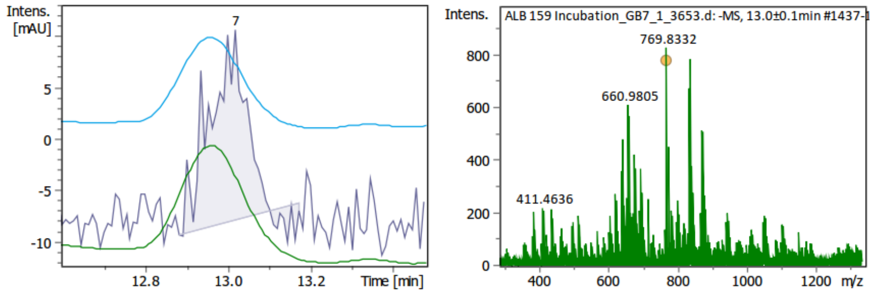

Meas. m/z	Ion Formula	Sum Formula	m/z	err [ppm]	mSigma	Adduct	z
769.4984	C86H99N25O42P5	C86H102N25O42P5	769.5028	5.7	29.7	M-H	3-

→ Le spectre de masse met en évidence la présence des adduits correspondant à un produit dont la formule brute est la suivante : $\mathbf{C_{86}H_{102}N_{25}O_{42}P_5}$.

5R>p-EDC

Figure S37. LC-MS chromatogram (negative ion mode), UV chromatograms (260 and 210 nm), ion extractions chromatograms for products of ligation, peaks and associated compounds obtained for the D4 experiment.

Prebiotic vesicles preparation in D4-like conditions:

Prebiotic vesicles were prepared as followed: A mixture of lipids containing LA, MLG, DLPA (1:1:1) was diluted in a flask in 1 mL of MeOH (5 mM total lipid concentration). The solvent was evaporated using a rotavapor and a bath at 40°C to form a thin lipid film. After dehydration overnight, the film was rehydrated overnight at room temperature with 1 mL of a buffer solution containing 100 mM NaCl pH 5.5. After hydration, the vesicles were left over for 64 h at pH 6.0 at 4°C and then 30 min at 60°C. Micrograph images were taken at each step adding 3 µM of *Nile Red*™.

4)3) Templated ligation attempts performed in one step and vesicle production in the presence of EDC and NaCl

For the last attempts (E experiments), the templated ligations were performed in a single step:

2,500 mol equivalents of EDC (250 mM) were added to 1 equivalent of oligonucleotides involved in the ligation (4-mers and 5-mers, 50 µM) and to 0.1 equivalent of the template (9-mers, 5 µM). 100 mM NaCl was also added, the pH was brought to 6.0 and the reaction was left overnight at 25°C (E experiments). The experiment was carried out for three types of oligonucleotides: RNA only (E.R), hybrid 5-mers and 4-mers with a DNA template (E.H) and DNA only (E.D). After that, 200 µL were sampled and frozen to –80°C for further analyses. The rest was left at 5°C.

The resulting compounds were analysed using LC-MS as detailed previously for the other ligation attempts. Once again, out of all EDC derivatives found the structure of only one of several possible isomers is shown (Figs. S38-S40).

E.R experiment (21h):

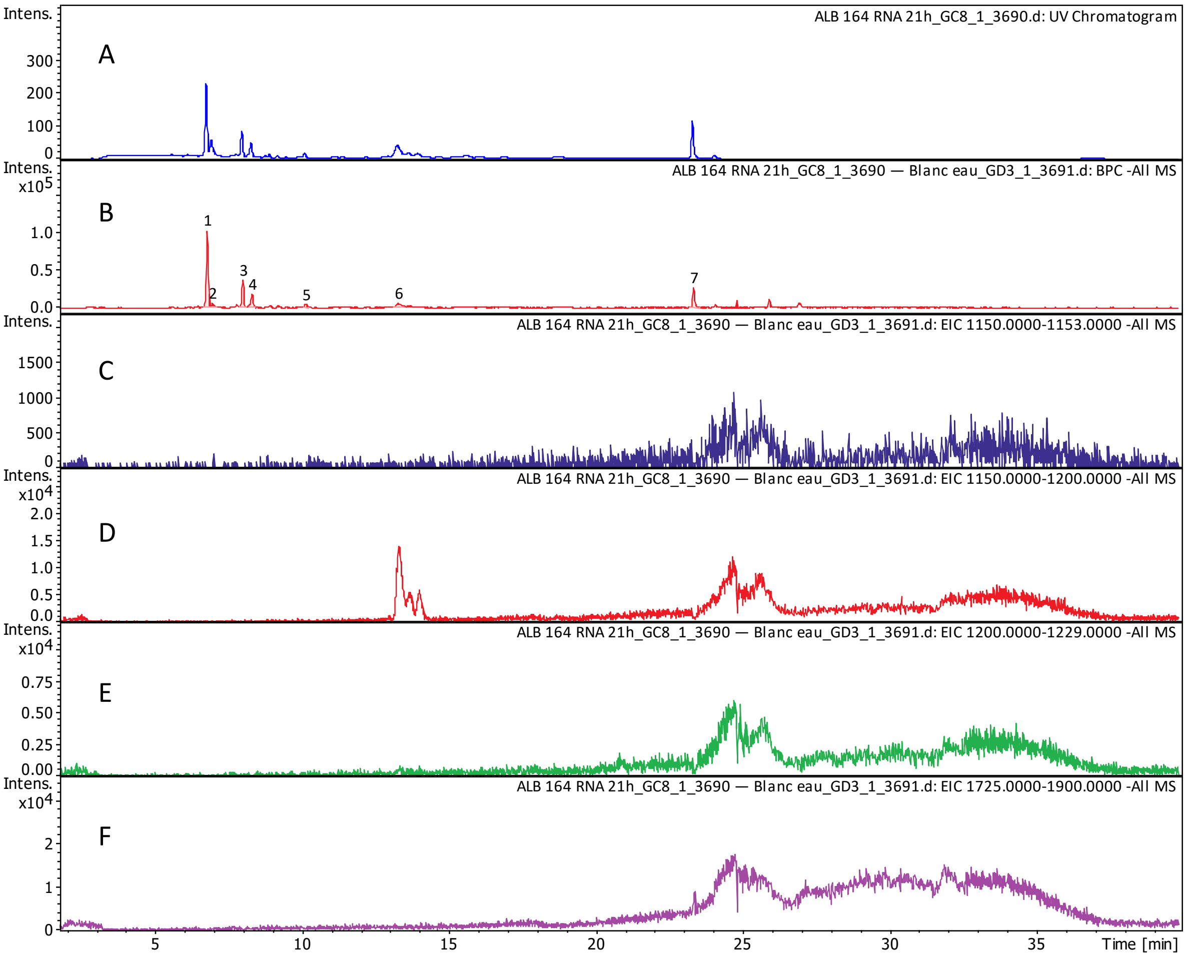

- Composé 1 - Tr = 6.8 min

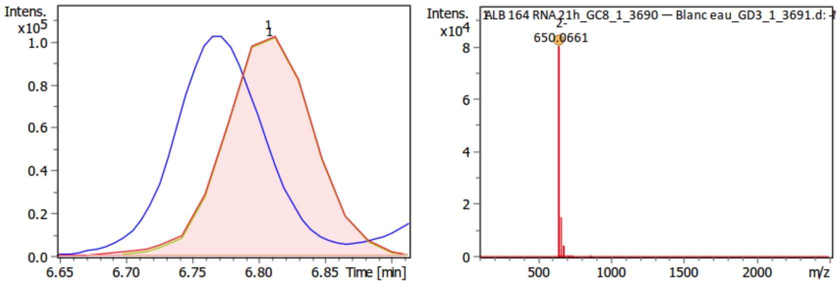

Meas. m/z	Ion Formula	Sum Formula	m/z	err [ppm]	mSigma	Adduct	z
650.0661	C38H44N14O30P4	C38H46N14O30P4	650.0655	-1	26.1	M-H	2-

→ Le spectre de masse met en évidence la présence des adduits correspondant à un produit dont la formule brute est la suivante : **$C_{38}H_{46}N_{14}O_{30}P_4$**.

4R>p

- Composé 2 - Tr = 7.0 min

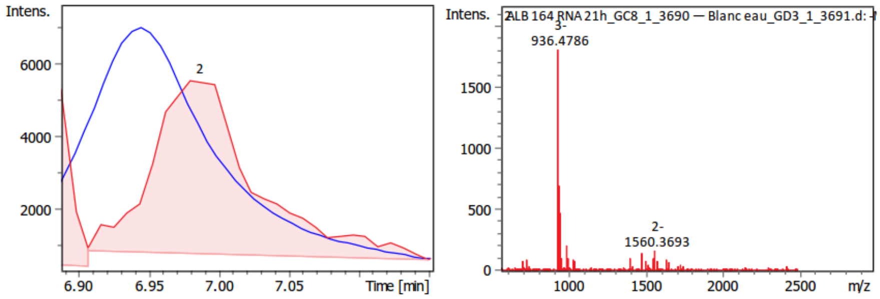

Meas. m/z	Ion Formula	Sum Formula	m/z	err [ppm]	mSigma	Adduct	z
936.1454	C85H106N35O59P8	C85H109N35O59P8	936.1429	-2.6	33.6	M-H	3-

→ Le spectre de masse met en évidence la présence des adduits correspondant à un produit dont la formule brute est la suivante : **$C_{85}H_{109}N_{35}O_{59}P_8$**.

9R

- Composé 3 - Tr = 8.0 min

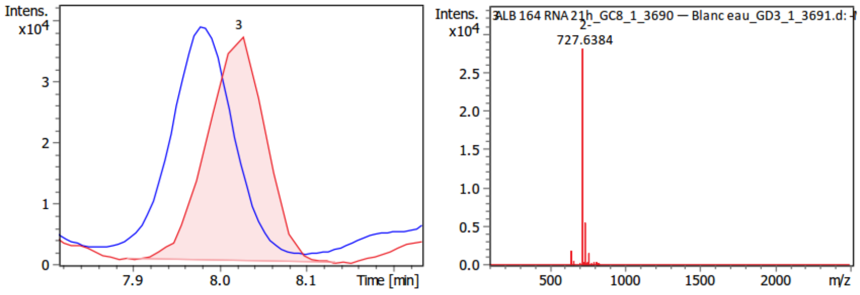

Mass observed: 1457.28

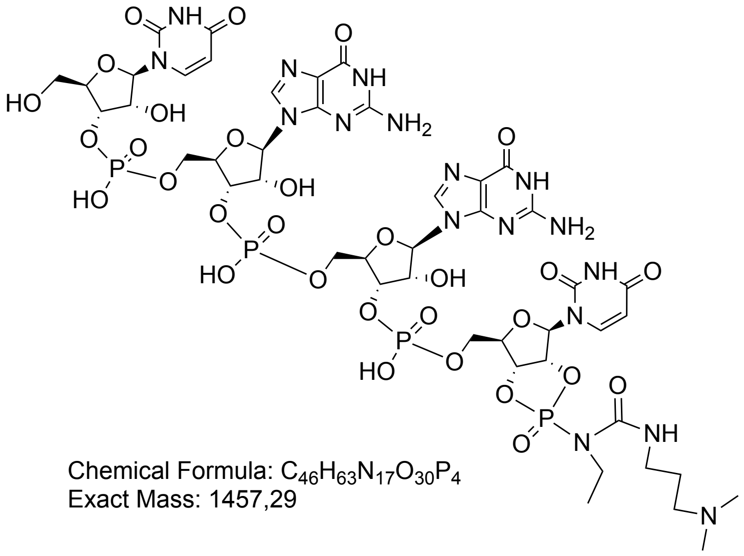

Chemical Formula: $C_{46}H_{63}N_{17}O_{30}P_4$

Exact Mass: 1457,29

4R>p-EDC

- Composé 4 - Tr = 8.3 min

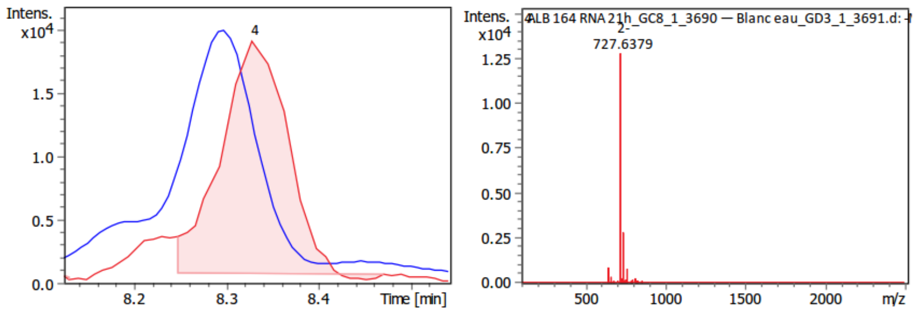

Mass observed: 1457.28

4R>p-EDC

- Composé 5 - Tr = 10.2 min

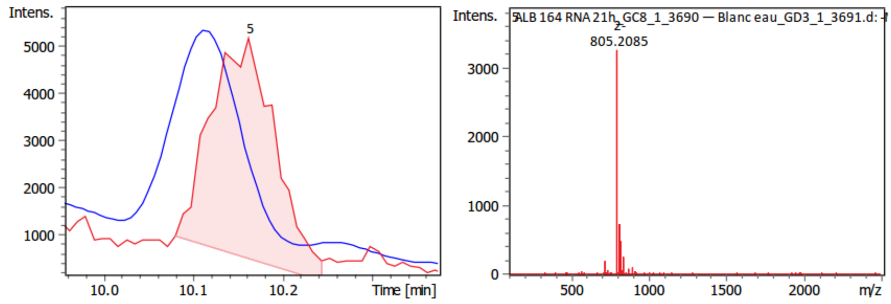

Mass observed: 1612.42

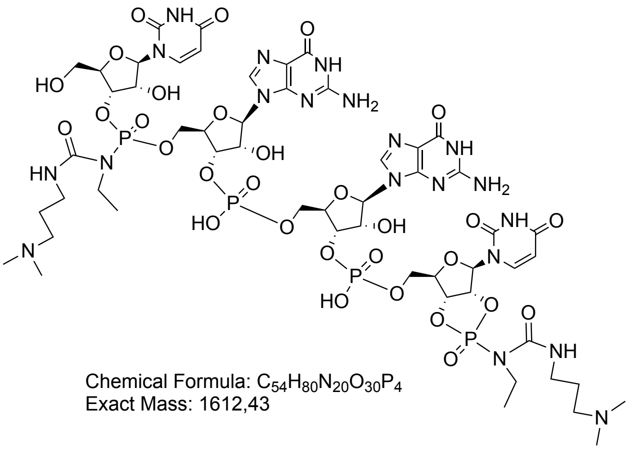

Chemical Formula: $C_{54}H_{80}N_{20}O_{30}P_4$
Exact Mass: 1612,43

4R>p-EDC-EDC

- Composé 6 - Tr = 13.3 min

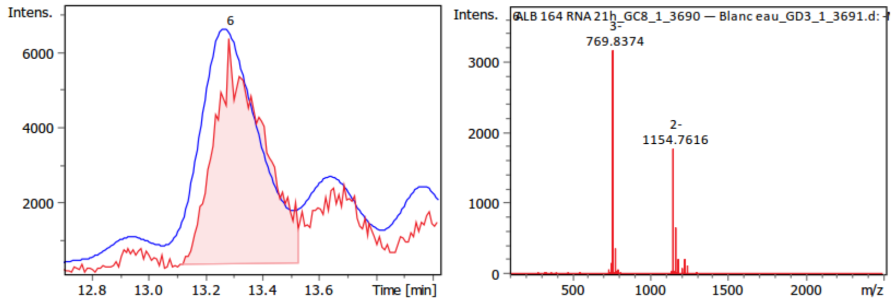

Mass observed: 2312.51 and 2311.5232 Unidentified derivative and 5R>p-EDC

- Composé 7 - Tr = 23.4 min

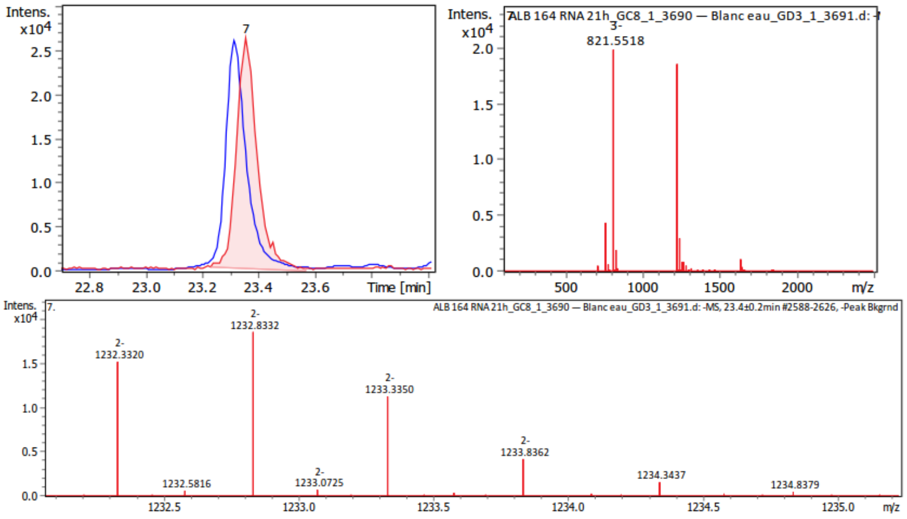

Mass observed: 2467.66 and 2466.67

Unidentified derivative and

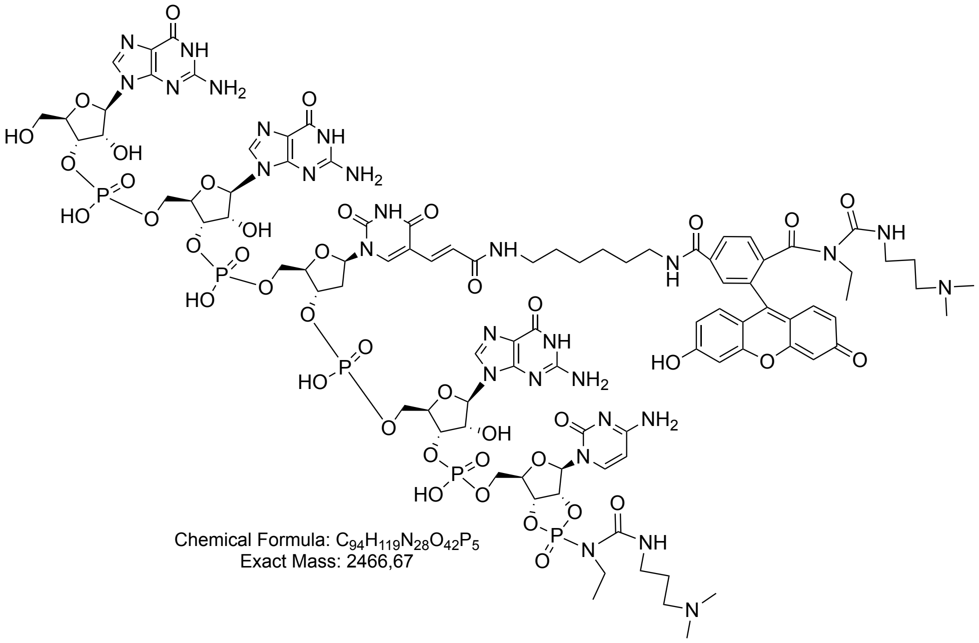

Chemical Formula: $C_{94}H_{119}N_{28}O_{42}P_5$
Exact Mass: 2466,67

5R>p-EDC-EDC

Figure S38. E.R experiment after 21 hours of incubation at ambient temperature. A) UV chromatogram (260 nm), B) all-negative-ion LC-MS chromatogram (main peaks annotated 1-7), C-F) extracted ion chromatograms (EIC) for mass ranges of the possible products of ligation targeting C: $[9Rf>p]^{3-}$, D: $[9Rf>p]^{3-}$ and $[9Rfp]^{3-}$ (found in peak 6: $[5R>p\text{-}EDC]^{2-}$ and 2 unidentified $[UI]^{2-}$), E: $[9Rf>p\text{-}EDC]^{3-}$ and $[9Rfp\text{-}EDC]^{3-}$, F: $[9Rf>p]^{2-}$, $[9Rfp]^{2-}$, $[9Rf>p\text{-}EDC]^{2-}$, $[9Rfp\text{-}EDC]^{2-}$ and $[9Rfp\text{-}EDC\text{-}EDC]^{2-}$ found in peak 7: $[5R>p\text{-}EDC\text{-}EDC]^{2-}$), followed by mass spectra of the annotated peaks, their molecular masses (1-7) and identified compounds (only one isomer shown).

E.H experiment (21h):

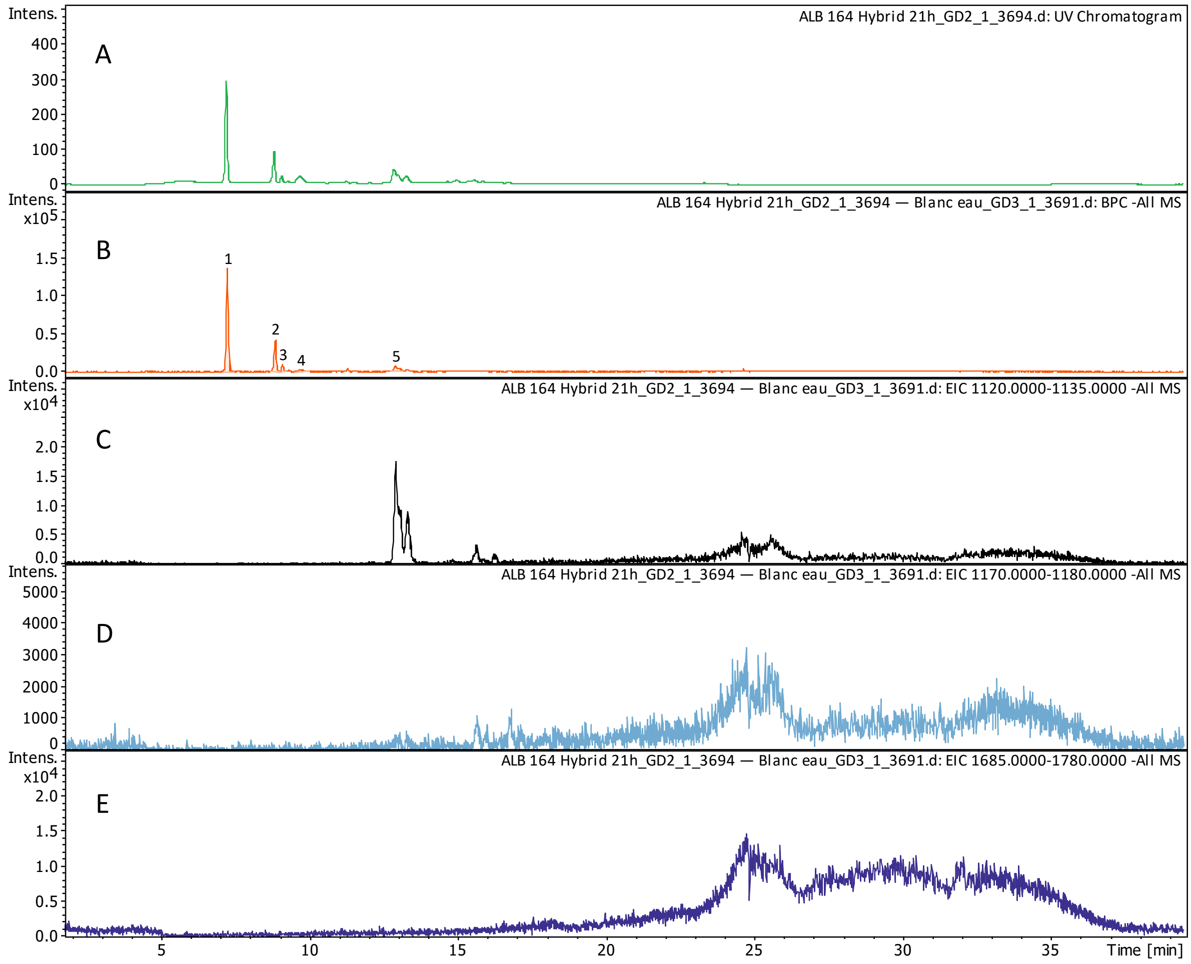

- Composé 1 - Tr = 7.3 min

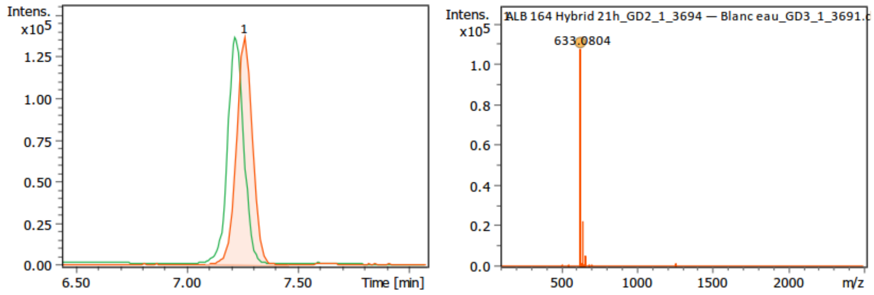

Meas. m/z	Ion Formula	Sum Formula	m/z	err [ppm]	mSigma	Adduct	z
633.0804	C39H46N14O27P4	C39H48N14O27P4	633.0809	0.9	19.5	M-H	2-

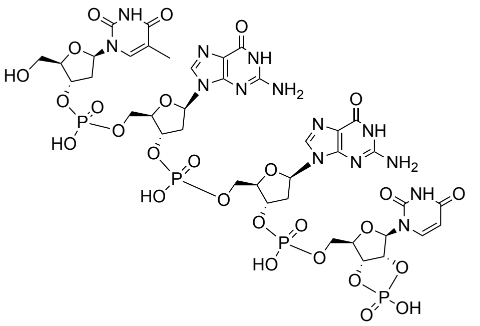

Chemical Formula: $C_{39}H_{48}N_{14}O_{27}P_4$
Exact Mass: 1268,18

4H>p

- Composé 2 - Tr = 8.9 min

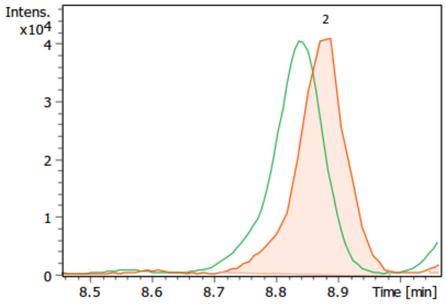

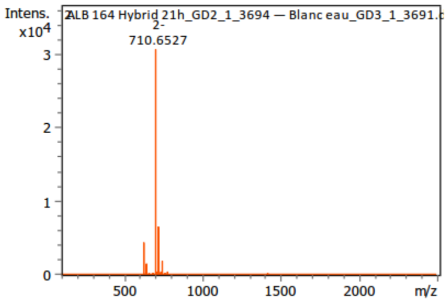

Mass observed: 1423.31

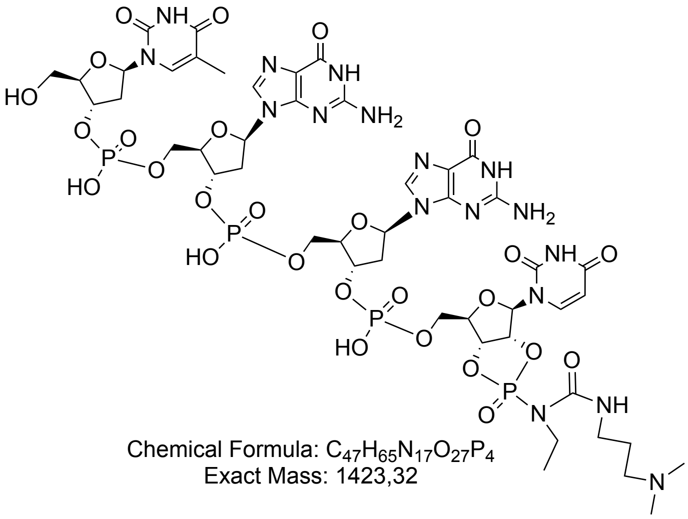

Chemical Formula: $C_{47}H_{65}N_{17}O_{27}P_4$
Exact Mass: 1423,32

4H>p-EDC

- Composé 3 - Tr = 9.1 min

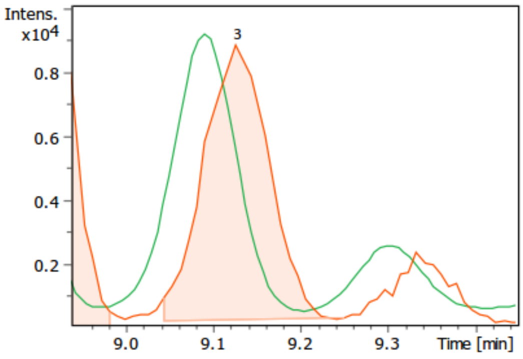

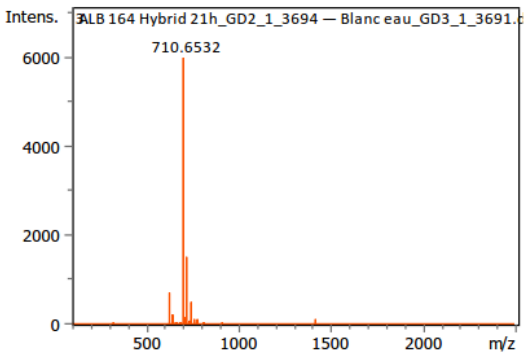

Mass observed: 1423.31

4H>p-EDC

- Composé 4 - Tr = 9.7 min

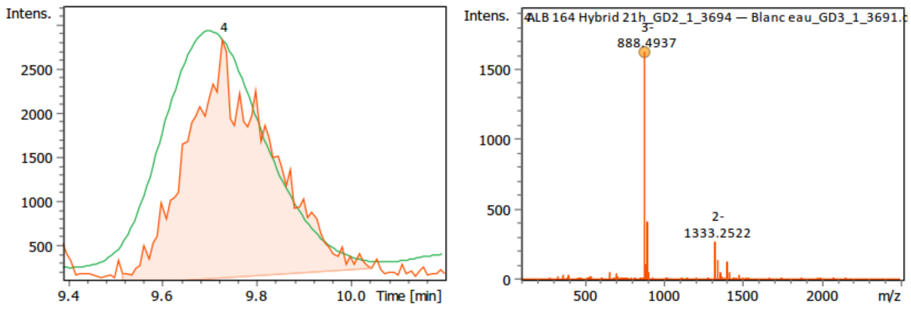

Meas. m/z	Ion Formula	Sum Formula	m/z	err [ppm]	mSigma	Adduct	z
888.1598	C85H106N35O50P8	C85H109N35O50P8	888.1582	-1.8	39.2	M-H	3-

. → Le spectre de masse met en évidence la présence des adduits correspondant à un produit dont la formule brute est la suivante : **$C_{85}H_{109}N_{35}O_{50}P_8$**.

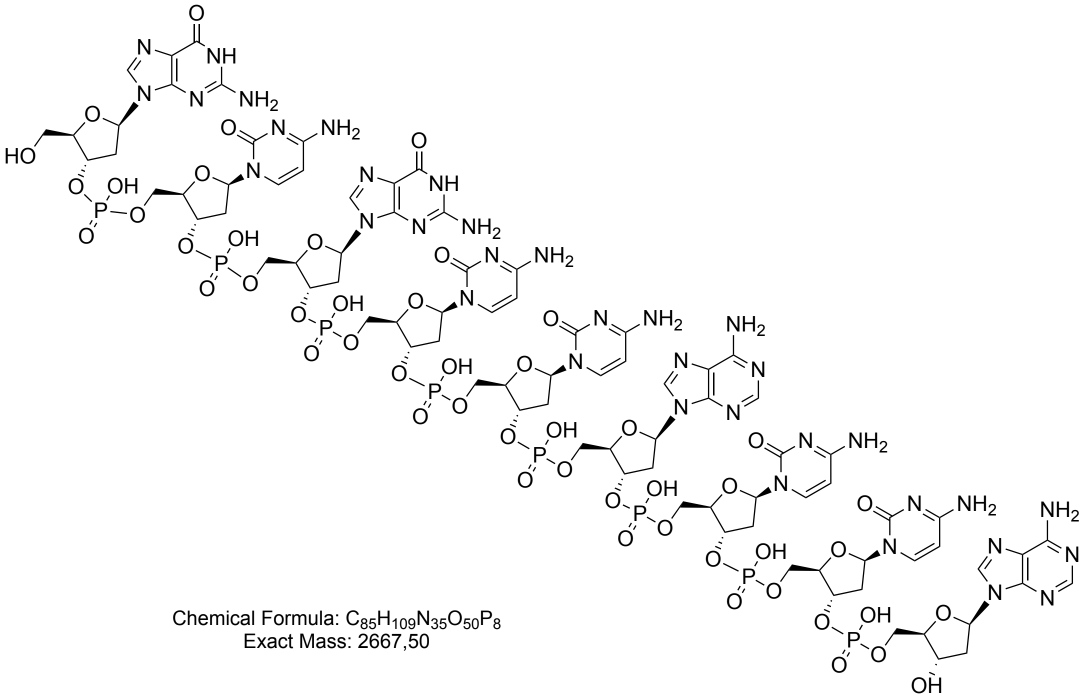

9D

- Composé 5 - Tr = 12.9min

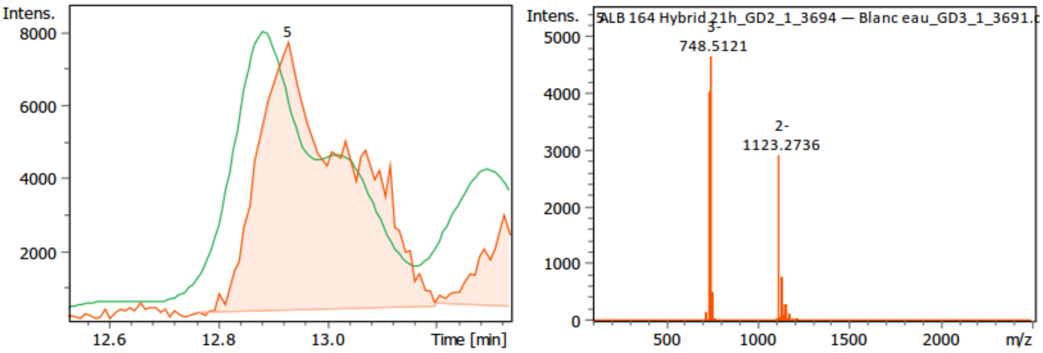

Masses observed: 2247.54 and 2248.55

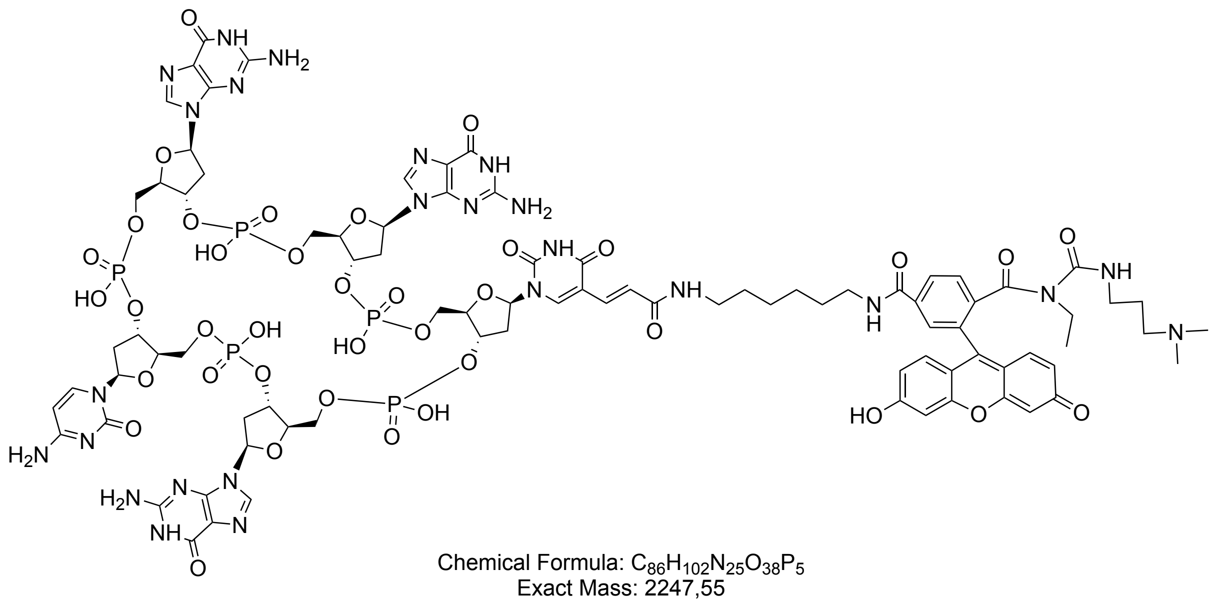

Chemical Formula: $C_{86}H_{102}N_{25}O_{38}P_5$
Exact Mass: 2247,55

c5Dp-EDC

Figure S39. E.H experiment after 21 hours of incubation at ambient temperature. A) UV chromatogram (260 nm), B) all-negative-ion LC-MS chromatogram (main peaks annotated 1-5), C-E) extracted ion chromatograms (EIC) for mass ranges of the possible products of ligation targeting C: $[9Hf{>}p]^{3-}$ and $[9Hfp]^{3-}$ (found in peak 5: $[c5Dp\text{-}EDC]^{2-}$, 13-17 min. several unidentified $[UI]^{2-}$), D: $[9Hf{>}p\text{-}EDC]^{2-}$ and $[9Hfp\text{-}EDC]^{2-}$, E: $[9Hf{>}p]^{2-}$, $[9Hfp]^{2-}$, $[9Hf{>}p\text{-}EDC]^{2-}$, $[9Hfp\text{-}EDC]^{2-}$ and $[9Hfp\text{-}EDC\text{-}EDC]^{2-}$, followed by mass spectra of the annotated peaks, their molecular masses (1-5) and identified compounds (only one isomer shown).

E.D experiment (21h):

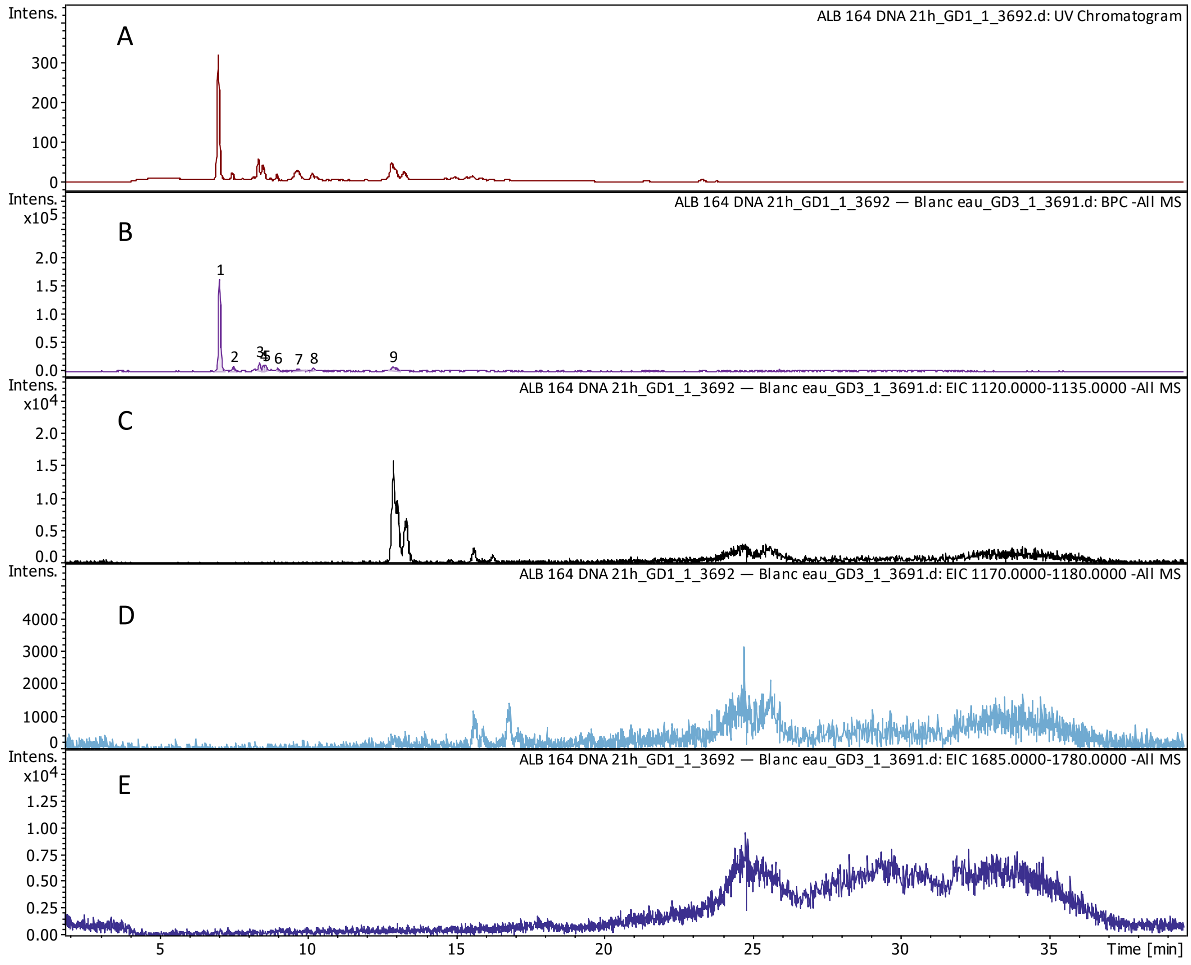

- Composé 1 - Tr = 7.1 min

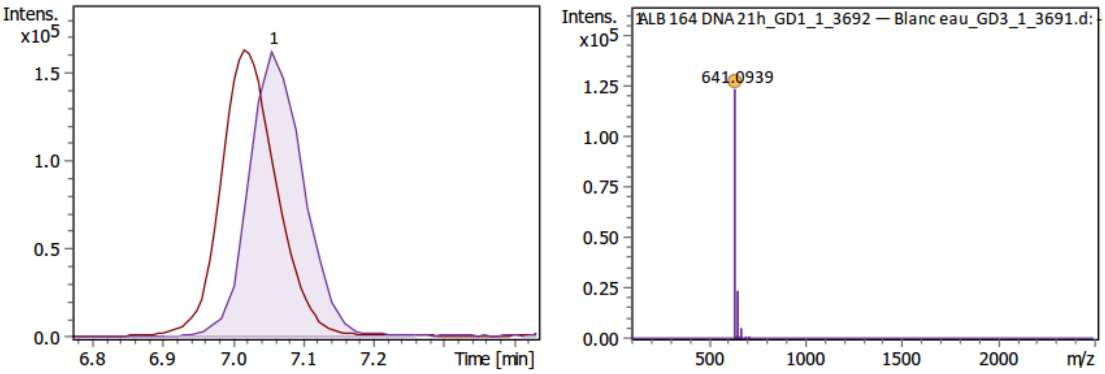

Meas. m/z	Ion Formula	Sum Formula	m/z	err [ppm]	mSigma	Adduct	z
641.0939	C40H50N14O27P4	C40H52N14O27P4	641.0966	4.1	27.1	M-H	2-

→ Le spectre de masse met en évidence la présence des adduits correspondant à un produit dont la formule brute est la suivante : **$C_{40}H_{52}N_{14}O_{27}P_4$**.

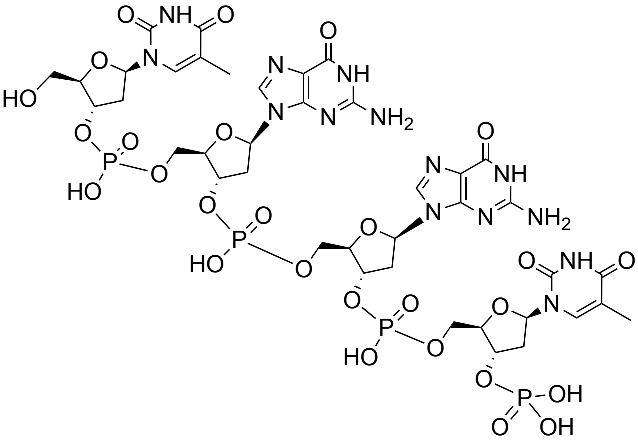

Chemical Formula: $C_{40}H_{52}N_{14}O_{27}P_4$
Exact Mass: 1284,21

4Dp

- Composé 2 - Tr = 7.5 min

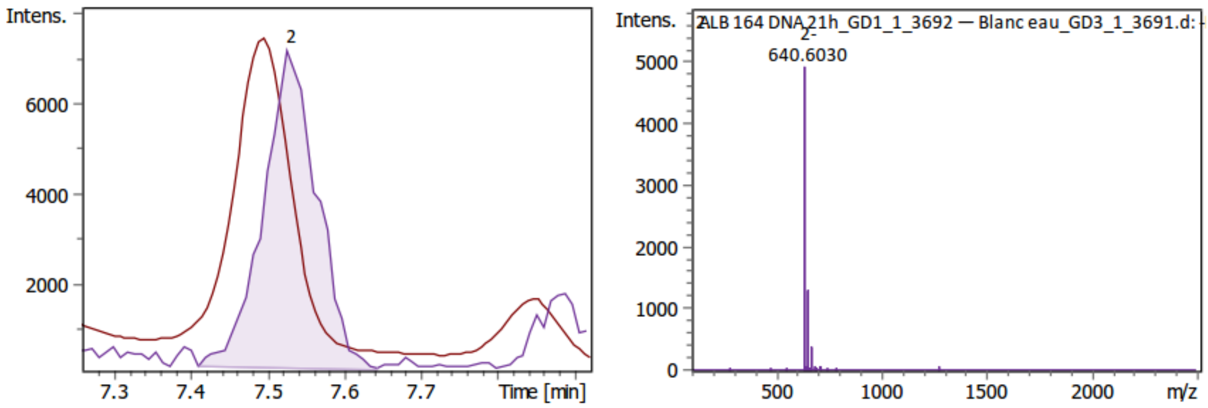

Mass observed: 1283.21

Unidentified derivative

- Composé 3 - Tr = 8.4 min

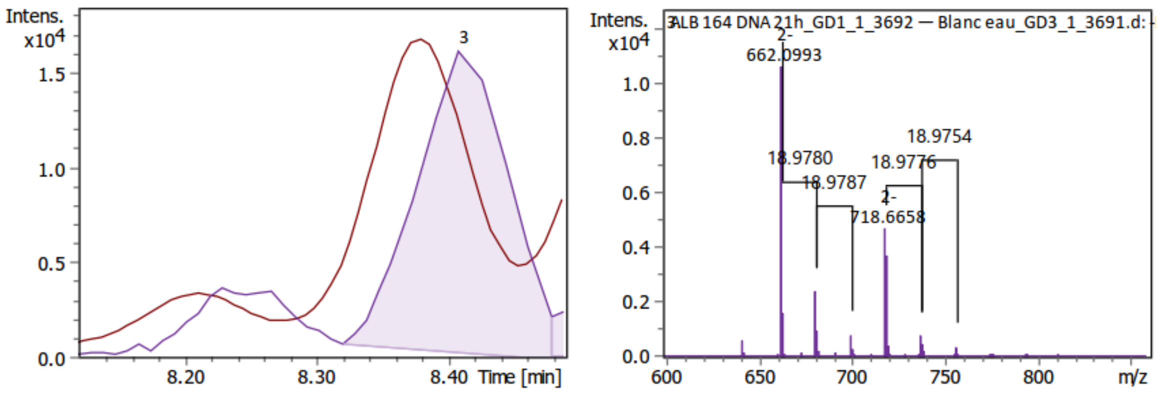

Mass observed: 1326.20

Unidentified derivative

- Composé 4 - Tr = 8.5 min

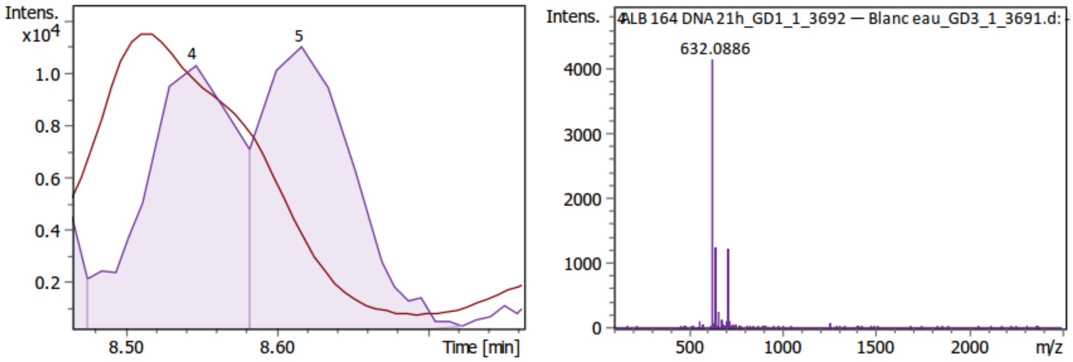

Mass observed: 1266.18

Unidentified derivative

- Composé 5 - Tr = 8.6 min

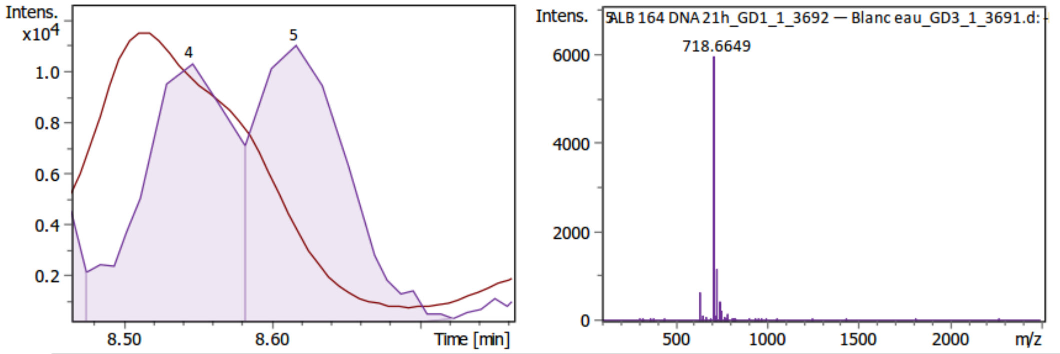

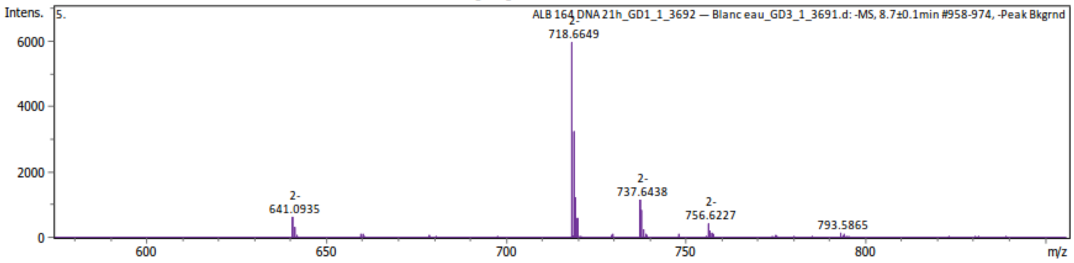

Mass observed: 1439.33

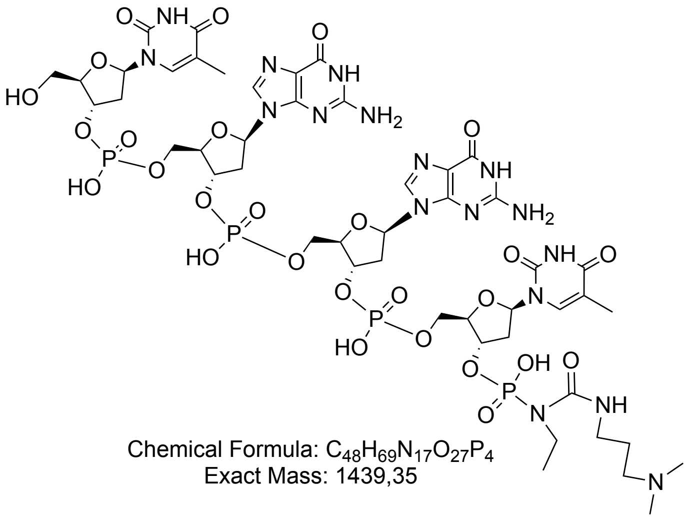

4Dp-EDC

- Composé 6 - Tr = 9.0 min

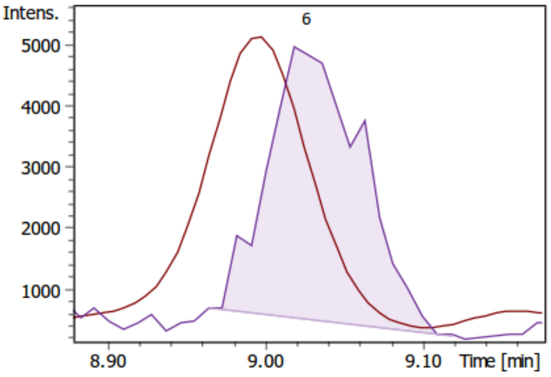

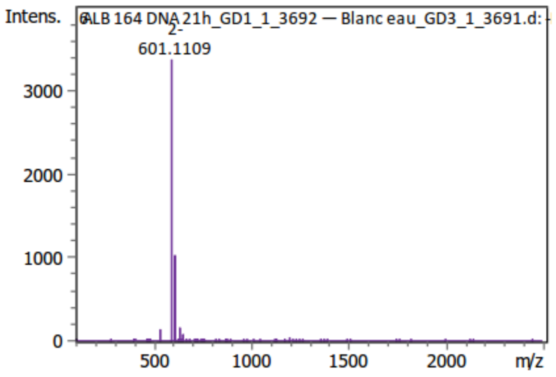

Mass observed: 1204.22

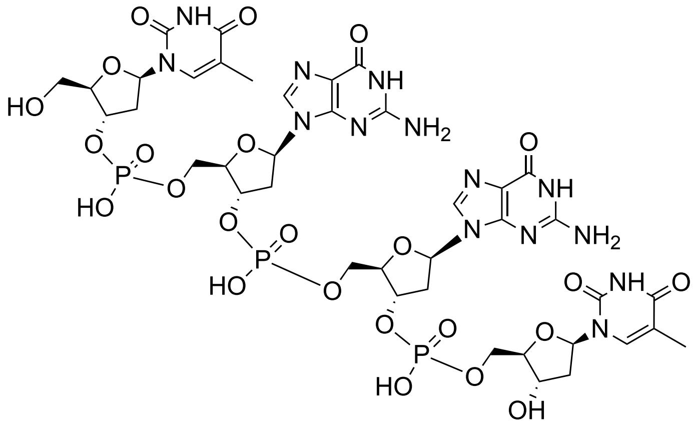

Chemical Formula: $C_{40}H_{51}N_{14}O_{24}P_3$
Exact Mass: 1204,24

4D

- Composé 7 - Tr = 9.7 min

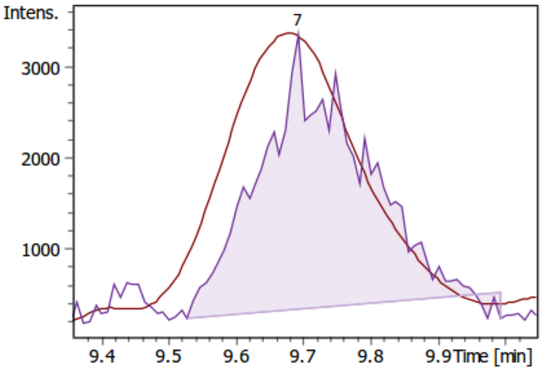

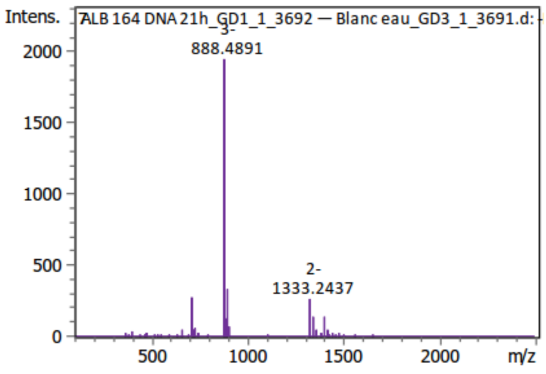

Meas. m/z	Ion Formula	Sum Formula	m/z	err [ppm]	mSigma	Adduct	z
888.1546	C85H106N35O50P8	C85H109N35O50P8	888.1582	4.1	25	M-H	3-

→ Le spectre de masse met en évidence la présence des adduits correspondant à un produit dont la formule brute est la suivante : **$C_{85}H_{109}N_{35}O_{50}P_8$.**

9D

- Composé 8 - Tr = 10.2 min

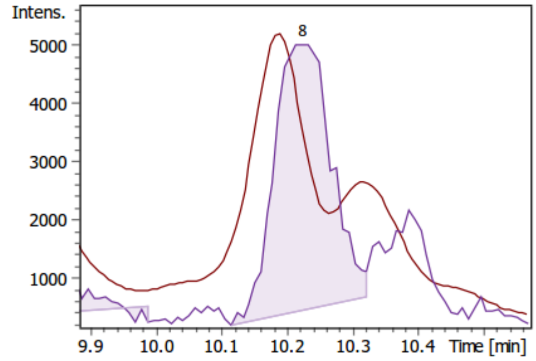

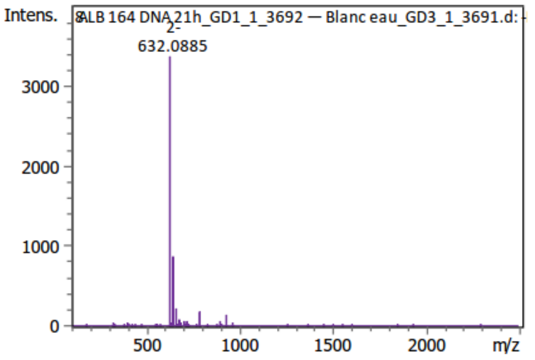

Mass observed: 1266.16 Unidentified derivative

- Composé 9 - Tr = 12.9 min

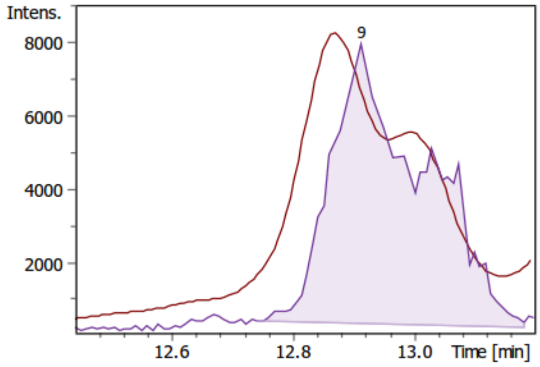

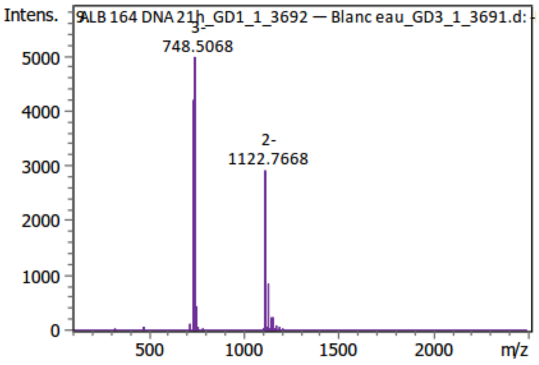

Masses observed: 2248.52 and 2247.53

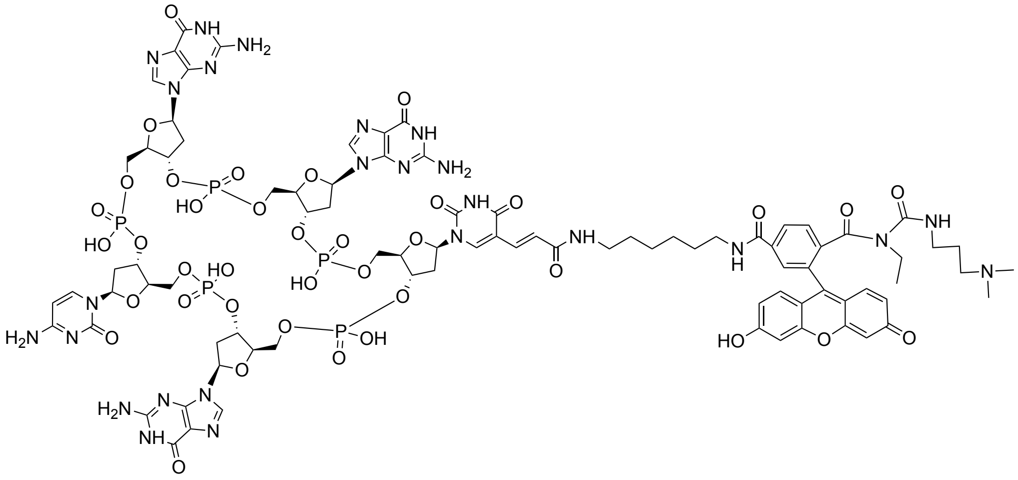

Chemical Formula: $C_{86}H_{102}N_{25}O_{38}P_5$
Exact Mass: 2247,55

Unidentified derivative and c5Dp-EDC

Figure S40. E.D experiment after 21 hours of incubation at ambient temperature. A) UV chromatogram (260 nm), B) all-negative-ion LC-MS chromatogram (main peaks annotated 1-9), C-E) extracted ion chromatograms (EIC) for mass ranges of the possible products of ligation targeting C: $[9Df{>}p]^{3-}$ and $[9Dfp]^{3-}$ (found in peak 9: $[c5Dp\text{-}EDC]^{2-}$, 13-17 min. several unidentified $[UI]^{2-}$), D: $[9Df{>}p\text{-}EDC]^{2-}$ and $[9Dfp\text{-}EDC]^{2-}$, E: $[9Df{>}p]^{2-}$, $[9Dfp]^{2-}$, $[9Df{>}p\text{-}EDC]^{2-}$, $[9Dfp\text{-}EDC]^{2-}$ and $[9Dfp\text{-}EDC\text{-}EDC]^{2-}$, followed by mass spectra of the annotated peaks, their molecular masses (1-9) and identified compounds (only one isomer shown).

E.R experiment (10 months):

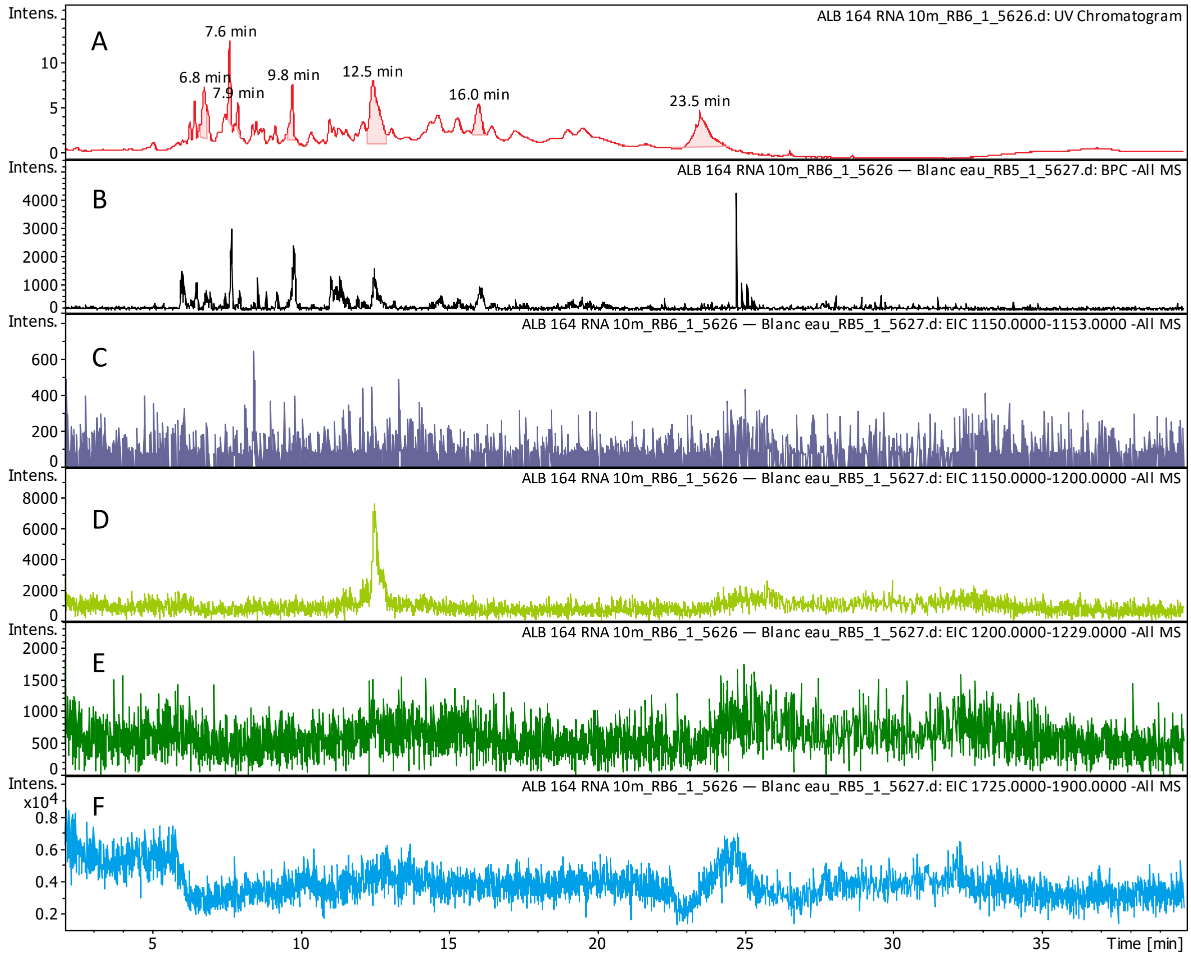

- Composé 1 - Tr = 6.8 min

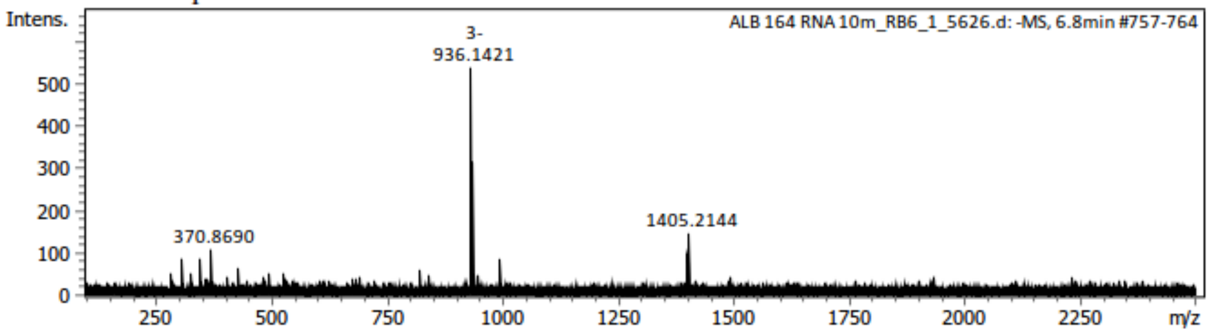

Mass observed: 2811.43

9R

- Composé 2 - Tr = 7.6 min

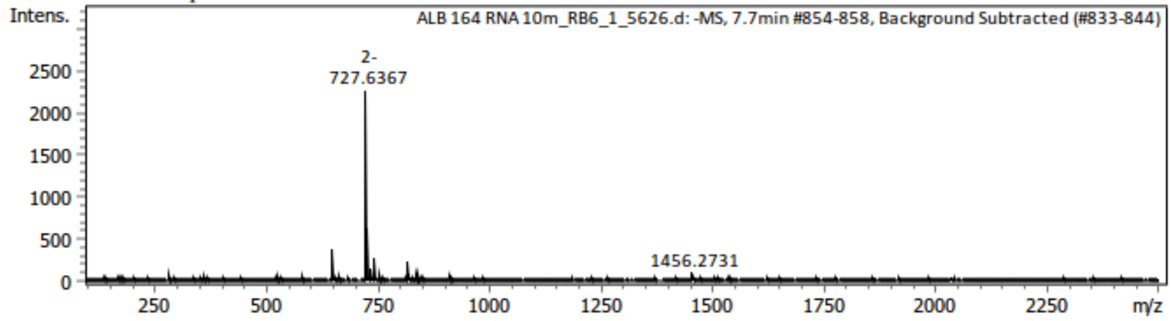

Mass observed: 1457.27

4R>p-EDC

- Composé 3 - Tr = 7.6 min

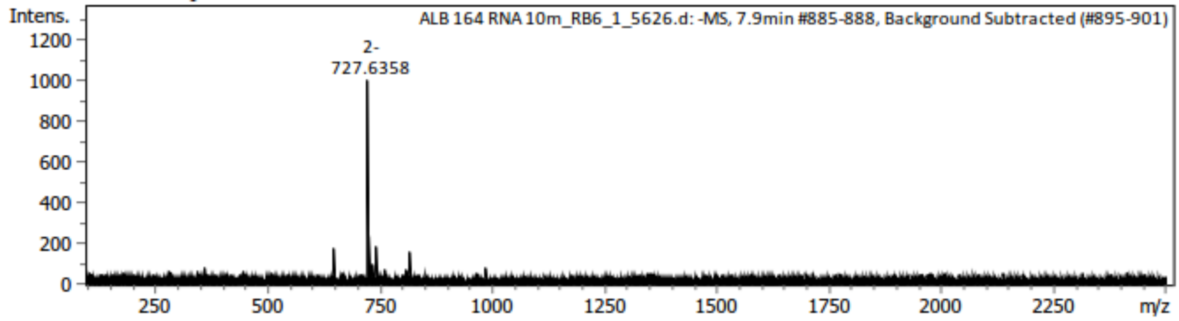

Mass observed: 1457.27

4R>p-EDC

- Composé 4 - Tr = 9.8 min

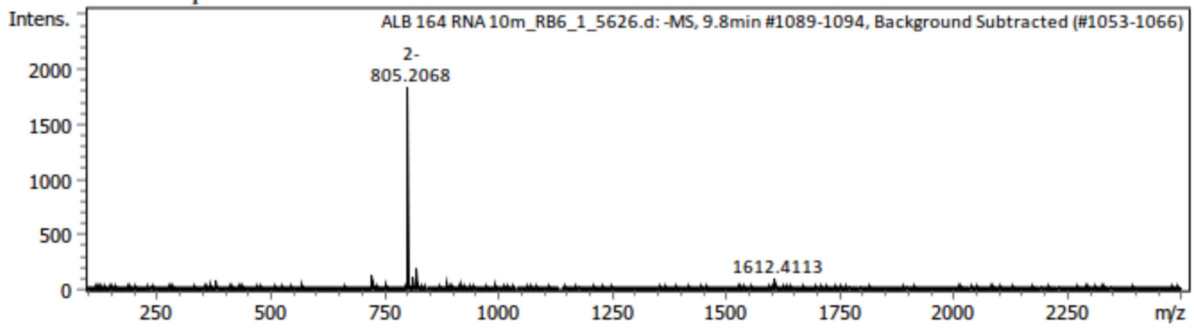

Mass observed: 1612.41

4R>p-EDC-EDC

- Composé 4 - Tr = 12.5 min

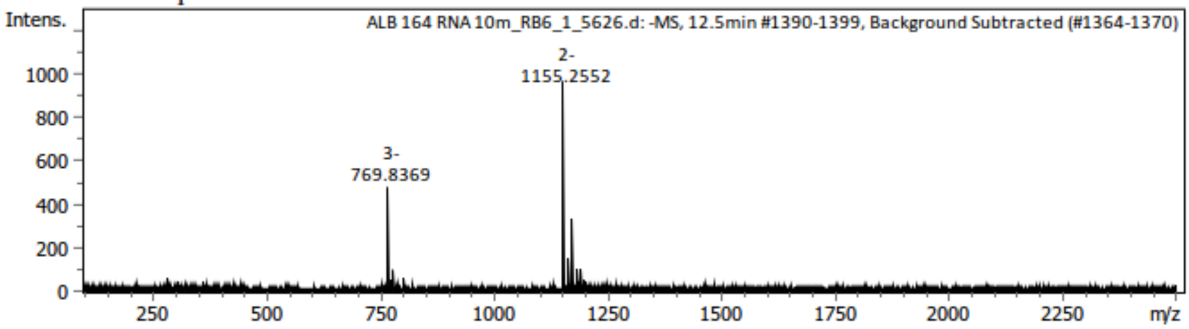

Mass observed: 2312.51

5R>p-EDC

- Composé 4 - Tr = 16.0 min

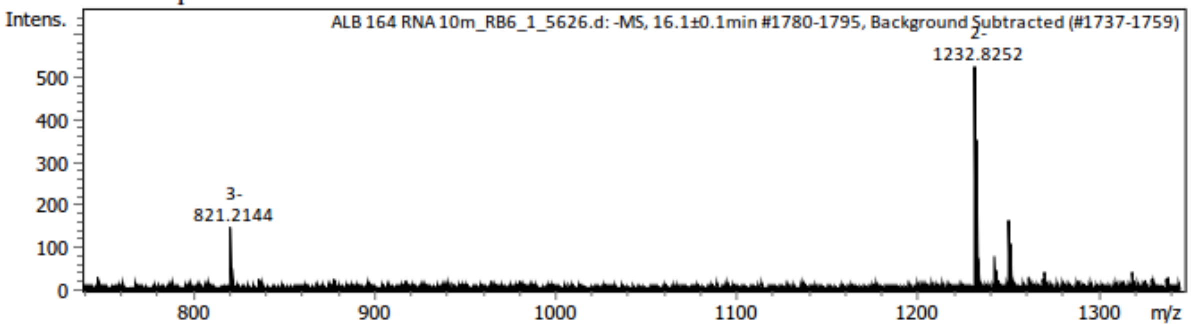

Mass observed: 2467.51

5R>p-EDC-EDC

- Composé 5 - Tr = 23.5 min

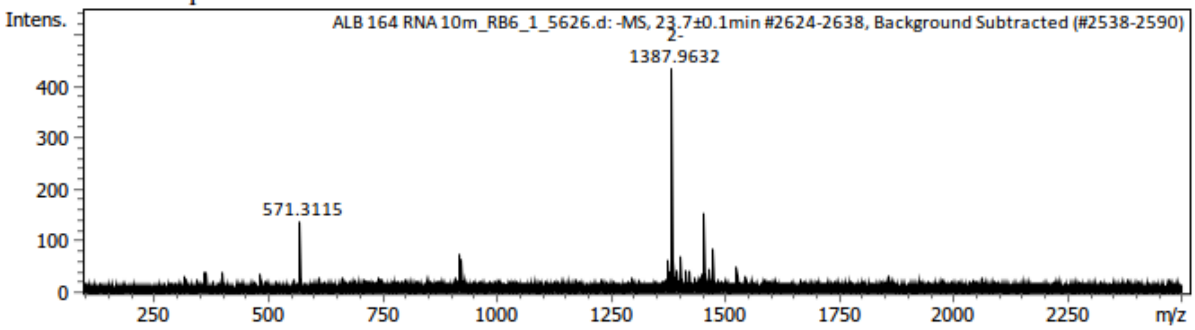

Mass observed: 2777.92

Unidentified derivative

Figure S41. E.R experiment after 10 months-incubation at 4-6°C. A) UV chromatogram (260 nm), B) all-negative-ion LC-MS chromatogram, picked (colour-filled) main peaks 1-5 annotated with their retention times, C-F) extracted ion chromatograms (EIC) for mass ranges of the possible products of ligation targeting C: $[9Rf>p]^{3-}$, D: $[9Rf>p]^{3-}$ and $[9Rfp]^{3-}$ (found at 12.5, 16.0 and 23.5 min: $[5R>p\text{-}EDC]^{2-}$, $[5R>p\text{-}EDC\text{-}EDC]^{2-}$ and unidentified $[UI]^{2-}$), E: $[9Rf>p\text{-}EDC]^{3-}$ and $[9Rfp\text{-}EDC]^{3-}$, F: $[9Rf>p]^{2-}$, $[9Rfp]^{2-}$, $[9Rf>p\text{-}EDC]^{2-}$, $[9Rfp\text{-}EDC]^{2-}$ and $[9Rfp\text{-}EDC\text{-}EDC]^{2-}$, followed by mass spectra of the annotated peaks, associated molecular masses (1-5) and identified compounds.

E.H experiment (10 months):

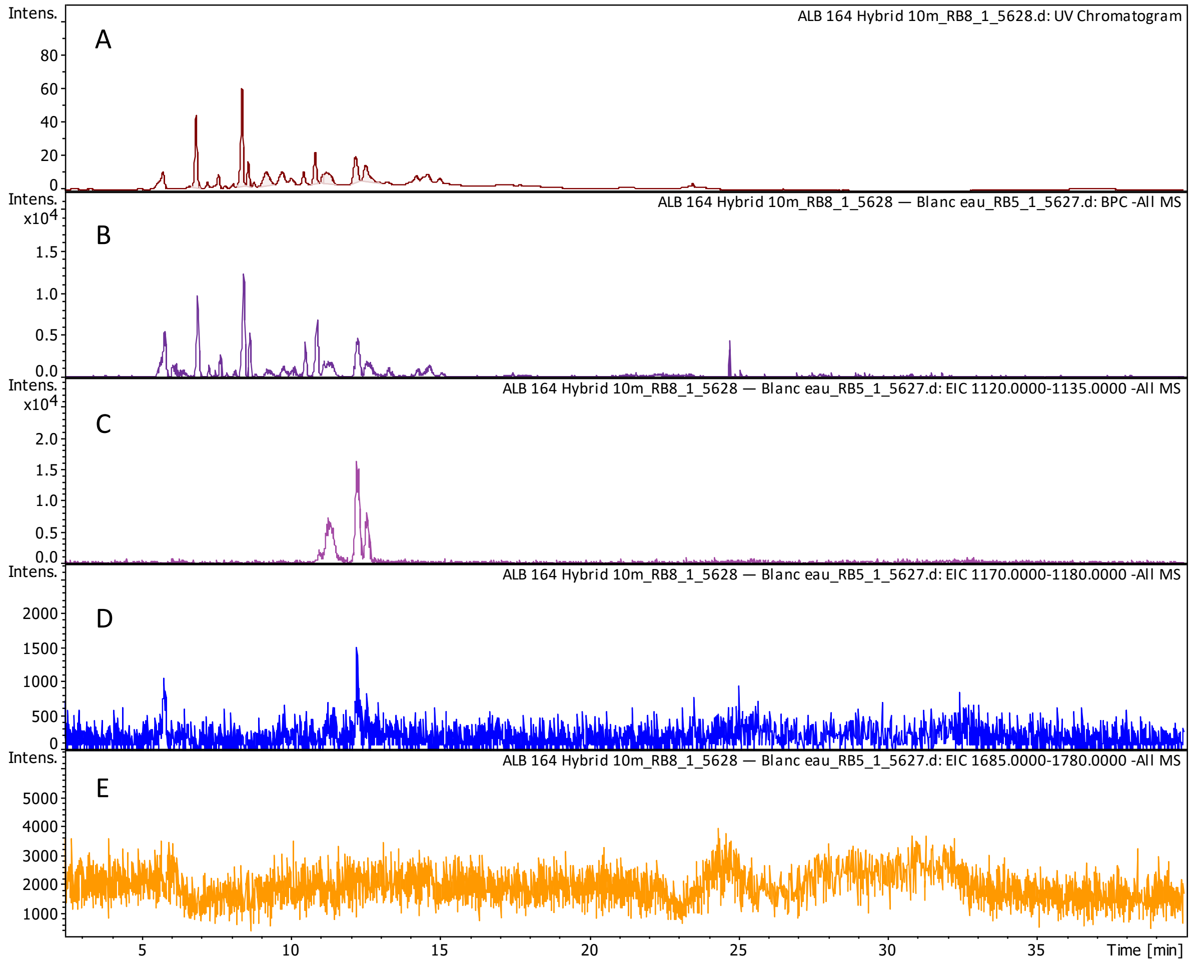

- Chromatogrammes (Haut : UV 260 nm, Bas : MS-)

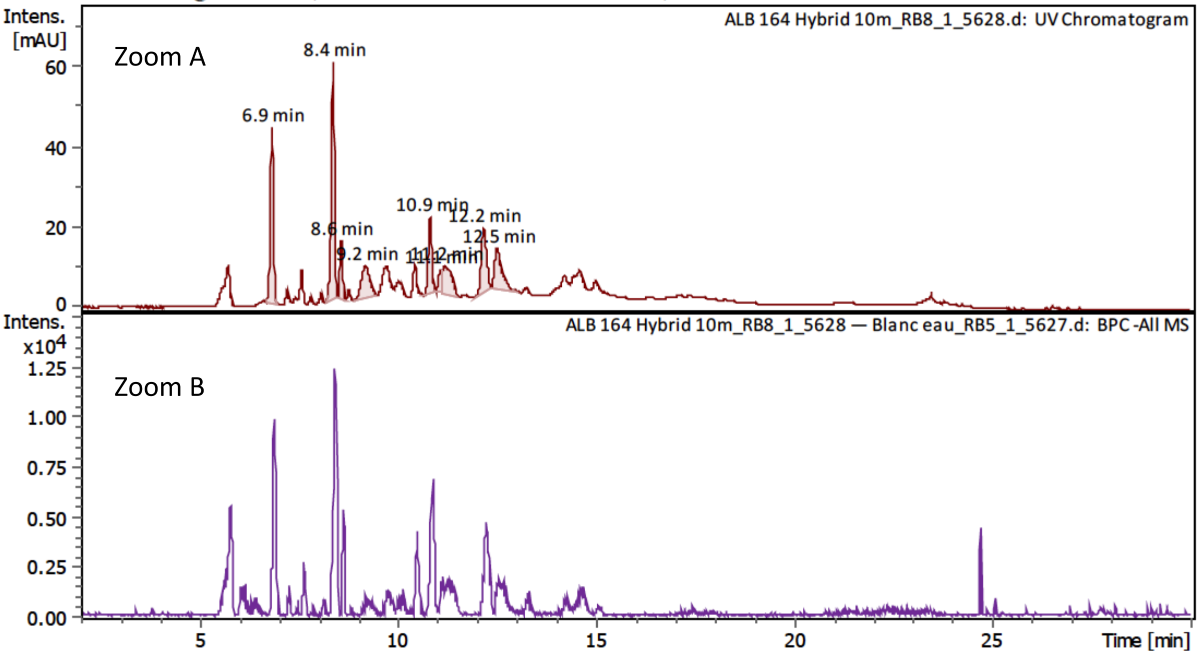

- Composé 1 – Tr 6.9 min

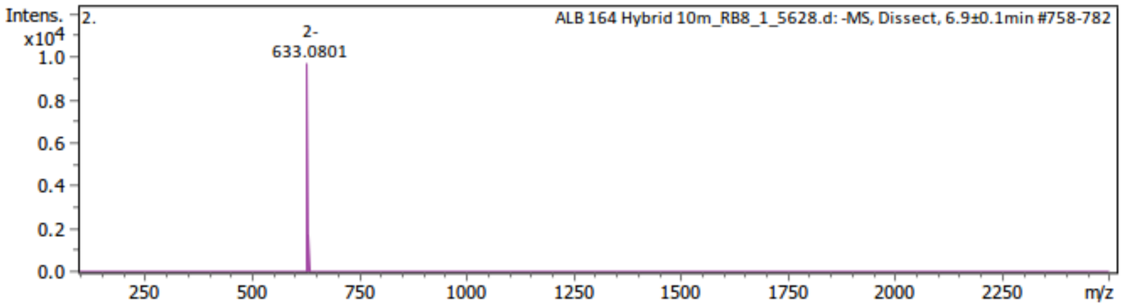

Meas. m/z	Ion Formula	Sum Formula	m/z	err [ppm]	mSigma	Adduct	z
633.0801	C39H46N14O27P4	C39H48N14O27P4	633.0809	1.3	21.6	M-H	2-

→ Le spectre de masse met en évidence la présence des adduits correspondant à un produit dont la formule brute est la suivante : **$C_{39}H_{46}N_{14}O_{27}P_4$.**

4H>p

- Composé 2 - Tr = 8.4 min

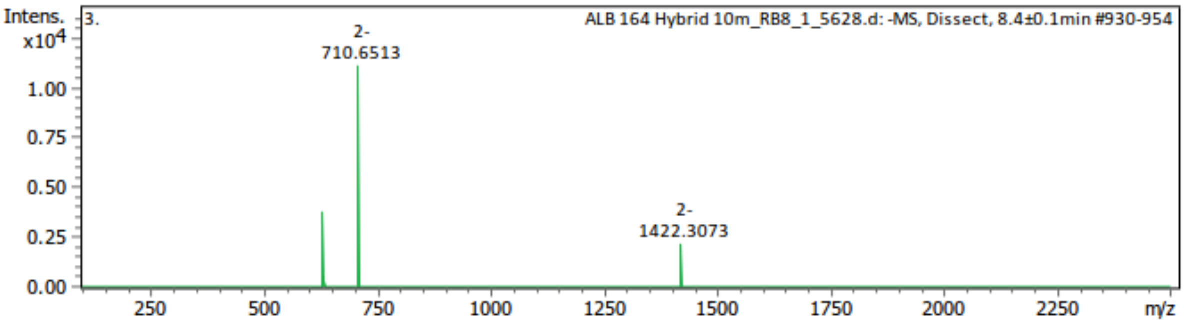

Zoom :

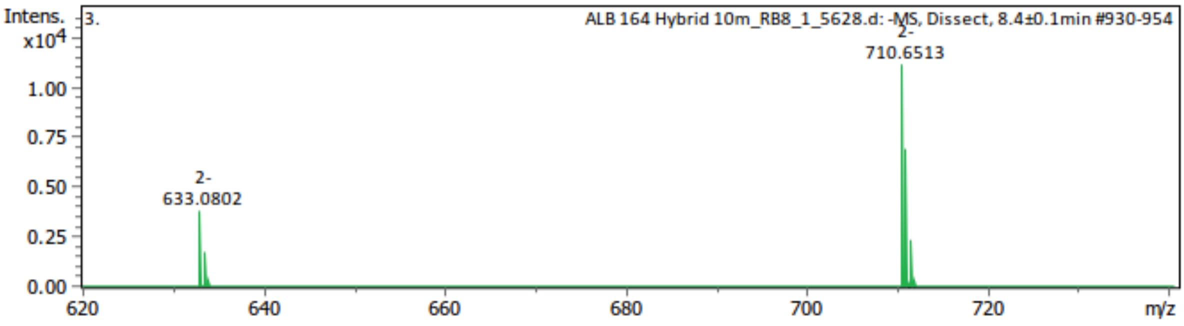

Mass observed: 1423.30

4H>p-EDC

- Composé 3 - Tr = 8.6 min

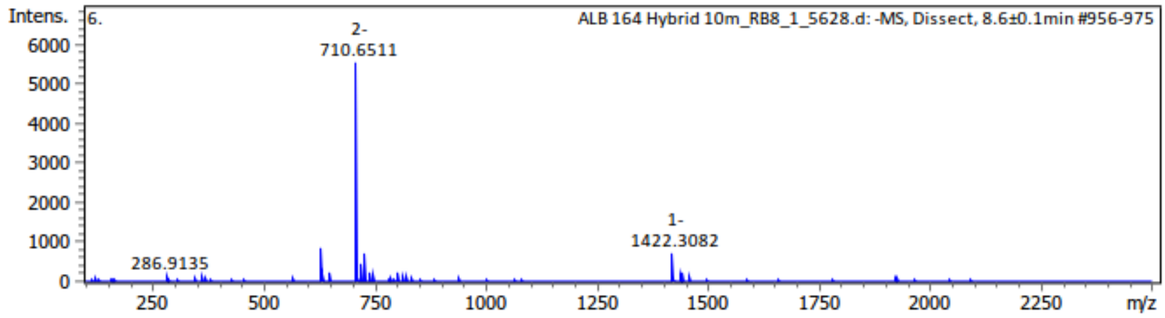

Zoom :

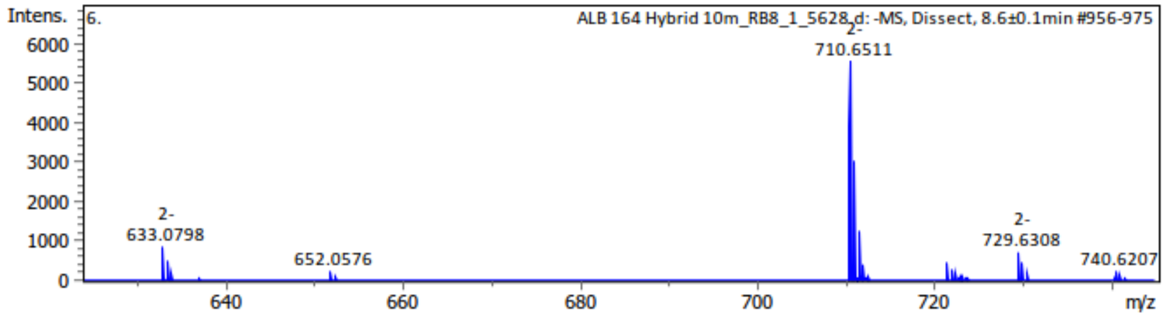

Mass observed: 1423.30

4H>p-EDC

- Composé 4 - Tr = 9.2 min

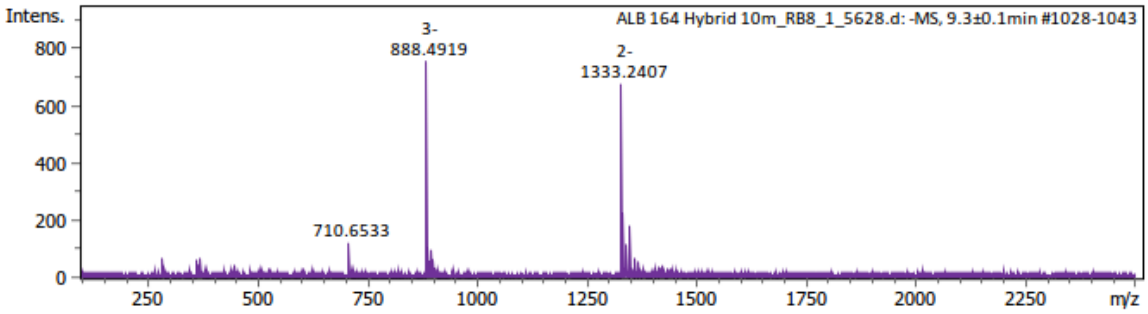

Meas. m/z	Ion Formula	Sum Formula	m/z	err [ppm]	mSigma	Adduct	z
888.1579	C85H106N35O50P8	C85H109N35O50P8	888.1582	0.3	37.7	M-H	3-

→ Le spectre de masse met en évidence la présence des adduits correspondant à un produit dont la formule brute est la suivante : **$C_{85}H_{109}N_{35}O_{50}P_8$.**

9D

- Composé 4’ - Tr = 10.9 min

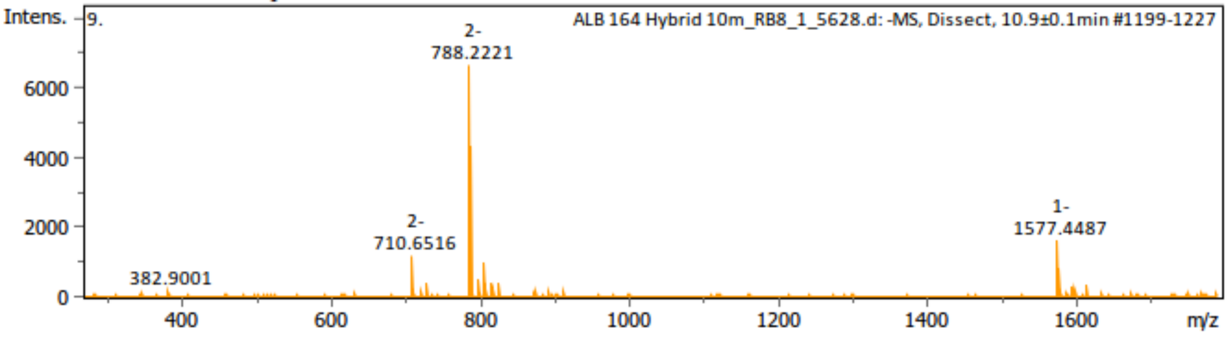

Mass observed: 1578.44

4H>p-EDC-EDC

- Composé 4’’ - Tr = 11.1 min

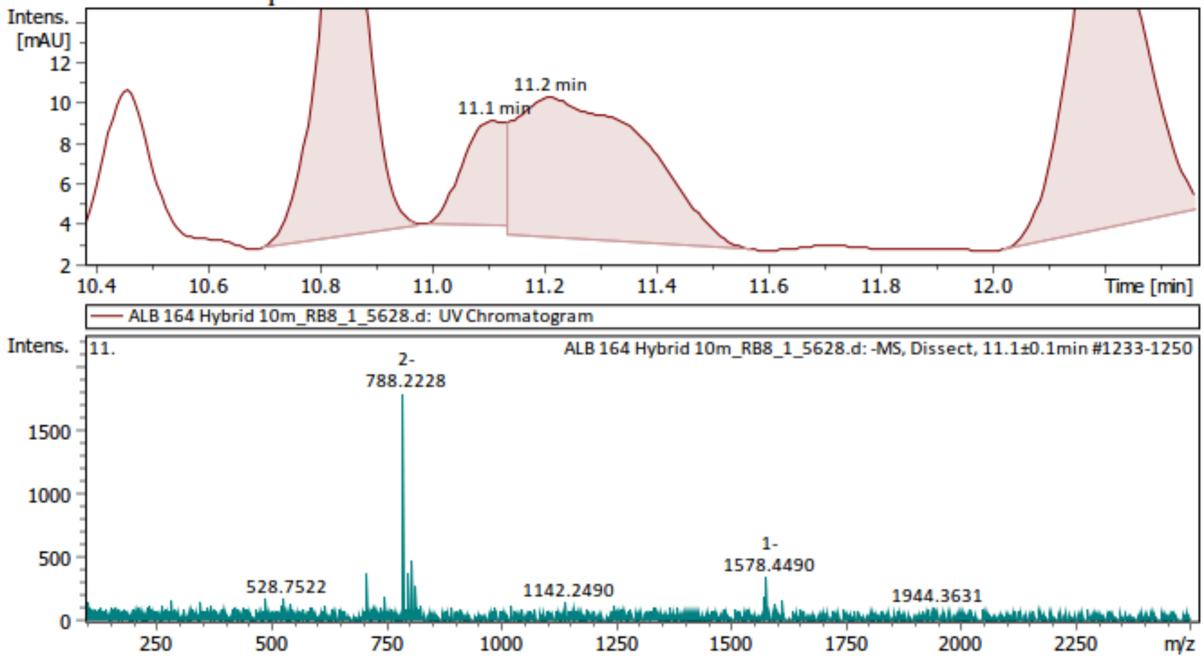

Mass observed: 1578.44

4H>p-EDC-EDC

- Composé 4’’’ - Tr = 11.2 min

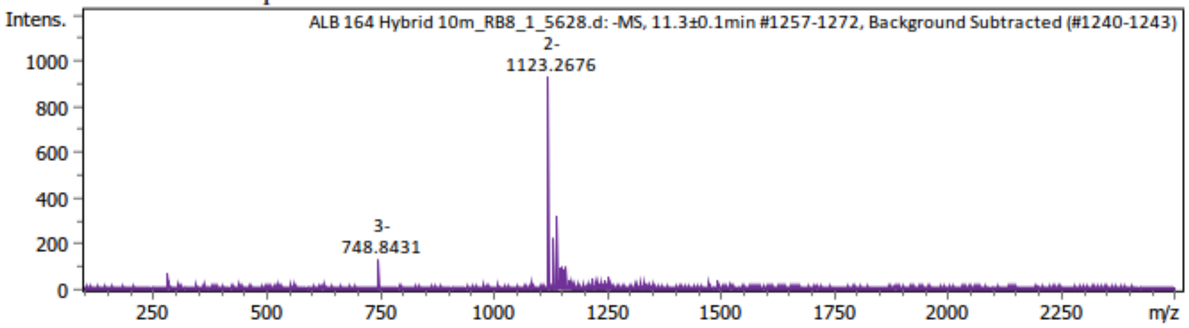

Mass observed: 2248.53

c5Dp-EDC

- Composé 5 – Tr = 12.2 min

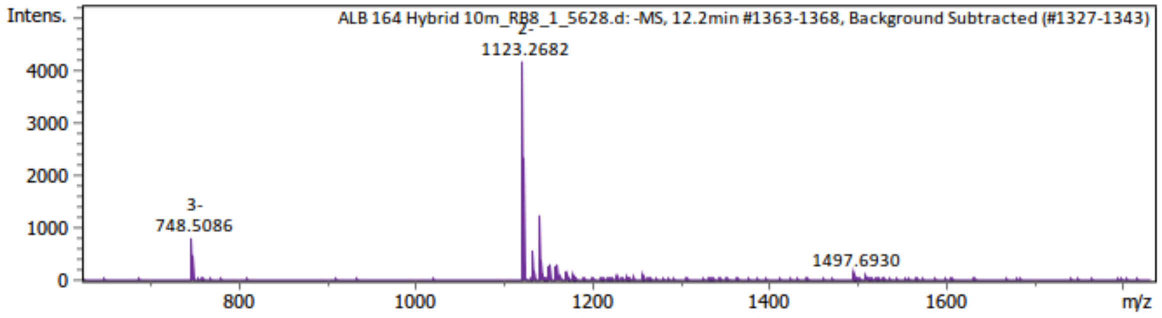

Mass observed: 2248.54

c5Dp-EDC

- Composé 6 - Tr = 12.5 min

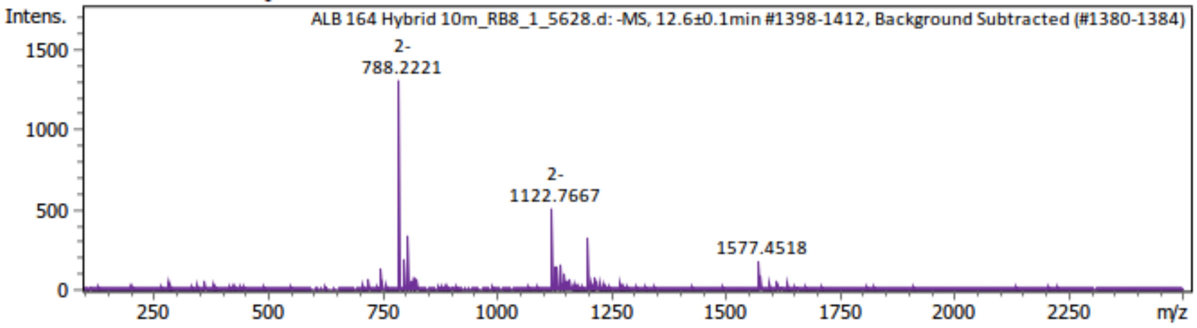

Mass observed: 1578.44

4H>p-EDC-EDC

Figure S42. E.H experiment after 10 months-incubation at 4-6°C. A) UV chromatogram (260 nm), B) all-negative-ion LC-MS chromatogram, picked (colour-filled) main peaks 1-6 annotated with their retention times (in Zoom A below), C-E) extracted ion chromatograms (EIC) for mass ranges of the possible products of ligation targeting C: $[9Hf{>}p]^{3-}$ and $[9Hfp]^{3-}$ (found at 11.2, 12.2 and 12.5 min compounds 4’’’, 5 and 6: $[c5Dp\text{-}EDC]^{2-}$ and $[4Hp\text{-}EDC\text{-}EDC]^{2-}$), D: $[9Hf{>}p\text{-}EDC]^{2-}$ and $[9Hfp\text{-}EDC]^{2-}$ (found 2 minor unidentified $[UI]^{2-}$ peaks of molecular mass < 3000 Da), E: $[9Hf{>}p]^{2-}$, $[9Hfp]^{2-}$, $[9Hf{>}p\text{-}EDC]^{2-}$, $[9Hfp\text{-}EDC]^{2-}$ and $[9Hfp\text{-}EDC\text{-}EDC]^{2-}$, followed by mass spectra of the annotated peaks, associated molecular masses (1-6) and identified compounds.

E.D experiment (10 months):

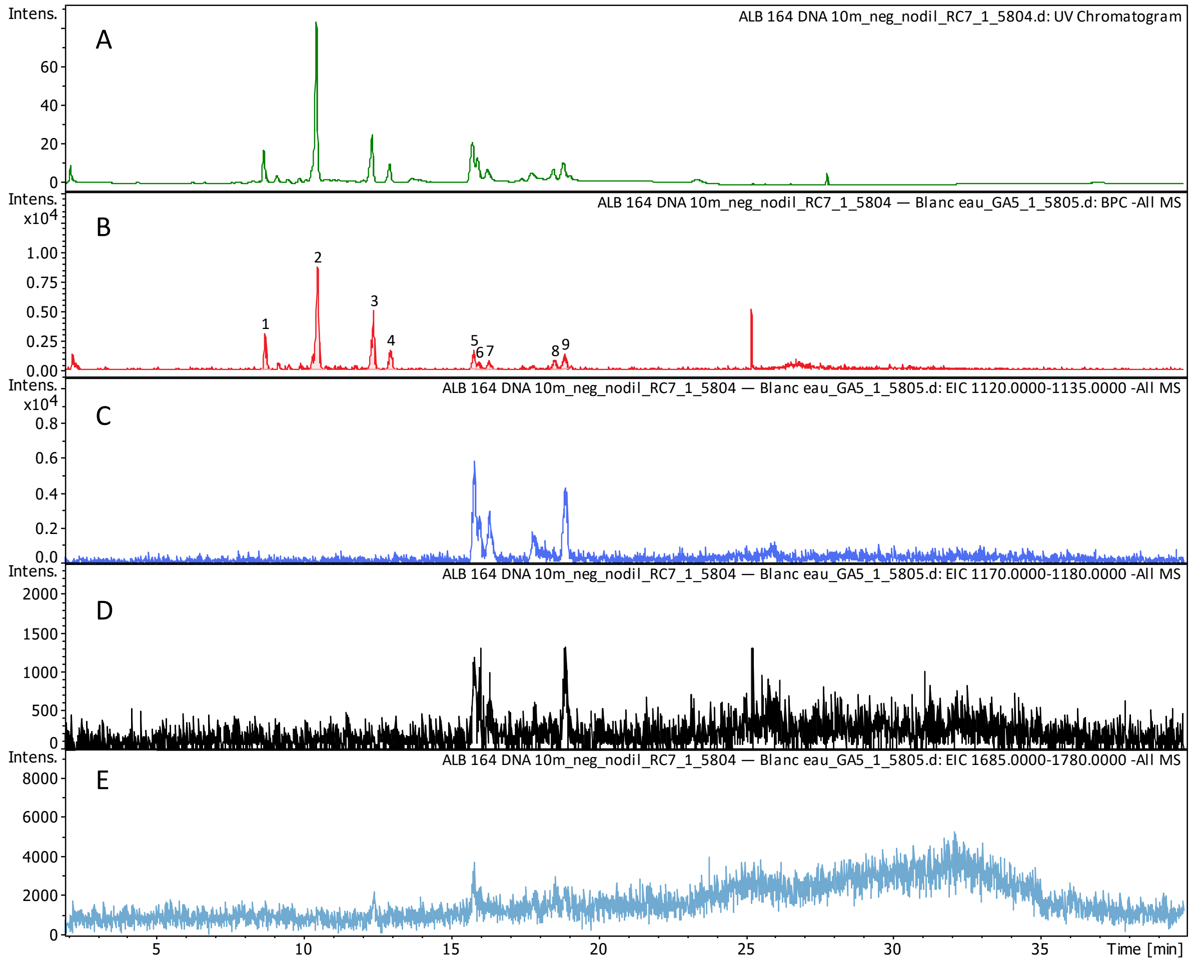

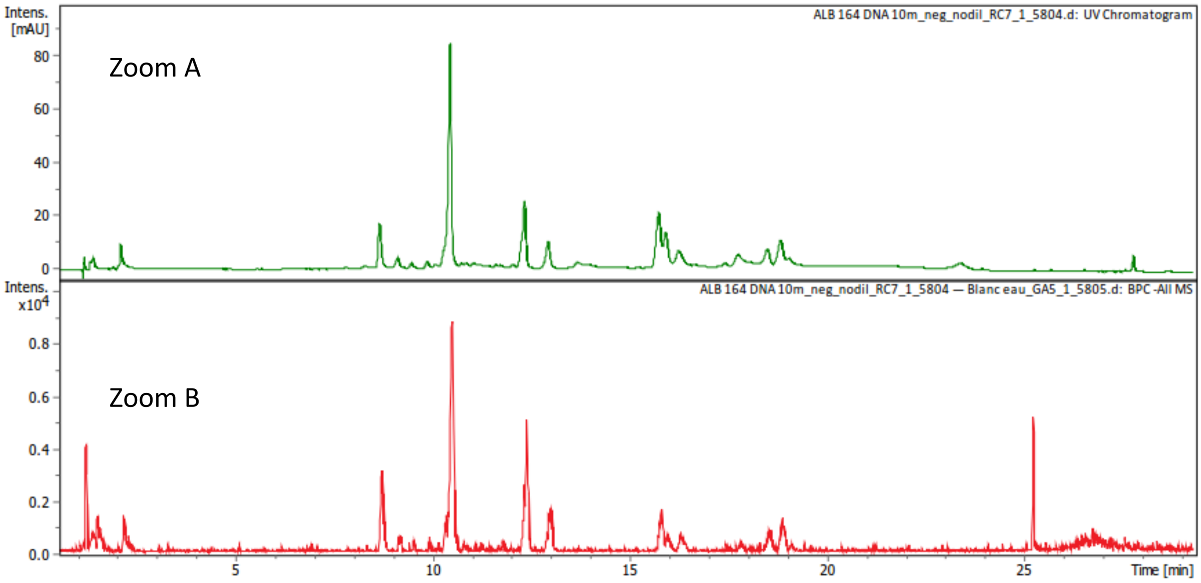

- Composé 1 - Tr = 8.7 min

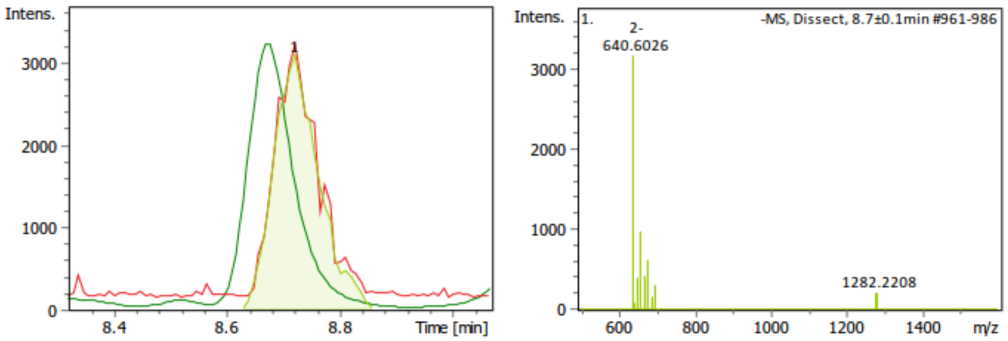

Meas. m/z	Ion Formula	Sum Formula	m/z	err [ppm]	mSigma	Adduct	z
640.6026	C45H51N13O24P4	C45H53N13O24P4	640.6066	6.2	24.7	M-H	2-
640.6026	C40H51N15O26P4	C40H53N15O26P4	640.6046	3.1	16	M-H	2-
640.6026	C39H55N11O30P4	C39H57N11O30P4	640.6039	2	20.3	M-H	2-
640.6026	C35H51N17O28P4	C35H53N17O28P4	640.6025	-0.1	29.9	M-H	2-
640.6026	C41H51N13O27P4	C41H53N13O27P4	640.5989	-5.7	15.5	M-H	2-
640.6026	C37H47N19O25P4	C37H49N19O25P4	640.5976	-7.8	20.8	M-H	2-
640.6026	C36H51N15O29P4	C36H53N15O29P4	640.5969	-8.8	27.8	M-H	2-

4Dp

- Composé 2 - Tr = 10.5 min

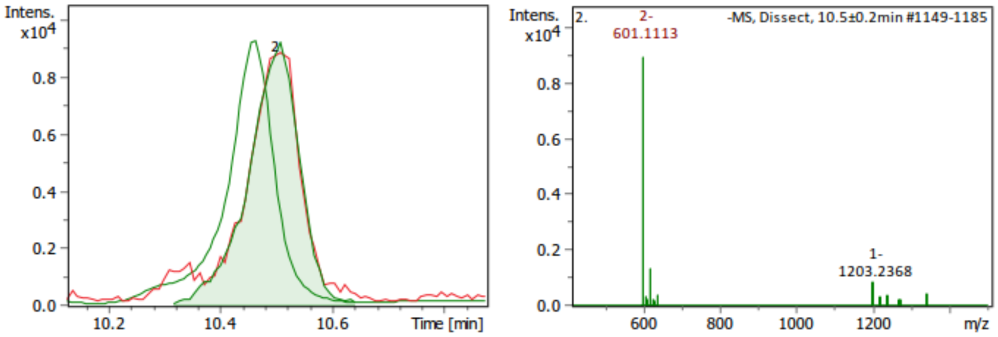

Meas. m/z	Ion Formula	Sum Formula	m/z	err [ppm]	mSigma	Adduct	z
601.1113	C38H54N12O25P4	C38H56N12O25P4	601.1142	4.8	27.3	M-H	2-
601.1113	C39H54N10O26P4	C39H56N10O26P4	601.1086	-4.5	24.9	M-H	2-
601.1113	C35H50N16O24P4	C35H52N16O24P4	601.1073	-6.7	37	M-H	2-

4D

- Composé 3 - Tr = 12.4 min

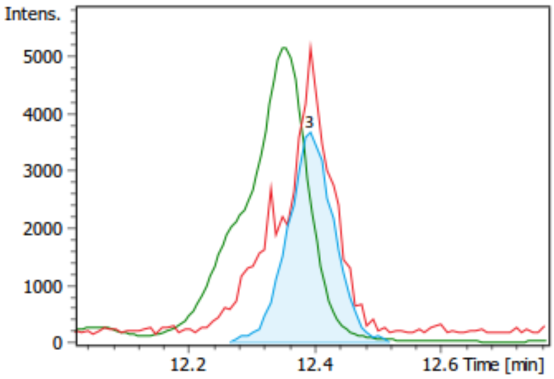

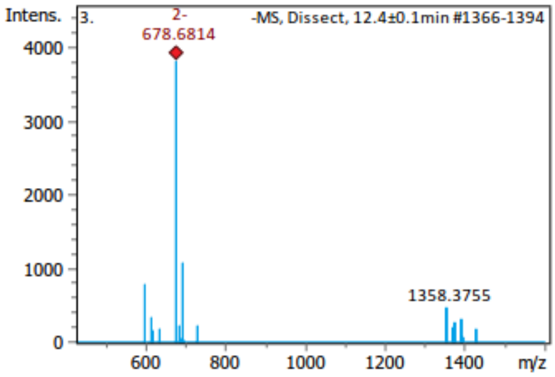

Soit M = 678.7*2+2=1359.4Da

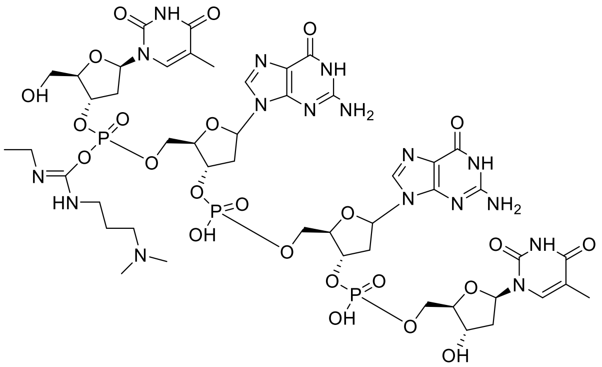

Chemical Formula: $C_{48}H_{68}N_{17}O_{24}P_3$
Exact Mass: 1359,38

4D-EDC

- Composé 4 - Tr = 13.0 min

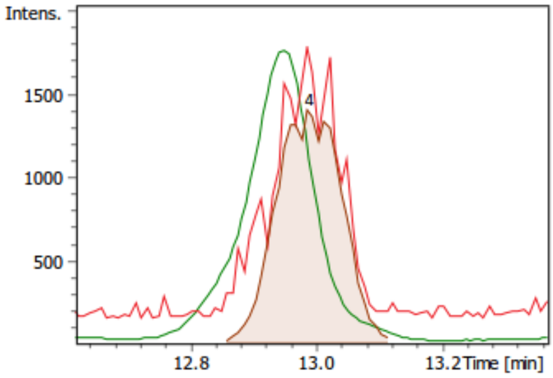

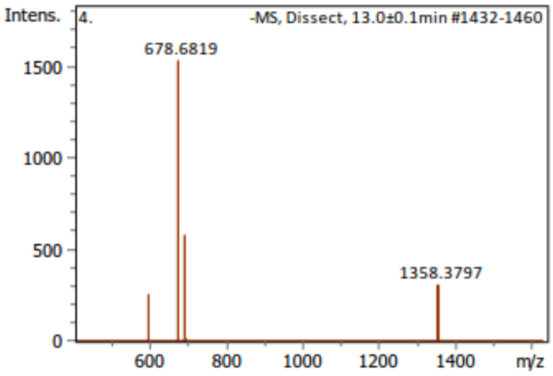

Soit M = 678.7*2+2=1359.4Da

4D-EDC

- Composé 5 - Tr = 15.8 min

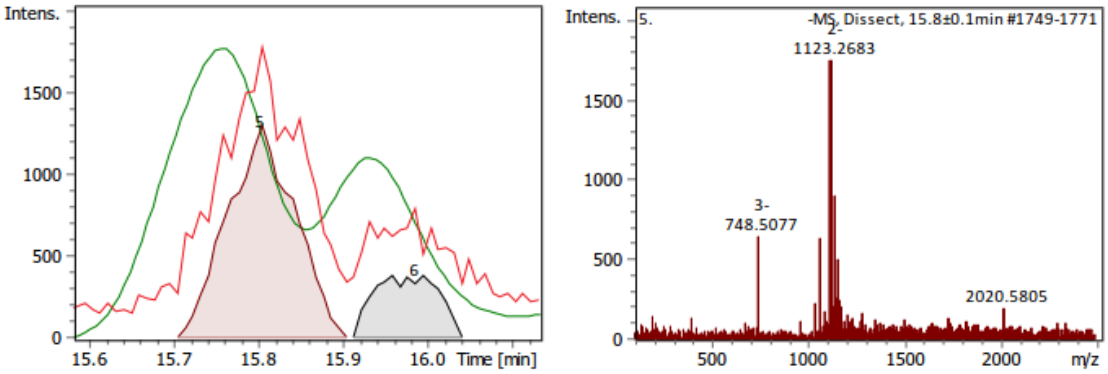

Soit M=1123.3*2+2=2248.6Da

c5Dp-EDC

- Composé 6 - Tr = 16.0 min

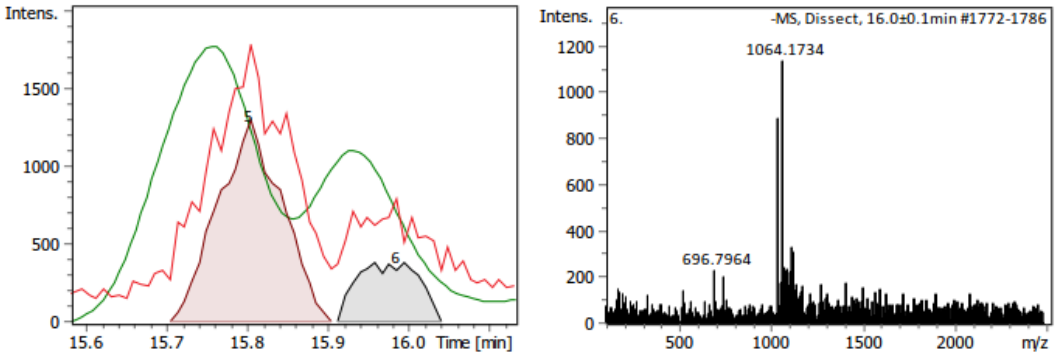

Mass observed: 2130.35

Unidentified derivative

- Composé 7 - Tr = 16.3 min

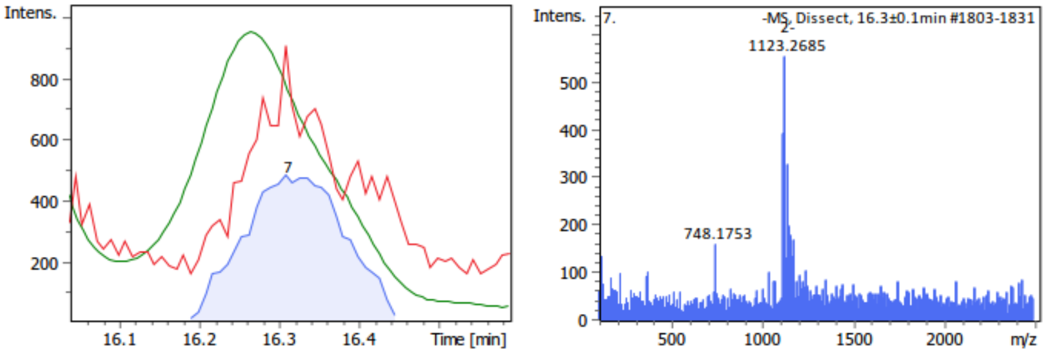

Soit M=1123.3*2+2=2248.6Da

c5Dp-EDC

- Composé 8 - Tr = 18.6 min

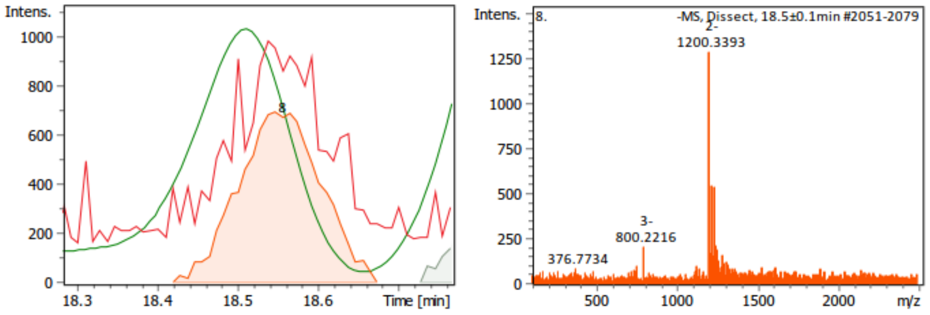

Soit M=1200.3*2+2=2402.6Da

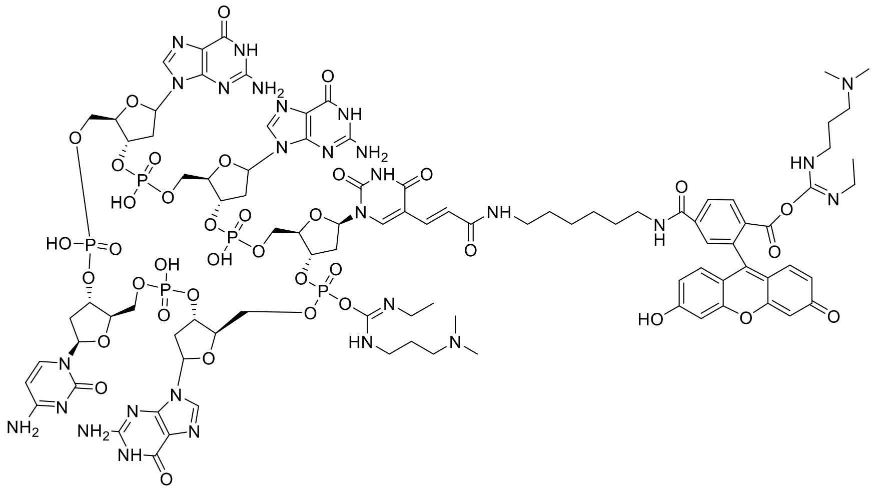

Chemical Formula: $C_{94}H_{119}N_{28}O_{38}P_5$
Exact Mass: 2402,69

c5Dp-EDC-EDC

- Composé 9 - Tr = 18.9 min

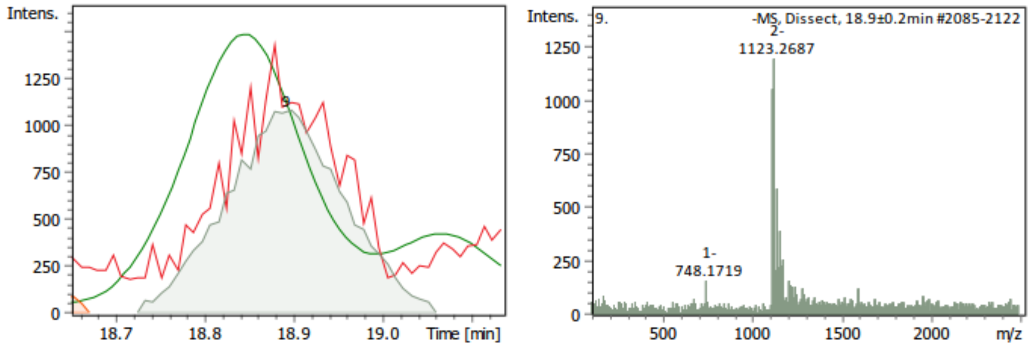

Soit M=1123.3*2+2=2248.6Da

c5Dp-EDC

Figure S43. E.D experiment after 10 months-incubation at 4-6°C. A) UV chromatogram (260 nm), B) all-negative-ion LC-MS chromatogram (main peaks annotated 1-9), C-E) extracted ion chromatograms (EIC) for mass ranges of the possible products of ligation targeting C: $[9Df>p]^{3-}$ and $[9Dfp]^{3-}$ (found in peak 9: $[c5Dp\text{-}EDC]^{2-}$, 13-17 min. several unidentified $[UI]^{2-}$), D: $[9Df>p\text{-}EDC]^{2-}$ and $[9Dfp\text{-}EDC]^{2-}$, E: $[9Df>p]^{2-}$, $[9Dfp]^{2-}$, $[9Df>p\text{-}EDC]^{2-}$, $[9Dfp\text{-}EDC]^{2-}$ and $[9Dfp\text{-}EDC\text{-}EDC]^{2-}$ (found several minor unidentified $[UI]^{2-}$ peaks of molecular mass < 3000 Da), followed by mass spectra of the annotated peaks, their molecular masses (1-9) and identified compounds (only one isomer shown).

Prebiotic vesicle preparation in E5/E5-like conditions:

A mixture of lipids containing LA, MLG, DLPA (1:1:1) was diluted in a flask in 1 mL of MeOH (5 mM total lipid concentration). The solvent was evaporated using a rotavapor and a bath at 40°C to form a thin lipid film. After dehydration overnight, the film was rehydrated overnight at room temperature with 1 mL of a buffer solution containing 100 mM NaCl, 250 mM EDC pH 6.0 (E-conditions) (± 5 mM $MgCl_2$ or $MnCl_2$) (E-like conditions). After hydration, the samples were supplemented with 250 mM of sulfo-NHS. Micrograph images were taken at each step using 3 µM of *Nile Red*™.

5) References

1. Jash, B.; Tremmel, P.; Jovanovic, D.; Richert, C. Single Nucleotide Translation without Ribosomes. *Nat. Chem.* **2021**, *13*, 751–757, https://doi.org/10.1038/s41557-021-00749-4.
2. Le Chevalier Isaad, A.; Carrara, P.; Stano, P.; Krishnakumar, K.S.; Lafont, D.; Zamboulis, A.; Buchet, R.; Bouchu, D.; Albrieux, F.; Strazewski, P. A Hydrophobic Disordered Peptide Spontaneously Anchors a Covalently Bound RNA Hairpin to Giant Lipidic Vesicles. *Org. Biomol. Chem.* **2014**, *12*, 6363–6373, https://doi.org/10.1039/C4OB00721B.
3. Eberle, F.; Gießler, K.; Deck, C.; Heeg, K.; Peter, M.; Richert, C.; Dalpke, A.H. Modifications in Small Interfering RNA that Separate Immunostimulation from RNA Interference. *J. Immunol.* **2008**, *180*, 3229–3237, https://doi.org/10.4049/jimmunol.180.5.3229.
4. Naylor, R.; Gilham, P.T. Studies on Some Interactions and Reactions of Oligonucleotides in Aqueous Solution. *Biochemistry* **1966**, *5*, 2722–2728, https://doi.org/10.1021/bi00872a032.
